# Pharmacological Investigation of Tongqiao Jiuxin Oil Against High-Altitude Hypoxia: Integrating Chemical Profiling, Network Pharmacology, and Experimental Validation

**DOI:** 10.3390/ph18081153

**Published:** 2025-08-02

**Authors:** Jiamei Xie, Yang Yang, Yuhang Du, Xiaohua Su, Yige Zhao, Yongcheng An, Xin Mao, Menglu Wang, Ziyi Shan, Zhiyun Huang, Shuchang Liu, Baosheng Zhao

**Affiliations:** 1 School of Chinese Materia Medica, Beijing University of Chinese Medicine, Beijing 102488, China; hbxiejiamei@163.com (J.X.); bucmyang2020@163.com (Y.Y.); 20230935235@bucm.edu.cn (Y.D.); zyg408716172@163.com (Y.Z.); hyss111@126.com (Y.A.); wml1251247317@163.com (M.W.); 15647039408@163.com (S.L.); 2 Guangzhou Baiyunshan Xingqun Pharmaceutical Co., Ltd., Guangzhou 510288, China; mxmaoxin@126.com (X.M.); huangzhiyunhzy@163.com (Z.H.); 3 School of Life Sciences, Beijing University of Chinese Medicine, Beijing 102488, China; 18645681606@163.com; 4 Beijing Research Institute of Chinese Medicine, Beijing University of Chinese Medicine, Beijing 100029, China

**Keywords:** Tongqiao Jiuxin Oil, acute mountain sickness, hypobaric hypoxia, network pharmacology, HIF-1α, traditional Chinese medicine

## Abstract

**Background**: Acute mountain sickness (AMS) is a prevalent and potentially life-threatening condition caused by rapid exposure to high-altitude hypoxia, affecting pulmonary and neurological functions. Tongqiao Jiuxin Oil (TQ), a traditional Chinese medicine formula composed of aromatic and resinous ingredients such as sandalwood, agarwood, frankincense, borneol, and musk, has been widely used in the treatment of cardiovascular and cerebrovascular disorders. Clinical observations suggest its potential efficacy against AMS, yet its pharmacological mechanisms remain poorly understood. **Methods:** The chemical profile of TQ was characterized using UHPLC-Q-Exactive Orbitrap HRMS. Network pharmacology was applied to predict the potential targets and pathways involved in AMS. A rat model of AMS was established by exposing animals to hypobaric hypoxia (~10% oxygen), simulating an altitude of approximately 5500 m. TQ was administered at varying doses. Physiological indices, oxidative stress markers (MDA, SOD, GSH), histopathological changes, and the expression of hypoxia- and apoptosis-related proteins (HIF-1α, VEGFA, EPO, Bax, Bcl-2, Caspase-3) in lung and brain tissues were assessed. **Results**: A total of 774 chemical constituents were identified from TQ. Network pharmacology predicted the involvement of multiple targets and pathways. TQ significantly improved arterial oxygenation and reduced histopathological damage in both lung and brain tissues. It enhanced antioxidant activity by elevating SOD and GSH levels and reducing MDA content. Mechanistically, TQ downregulated the expression of HIF-1α, VEGFA, EPO, and pro-apoptotic markers (Bax/Bcl-2 ratio, Caspase-3), while upregulated Bcl-2, the anti-apoptotic protein expression. **Conclusions**: TQ exerts protective effects against AMS-induced tissue injury by improving oxygen homeostasis, alleviating oxidative stress, and modulating hypoxia-related and apoptotic signaling pathways. This study provides pharmacological evidence supporting the potential of TQ as a promising candidate for AMS intervention, as well as the modern research method for multi-component traditional Chinese medicine.

## 1. Introduction

Acute mountain sickness (AMS) is a common clinical syndrome that arises from rapid ascent to high altitudes, typically above 2500 m, and is characterized by symptoms such as headache, dizziness, fatigue, gastrointestinal discomfort, and sleep disturbance [1,2]. Epidemiological studies report that 25–40% of individuals develop AMS at 3000 m, and the incidence can exceed 75% at elevations above 4000 m in unacclimatized populations [3,4]. If left untreated, AMS may progress to more severe forms, including high-altitude cerebral edema (HACE) or high-altitude pulmonary edema (HAPE), which are potentially fatal [5].

One of the key molecular mechanisms involved in the pathogenesis of AMS is hypoxia-induced stabilization of hypoxia-inducible factor-1α (HIF-1α), a transcription factor that mediates cellular adaptation to low oxygen environments. Under hypoxic stress, HIF-1α activates downstream genes such as vascular endothelial growth factor A (VEGFA) and erythropoietin (EPO), which promote angiogenesis and erythropoiesis, respectively [6,7]. However, excessive or sustained HIF-1α activation can also contribute to oxidative stress, mitochondrial dysfunction, and apoptosis, particularly via the B-cell lymphoma-2 (Bcl-2)/Bcl-2-Associated X (Bax) axis and Caspase-3 activation [8,9]. Thus, the HIF-1α/VEGFA/EPO axis and Caspase-3-mediated apoptosis are considered critical molecular mechanisms in the development and progression of high-altitude illness.

Tongqiao Jiuxin Oil (TQ) is a traditional Chinese medicine formulation composed of aromatic and resinous compounds such as borneolum syntheticum, agarwood, musk, sandalwood, frankincense, storax, L-menthol, and camphor [10]. It has been widely applied in treating cardiovascular and cerebrovascular conditions in clinical practice. According to traditional Chinese medicine (TCM) theory, TQ functions by “opening orifices, promoting Qi circulation, and restoring consciousness”, aligning with the pathophysiological features of AMS, including Qi stagnation and impaired oxygen delivery. For example, borneol has been shown to cross the blood–brain barrier, reduce oxidative stress and caspase-3-mediated apoptosis, and modulate neuroinflammation in cerebral ischemia models [11].

Despite these indications, the pharmacodynamic effects and molecular mechanisms of TQ in the context of AMS have not been systematically investigated. Therefore, this study aimed to characterize the chemical constituents of TQ, predict its putative targets and pathways using network pharmacology, and validate its efficacy in a rat model of AMS. Special focus was placed on oxidative stress, hypoxia signaling (HIF-1α/VEGFA/EPO), and mitochondrial apoptosis-related proteins (Caspase-3, Bax, Bcl-2) to provide mechanistic insights into TQ’s therapeutic potential in high-altitude environments.

## 2. Results

### 2.1. Identification of Chemical Constituents in TQ

**Figure 1:**
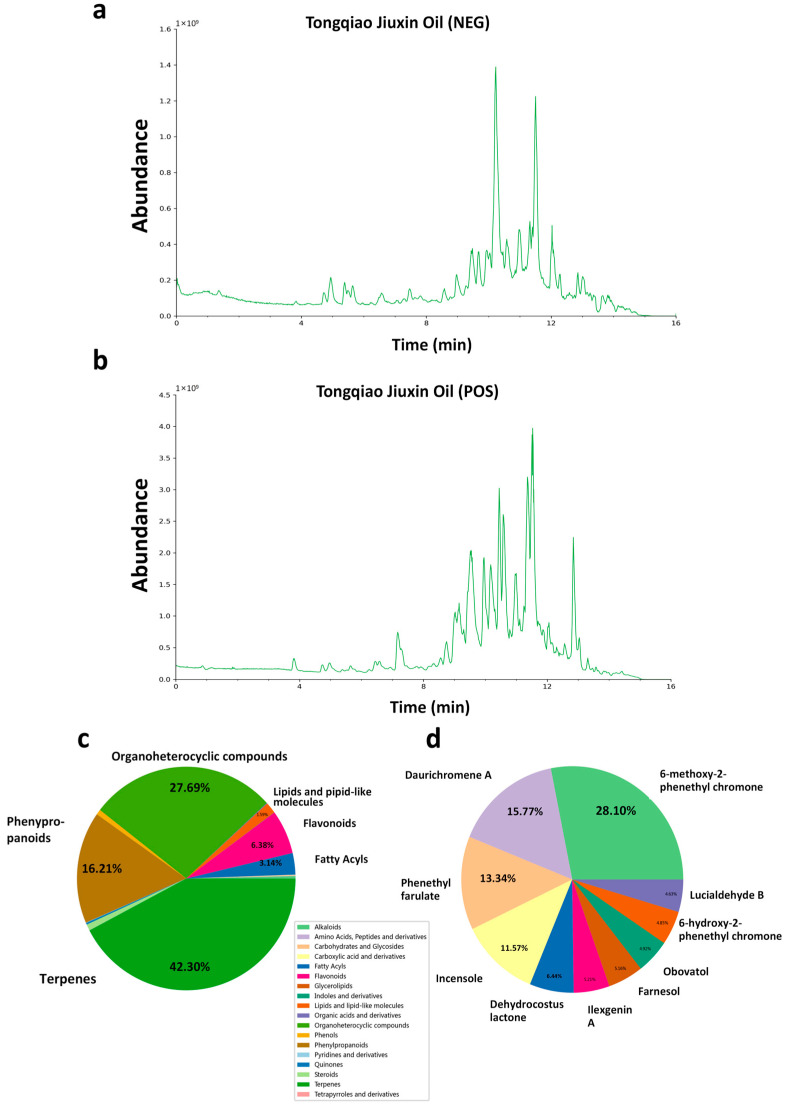
Chemical profiling of TQ using UHPLC-Q-Exactive Orbitrap HRMS. (**a**,**b**) TIC of TQ in positive ion mode. (**c**) Classification of compounds identified in TQ based on chemical categories. Terpenes were the most abundant class, followed by organoheterocyclic compounds and phenylpropanoids. (**d**) The top ten chemical constituents in the methanolic extract of TQ, ranked by relative abundance based on peak area.

To comprehensively evaluate the chemical composition of the methanolic extract of TQ, ultra-high-performance liquid chromatography coupled with UHPLC-Q-Exactive Orbitrap HRMS was employed. As shown in Figure 1a,b, the total ion chromatograms (TICs) were obtained under both ESI− and ESI+ ionization modes. In total, 774 chemical constituents were identified in the methanolic extract of TQ. These primarily include 219 terpenoids, 78 phenylpropanoids, 63 organic heterocycles, 62 fatty acyl compounds, 60 flavonoids, 26 phenols, 23 steroids, 10 alkaloids, 8 saccharides and glycosides, 6 quinones, and 6 organic acids and their derivatives (Figure 1c). Figure 1d shows the top ten chemical constituents of the methanolic extract of TQ based on peak area, along with their relative abundances. The most abundant compound was 6-methoxy-2-phenethyl chromone. Further compound structures are provided in the Supplementary Materials. Detailed mass spectrometry data are provided in Table S1.

### 2.2. Network-Based Identification of AMS-Related Targets and Pathways

**Figure 2:**
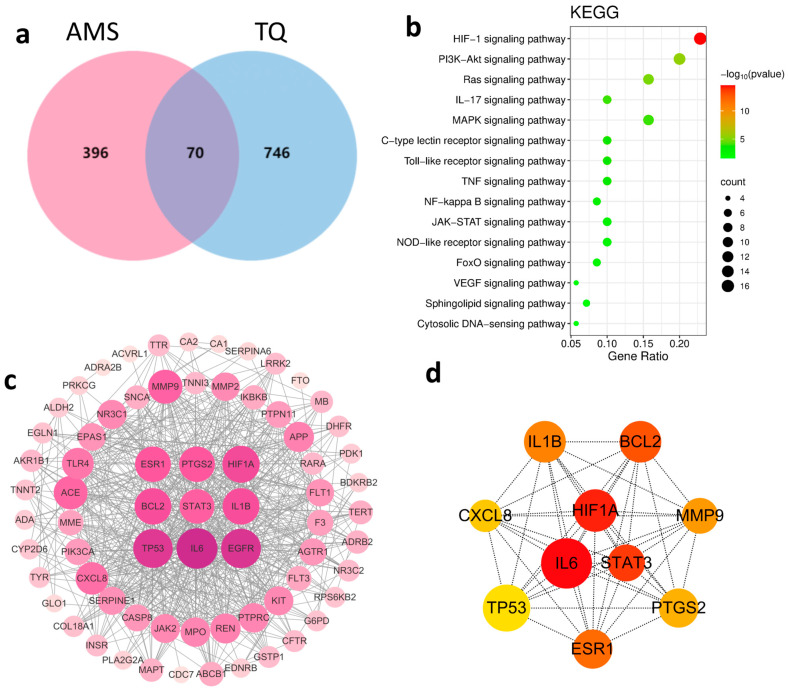

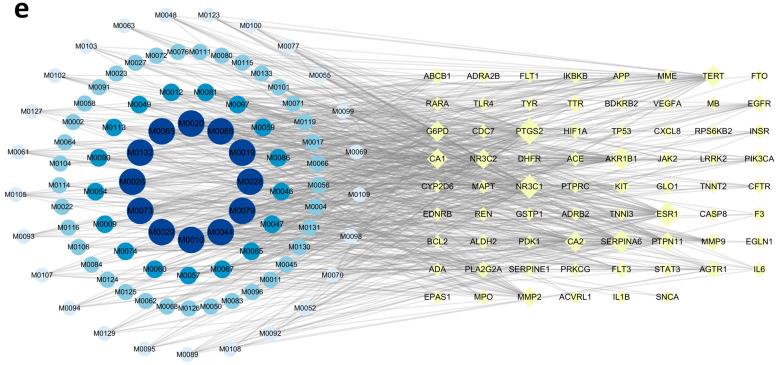
Network pharmacology analysis of TQ in the treatment of AMS. (**a**) Venn diagram showing the overlapping targets between AMS-related genes and TQ-related targets. (**b**) KEGG pathway enrichment analysis of the 70 overlapping targets. The top 15 enriched signaling pathways are shown. (**c**) PPI network of overlapping targets. Node size and color reflect degree centrality, with hub genes highlighted. (**d**) Core gene subnetwork extracted from the PPI network, identifying key genes with the highest degree values. (**e**) Compound–target interaction network. Blue nodes represent active compounds, yellow nodes indicate potential therapeutic targets, and gray edges denote predicted interactions. For clarity of presentation, chemical components are labeled with numbers in the figure; detailed information is provided in Table S1.

SwissTargetPrediction was used to predict potential targets for 134 identified primary compounds in TQ. After removing duplicates, a total of 816 unique targets were obtained. Meanwhile, using “acute mountain sickness” as the keyword, 466 unique disease-related targets were retrieved from the OMIM and GeneCards databases after de-duplication. Venn analysis revealed 70 overlapping targets, which were considered to be potential therapeutic targets of TQ for AMS (Figure 2a).

To explore the underlying mechanisms, Kyoto Encyclopedia of Genes and Genomes (KEGG) pathway enrichment analysis of the 70 intersecting targets was conducted using the DAVID database. The top 15 significantly enriched pathways were identified (Figure 2b), among which the HIF-1 signaling pathway and VEGF signaling pathway are closely associated with hypoxia.

A protein–protein interaction (PPI) network was then constructed using Cytoscape 3.10.0, consisting of 70 nodes and 625 edges (Figure 2c). The top 10 hub genes were identified by the cytoHubba plugin based on the Maximal Clique Centrality (MCC) algorithm (Figure 2d). The results highlighted the critical roles of HIF-1α, inflammatory cytokines, and apoptosis-related proteins in the pathogenesis of AMS. The compound–target interaction network is shown in Figure 2e.

### 2.3. TQ Improves Blood Gas Parameters, Hematological Profiles, and Oxidative Stress Markers in AMS Rats

**Figure 3:**
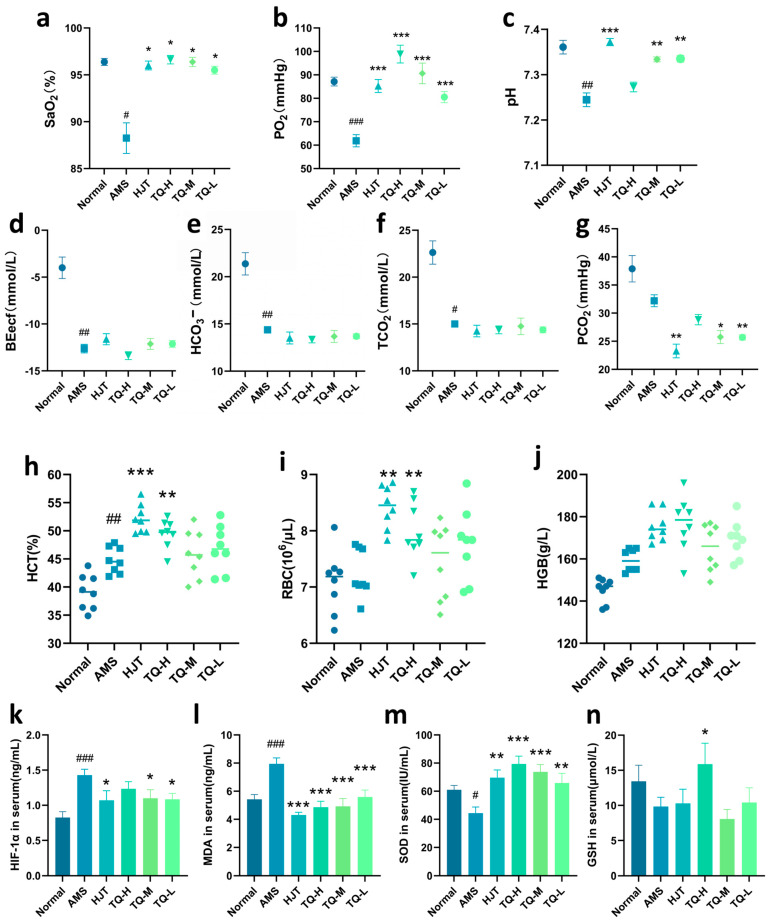
Effects of TQ on blood gas parameters, hematological indices, and serum biochemical markers in AMS rats. (**a**–**g**) TQ improved SaO_2_, PO_2_, and pH, reduced PCO_2_, and had no apparent effect on BEecf, HCO_3_^−^, and TCO_2_ levels. (**h**–**j**) TQ helped restore HCT and RBC, with slight changes in HGB. (**k**–**n**) AMS elevated HIF-1α and MDA, and reduced SOD and GSH. TQ significantly improved these serum biomarkers. Data are presented as mean ± SEM (*n* = 8). ^#^ *p* < 0.05, ^##^ *p* < 0.01, ^###^ *p* < 0.001 vs. Normal group; * *p* < 0.05, ** *p* < 0.01, *** *p* < 0.001 vs. AMS group.

As illustrated in Figure 3a–f, blood gas analysis showed that the AMS group exhibited marked reductions in oxygen saturation (SaO_2_), partial pressure of oxygen (PO_2_), pH, base excess in the extracellular fluid compartment (BEecf), bicarbonate ion concentration (HCO_3_^−^), and total carbon dioxide (TCO_2_) compared to the Normal group. Although partial pressure of carbon dioxide (PCO_2_) levels also declined, the changes were not considered meaningful (Figure 3g). In the HJT-treated group, PO_2_, SaO_2_, and pH were noticeably elevated, accompanied by a distinct decrease in PCO_2_, whereas HCO_3_^−^ and TCO_2_ remained relatively stable. Similarly, the TQ-H, TQ-M, and TQ-L groups demonstrated enhanced PO_2_ and SaO_2_ levels. Among these, TQ-M and TQ-L treatments also led to a clear increase in pH and a reduction in PCO_2_. However, BEecf, HCO_3_^−^, and TCO_2_ levels appeared unaffected by TQ administration.

In terms of hematological parameters (Figure 3h–j), the AMS group showed a pronounced elevation in HCT compared with the Normal group, while red blood cell count (RBC) and hemoglobin (HGB) exhibited a mild upward trend with no evident differences. Administration of HJT resulted in a noticeable rise in both hematocrit (HCT) and RBC levels relative to AMS. Likewise, the TQ-H group demonstrated increases in RBC and HCT, although HGB showed only a modest, non-conclusive elevation. In contrast, no appreciable alterations in HGB, HCT, or RBC were observed in the TQ-M and TQ-L groups.

As for serum biochemistry (Figure 3k–n), malondialdehyde (MDA) and HIF-1α levels were clearly elevated in the AMS group, whereas superoxide dismutase (SOD) was diminished, and glutathione (GSH) exhibited a downward tendency that did not reach a reliable threshold. Treatment with HJT led to a substantial decline in MDA and HIF-1α levels, along with a notable enhancement of SOD activity and a slight increase in GSH. All TQ-treated groups showed an evident upregulation of SOD activity and suppression of MDA levels. Furthermore, HIF-1α was reduced in both the TQ-M and TQ-L groups, and a discernible rise in GSH content was observed in the TQ-H group.

### 2.4. Organ Protective Effects of TQ in AMS Rats

**Figure 4:**
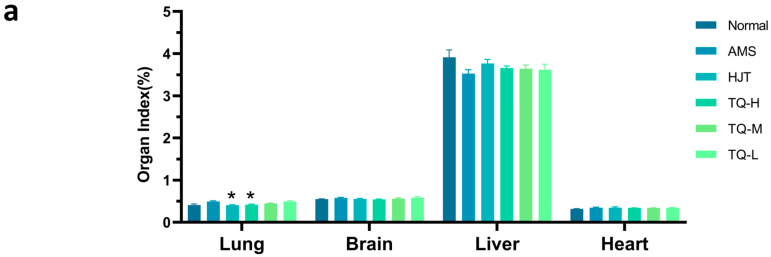

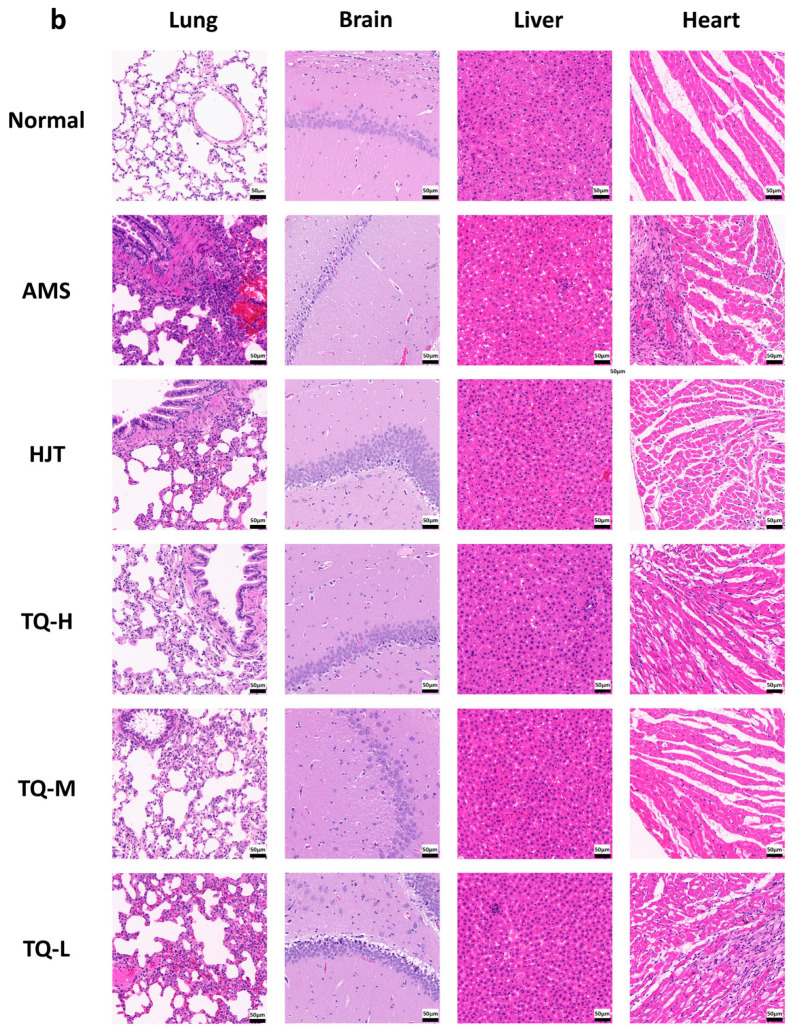
Effects of TQ on organ indices and histological changes in AMS rats. (**a**) Organ indices (%) of lung, brain, liver, and heart across different groups. Data are shown as mean ± SEM (*n* = 8). * *p* < 0.05 vs. AMS group. (**b**) Representative H&E-stained sections of lung, brain, liver, and heart (scale bar = 50 μm).

Compared with the Normal group, the AMS group exhibited an upward trend in lung index (Figure 4a). Following treatment, lung indices were reduced in the HJT and TQ-H groups and also showed a declining tendency in the TQ-M and TQ-L groups; however, none of these differences reached statistical significance. Similarly, AMS rats showed a decreasing trend in the liver index and an increase in the brain index relative to controls, both of which were modestly attenuated by drug interventions, albeit without statistical significance. No significant changes in the heart index were observed among the groups.

Histological examination of lung tissue (Figure 4b) revealed a well-preserved alveolar architecture in the Normal group, with thin alveolar walls, no exudate, and an absence of congestion or inflammatory cell infiltration. In contrast, AMS rats displayed marked pathological changes, including congestion, interstitial edema, thickened alveolar walls and septa, epithelial hyperplasia, and substantial neutrophil infiltration. These alterations were partially mitigated in the HJT, TQ-H, and TQ-M groups, which exhibited mild interstitial thickening and limited inflammatory infiltration. In the TQ-L group, moderate thickening of alveolar walls and septa, epithelial proliferation, and neutrophil infiltration persisted.

In the brain, normal neuronal morphology with clearly visible nucleoli was observed in the Normal group. AMS exposure led to cytoplasmic edema, nuclear pyknosis, and widening of intercellular spaces. These pathological features were partially reversed in the HJT, TQ-H, and TQ-M groups, while the TQ-L group showed no appreciable improvement.

Hepatic tissue in the Normal group exhibited radially arranged hepatocytes surrounding the central vein with clear cellular boundaries. AMS induction resulted in disrupted lobular architecture, hepatocyte swelling, and inflammatory infiltration. These abnormalities were alleviated in the HJT, TQ-H, and TQ-M groups, which displayed only mild structural disorganization and swelling with minimal inflammation. In contrast, the TQ-L group continued to exhibit hepatocellular swelling and scattered inflammatory cell infiltration.

Myocardial tissue from the Normal group showed well-aligned fibers with no signs of degeneration or inflammation. In the AMS group, myocardial disarray, cellular necrosis, interstitial edema, and inflammatory infiltration were evident. These pathological changes were markedly attenuated in the HJT and TQ-H groups, while the TQ-M and TQ-L groups presented mild fiber disarray, slight edema, and occasional inflammatory cell infiltration.

### 2.5. Effects of TQ on Hypoxia and Apoptosis-Related Proteins in the Lung and Brain of AMS Rats

Based on network pharmacology analysis and prior studies, the HIF-1α/VEGFA/EPO axis was identified as a key regulatory pathway in AMS. Additionally, hypoxia has been reported to trigger apoptosis via multiple signaling cascades [8,9]. To explore these mechanisms, we evaluated the expression levels of HIF-1α, VEGFA, EPO, Caspase-3, Bax, and Bcl-2 proteins in lung and brain tissues of AMS rats using Western blot analysis.

**Figure 5:**
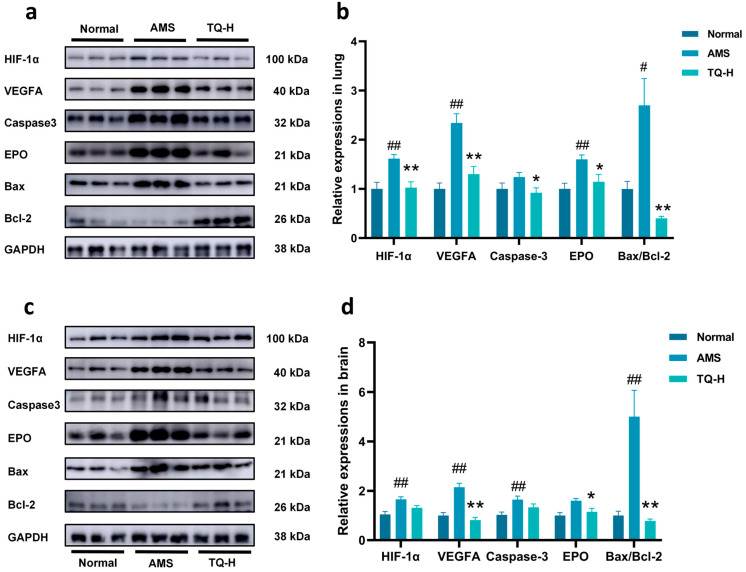
Effects of TQ on hypoxia- and apoptosis-related proteins in the lung and brain tissues of AMS rats. (**a**,**b**) Western blot analysis of HIF-1α, VEGFA, EPO, Caspase-3, Bax, and Bcl-2 expression in lung tissue. (**c**,**d**) Western blot analysis of the same proteins in brain tissue. Protein levels were normalized to GAPDH and are presented as relative expression. Data are shown as mean ± SEM (*n* = 3). ^#^ *p* < 0.05, ^##^ *p* < 0.01 vs. Normal group; * *p* < 0.05, ** *p* < 0.01 vs. AMS group.

As shown in Figure 5a,b, the AMS group exhibited significantly elevated expression of HIF-1α, VEGFA, and EPO and an increased Bax/Bcl-2 ratio in lung tissue compared to the Normal group. Notably, TQ-H treatment markedly reduced the expression of HIF-1α, VEGFA, Caspase-3, EPO, and the Bax/Bcl-2 ratio.

Similarly, in the brain (Figure 5c,d), AMS rats showed significantly increased levels of HIF-1α, VEGFA, Caspase-3, and Bax/Bcl-2 proteins relative to the Normal group. Treatment with TQ-H significantly suppressed the expression of VEGFA, EPO, and the Bax/Bcl-2 ratio compared to the AMS group.

## 3. Discussion

In addition to typical clinical manifestations, AMS caused by hypobaric hypoxia also induces systemic damage to highly oxygen-dependent organs such as the lungs and brain at the molecular and cellular levels. Studies have shown that AMS is associated with prominent oxidative stress, characterized by excessive reactive oxygen species (ROS) production, decreased activities of SOD and GSH peroxidase, and increased lipid peroxidation. These changes trigger nuclear factor kappa-B (NF-κB) activation and the release of pro-inflammatory cytokines, resulting in increased vascular permeability and the development of edematous lesions [12]. Meanwhile, HIF-1α is stabilized under hypoxic conditions and mediates adaptive responses. However, sustained HIF-1α activation also contributes to mitochondrial apoptotic pathways, as evidenced by increased Bax/Bcl-2 ratios and Caspase-3 activation, leading to neuronal and alveolar cell apoptosis [13,14]. These mechanisms explain the rapid progression and complications of AMS and highlight the need for pharmacological strategies targeting oxidative stress, inflammation, and apoptosis.

Oxidative stress plays a central role in the pathophysiology of AMS. Under hypobaric hypoxia, the excessive generation of ROS leads to lipid peroxidation, indicated by elevated MDA and depletion of endogenous antioxidants such as SOD and GSH. Both human and animal studies consistently report increased MDA and decreased antioxidant enzyme activities following acute high-altitude exposure, correlating with oxidative damage in the lung and brain, mitochondrial dysfunction, and exacerbated AMS symptoms [15]. In an animal study, pulmonary MDA levels were significantly elevated in rats after just 6 h of hypoxic exposure [16]. Additionally, clinical investigations have demonstrated that exposure to hypobaric hypoxia leads to a marked reduction in plasma SOD concentrations [17]. Another study found that hypobaric hypoxia decreases GSH activity [18]. These findings collectively highlight a strong correlation between oxidative stress and the severity of AMS. In this study, TQ significantly elevated SOD and GSH levels and reduced MDA, demonstrating potent antioxidant activity that effectively mitigates ROS-induced injury and restores cellular redox balance.

Beyond direct cytotoxicity, ROS also play a crucial role in signaling under hypoxic conditions. ROS production can inhibit prolyl hydroxylase activity, leading to stabilization of HIF-1α and subsequent upregulation of downstream targets such as VEGFA and EPO—key regulators of angiogenesis and erythropoiesis [19]. HIF-1α is a central transcriptional regulator of cellular adaptation to hypoxia, promoting angiogenesis and erythropoiesis primarily through the upregulation of VEGFA and EPO [6]. Several bioactive components of TQ, such as borneol, camphor, and musk, have been previously reported to exhibit anti-hypoxic and neuroprotective activities. These actions were mediated through mechanisms including improved oxygen utilization, mitochondrial stabilization, anti-inflammatory responses, and regulation of hypoxia-responsive pathways such as HIF-1α signaling [20,21]. In our study, TQ treatment significantly downregulated HIF-1α, VEGFA, and EPO expression, consistent with the observed elevations in SaO_2_ and PO_2_, indicating effective restoration of systemic oxygen homeostasis.

Under hypoxic stress, mitochondrial dysfunction promotes the release of cytochrome c, increases the Bax/Bcl-2 ratio, and activates downstream Caspase-3, leading to apoptosis [22]. Our findings demonstrated that TQ suppressed pro-apoptotic markers and upregulated Bcl-2 expression, thereby protecting alveolar and neuronal cells from hypoxia-induced apoptosis. According to previous studies, borneol has been shown to alleviate myocardial ischemia–reperfusion injury by reducing the expression of Bax, Bcl-2, and Caspase-3 in cardiomyocytes [23] and to protect neuronal cells by modulating the p38 mitogen-activated protein kinase (MAPK) pathway [24]. Similarly, activation of the PI3K/Akt pathway has been implicated in promoting Bcl-2 expression and counteracting Bax-mediated apoptosis [25], while musk ketone has been reported to activate PI3K/Akt signaling in ischemic neural stem cells, contributing to neuroprotection [26]. These converging lines of evidence support the notion that TQ exerts multi-organ protection via coordinated modulation of apoptosis-related pathways under hypoxic conditions.

Although the HIF-1α pathway was the main focus of our mechanistic validation, the results of our network pharmacology and KEGG enrichment analyses suggest that TQ may also influence other key signaling cascades, including PI3K/Akt, MAPK, and NF-κB. These pathways play critical roles in cellular responses to oxidative stress and inflammation. In line with our findings, in vitro studies have shown that inhibition of p38 MAPK and NF-κB phosphorylation, along with reduced Bax/Bcl-2 and cleaved Caspase-3/Caspase-3 ratios, can mitigate hypoxia-induced oxidative stress and cell death [27].

In silico target prediction using SwissTargetPrediction further supports these results by indicating potential direct interactions between TQ components and targets such as *HIF1A*, *AKT1*, *BAX*, and *CASP3*, reinforcing the “component–target–pathway” paradigm that underpins traditional Chinese medicine pharmacology. Borneol, for example, enhances blood–brain barrier permeability [28]; frankincense and its derivatives have potent antioxidant and anti-inflammatory effects [29,30]; musk and styrax compounds are known to activate PI3K/Akt and MAPK signaling pathways, contributing to cardiovascular and neural protection [26,31].

**Figure 6:**
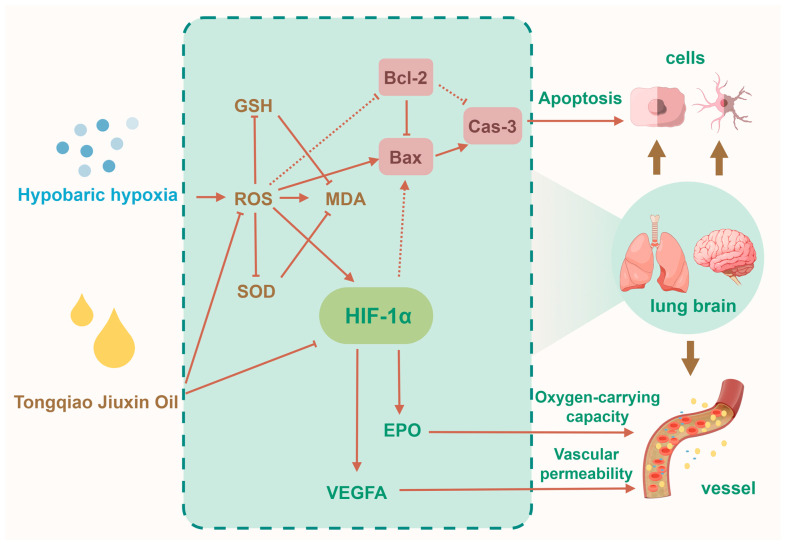
Schematic illustration of the proposed mechanism of Tongqiao Jiuxin Oil in alleviating acute mountain sickness. TQ confers protection to pulmonary and cerebral tissues through downregulating ROS, suppressing the HIF-1α/VEGFA/EPO axis, attenuating hypoxia-induced oxidative stress and cellular apoptosis, and reducing vascular permeability, thereby alleviating AMS.

This study provides the first systematic evaluation of Tongqiao Jiuxin Oil—a TCM formula comprising sandalwood, agarwood, frankincense, styrax, borneol, menthol, camphor, and musk—in mitigating pulmonary and cerebral injuries in a rat model of AMS induced by hypobaric hypoxia. The administration of TQ significantly improved arterial oxygenation, enhanced endogenous antioxidant defense mechanisms, and attenuated histopathological damage in both lung and brain tissues. Mechanistic investigations revealed that these protective effects may be associated with the downregulation of the HIF-1α/VEGFA/EPO axis and modulation of mitochondrial-mediated apoptosis, specifically through reductions in the Bax/Bcl-2 ratio and Caspase-3 activation (Figure 6).

Although the current study provided compelling evidence for the protective effects of TQ against AMS, several questions remain to be addressed. Firstly, more detailed pharmacokinetic studies are warranted to elucidate the brain and lung tissue distribution of individual bioactive components—particularly borneol and menthol—which are believed to facilitate blood–brain barrier penetration and central nervous system delivery. Secondly, the use of gene knockout or overexpression models (e.g., HIF-1α−/− transgenic rats) may help to dissect the precise signaling cascades modulated by TQ. Finally, clinical validation in high-altitude-exposed populations or patients at risk of AMS would be critical to establish the translational potential of TQ and promote the modernization of traditional Chinese medicine through evidence-based approaches.

## 4. Materials and Methods

### 4.1. Preparation of TQ

A 100 μL aliquot of TQ was transferred into a 1.5 mL centrifuge tube and mixed with 900 μL of methanol. The mixture was vortexed for 1 min and subjected to ultrasonic extraction in an ice-water bath for 1 h. After centrifugation at 12,000 rpm for 10 min at 4 °C, 200 μL of supernatant was collected and transferred to an LC-MS vial for analysis.

### 4.2. Chemical Profiling of TQ

Chemical constituents in TQ were investigated by using the UHPLC-Q-Exactive Orbitrap HRMS (Thermo Fisher Scientific, Waltham, MA, USA; Thermo-Orbitrap-QE HF). The mobile phase was composed of water/0.1% formic acid (A) and acetonitrile (B). The displacement step gradient conditions were set as follows: 0–4 min, 5.0–30% B; 4–8 min, 30–50% B; 8–10 min, 50–80% B; 10–14 min, 80–100% B; 14–15 min, 100% B; 15–15.1 min, 100–5.0% B; and 15.1–16 min, 5.0% B. The chromatographic conditions, including the mobile phase gradient program, were identical for both positive and negative ion modes. Full-scan data within the range of *m/z* (mass-to-charge ratio) 100–1500 were acquired. Based on accurate mass measurements, MS/MS fragmentation patterns, and isotopic distribution profiles, compound identification was performed using TCM databases (https://www.tcmsp-e.com/, accessed on 9 January 2025). After merging and de-duplicating compounds detected in both positive and negative ionization modes, the relative peak areas of all identified metabolites were normalized to a total of 100% to construct a qualitative–quantitative data matrix. This matrix encompassed all analyzable information extracted from the raw data and served as the basis for all subsequent analyses.

### 4.3. Network Pharmacology Analysis

After compound identification in TQ, the standardized SMILES for each constituent was retrieved from the PubChem database (http://bioinfo.org/kobas/, accessed on 23 May 2025). Target prediction for these compounds was then performed using the SwissTargetPrediction database (http://swisstargetprediction.ch/, accessed on 24 May 2025) based on the input of their respective standard SMILES structures.

To identify potential disease-related targets for AMS, the keyword “acute mountain sickness” was queried in both the GeneCards (https://www.genecards.org/, accessed on 25 May 2025) and OMIM (https://www.ncbi.nlm.nih.gov/omim/, accessed on 25 May 2025) databases. Drug-related and disease-related targets were collected and de-duplicated separately. The overlapping targets were visualized using Venny 2.1 (https://bioinfogp.cnb.csic.es/tools/venny/, accessed on 25 May 2025), generating a Venn diagram to illustrate the intersection between TQ-predicted targets and AMS-associated targets. The intersected targets were subsequently extracted for further analysis [32].

A compound–target–disease interaction network was constructed using Cytoscape software (version 3.10.0). PPI data for the intersected targets were obtained from the STRING database (https://string-db.org/, accessed on 25 May 2025), with the confidence score threshold set to 0.7 (high confidence). The PPI network was then visualized in Cytoscape, and the top 10 hub targets were identified using the cytoHubba plugin, ranked by MCC scores.

To elucidate the biological pathways involved, KEGG pathway enrichment analysis was performed using the DAVID database (https://david.ncifcrf.gov/, accessed on 25 May 2025), with the organism restricted to Homo sapiens [33]. The top 15 significantly enriched pathways were selected based on *p*-value rankings and visualized for annotation.

### 4.4. Animals

A total of 48 male SPF Wistar rats (6–8 weeks old, body weight 200 ± 20 g) were purchased from Vital River Laboratory Animal Technology Co., Ltd., Zhejiang, China (License No. SCXK [Zhe] 2020-0002). Animals were housed under standard laboratory conditions with a 12 h light/dark cycle, temperature maintained at 20–26 °C, and relative humidity at 30–70%. Rats had free access to standard chow and tap water. All animals underwent a 3-day acclimatization period prior to the start of the experiment. All experimental procedures were conducted in accordance with institutional guidelines and were approved by the Medical and Experimental Animal Ethics Committee of Beijing University of Chinese Medicine (Approval No. BUCM-2024030901-1014, approved on 9 March 2024).

### 4.5. Establishment of AMS Rat Model and Drug Administration

All rats were randomly assigned into six groups (*n* = 8) based on body weight: the normal group (Normal), the model group (Model), the Hongjingtian Oral Liquid (230508, Tibet Tibetan Medicine Group Co., Ltd., Nyingchi, China) group (HJT, 1.8 mL/kg), the high-dose Tongqiao Jiuxin Oil group (TQ-H, 48.20 mg/kg), the medium-dose group (TQ-M, 24.10 mg/kg, equivalent dose), and the low-dose group (TQ-L, 12.05 mg/kg). TQ was provided by Guangzhou Baiyunshan Xingqun (Pharmaceutical) Co., Ltd., Guangzhou, China (batch number: UD40003). All TQ preparations were diluted with tea oil and administered orally by gavage at a volume of 5 mL/kg. Rats in the Normal and Model groups received an equal volume of tea oil daily by gavage. Rats in the HJT group received HJT at 1.8 mL/kg, also in a gavage volume of 5 mL/kg. Except for the Normal group, all other groups underwent a 10-day pre-treatment period under normoxic conditions, followed by continuous drug administration for an additional 3 days in a hypobaric hypoxia chamber (simulating ~10% oxygen, approximately 5500 m altitude) to induce AMS-like symptoms.

### 4.6. Tissue Sample Collection and Organ Coefficient Calculation

Following three days of exposure to hypobaric hypoxia, all rats were anesthetized, and blood was collected from the abdominal aorta. Blood samples were collected into three types of tubes: one containing EDTA, one with heparin sodium, and one plain tube. The anticoagulation tubes were gently inverted several times to ensure thorough mixing of the blood with the anticoagulant and then set aside for further use. The plain tubes were left undisturbed at room temperature for 4 h; then, blood was centrifuged at 3000 rpm for 10 min and the serum was collected and stored at −80 °C until analysis.

The heart, liver, brain, and lungs were rapidly excised and isolated on ice and weighed. Organ coefficients were calculated using the following formula:

(1) $$Organ\;coefficient(\%)=\left(\frac{Organ\;weight(g)}{Body\;weight(g)}\right)\times 100$$

Tissue blocks from the same anatomical locations of the heart, liver, brain, and lungs were fixed in 4% paraformaldehyde for histopathological examination. Remaining tissues were placed in cryogenic tubes and stored at −80 °C for further biochemical analysis.

### 4.7. Blood Gas Analysis

Blood gas parameters were measured using a portable blood gas analyzer (CS-T180, DIRUI Industrial Co., Ltd., Changchun, China). Whole-blood samples collected in heparinized tubes were analyzed immediately. The following indices were recorded: PO_2_, SaO_2_, BEecf, HCO_3_^−^, PCO_2_, TCO_2_, and pH. All measurements were performed according to the instructions of the manufacturer.

### 4.8. Peripheral Blood Cell Analysis

Peripheral blood samples in EDTA anticoagulant tubes were analyzed using an automated five-classification animal blood cell analyzer (URIT-5160Vet, URIT Medical Electronic Co., Ltd., Guilin, China). The following hematological parameters were recorded: HGB, HCT, and RBC. All procedures were carried out in accordance with the manufacturer’s instruction.

### 4.9. Biochemical Analysis

Serum levels of HIF-1α, MDA, and GSH were measured using enzyme-linked immunosorbent assay (ELISA) kits followed the manufacturer’s protocols. For the quantification of SOD, serum samples were loaded into biochemical assay cups and analyzed using a fully automated biochemical analyzer (DIRUI CS-T180, DIRUI Industrial Co., Ltd., Changchun, China).

### 4.10. Histological Analysis of Heart, Liver, Lung and Brain Tissues

Tissue samples of the hearts, livers, lungs, and brains were fixed in 4% paraformaldehyde for 48 h. Subsequently, the specimens were dehydrated through a graded ethanol series and rendered transparent using xylene. After these preparatory steps, tissues were embedded in paraffin blocks following standard protocols. Sections were then cut, stained with hematoxylin and eosin (H&E), and examined under a light microscope for histopathological evaluation.

### 4.11. Western Blot Analysis

Western blotting was employed to detect the expression of HIF-1α, VEGFA, EPO, Caspase-3, Bax, and Bcl-2 proteins in lung and brain tissues. Tissue was lysed in RIPA lysis buffer containing protease and phosphatase inhibitors. Protein concentration was measured using the BCA protein assay kit. Total protein (10 μg) from each sample was loaded into SDS-PAGE gels for protein separation and then transferred to PVDF membranes. The PVDF membranes containing proteins were incubated in blocking solution for 2 h. Subsequently, the PVDF membranes were incubated with the respective primary antibodies overnight at 4 °C, including HIF-1α antibody (ab179483, Abcam, Cambridge, UK), VEGFA antibody (ab214424, Abcam, Cambridge, UK), EPO antibody (YT7988, Immunoway, Plano, TX, USA), Caspase-3 antibody (ab184787, Abcam, Cambridge, UK), Bax antibody (ab32503, Abcam, Cambridge, UK), Bcl-2 antibody (YM3041, Immunoway, Plano, TX, USA), and glyceraldehyde-3-phosphate dehydrogenase (GAPDH) antibody (YM3029, Immunoway, Plano, TX, USA). Afterward, the membranes were incubated with HRP-conjugated Affinipure Goat Anti-Rabbit IgG (H+L) (ab205718, Abcam, Cambridge, UK) or HRP-conjugated Affinipure Goat anti-Mouse IgG (H+L) (SA00001-1, Proteintech, Wuhan, China) for 2 h. The PVDF membranes were then washed with TBST solution, treated with chemiluminescent reagents, and exposed and photographed. Protein bands on the Western blot were quantified using ImageJ-win64, and relative expression levels were analyzed based on the ratio of the corresponding bands to GAPDH.

### 4.12. Statistical Analysis

All data were analyzed using SPSS 20.0 software. Quantitative results are presented as mean ± SEM. One-way ANOVA was used for comparisons among groups when the data were normally distributed and exhibited homogeneity of variance. Post hoc comparisons between groups were performed using the LSD test. In cases where variances were unequal, the Games–Howell test was applied. The normal distribution of experimental data was checked with the Shapiro–Wilk test. For data not following a normal distribution, the Kruskal–Wallis test was used. A *p*-value of less than 0.05 was considered statistically significant.

## 5. Conclusions

This study provides the first comprehensive evidence that TQ, a traditional multi-component Chinese medicine, exerts significant protective effects against AMS-induced pulmonary and cerebral injury under hypobaric hypoxia. Through improving oxygenation, enhancing antioxidant defenses, and modulating the HIF-1α/VEGFA/EPO axis and apoptosis pathways, TQ demonstrated multi-targeted efficacy. These findings not only deepen the mechanistic understanding of TQ in high-altitude adaptation but also highlight its potential as a novel therapeutic strategy for AMS, offering scientific support for its clinical application and modern development. Future work should focus on optimizing TQ’s formulation, identifying key active components, enhancing bioavailability, developing advanced delivery systems for targeted action, and conducting systematic experimental validation in clinical settings.

## Data Availability

Data presented in this study is contained within the article and Supplementary Material. Further inquiries can be directed to the corresponding author.

## Supplementary Materials

The following supporting information can be downloaded at https://www.mdpi.com/article/10.3390/ph18081153/s1, Table S1: Detailed chemical identification data of TQ.

## Abbreviations

The following abbreviations are used in this manuscript:

Akt	Protein kinase B
AMS	Acute mountain sickness
Bax	Bcl-2-Associated X
Bcl-2	B-cell lymphoma-2
BEecf	Base excess in the extracellular fluid compartment
ELISA	Enzyme-linked immunosorbent assay
EPO	Erythropoietin
GAPDH	Glyceraldehyde-3-phosphate dehydrogenase
GSH	Glutathione
HACE	High-altitude cerebral edema
HAPE	High-altitude pulmonary edema
H&E	Hematoxylin and eosin
HCO_3_^−^	Bicarbonate ion concentration
HCT	Hematocrit
HGB	Hemoglobin
HIF-1α	Hypoxia-inducible factor-1α
HJT	Hongjingtian
KEGG	Kyoto Encyclopedia of Genes and Genomes
MAPK	Mitogen-activated protein kinase
MCC	Maximal Clique Centrality
MDA	Malondialdehyde
NF-κB	Nuclear factor kappa-B
PCO_2_	Partial pressure of carbon dioxide
PI3K	Phosphatidylinositide 3-kinases
PO_2_	Partial pressure of oxygen
RBC	Red blood cell count
ROS	Reactive oxygen species
SOD	Superoxide dismutase
TCM	Traditional Chinese medicine
TCO_2_	Total carbon dioxide
TIC	Total ion chromatogram
TQ	Tongqiao Jiuxin Oil
VEGFA	Vascular endothelial growth factor A

## References

[1] Imray C., Wright A., Subudhi A., Roach R. (2010). Acute Mountain Sickness: Pathophysiology, Prevention, and Treatment. Prog. Cardiovasc. Dis..

[2] Basnyat B., Murdoch D.R. (2003). High-Altitude Illness. Lancet.

[3] Hackett P.H., Roach R.C. (2001). High-Altitude Illness. N. Engl. J. Med..

[4] West J.B. (2015). High-Altitude Medicine. Lancet Respir. Med..

[5] Bärtsch P., Swenson E.R. (2013). Clinical Practice: Acute High-Altitude Illnesses. N. Engl. J. Med..

[6] Semenza G.L. (2012). Hypoxia-Inducible Factors in Physiology and Medicine. Cell.

[7] Haase V.H. (2013). Regulation of Erythropoiesis by Hypoxia-Inducible Factors. Blood Rev..

[8] Yu A.Y., Shimoda L.A., Iyer N.V., Huso D.L., Sun X., McWilliams R., Beaty T., Sham J.S.K., Wiener C.M., Sylvester J.T. (1999). Impaired Physiological Responses to Chronic Hypoxia in Mice Partially Deficient for Hypoxia-Inducible Factor 1α. J. Clin. Investig..

[9] Chan D.A., Giaccia A.J. (2007). Hypoxia, Gene Expression, and Metastasis. Cancer Metastasis Rev..

[10] Zhuang Y.Y., Lu Y.Y., Li R., Jin Z., Chu Q.M., Liang J.H. (2019). Effects of sublingual administration of Tongqiao Jiuxin Oil on the quality of life in unstable angina pectoris patients with qi stagnation and blood stasis syndrome. Chin. Gen. Pract..

[11] Liu R., Zhang L., Lan X., Li L., Zhang T.-T., Sun J.-H., Du G.-H. (2011). Protection by Borneol on Cortical Neurons against Oxygen-Glucose Deprivation/Reperfusion: Involvement of Anti-Oxidation and Anti-Inflammation through Nuclear Transcription Factor κappaB Signaling Pathway. Neuroscience.

[12] Pu X., Li F., Lin X., Wang R., Chen Z. (2022). Oxidative Stress and Expression of Inflammatory Factors in Lung Tissue of Acute Mountain Sickness Rats. Mol. Med. Rep..

[13] Coimbra-Costa D., Alva N., Duran M., Carbonell T., Rama R. (2017). Oxidative Stress and Apoptosis after Acute Respiratory Hypoxia and Reoxygenation in Rat Brain. Redox Biol..

[14] Yan X., Liu J., Zhu M., Liu L., Chen Y., Zhang Y., Feng M., Jia Z., Xiao H. (2021). Salidroside Orchestrates Metabolic Reprogramming by Regulating the HIF-1α Signalling Pathway in Acute Mountain Sickness. Pharm. Biol..

[15] Pena E., El Alam S., Siques P., Brito J. (2022). Oxidative Stress and Diseases Associated with High-Altitude Exposure. Antioxidants.

[16] Sarada S., Himadri P., Mishra C., Geetali P., Ram M.S., Ilavazhagan G. (2008). Role of Oxidative Stress and NFkB in Hypoxia-Induced Pulmonary Edema. Exp. Biol. Med..

[17] Tang X.-G., Wen J., Zhang X.-S., Jiang D.-C. (2018). Association between Decreased Osteopontin and Acute Mountain Sickness upon Rapid Ascent to 3500 m among Young Chinese Men. J. Travel. Med..

[18] Irarrázaval S., Allard C., Campodónico J., Pérez D., Strobel P., Vásquez L., Urquiaga I., Echeverría G., Leighton F. (2017). Oxidative Stress in Acute Hypobaric Hypoxia. High. Alt. Med. Biol..

[19] Calvani M., Comito G., Giannoni E., Chiarugi P. (2012). Time-Dependent Stabilization of Hypoxia Inducible Factor-1α by Different Intracellular Sources of Reactive Oxygen Species. PLoS ONE.

[20] Li Y., Ren M., Wang J., Ma R., Chen H., Xie Q., Li H., Li J., Wang J. (2021). Progress in Borneol Intervention for Ischemic Stroke: A Systematic Review. Front. Pharmacol..

[21] Liu F., Cao L., Hu S., Ye H., Wu Q., Wu L. (2023). Muscone Promotes Functional Recovery by Facilitating Microglia Polarization into M2 Phenotype through PPAR-γ Pathway after Ischemic Stroke. Cell. Immunol..

[22] Elmore S. (2007). Apoptosis: A Review of Programmed Cell Death. Toxicol. Pathol..

[23] Zhang H., Dong J., Zhang J., Chen H., Liu T., Gan R., Wen J., Li Y. (2025). Effects of Borneol on Apoptosis of Hypoxia/Reoxygenation H9c2 Cells and Myocardial Ischemia-Reperfusion Injury Rats. Acta Cir. Bras..

[24] Xie Q., Lu D., Yuan J., Ren M., Li Y., Wang J., Ma R., Wang J. (2023). L-Borneol Promotes Neurovascular Unit Protection in the Subacute Phase of Transient Middle Cerebral Artery Occlusion Rats: P38-MAPK Pathway Activation, Anti-Inflammatory, and Anti-Apoptotic Effect. Phytother. Res..

[25] Manning B.D., Cantley L.C. (2007). AKT/PKB Signaling: Navigating Downstream. Cell.

[26] Zhou Z., Dun L., Wei B., Gan Y., Liao Z., Lin X., Lu J., Liu G., Xu H., Lu C. (2020). Musk Ketone Induces Neural Stem Cell Proliferation and Differentiation in Cerebral Ischemia via Activation of the PI3K/Akt Signaling Pathway. Neuroscience.

[27] Wang Z., Zhao G., Zibrila A.I., Li Y., Liu J., Feng W. (2021). Acetylcholine Ameliorated Hypoxia-Induced Oxidative Stress and Apoptosis in Trophoblast Cells via P38 MAPK/NF-κB Pathway. Mol. Hum. Reprod..

[28] Zheng Q., Chen Z.X., Xu M.B., Zhou X.L., Huang Y.Y., Zheng G.Q., Wang Y. (2018). Borneol, a Messenger Agent, Improves Central Nervous System Drug Delivery through Enhancing Blood-Brain Barrier Permeability: A Preclinical Systematic Review and Meta-Analysis. Drug Deliv..

[29] Nischang V., Witt F.M., Börner F., Gomez M., Jordan P.M., Werz O. (2024). Frankincense Preparation Promotes Formation of Inflammation-Resolving Lipid Mediators by Manipulating Lipoxygenases in Human Innate Immune Cells. Front. Pharmacol..

[30] Loeser K., Seemann S., König S., Lenhardt I., Abdel-Tawab M., Koeberle A., Werz O., Lupp A. (2018). Protective Effect of Casperome^®^, an Orally Bioavailable Frankincense Extract, on Lipopolysaccharide- Induced Systemic Inflammation in Mice. Front. Pharmacol..

[31] Xu Z., Lu D., Yuan J., Ren M., Ma R., Xie Q., Li Y., Li J., Wang J. (2021). Storax, A Promising Botanical Medicine for Treating Cardio-Cerebrovascular Diseases: A Review. Front. Pharmacol..

[32] Shi L., An Y., Cheng L., Li Y., Li H., Wang C., Lv Y., Duan Y., Dai H., He C. (2022). Qingwei San Treats Oral Ulcer Subjected to Stomach Heat Syndrome in Db/Db Mice by Targeting TLR4/MyD88/NF-κB Pathway. Chin. Med..

[33] Shan Z., Zhang H., He C., An Y., Huang Y., Fu W., Wang M., Du Y., Xie J., Yang Y. (2024). High-Protein Mulberry Leaves Improve Glucose and Lipid Metabolism via Activation of the PI3K/Akt/PPARα/CPT-1 Pathway. Int. J. Mol. Sci..

