# Supplementary figures and images for: Pharmacological Investigation of Tongqiao Jiuxin Oil Against High-Altitude Hypoxia: Integrating Chemical Profiling, Network Pharmacology, and Experimental Validation (part 2 of 2)
Source: Pharmaceuticals (Basel). 2025 Aug 2;18(8):1153. doi: 10.3390/ph18081153 (PMC12389502; doi:10.3390/ph18081153)

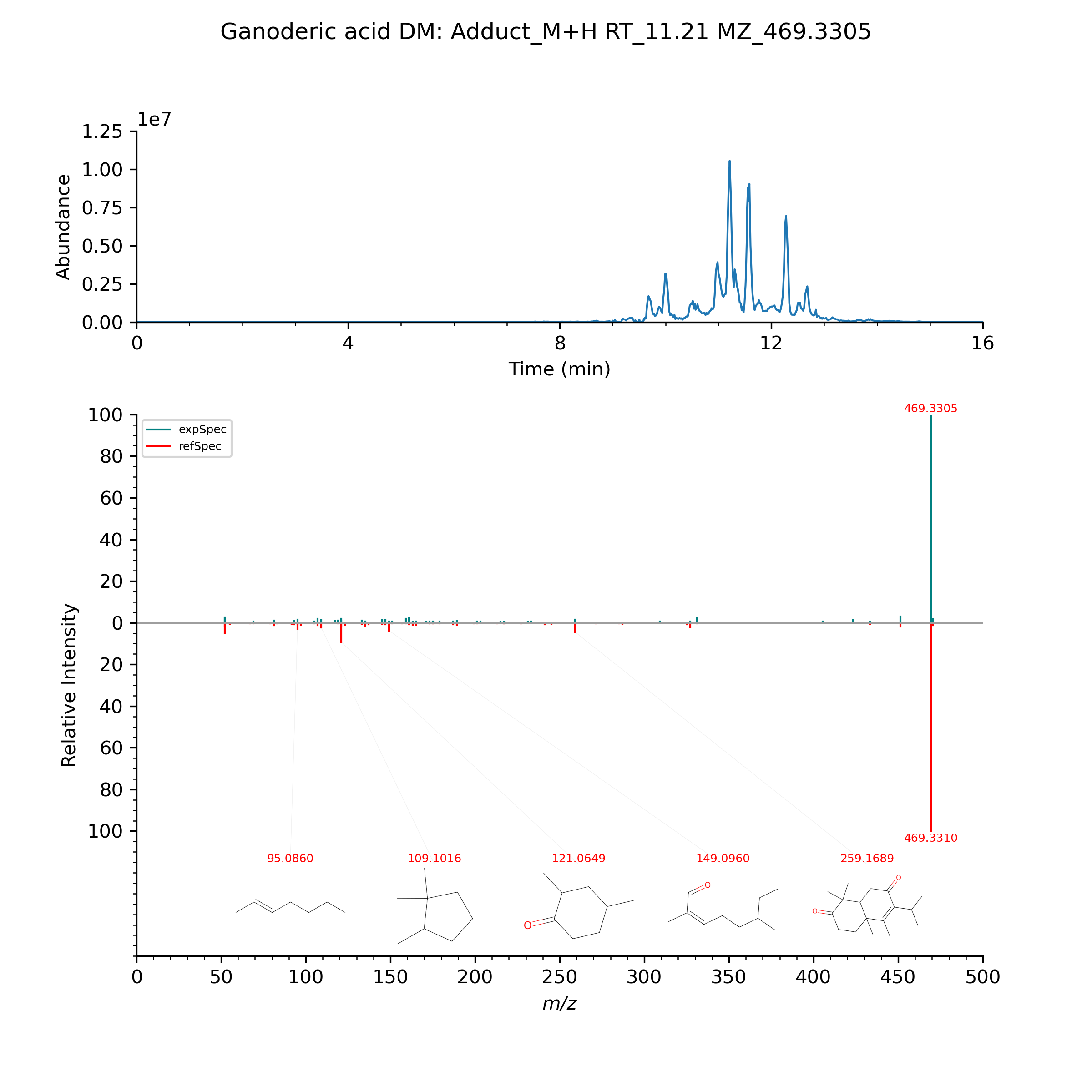

Supplement: Supplementary file 1 [file pharmaceuticals-18-01153-s001.zip › compound structures/M0101.png]

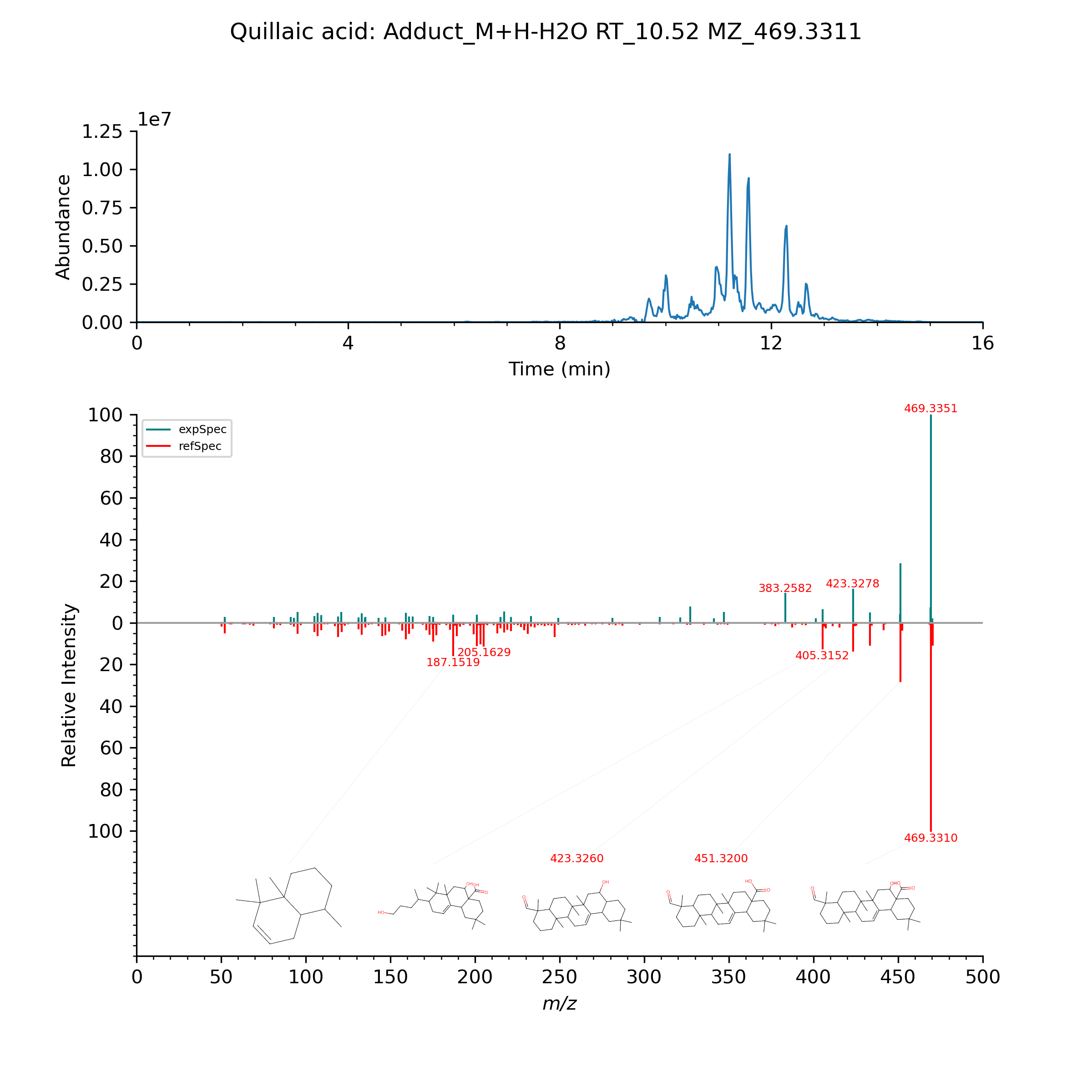

Supplement: Supplementary file 1 [file pharmaceuticals-18-01153-s001.zip › compound structures/M0102.png]

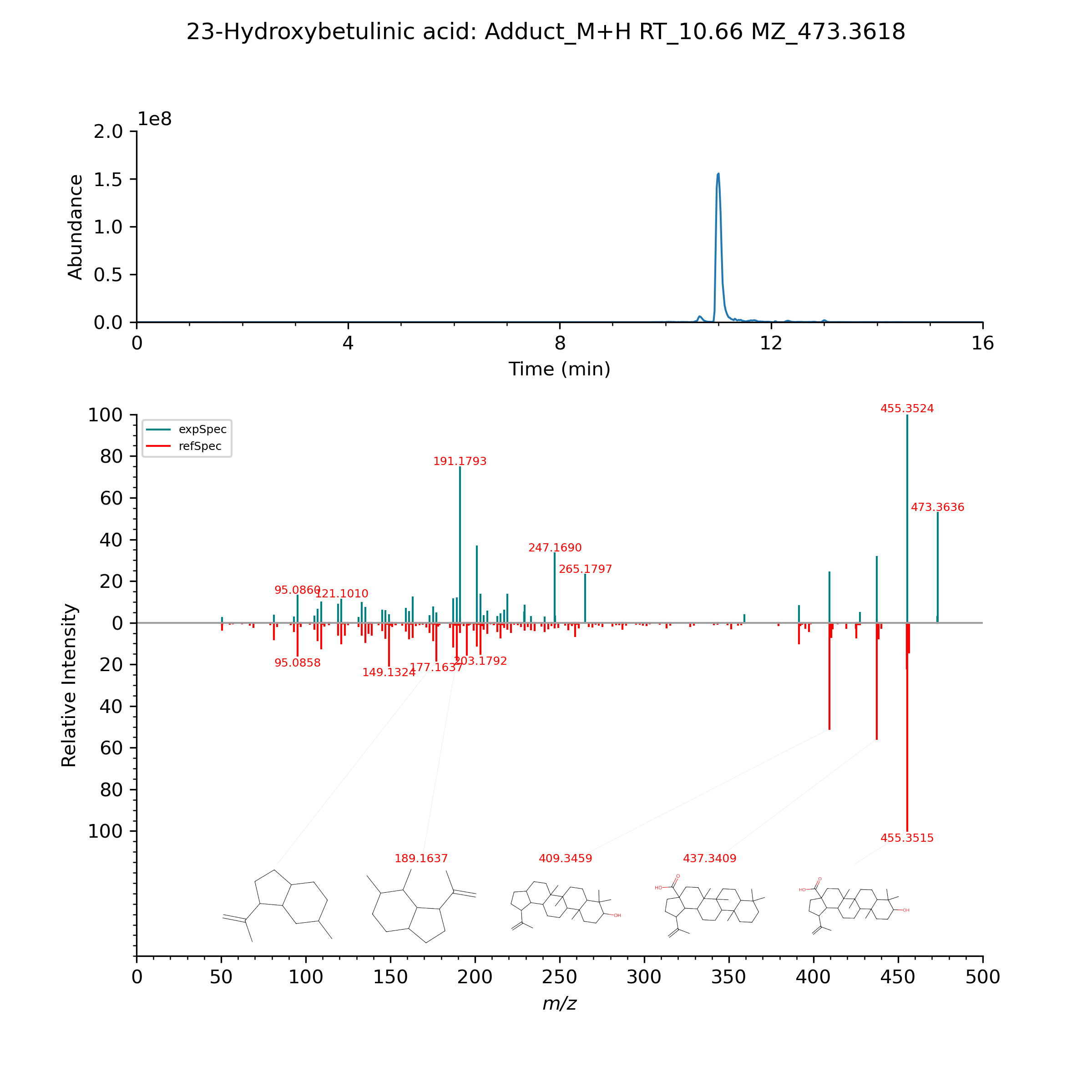

Supplement: Supplementary file 1 [file pharmaceuticals-18-01153-s001.zip › compound structures/M0103.png]

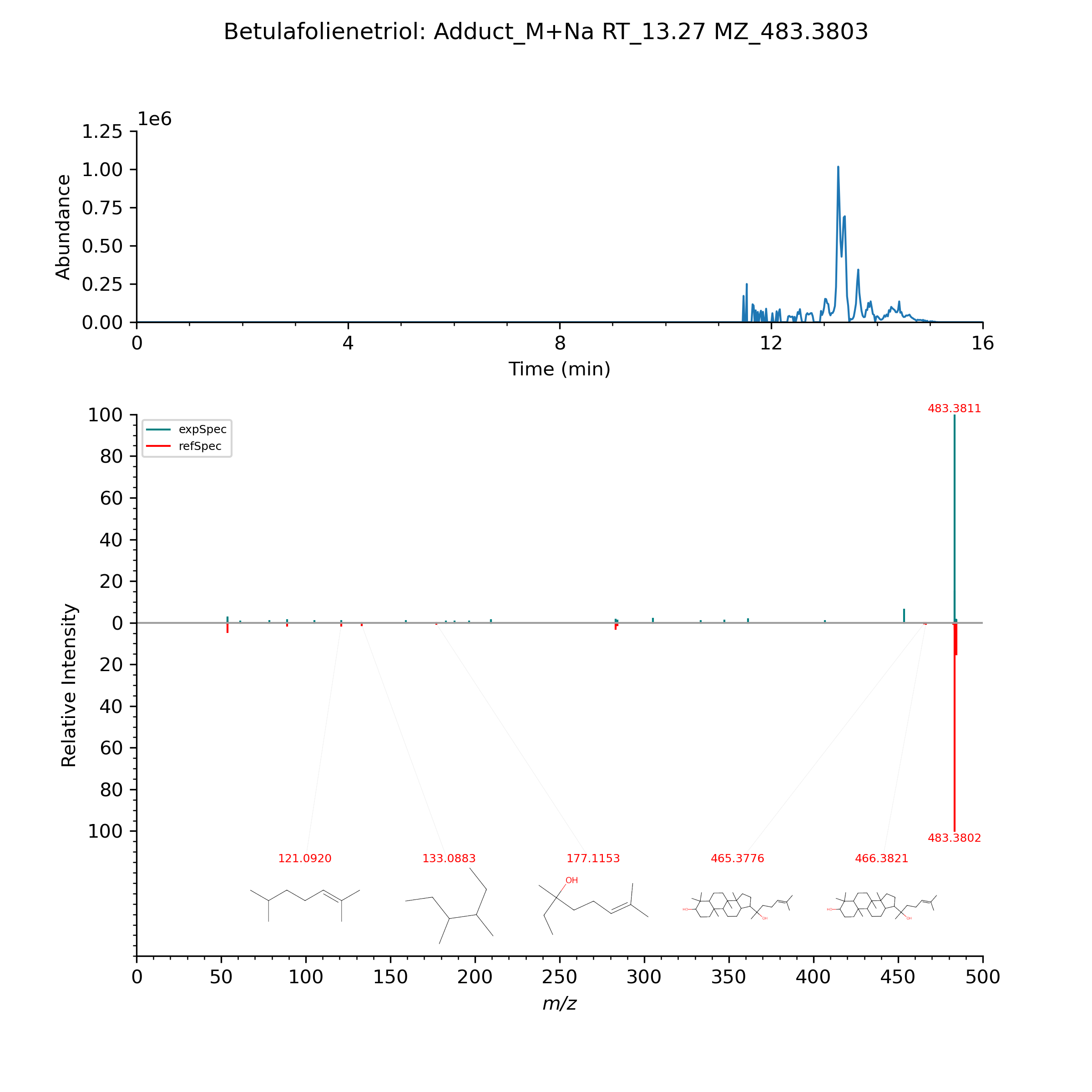

Supplement: Supplementary file 1 [file pharmaceuticals-18-01153-s001.zip › compound structures/M0104.png]

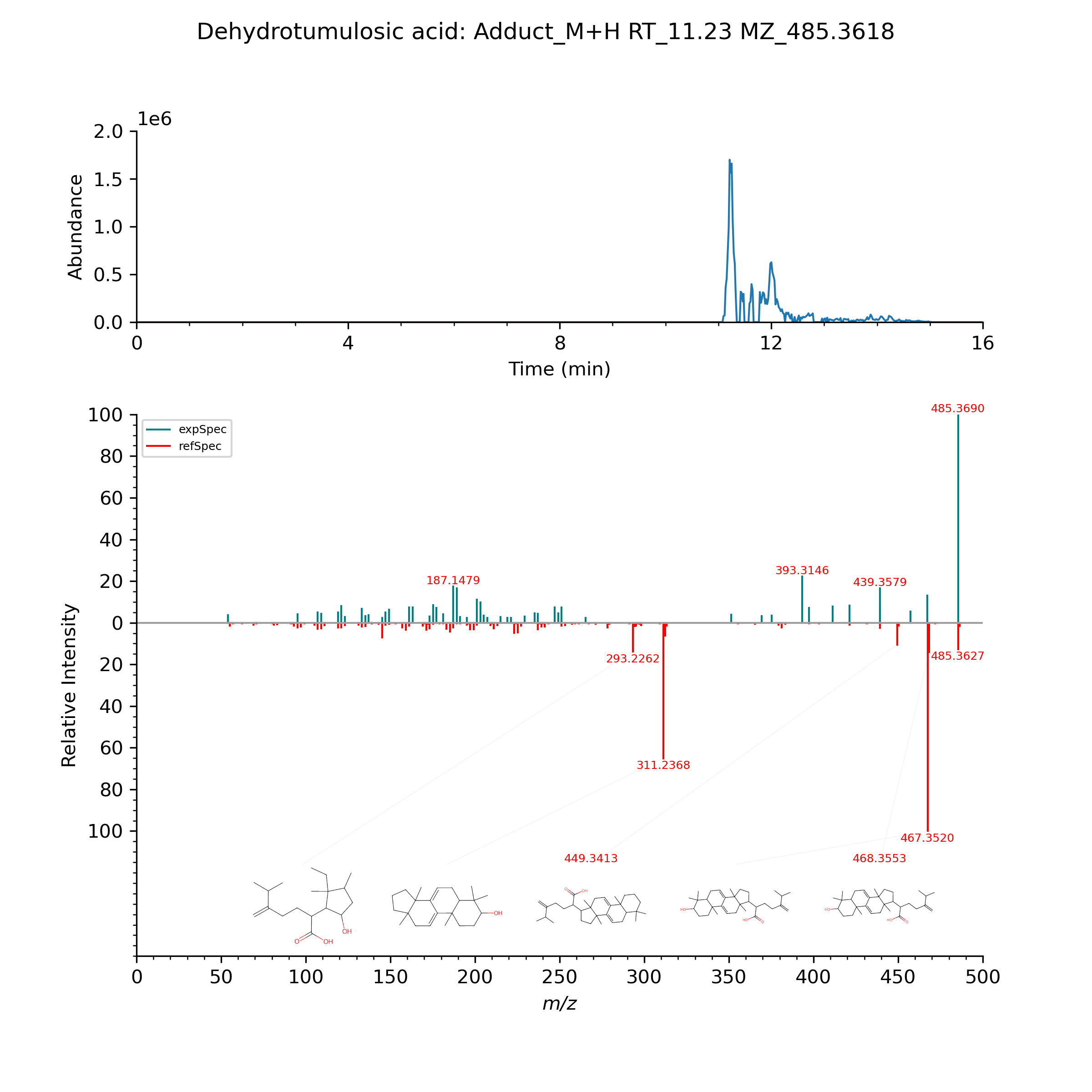

Supplement: Supplementary file 1 [file pharmaceuticals-18-01153-s001.zip › compound structures/M0105.png]

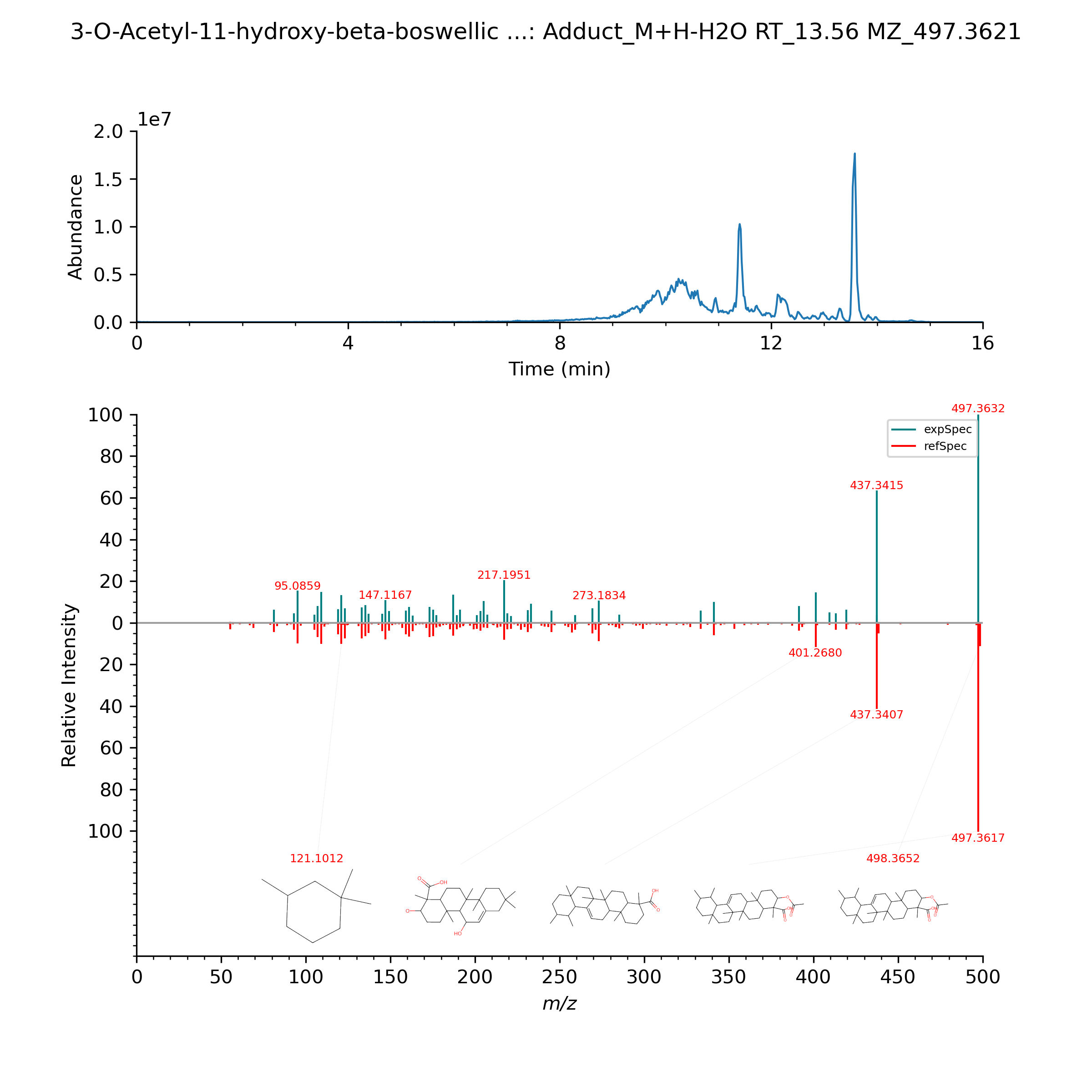

Supplement: Supplementary file 1 [file pharmaceuticals-18-01153-s001.zip › compound structures/M0106.png]

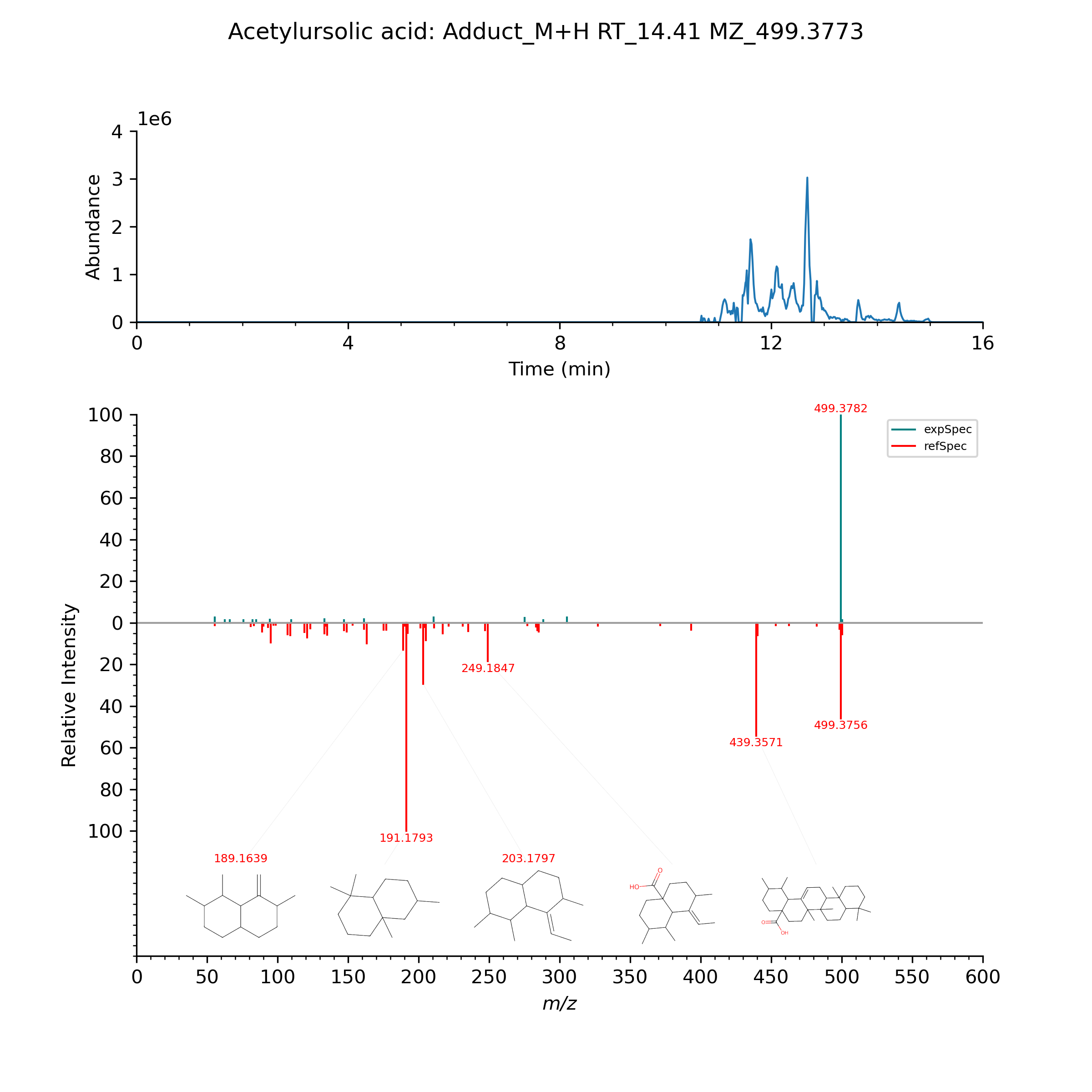

Supplement: Supplementary file 1 [file pharmaceuticals-18-01153-s001.zip › compound structures/M0107.png]

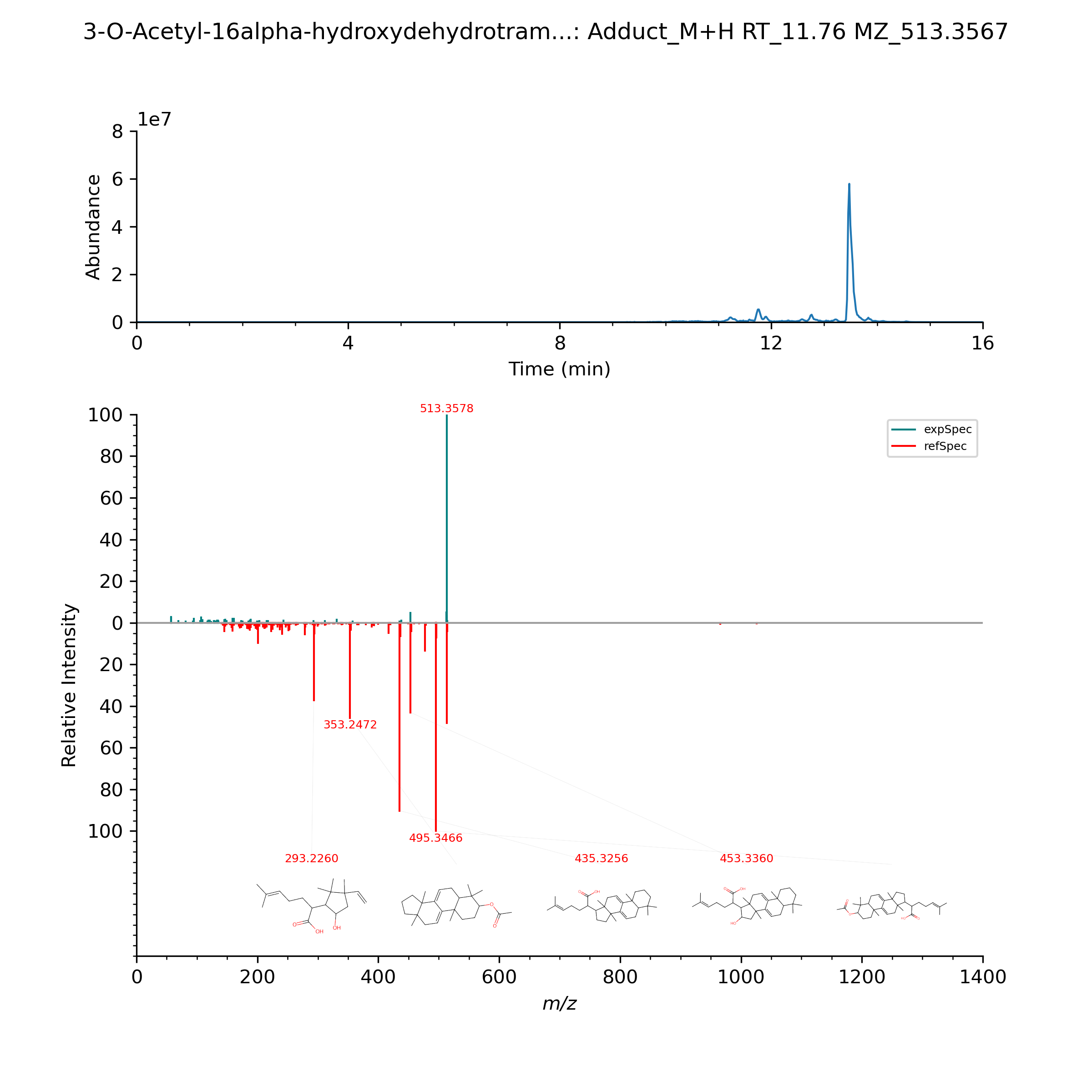

Supplement: Supplementary file 1 [file pharmaceuticals-18-01153-s001.zip › compound structures/M0108.png]

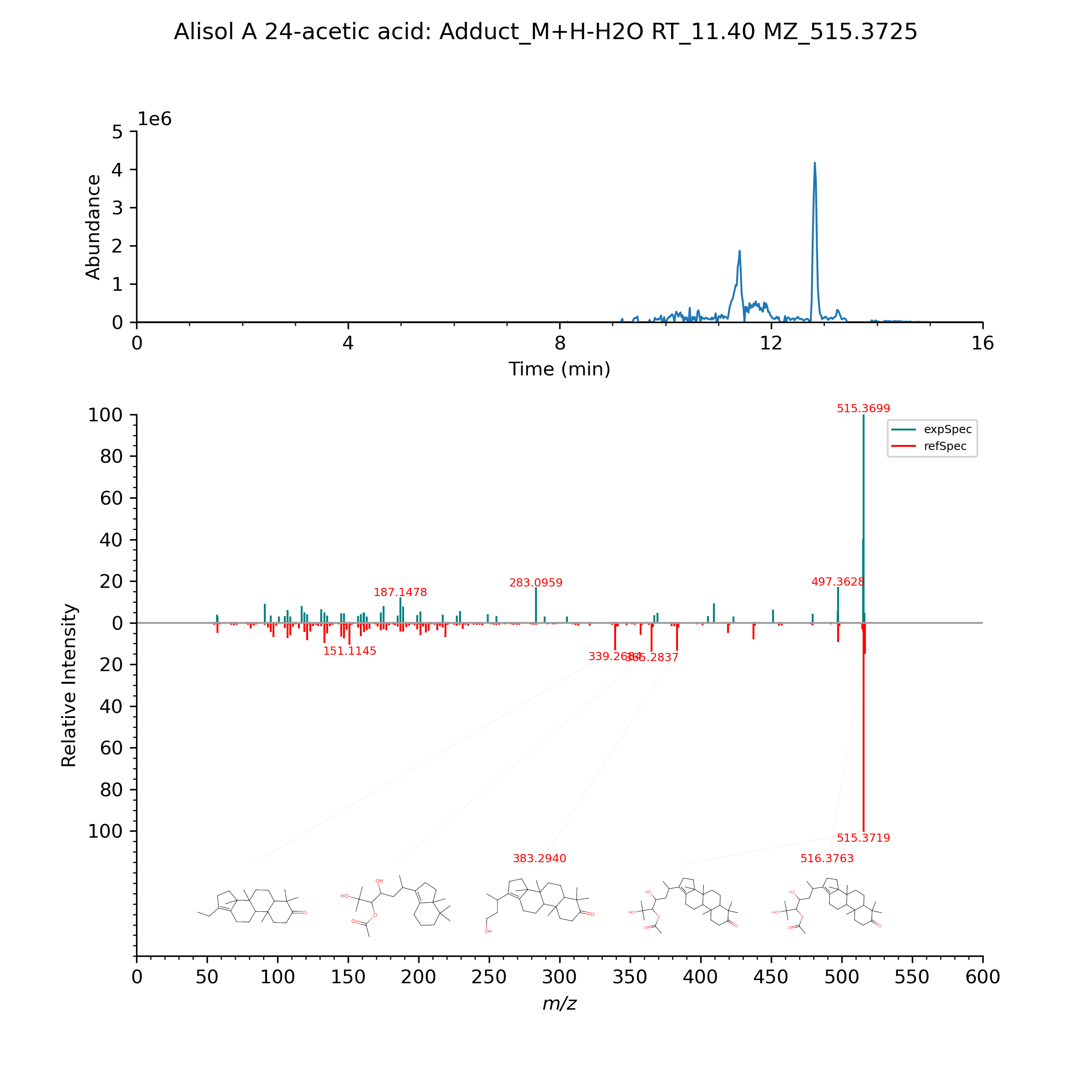

Supplement: Supplementary file 1 [file pharmaceuticals-18-01153-s001.zip › compound structures/M0109.png]

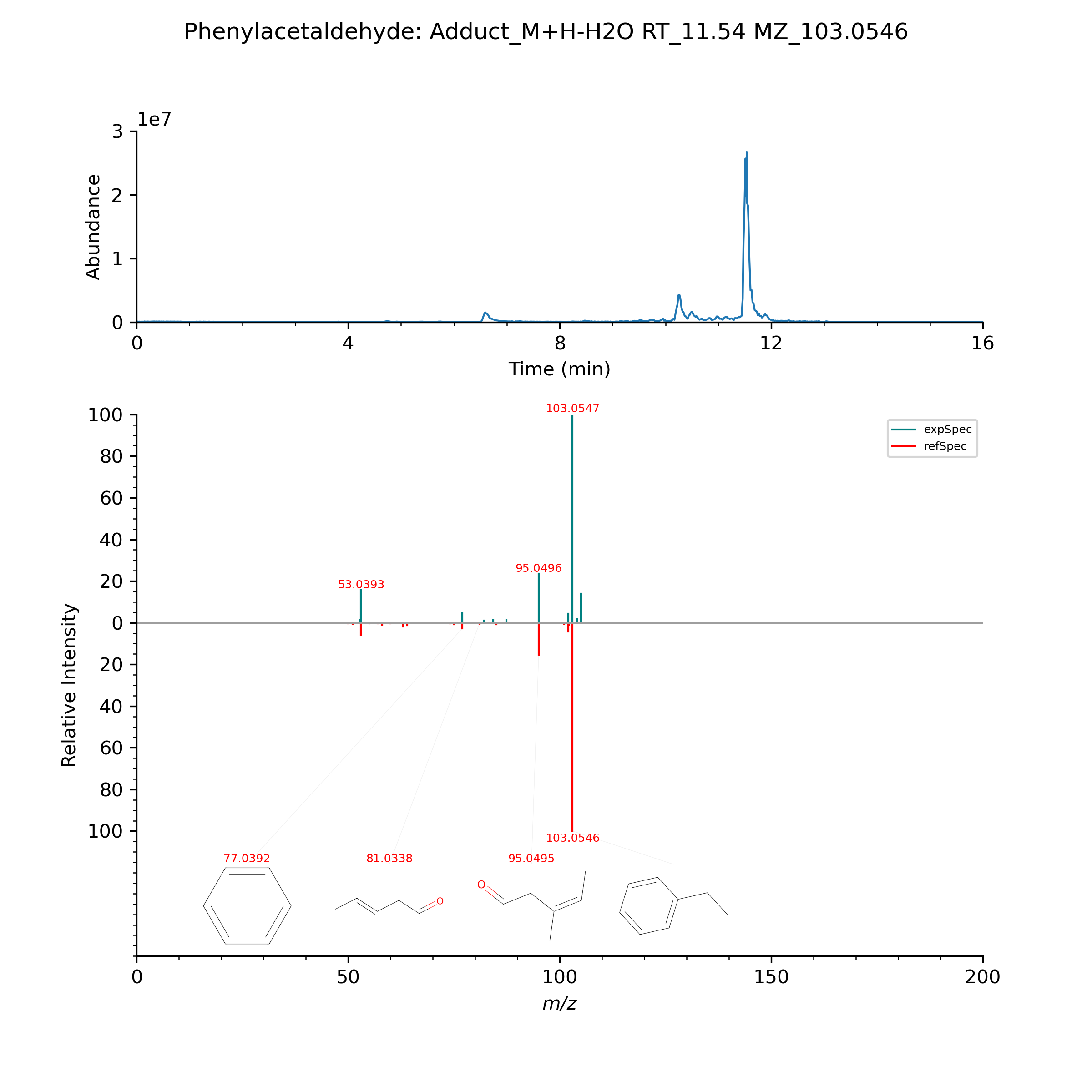

Supplement: Supplementary file 1 [file pharmaceuticals-18-01153-s001.zip › compound structures/M0110.png]

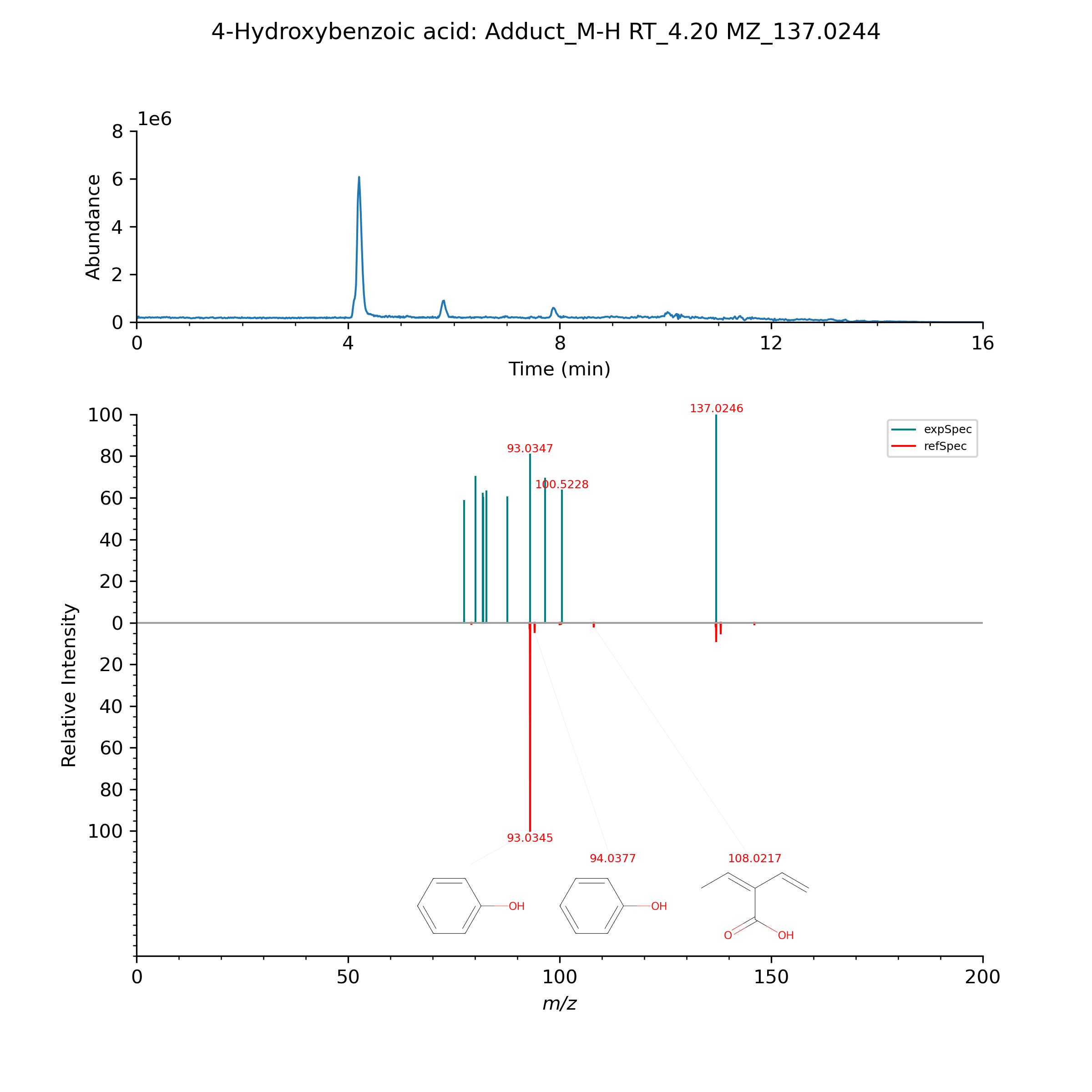

Supplement: Supplementary file 1 [file pharmaceuticals-18-01153-s001.zip › compound structures/M0111.png]

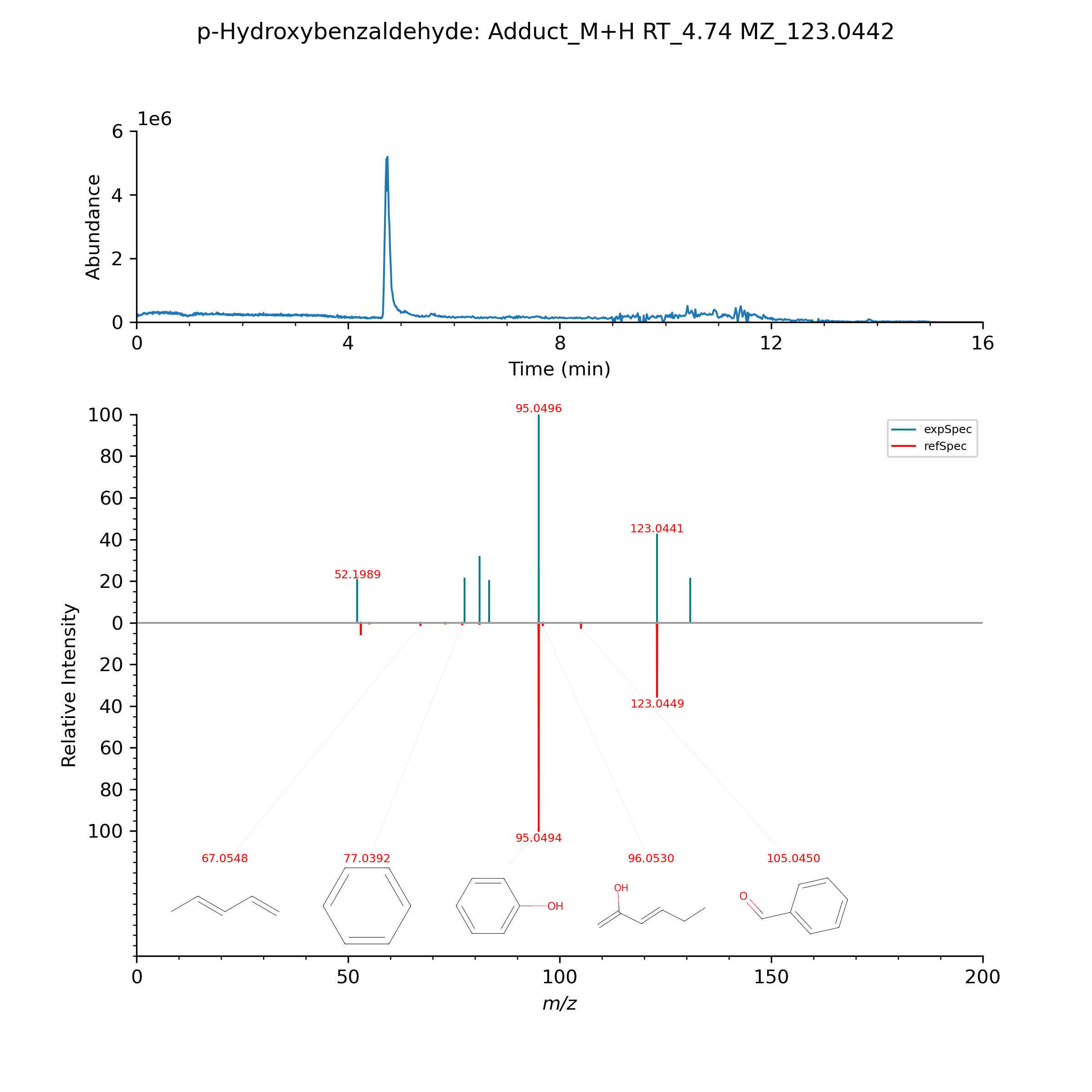

Supplement: Supplementary file 1 [file pharmaceuticals-18-01153-s001.zip › compound structures/M0112.png]

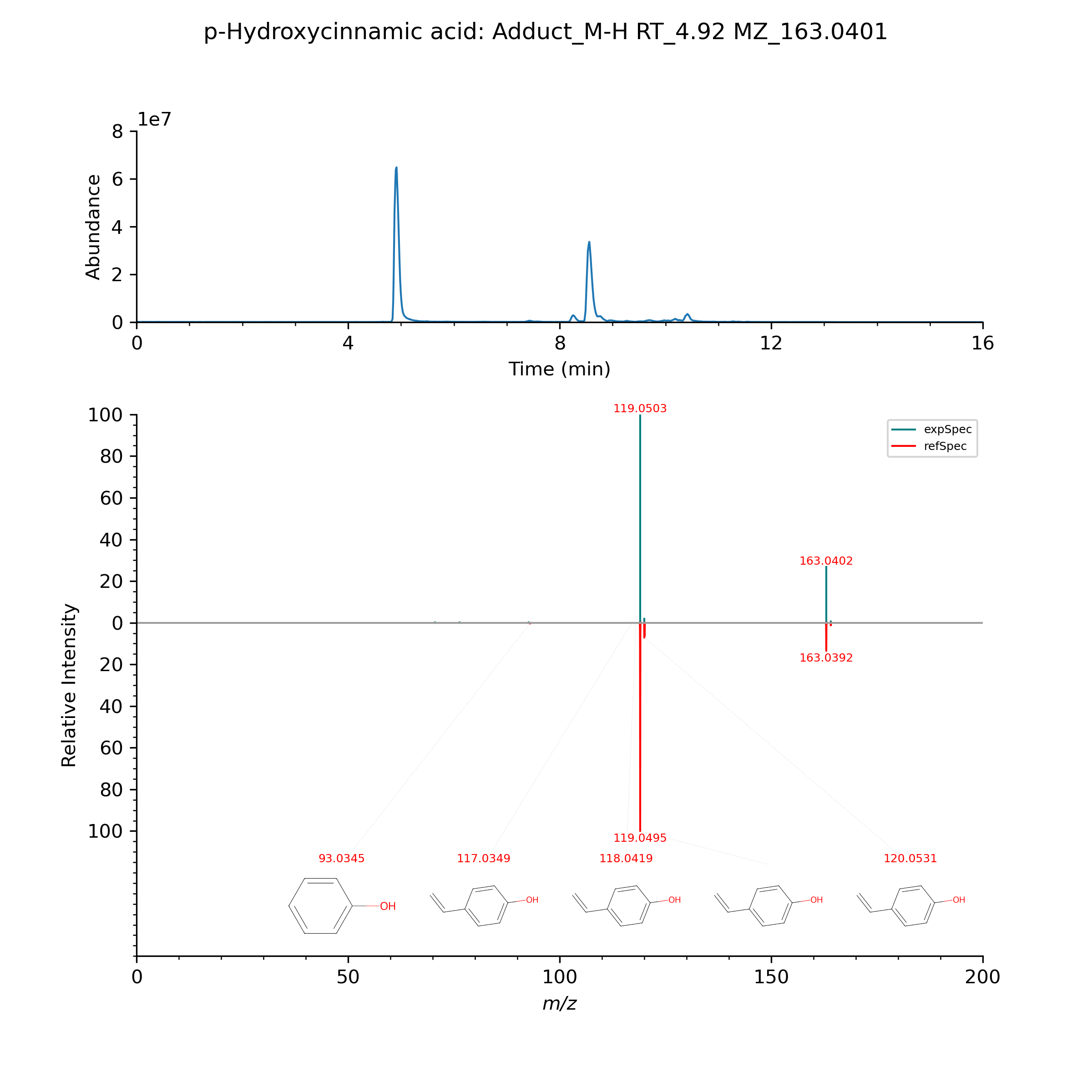

Supplement: Supplementary file 1 [file pharmaceuticals-18-01153-s001.zip › compound structures/M0113.png]

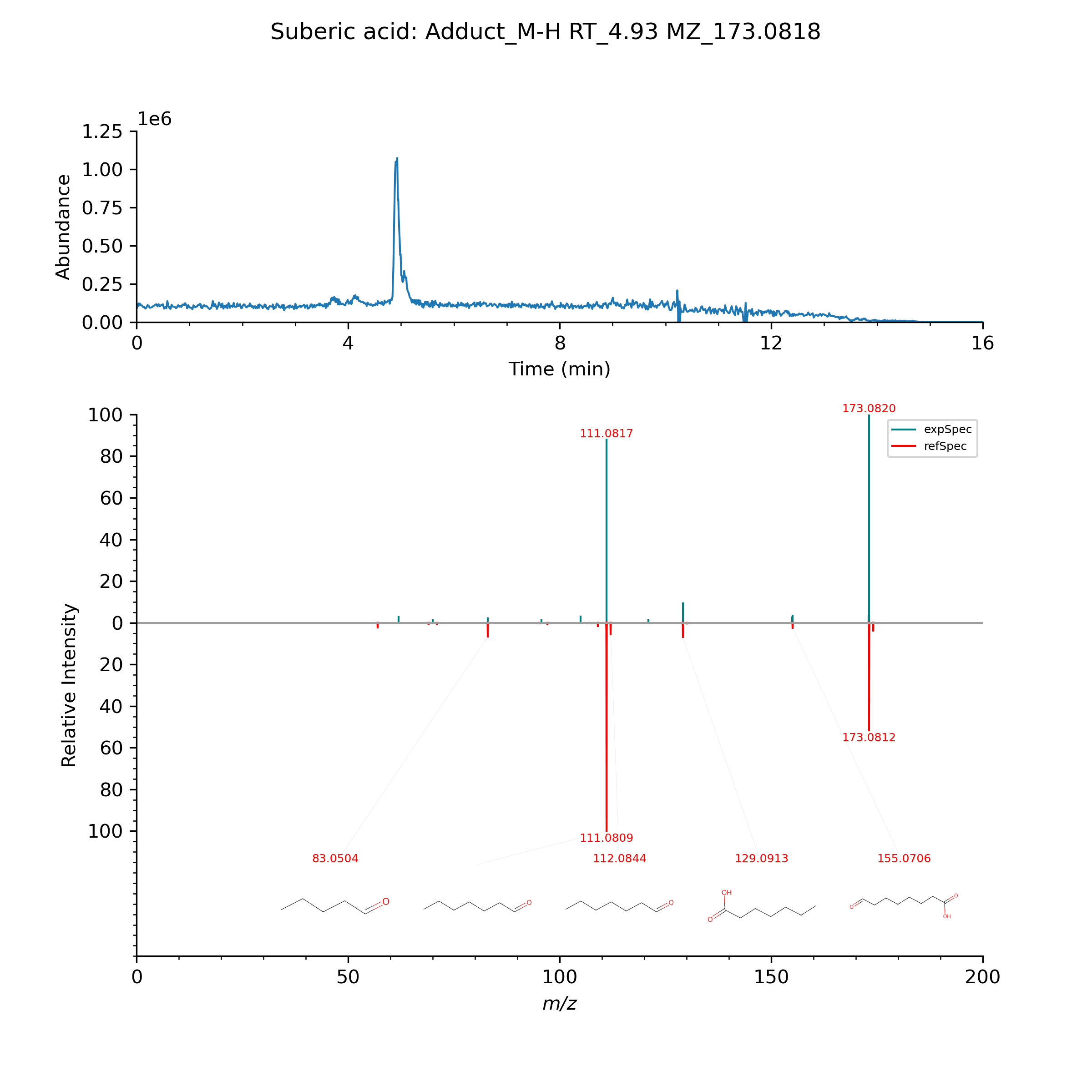

Supplement: Supplementary file 1 [file pharmaceuticals-18-01153-s001.zip › compound structures/M0114.png]

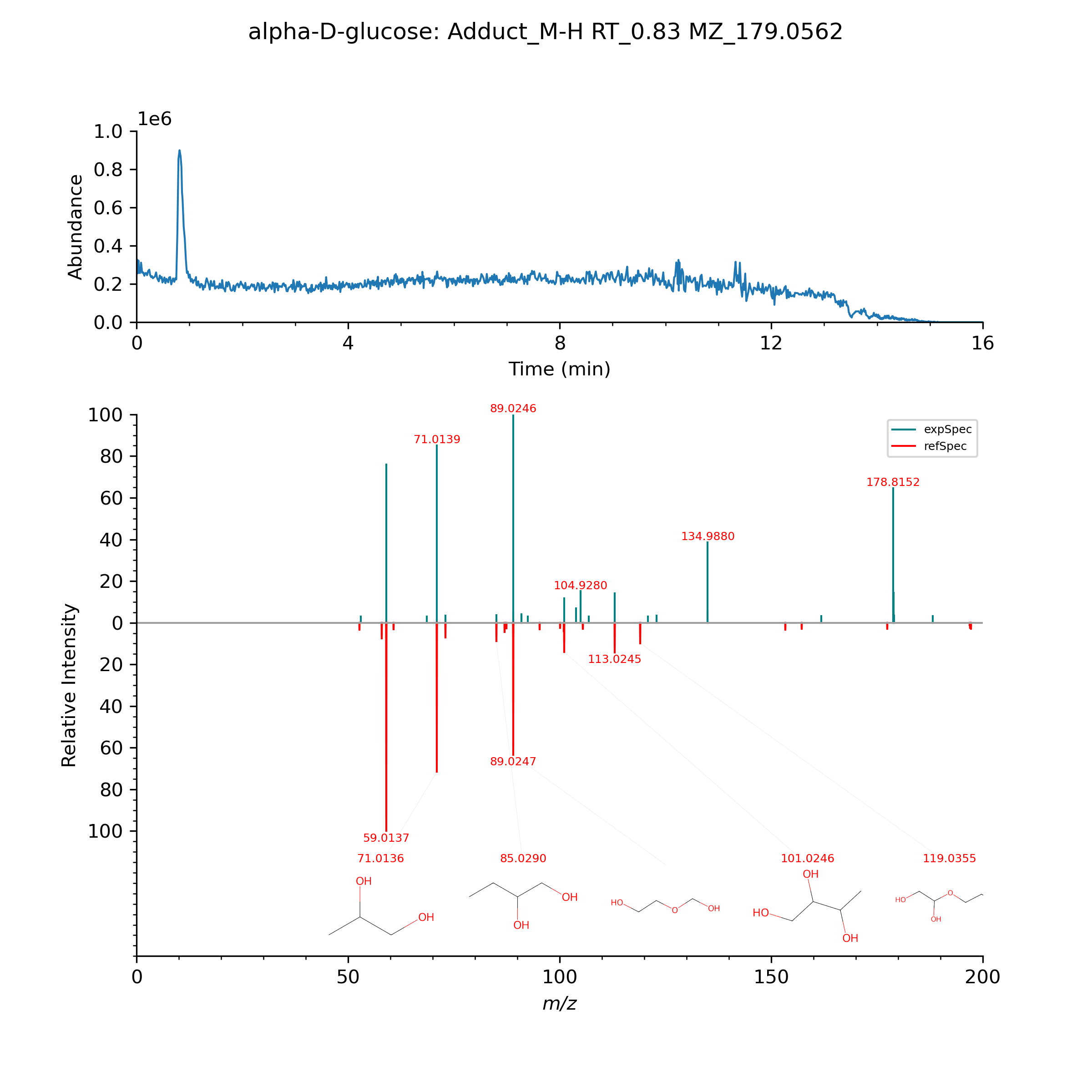

Supplement: Supplementary file 1 [file pharmaceuticals-18-01153-s001.zip › compound structures/M0115.png]

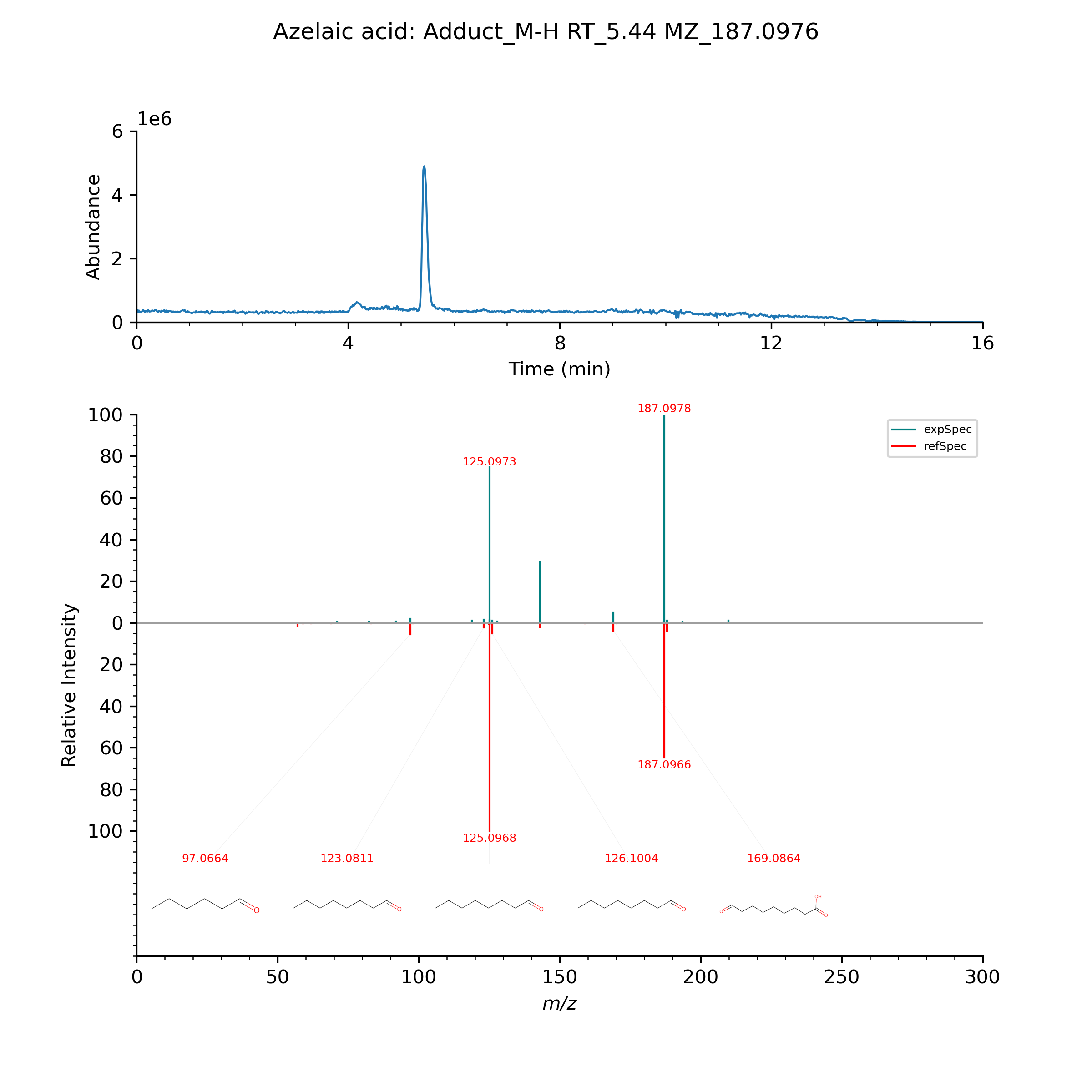

Supplement: Supplementary file 1 [file pharmaceuticals-18-01153-s001.zip › compound structures/M0116.png]

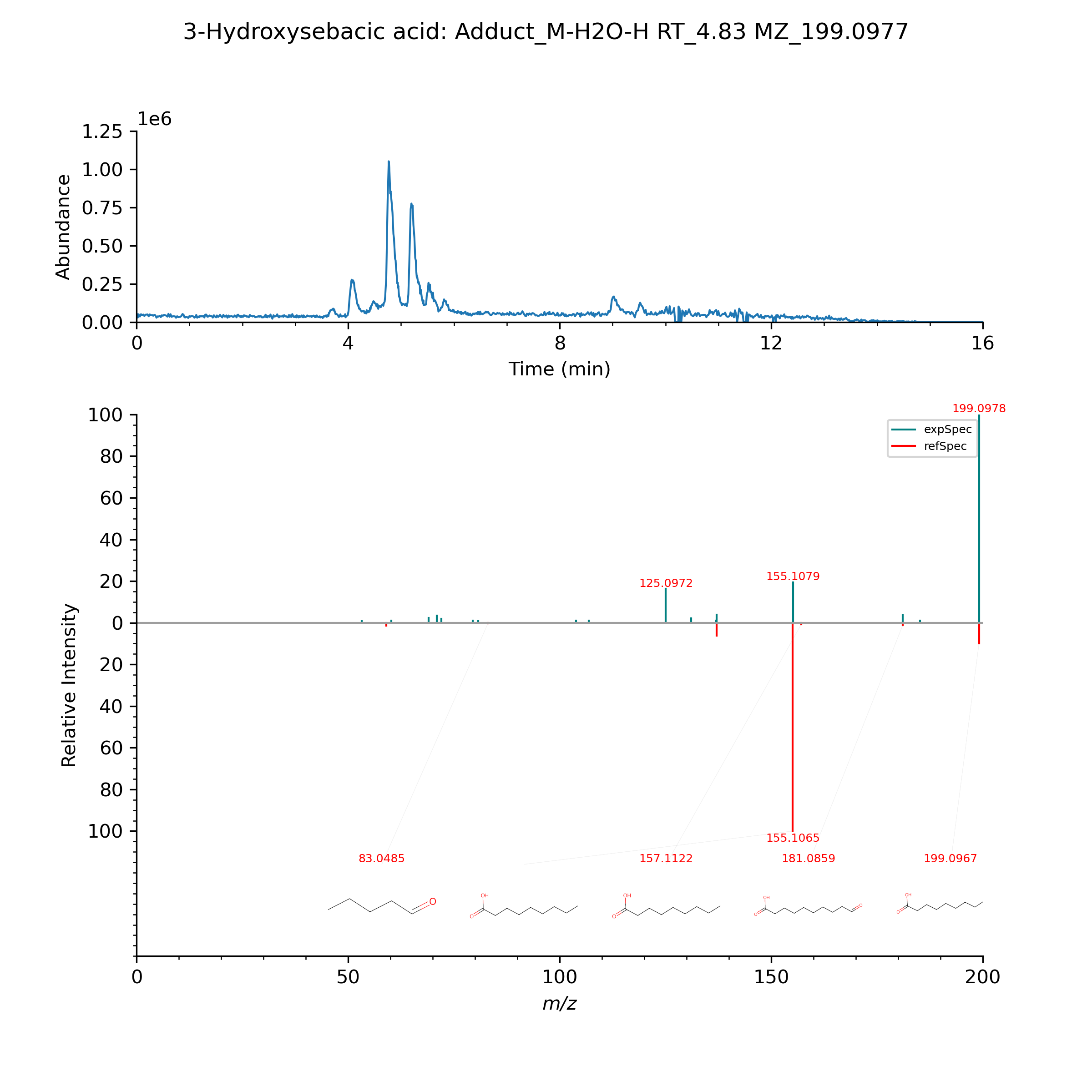

Supplement: Supplementary file 1 [file pharmaceuticals-18-01153-s001.zip › compound structures/M0117.png]

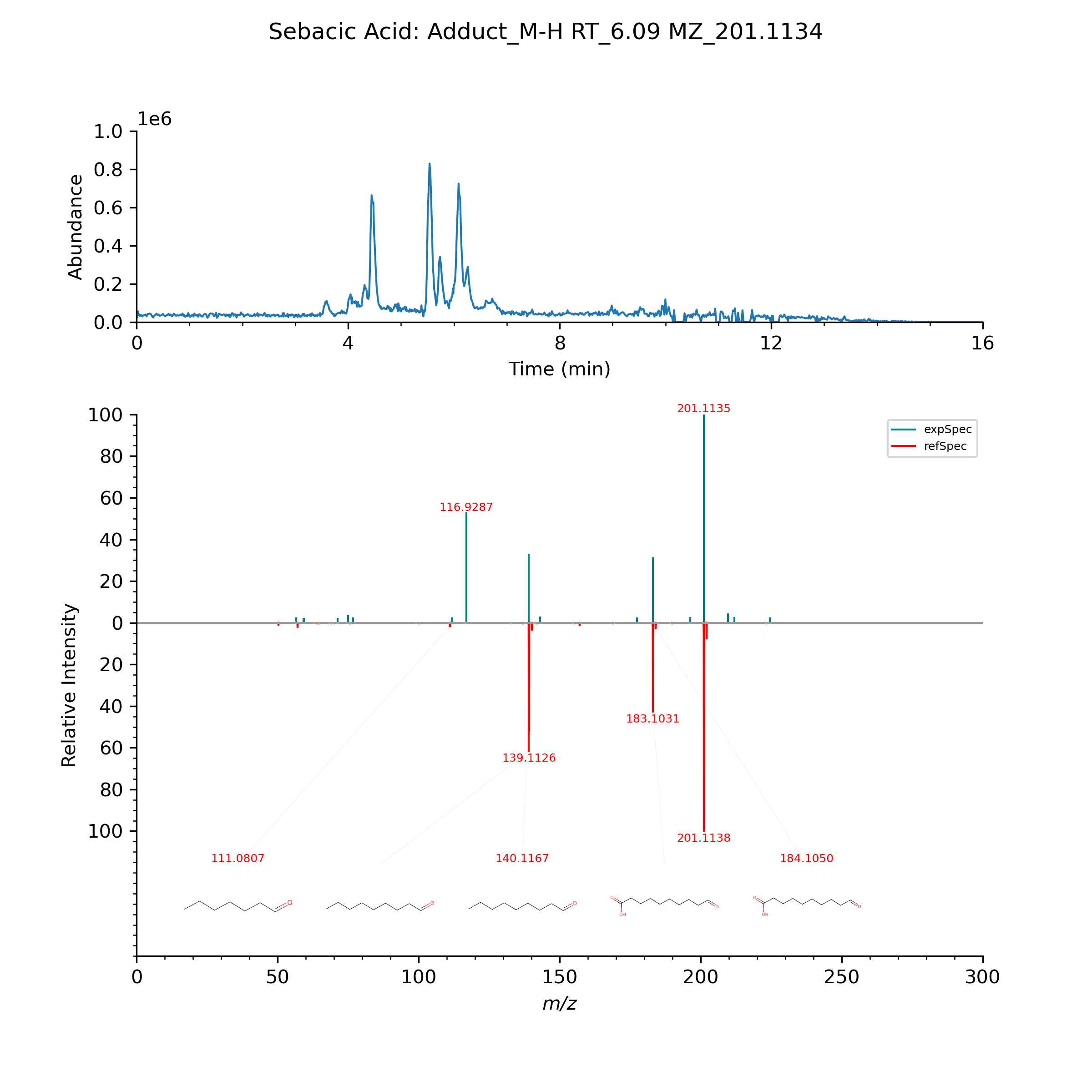

Supplement: Supplementary file 1 [file pharmaceuticals-18-01153-s001.zip › compound structures/M0118.png]

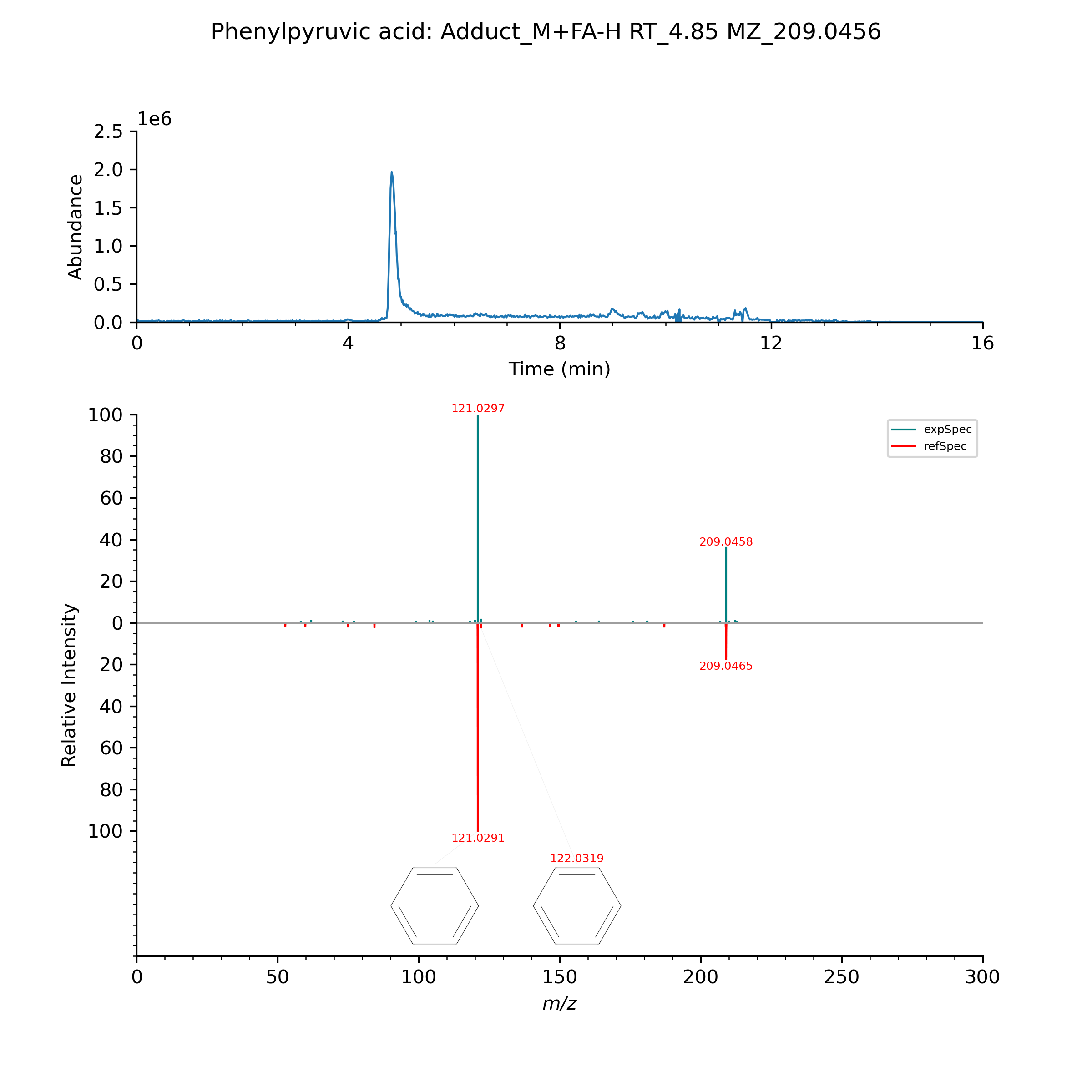

Supplement: Supplementary file 1 [file pharmaceuticals-18-01153-s001.zip › compound structures/M0119.png]

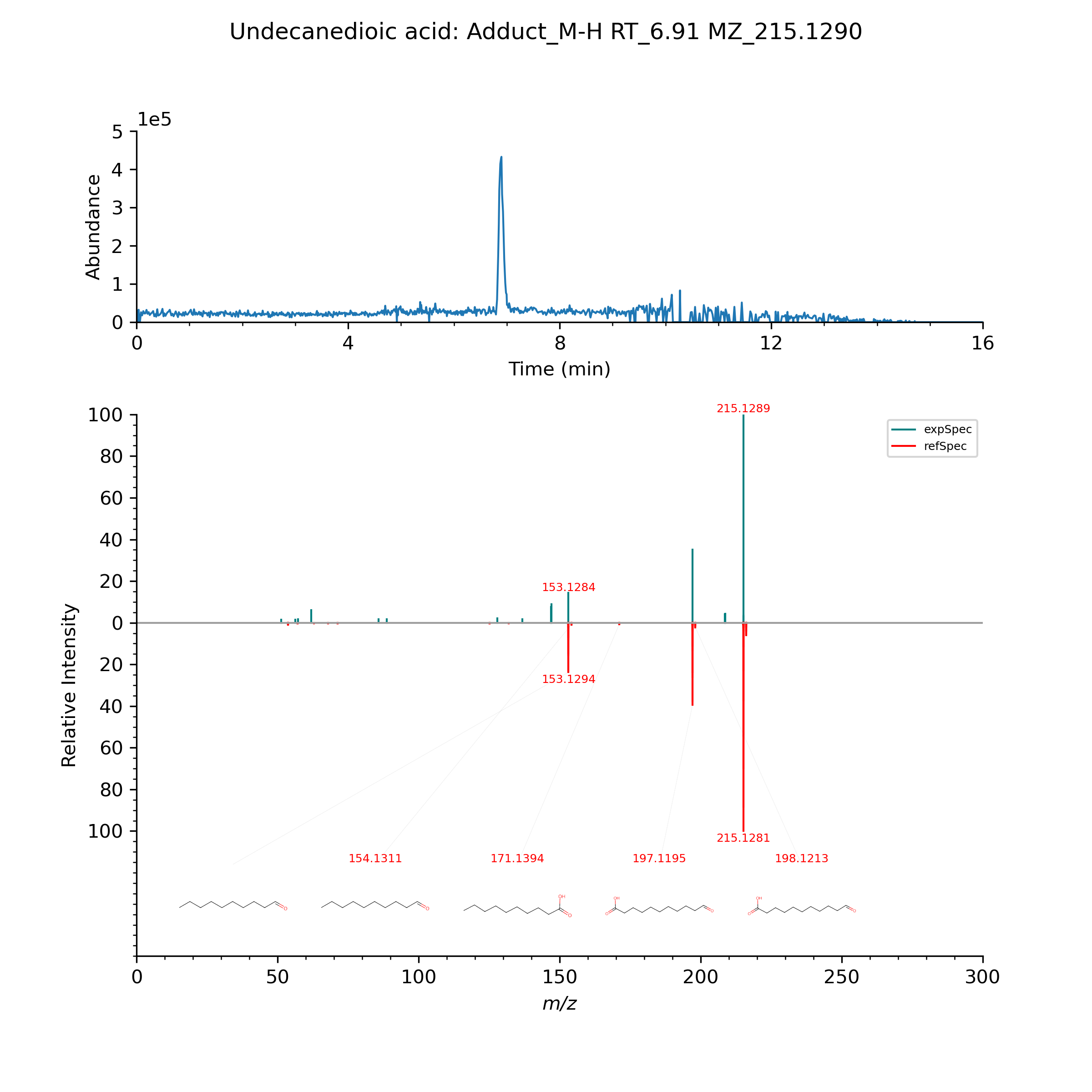

Supplement: Supplementary file 1 [file pharmaceuticals-18-01153-s001.zip › compound structures/M0120.png]

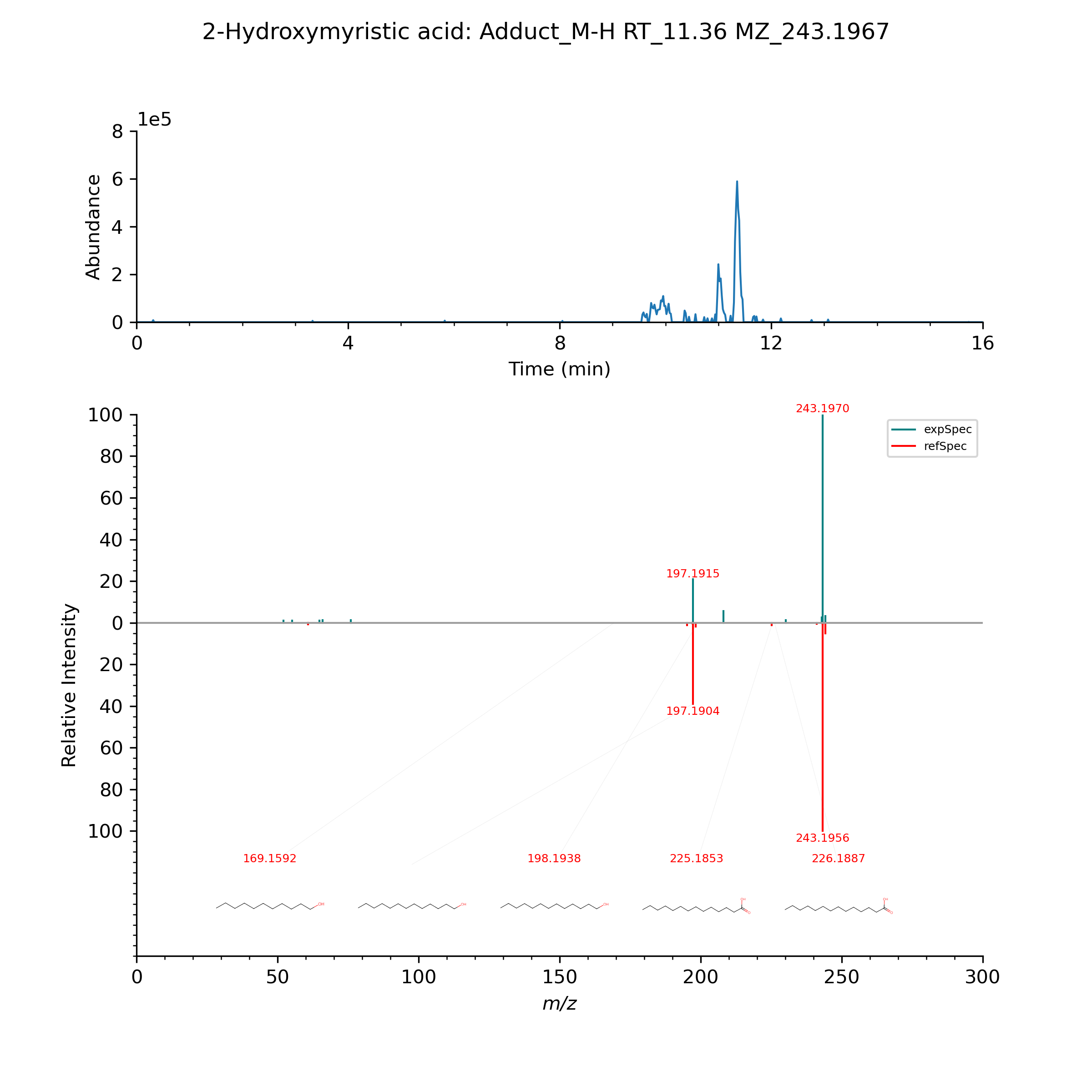

Supplement: Supplementary file 1 [file pharmaceuticals-18-01153-s001.zip › compound structures/M0121.png]

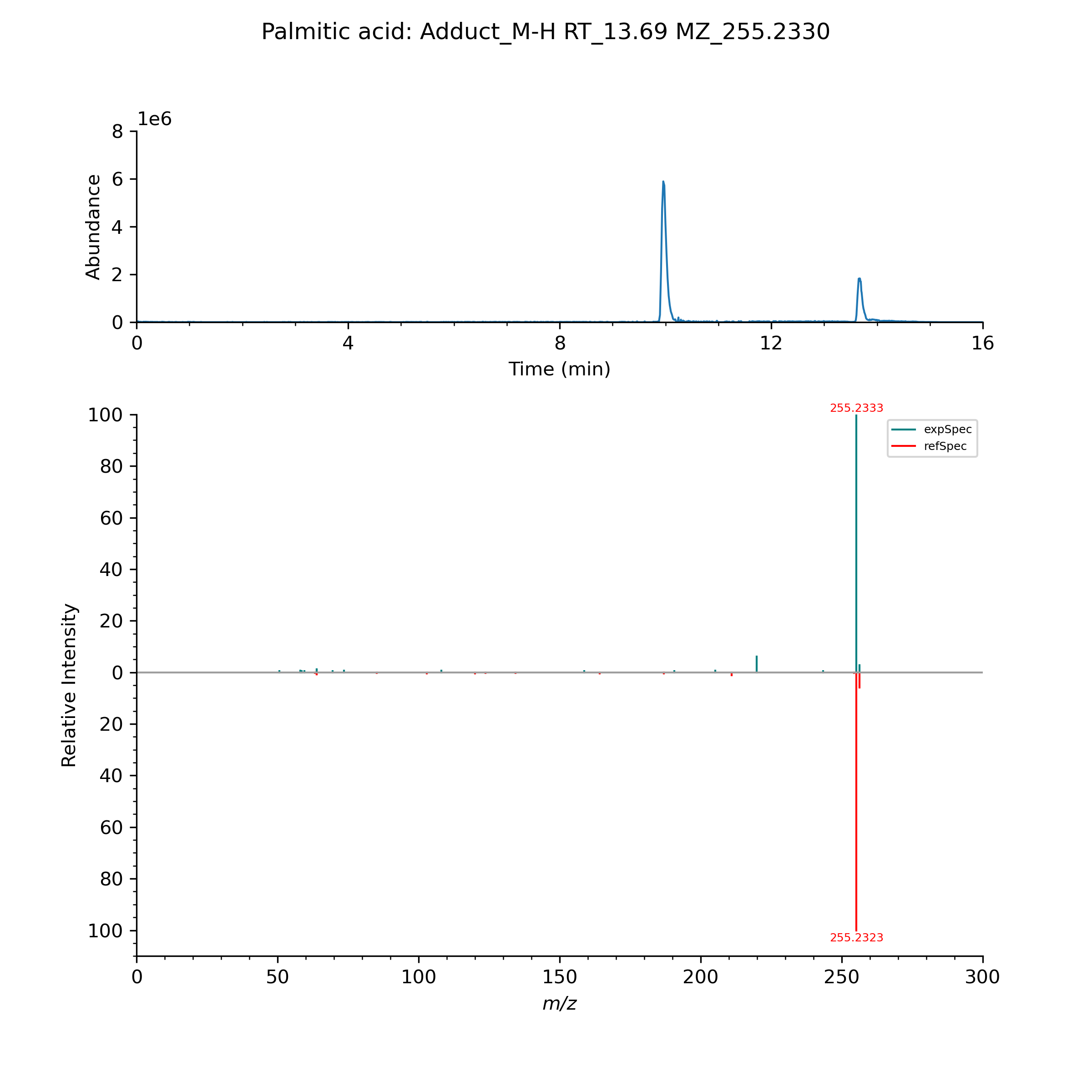

Supplement: Supplementary file 1 [file pharmaceuticals-18-01153-s001.zip › compound structures/M0122.png]

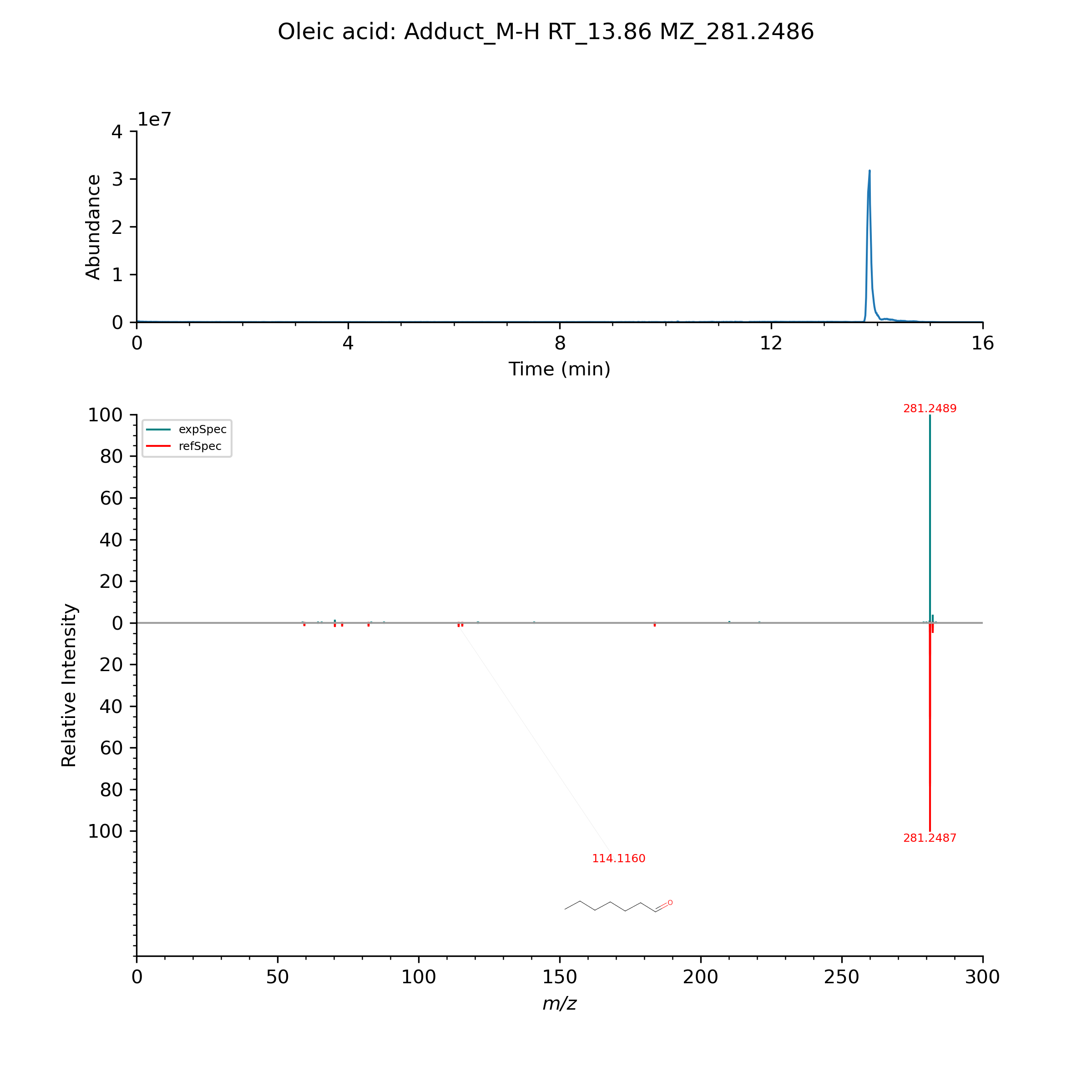

Supplement: Supplementary file 1 [file pharmaceuticals-18-01153-s001.zip › compound structures/M0123.png]

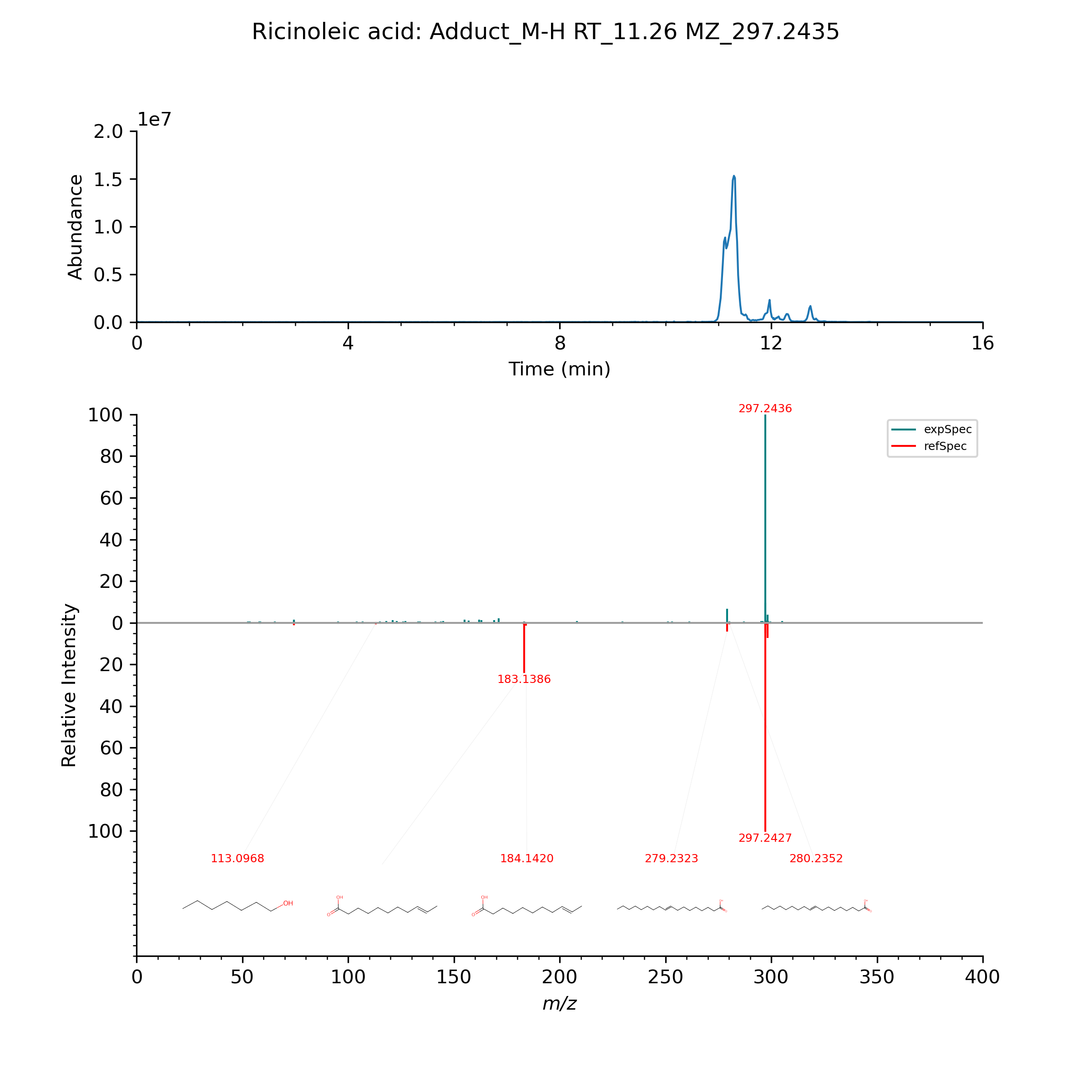

Supplement: Supplementary file 1 [file pharmaceuticals-18-01153-s001.zip › compound structures/M0124.png]

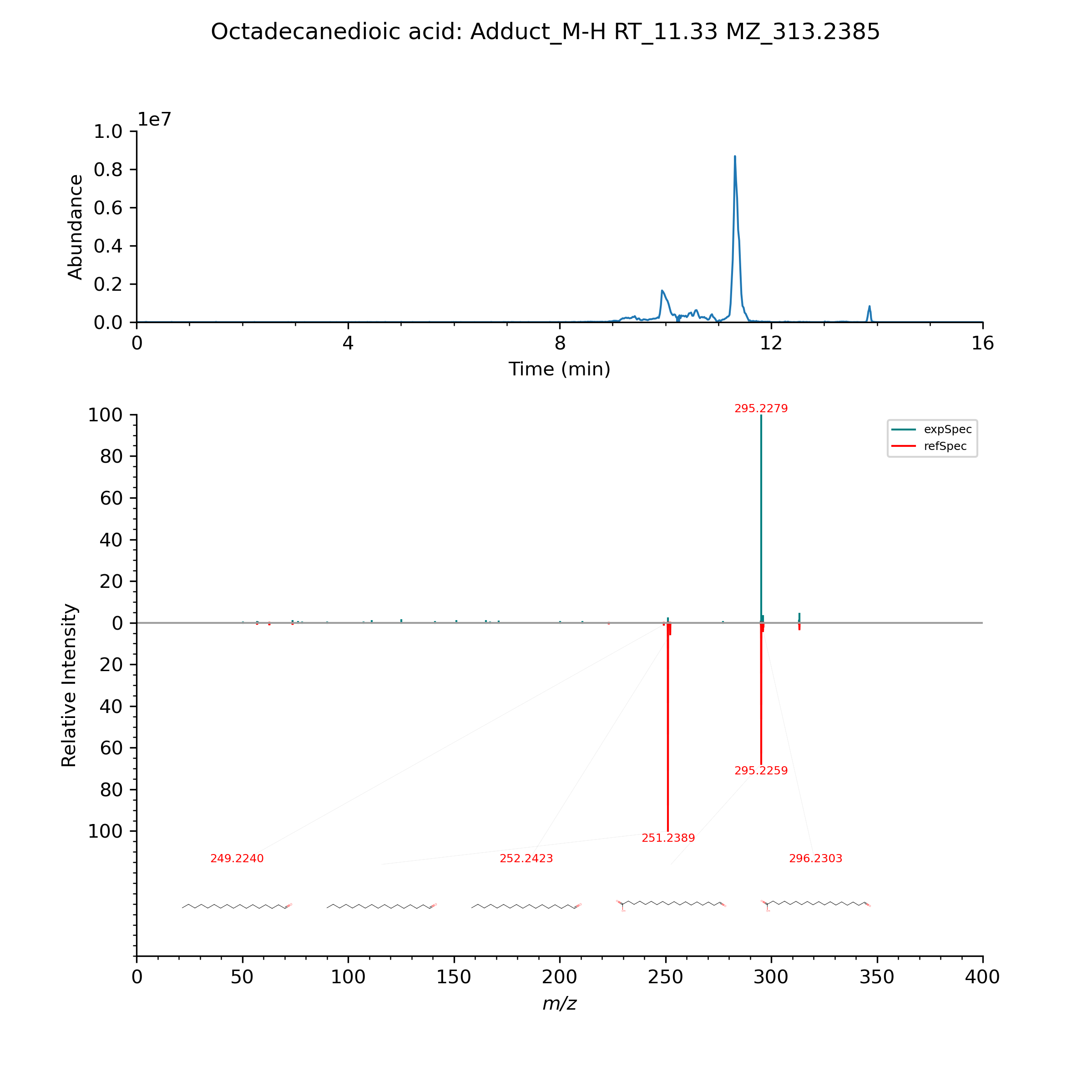

Supplement: Supplementary file 1 [file pharmaceuticals-18-01153-s001.zip › compound structures/M0125.png]

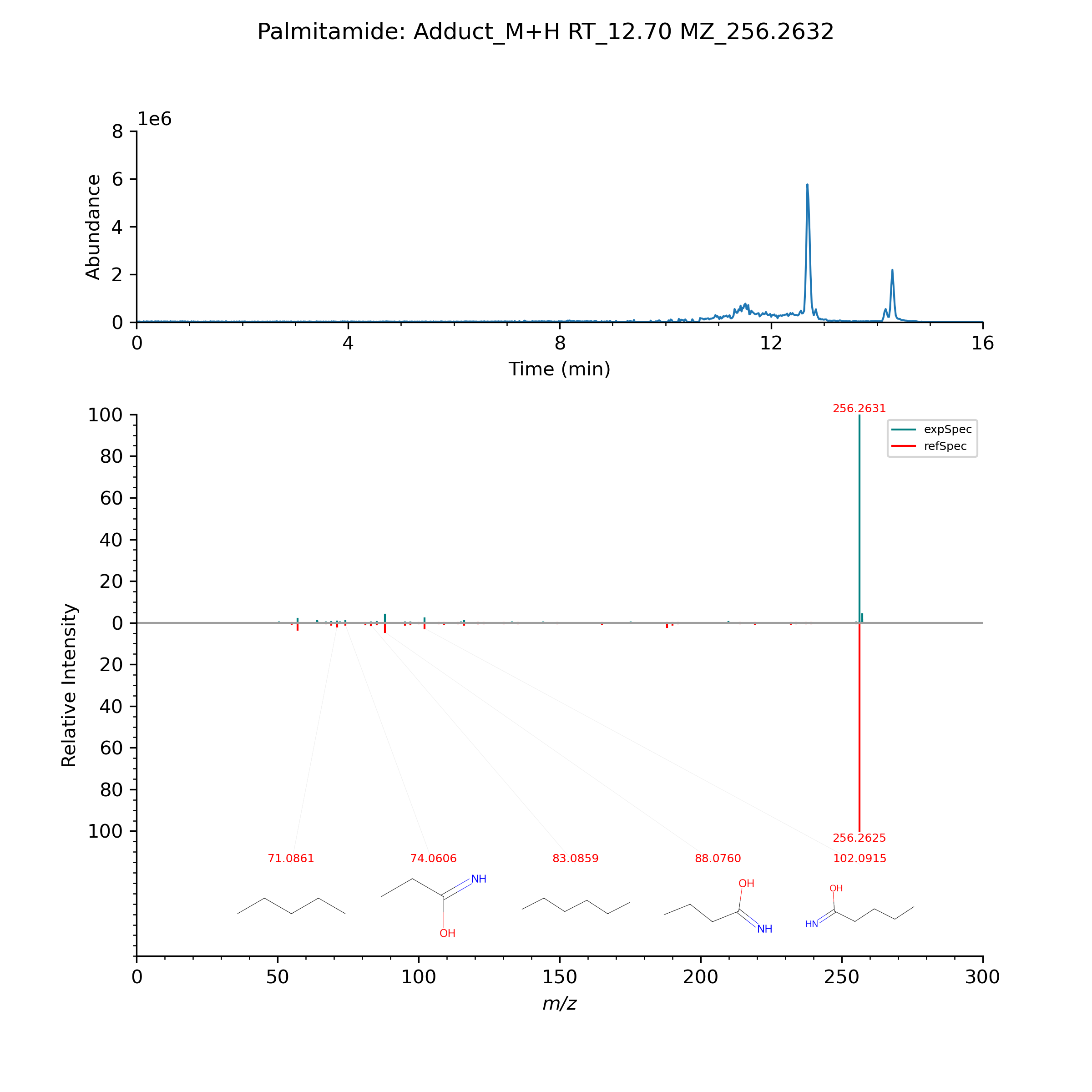

Supplement: Supplementary file 1 [file pharmaceuticals-18-01153-s001.zip › compound structures/M0126.png]

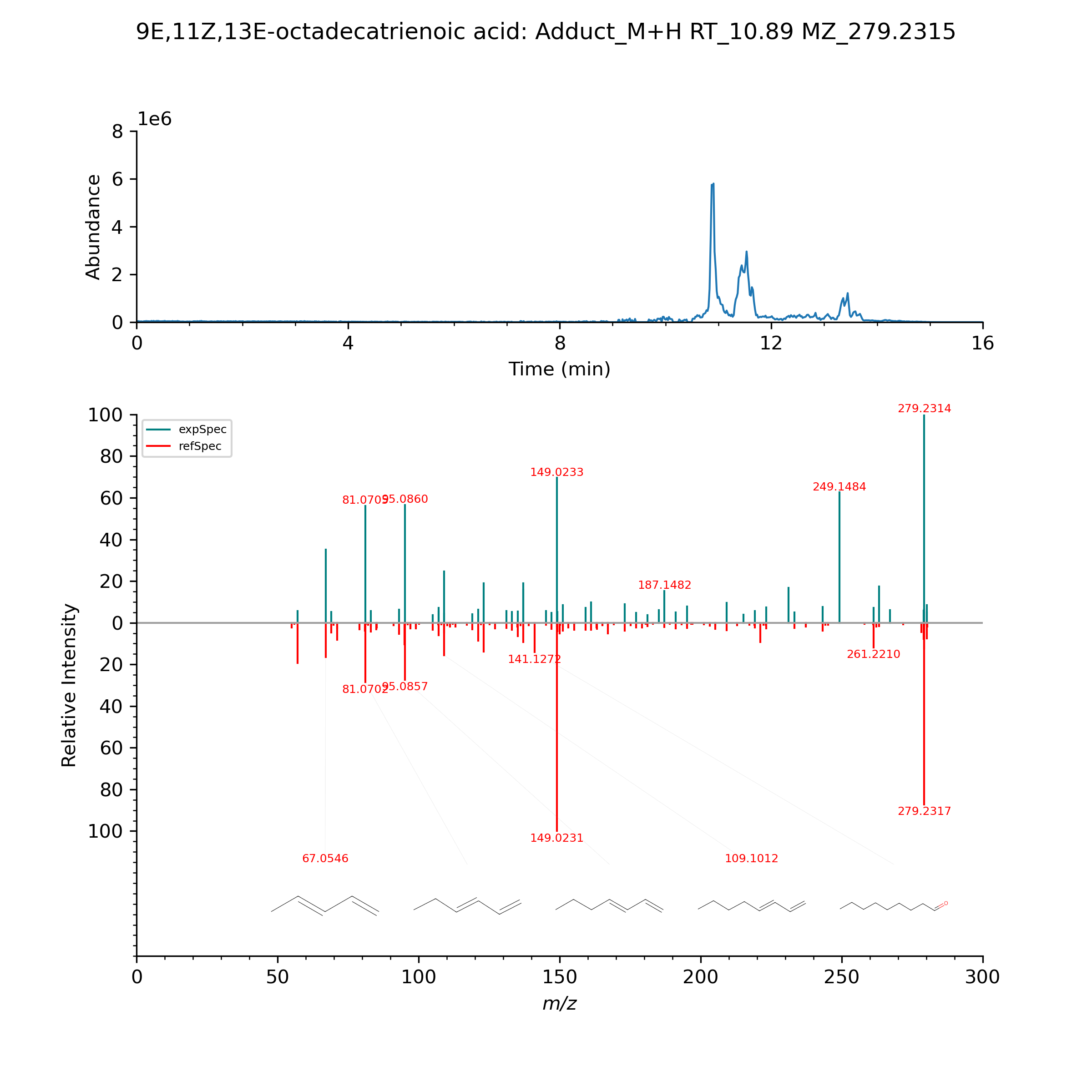

Supplement: Supplementary file 1 [file pharmaceuticals-18-01153-s001.zip › compound structures/M0127.png]

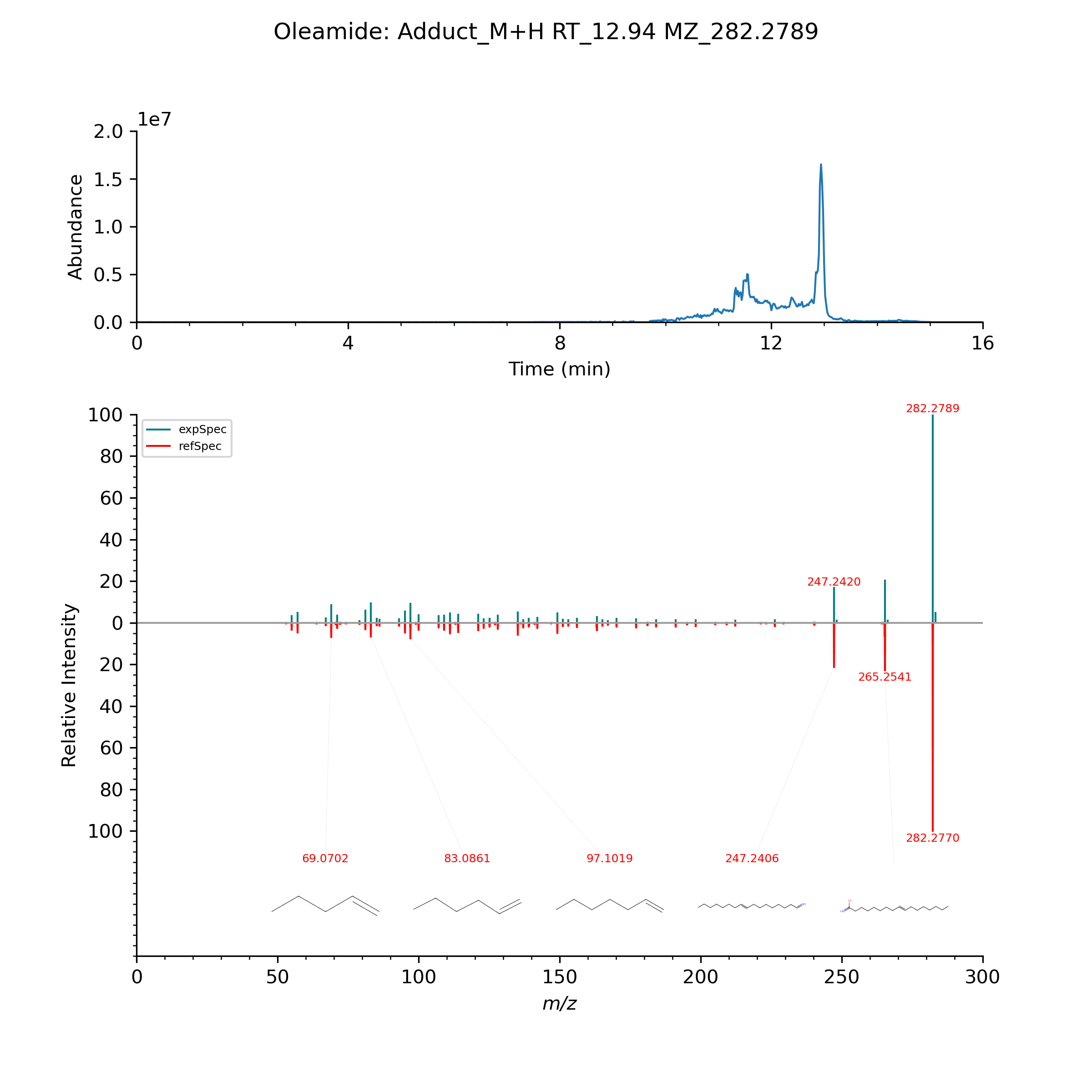

Supplement: Supplementary file 1 [file pharmaceuticals-18-01153-s001.zip › compound structures/M0128.png]

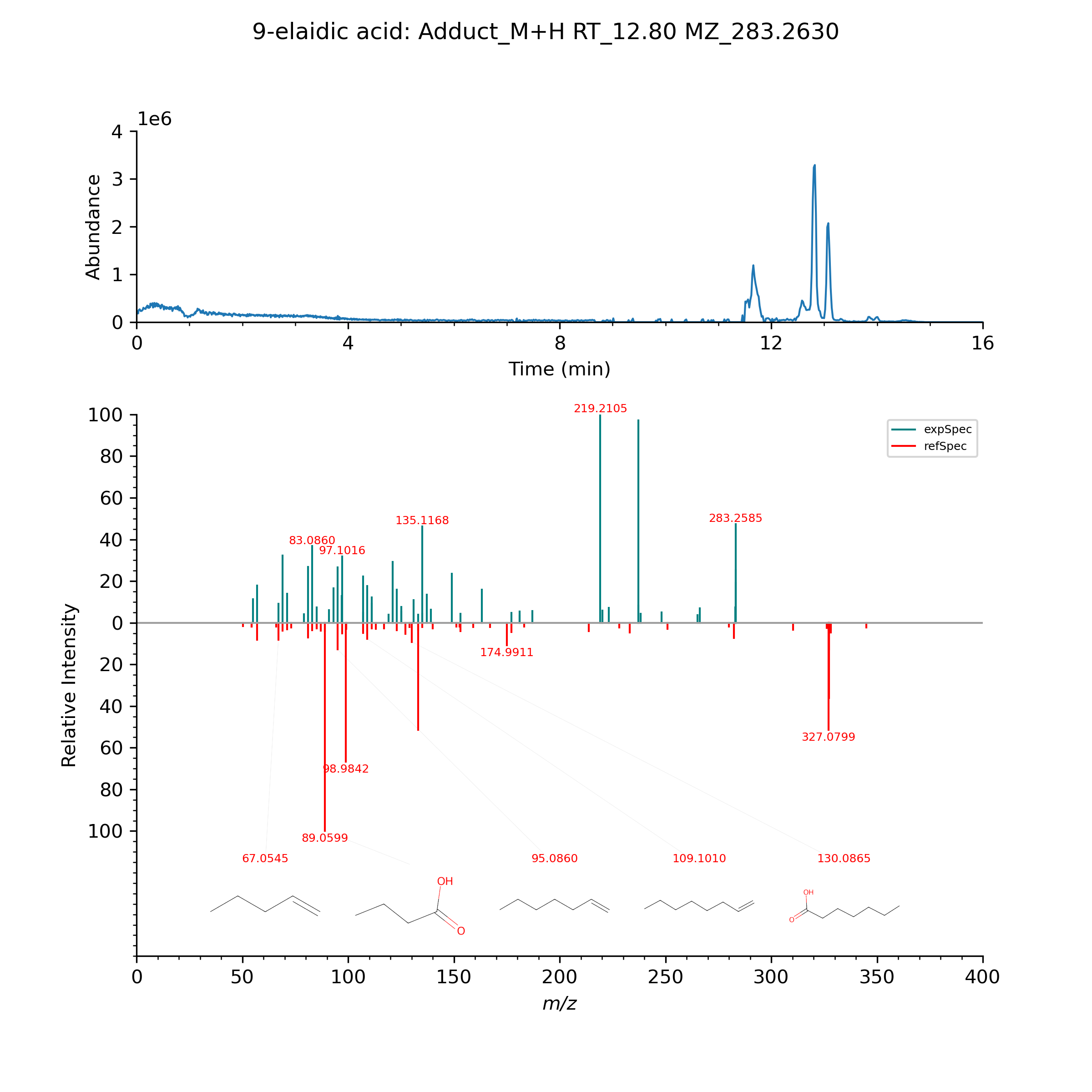

Supplement: Supplementary file 1 [file pharmaceuticals-18-01153-s001.zip › compound structures/M0129.png]

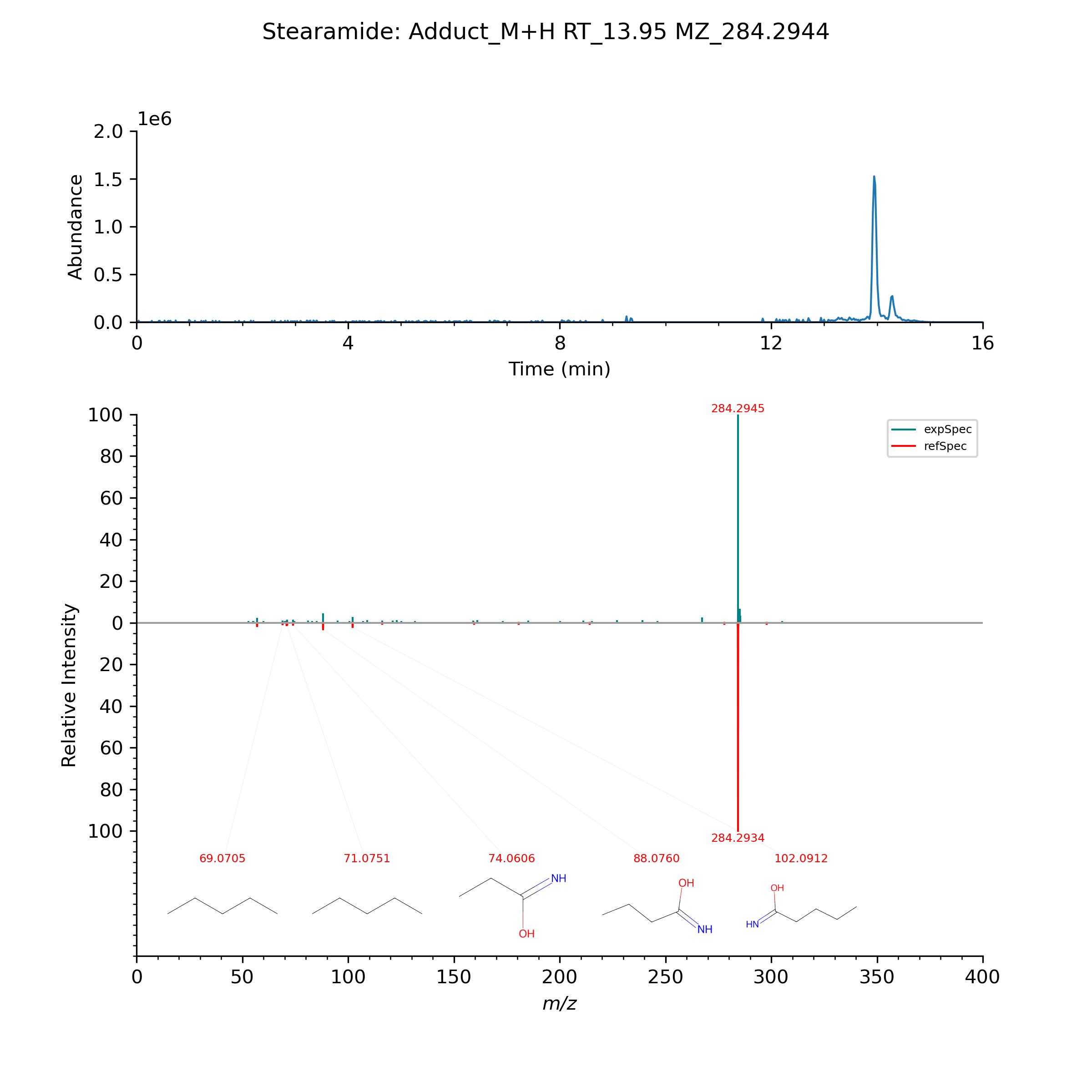

Supplement: Supplementary file 1 [file pharmaceuticals-18-01153-s001.zip › compound structures/M0130.png]

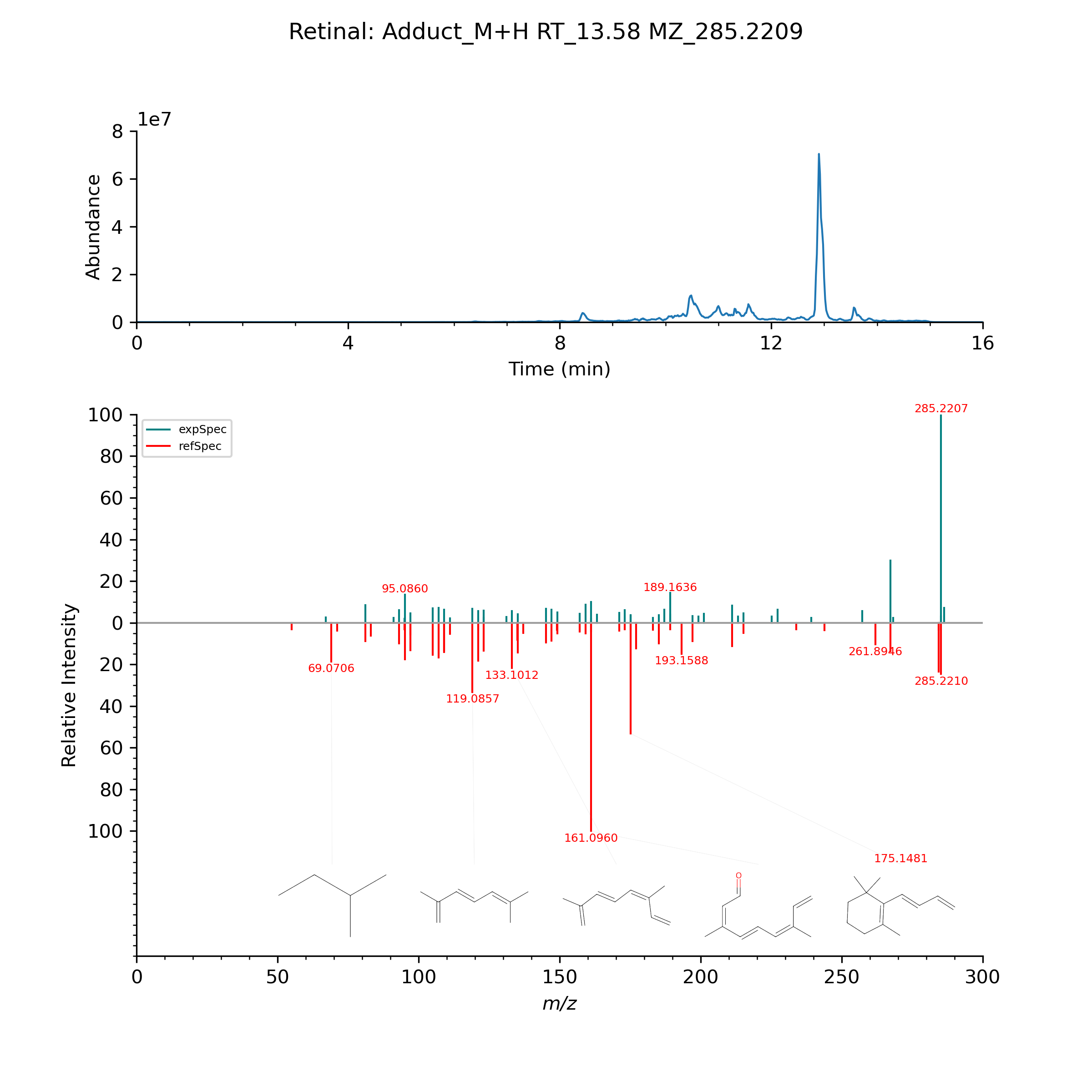

Supplement: Supplementary file 1 [file pharmaceuticals-18-01153-s001.zip › compound structures/M0131.png]

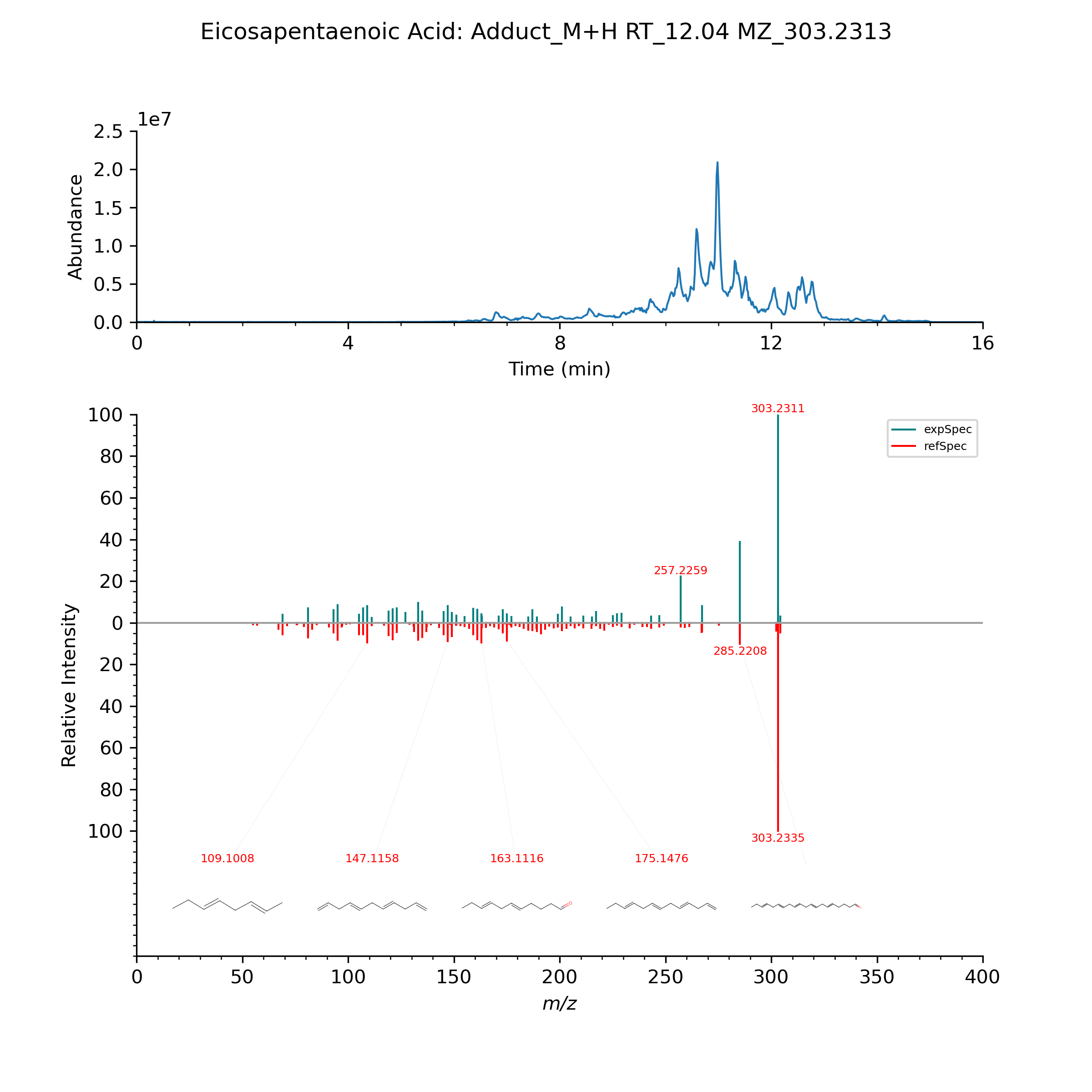

Supplement: Supplementary file 1 [file pharmaceuticals-18-01153-s001.zip › compound structures/M0132.png]

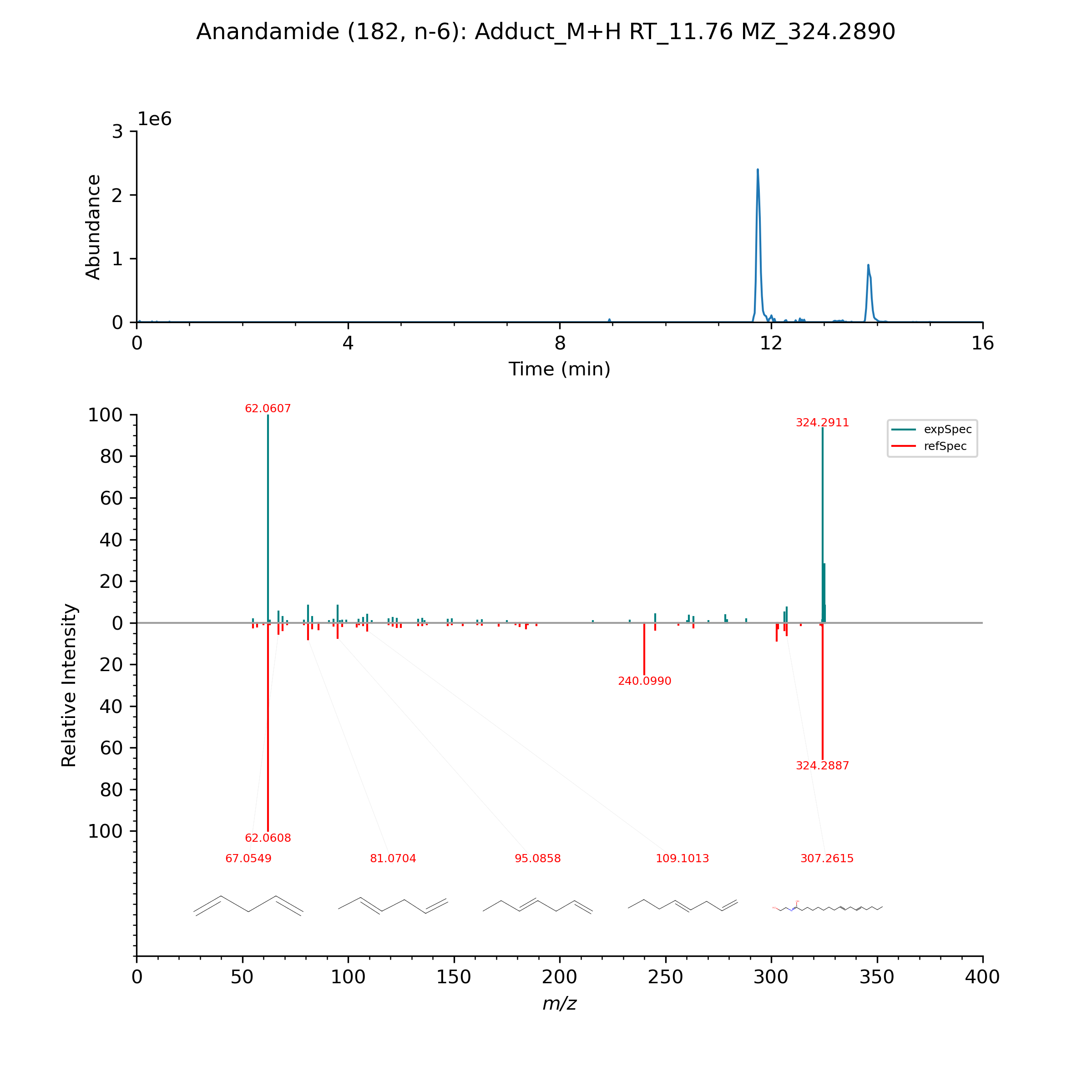

Supplement: Supplementary file 1 [file pharmaceuticals-18-01153-s001.zip › compound structures/M0133.png]

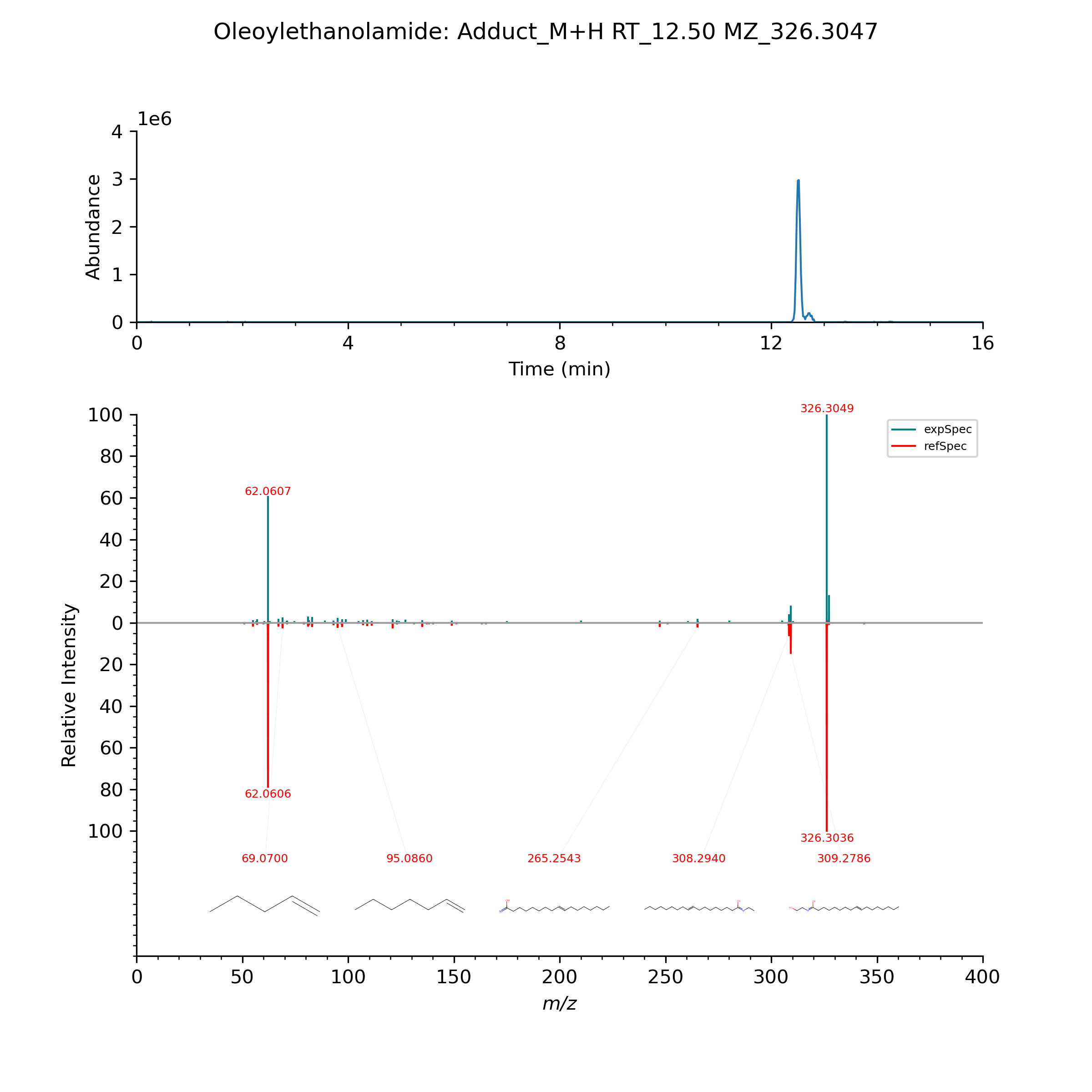

Supplement: Supplementary file 1 [file pharmaceuticals-18-01153-s001.zip › compound structures/M0134.png]

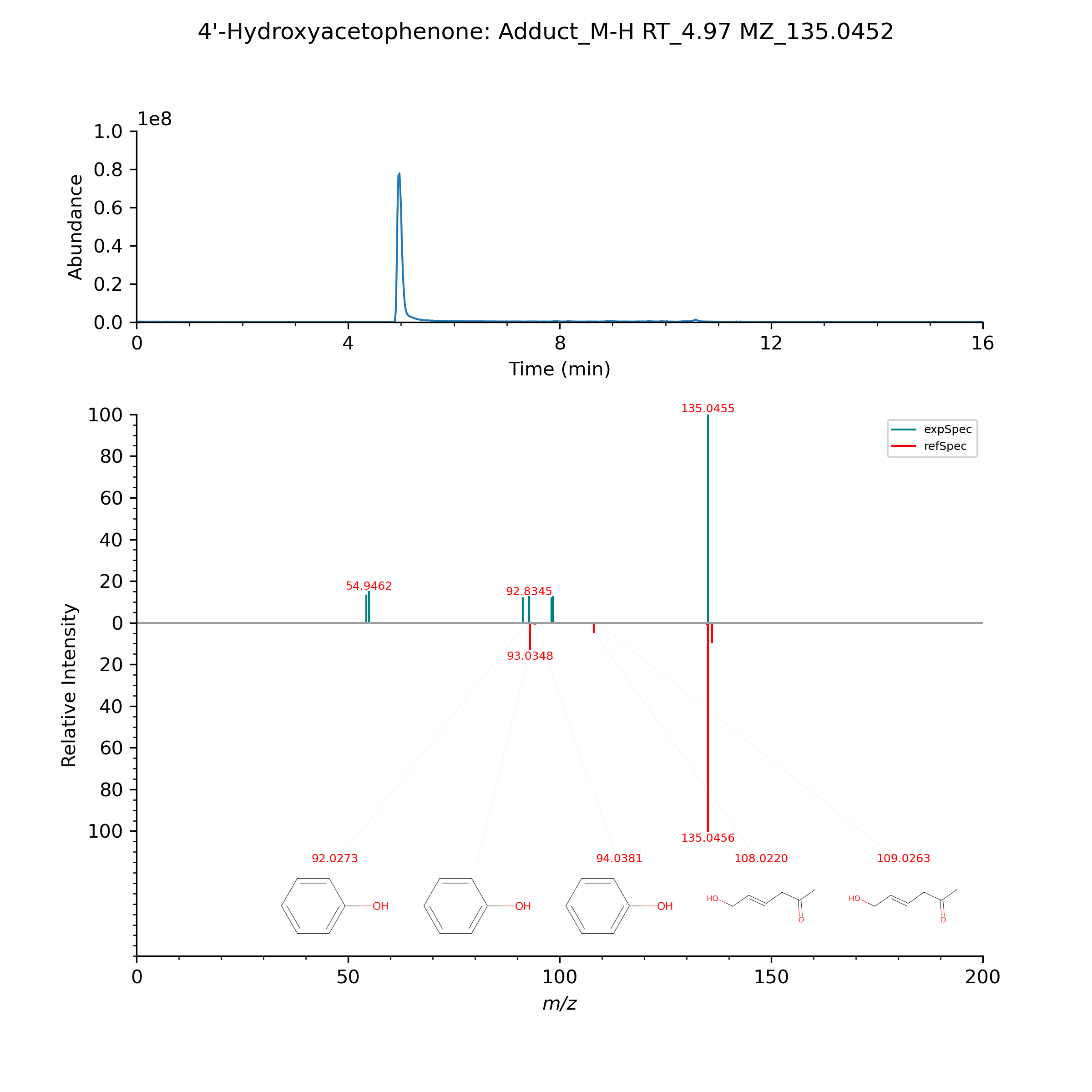

Supplement: Supplementary file 1 [file pharmaceuticals-18-01153-s001.zip › compound structures/M0135.png]

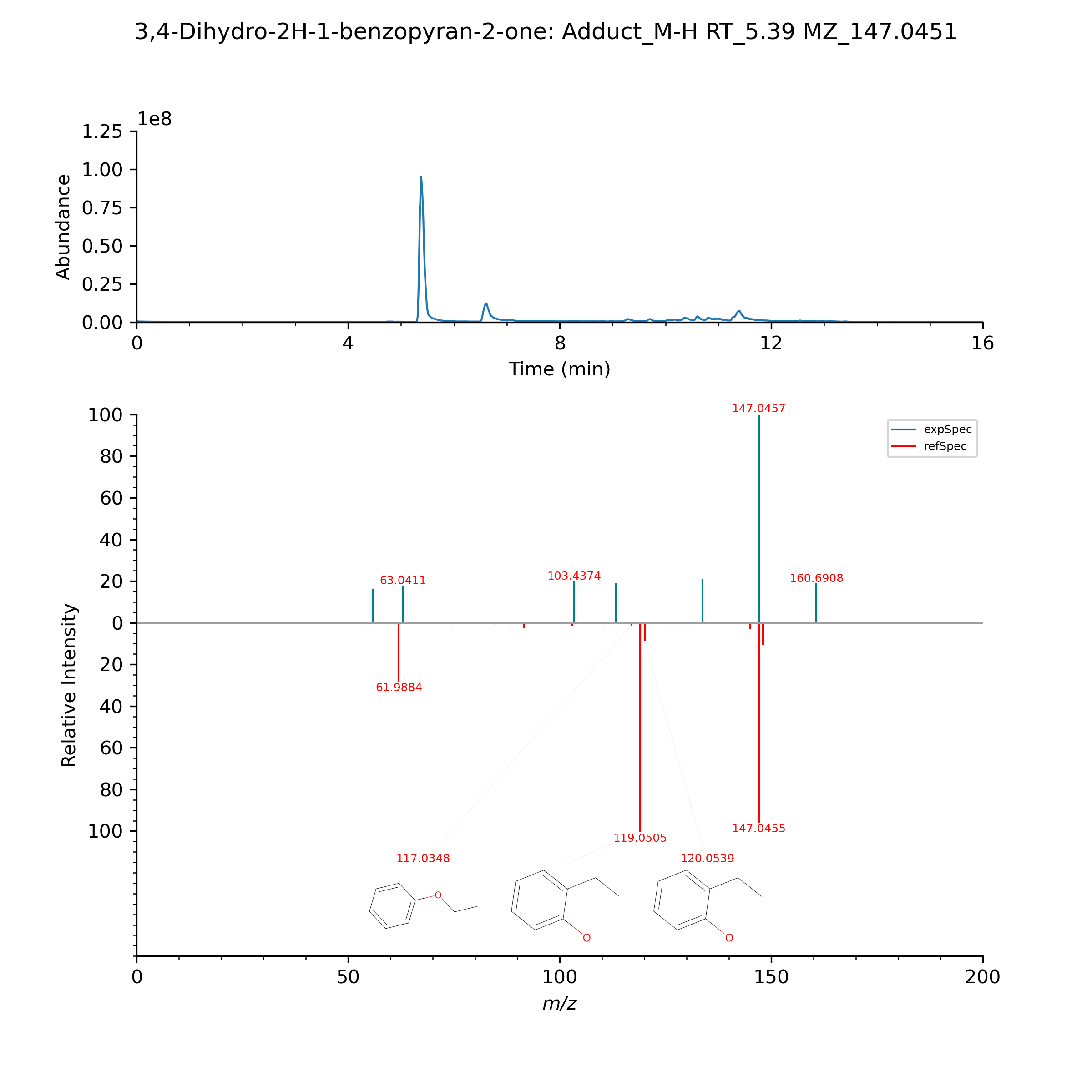

Supplement: Supplementary file 1 [file pharmaceuticals-18-01153-s001.zip › compound structures/M0136.png]

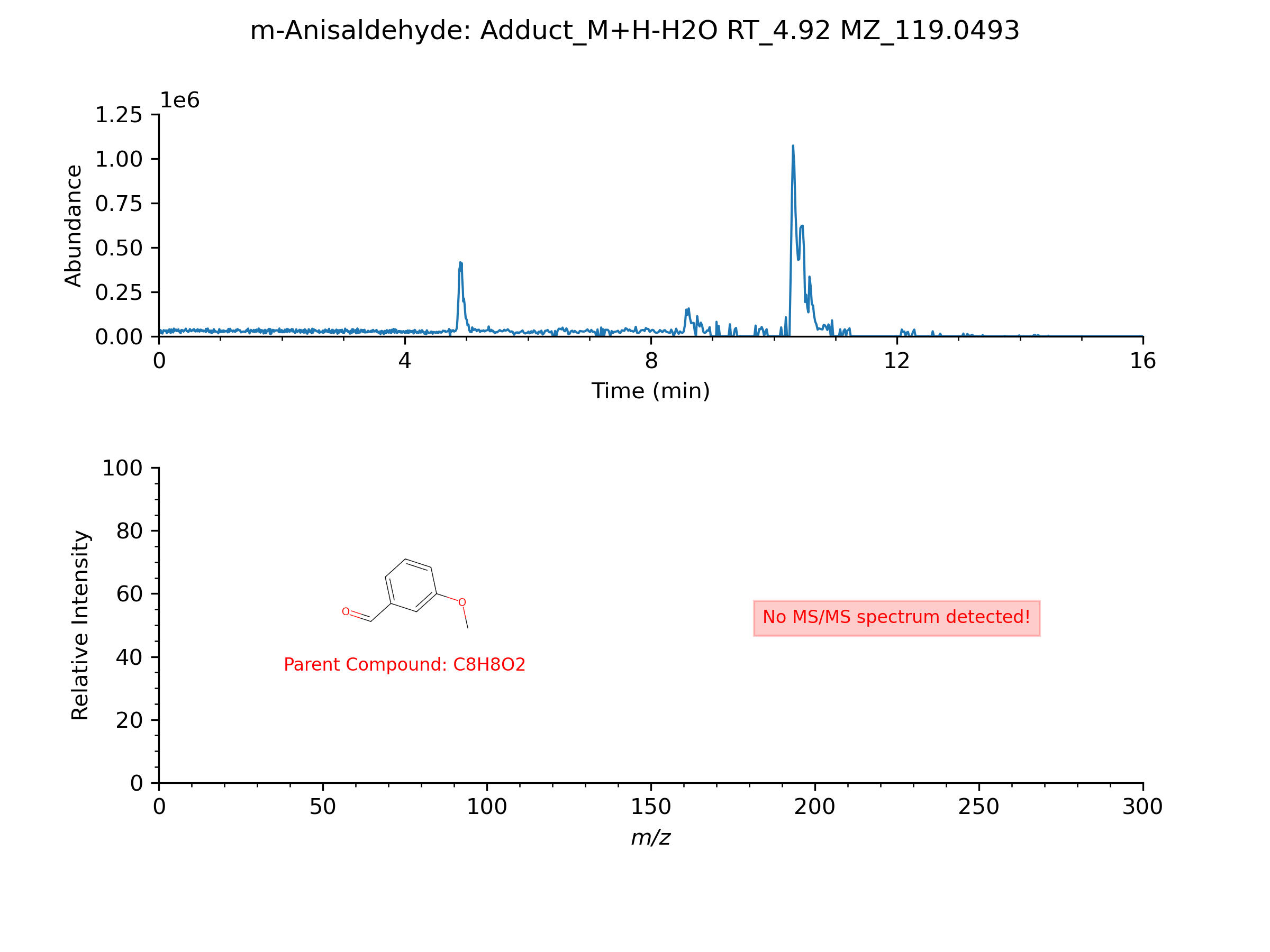

Supplement: Supplementary file 1 [file pharmaceuticals-18-01153-s001.zip › compound structures/M0137.png]

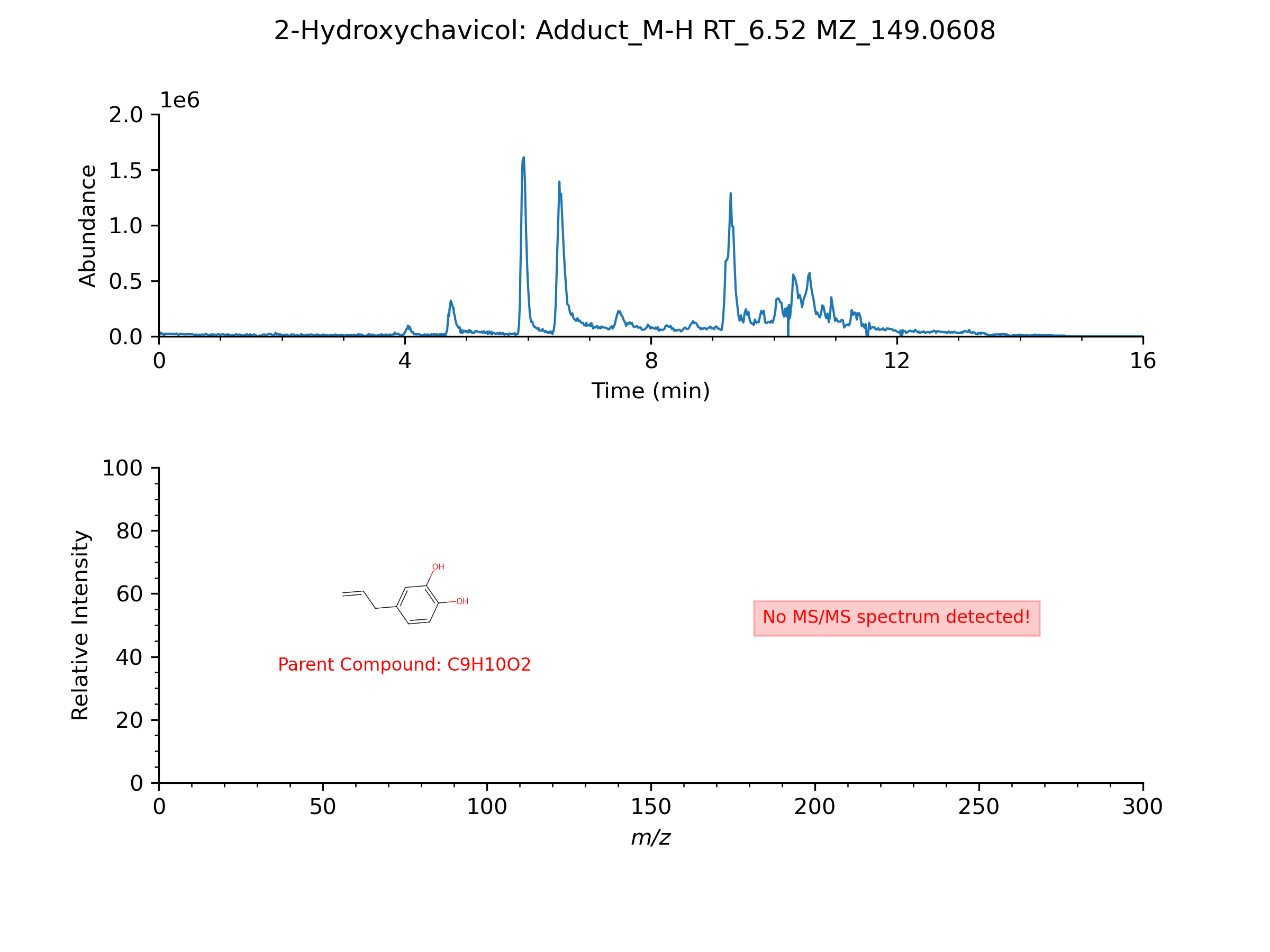

Supplement: Supplementary file 1 [file pharmaceuticals-18-01153-s001.zip › compound structures/M0138.png]

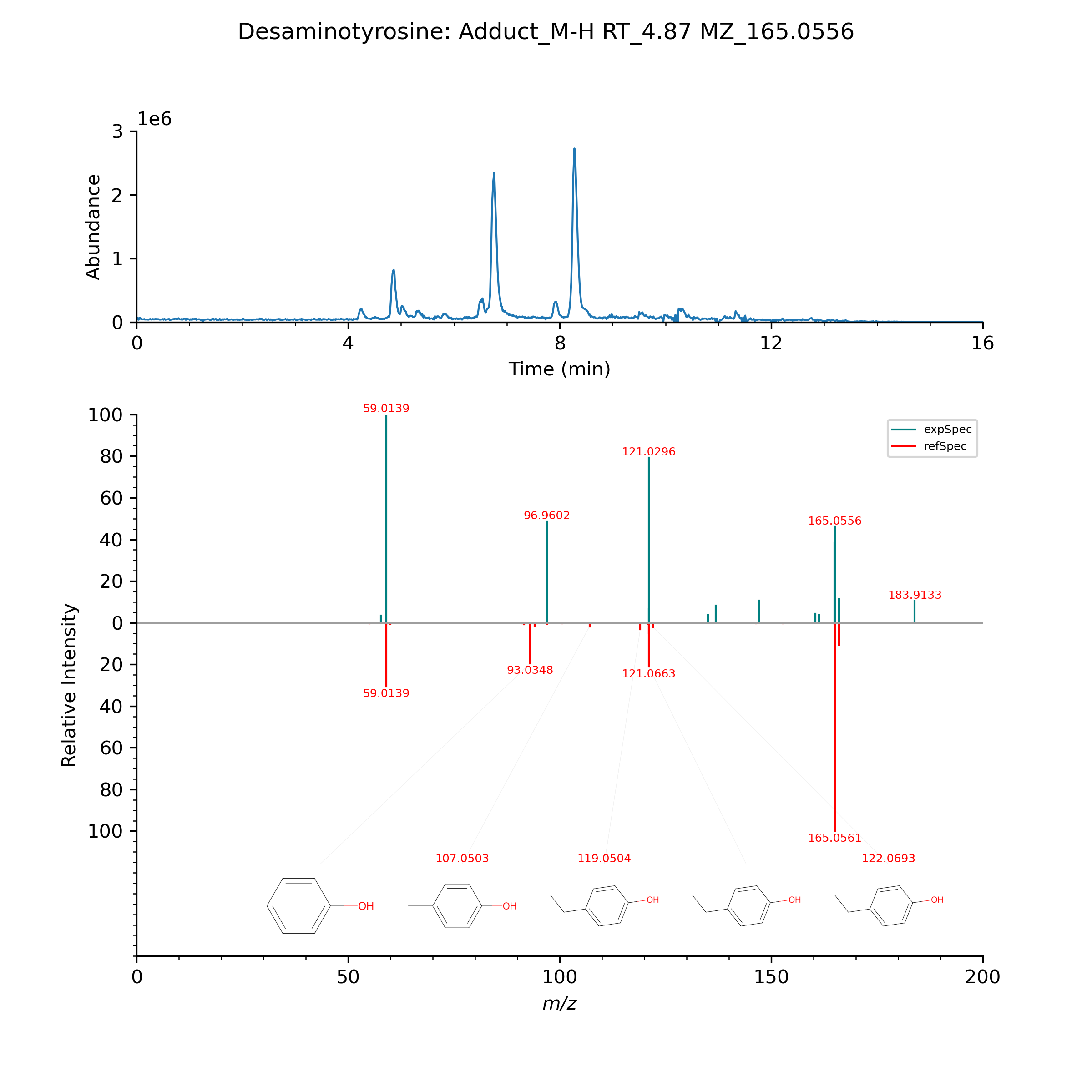

Supplement: Supplementary file 1 [file pharmaceuticals-18-01153-s001.zip › compound structures/M0139.png]

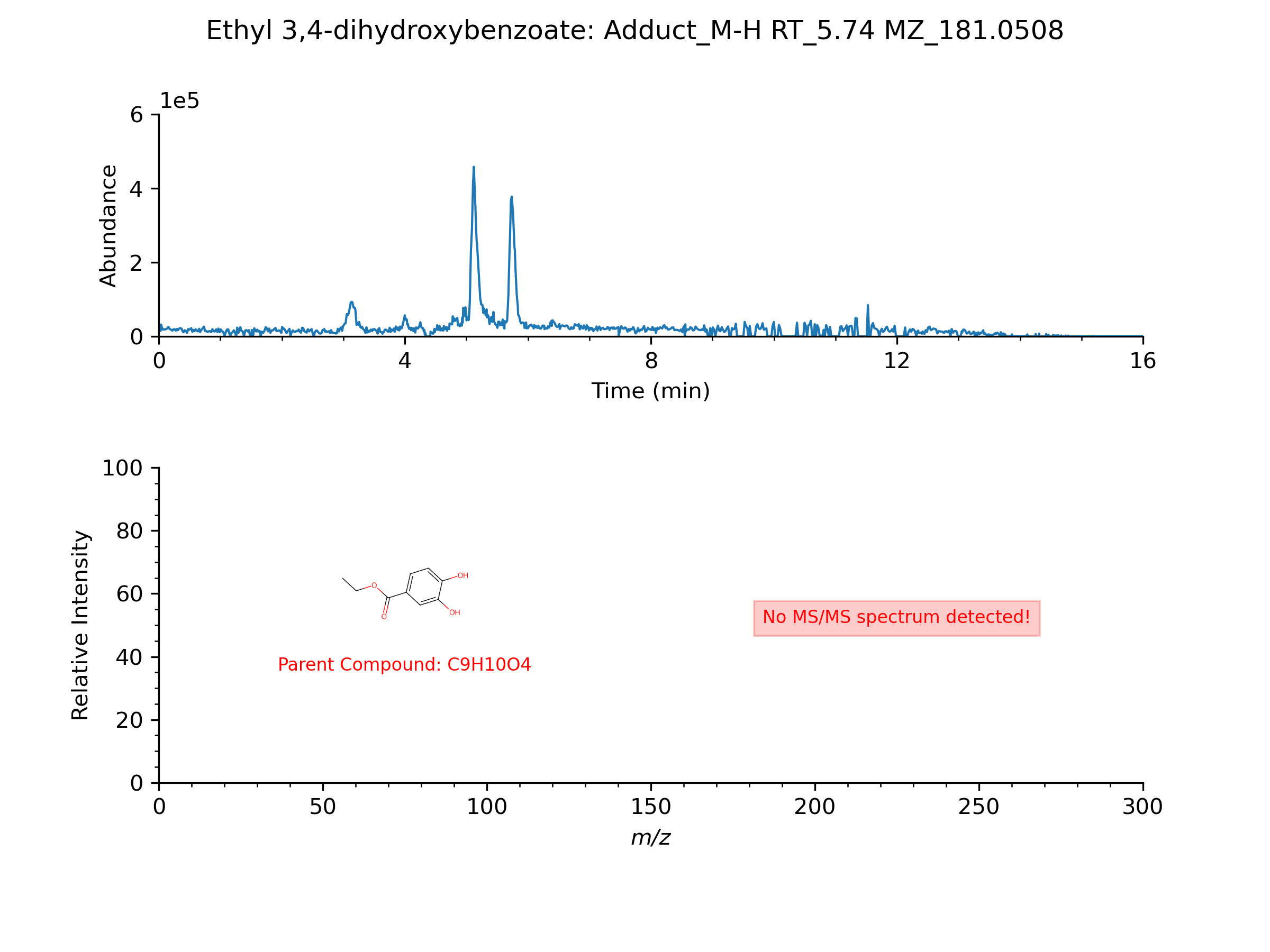

Supplement: Supplementary file 1 [file pharmaceuticals-18-01153-s001.zip › compound structures/M0140.png]

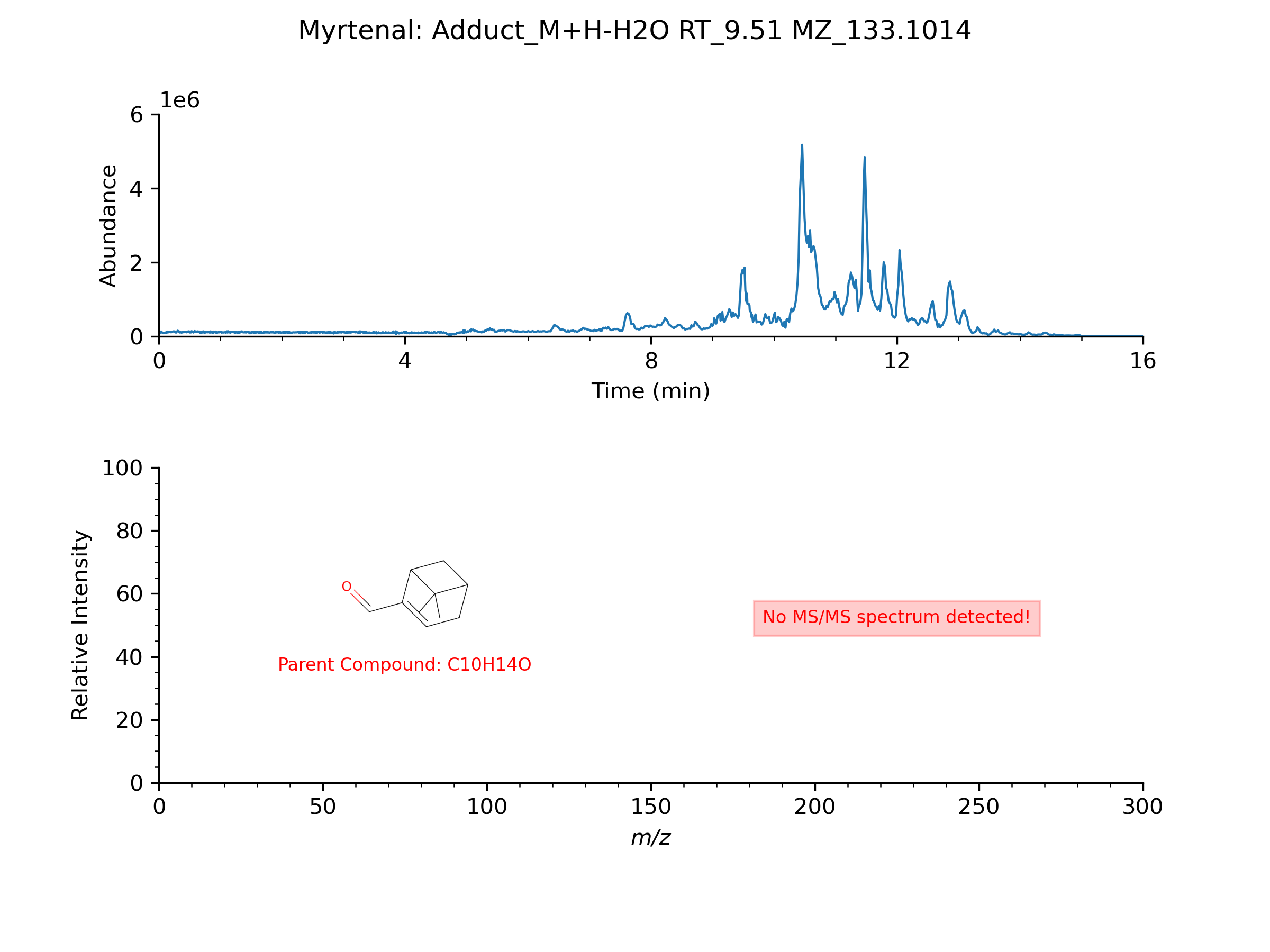

Supplement: Supplementary file 1 [file pharmaceuticals-18-01153-s001.zip › compound structures/M0141.png]

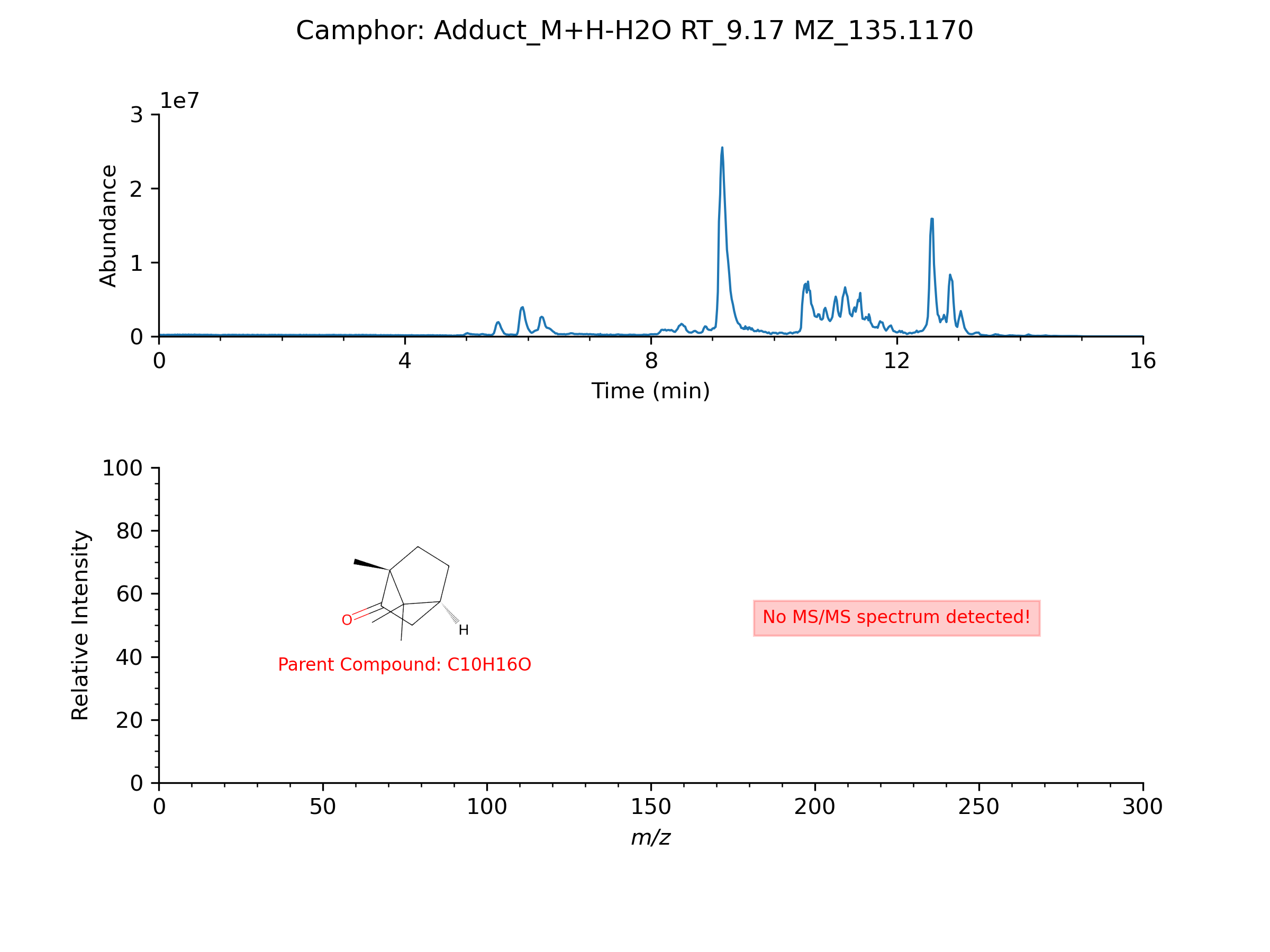

Supplement: Supplementary file 1 [file pharmaceuticals-18-01153-s001.zip › compound structures/M0142.png]

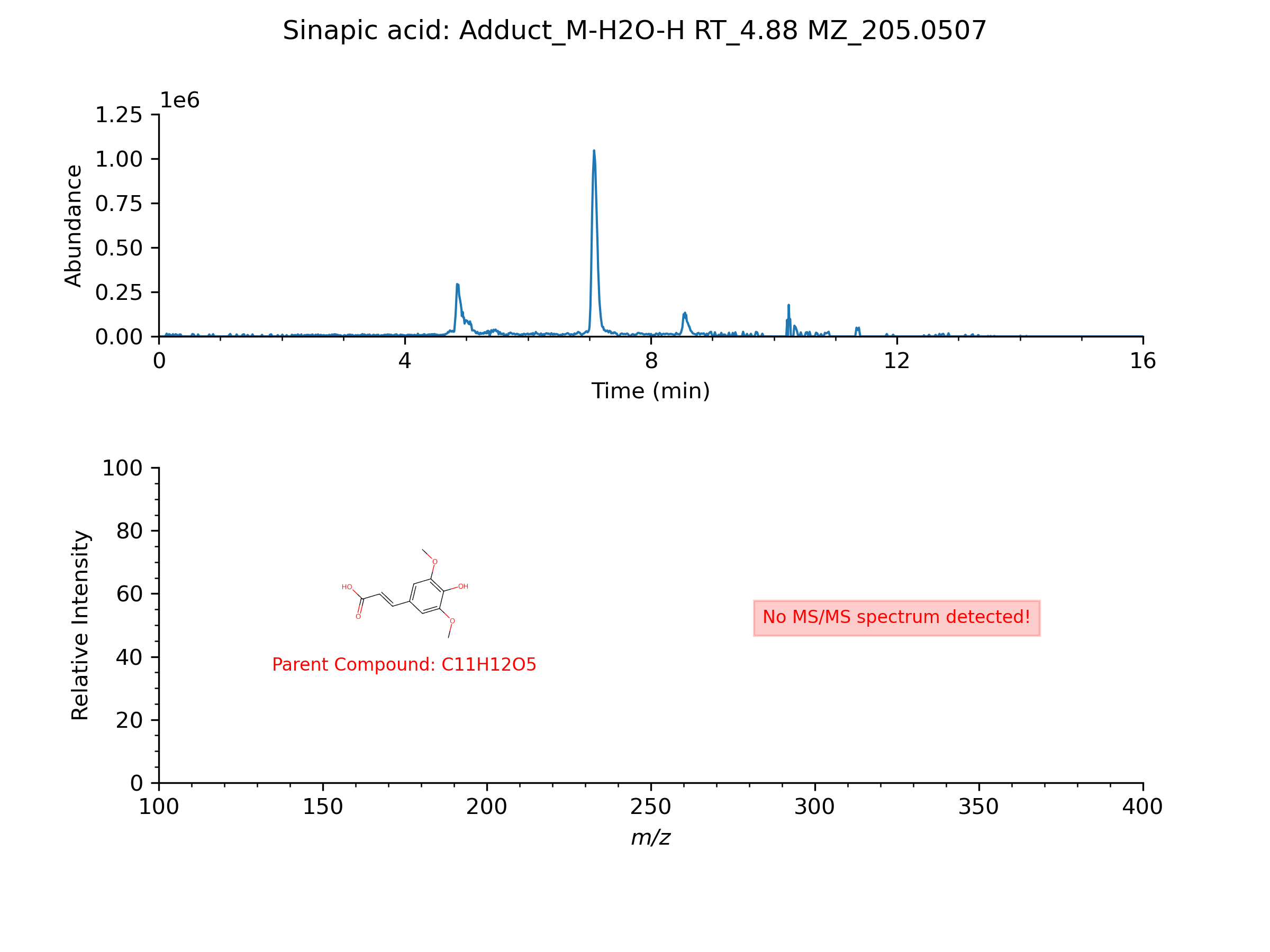

Supplement: Supplementary file 1 [file pharmaceuticals-18-01153-s001.zip › compound structures/M0143.png]

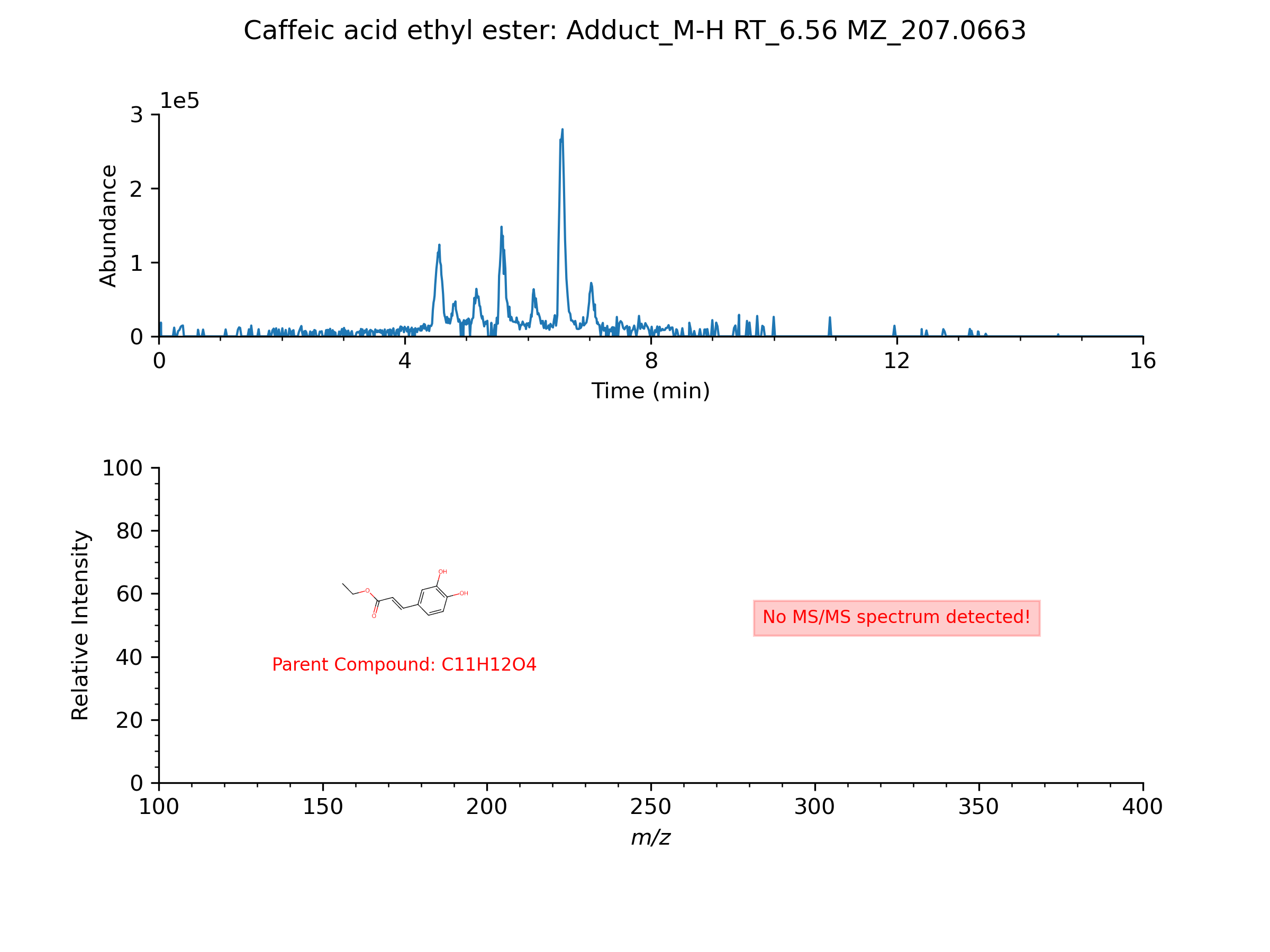

Supplement: Supplementary file 1 [file pharmaceuticals-18-01153-s001.zip › compound structures/M0144.png]

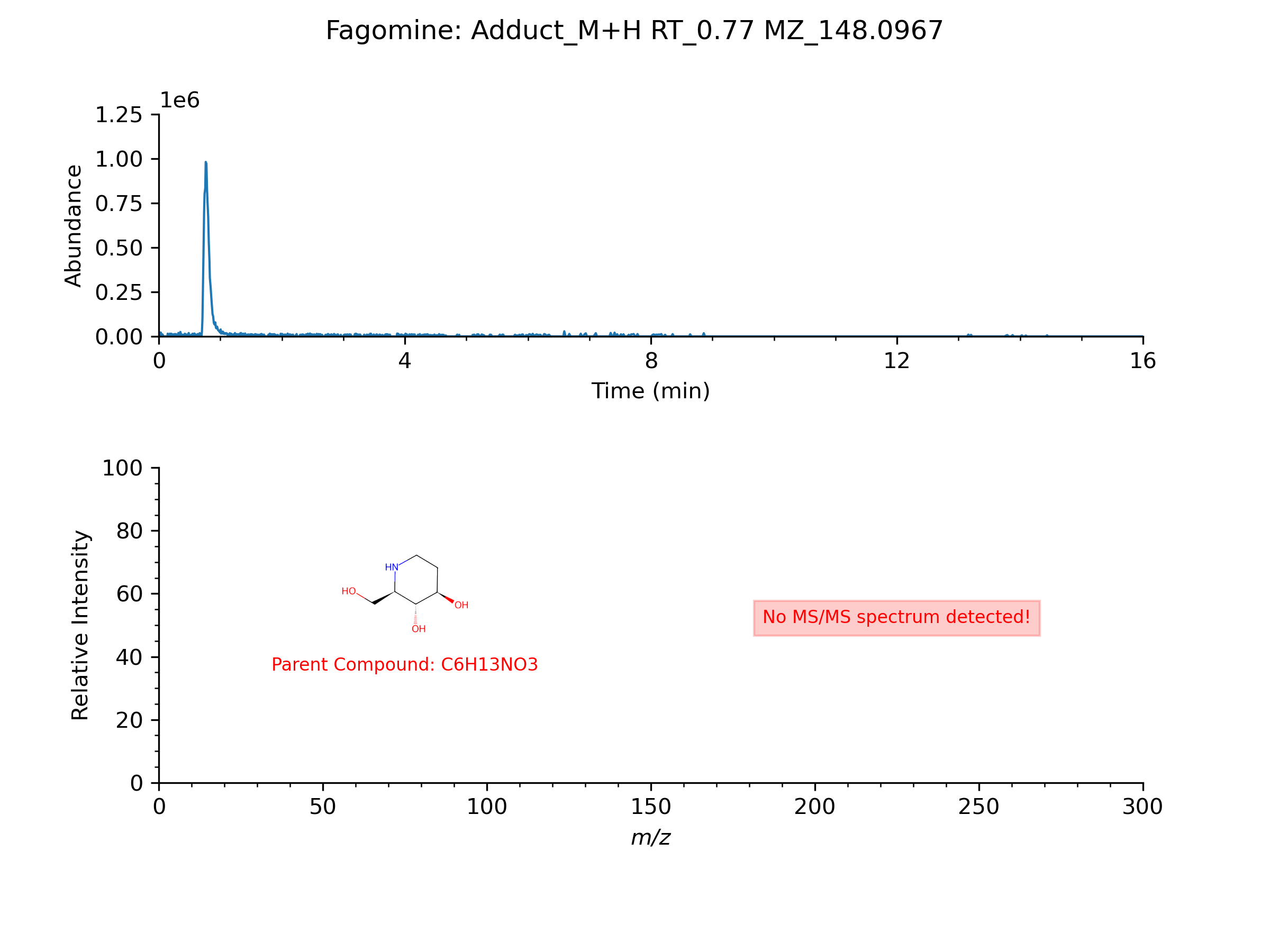

Supplement: Supplementary file 1 [file pharmaceuticals-18-01153-s001.zip › compound structures/M0145.png]

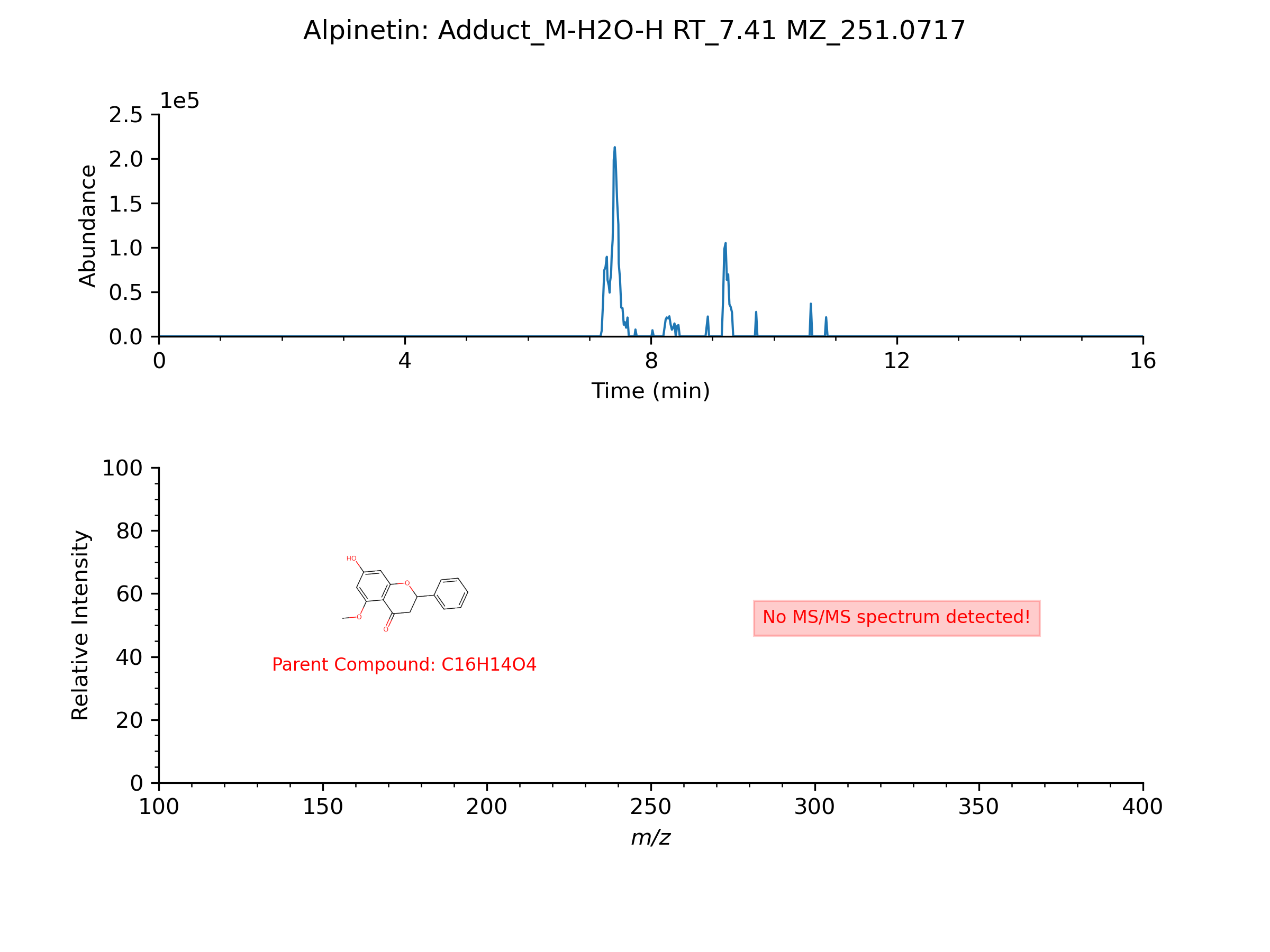

Supplement: Supplementary file 1 [file pharmaceuticals-18-01153-s001.zip › compound structures/M0146.png]

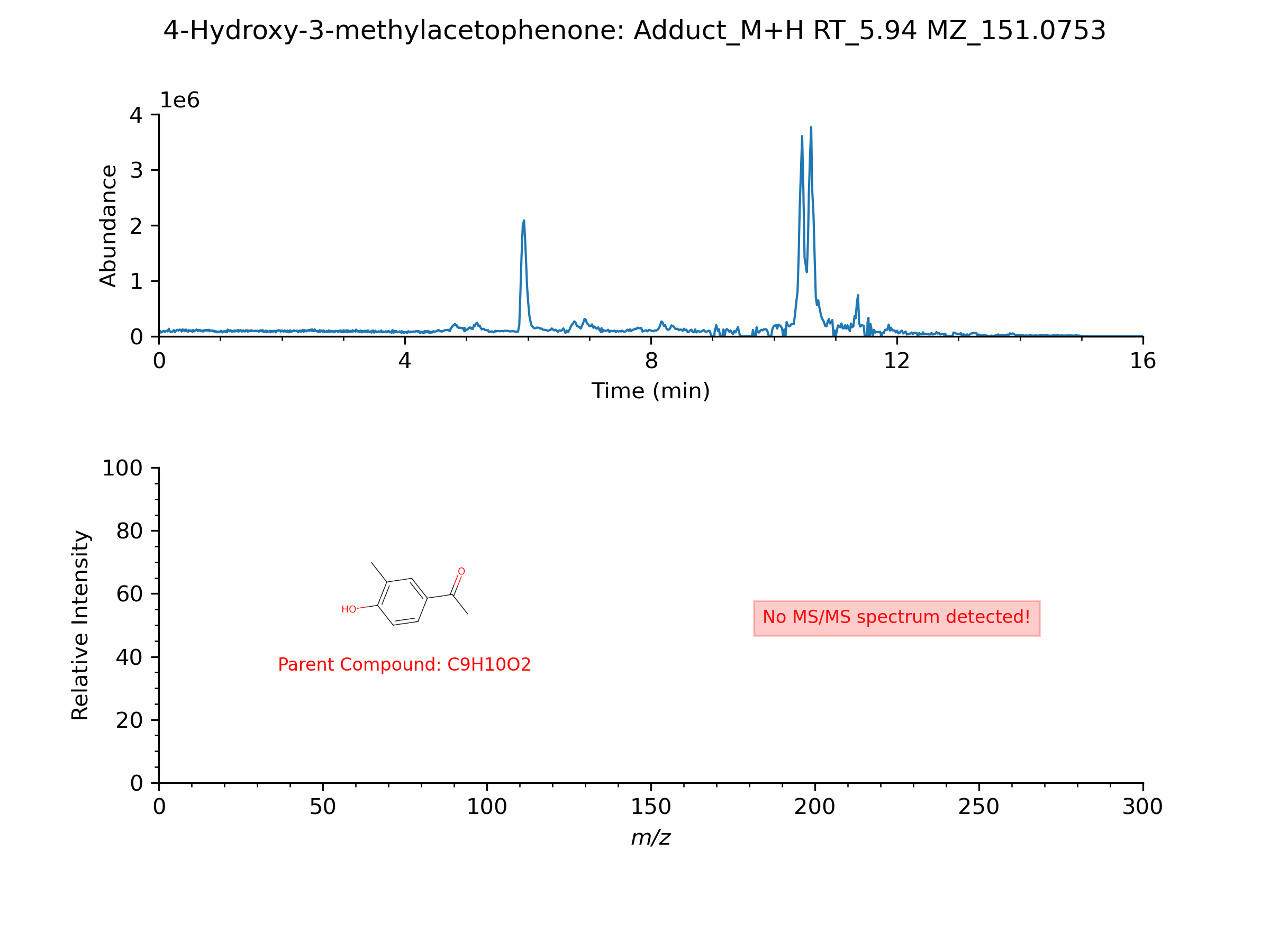

Supplement: Supplementary file 1 [file pharmaceuticals-18-01153-s001.zip › compound structures/M0147.png]

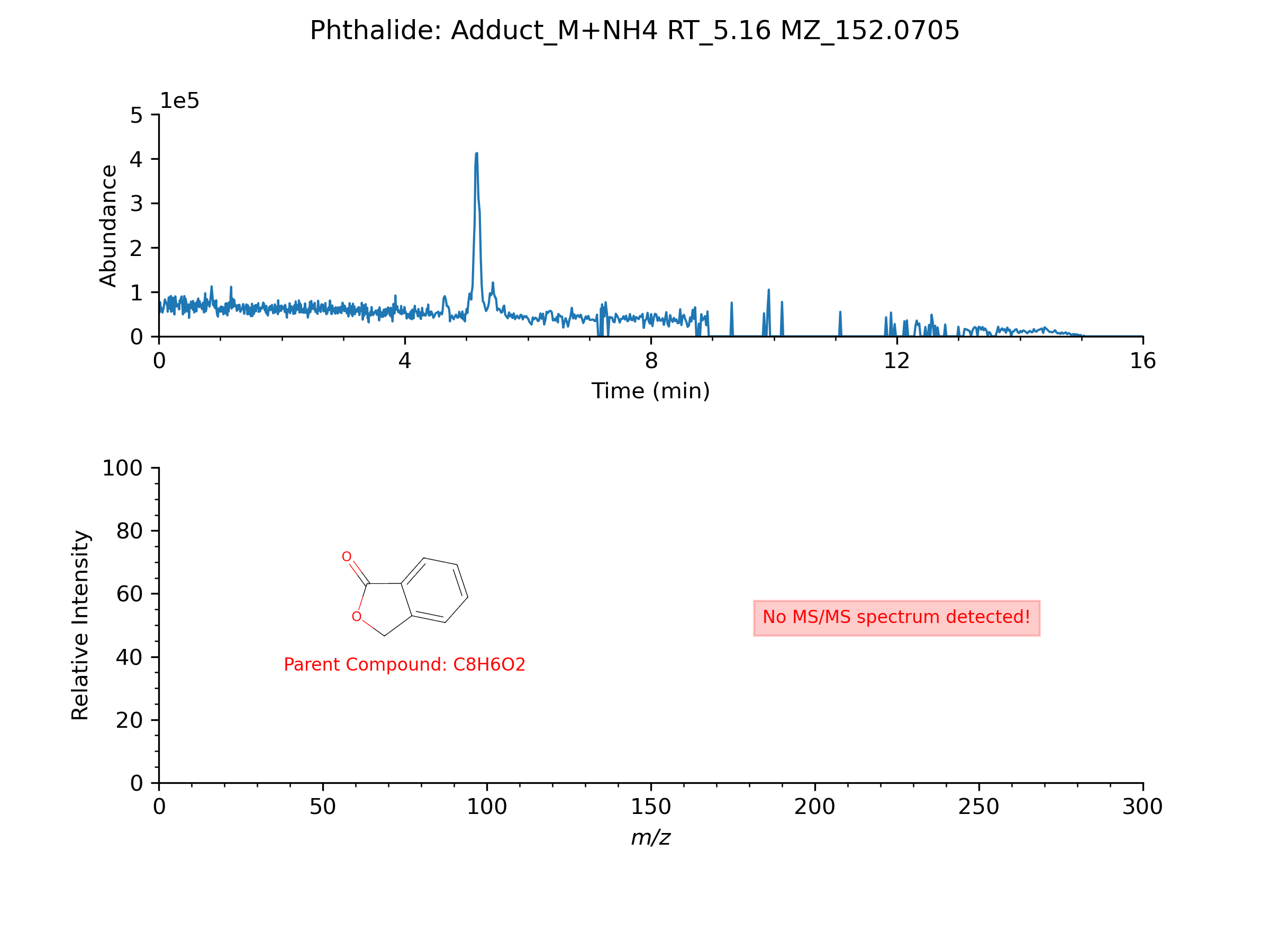

Supplement: Supplementary file 1 [file pharmaceuticals-18-01153-s001.zip › compound structures/M0148.png]

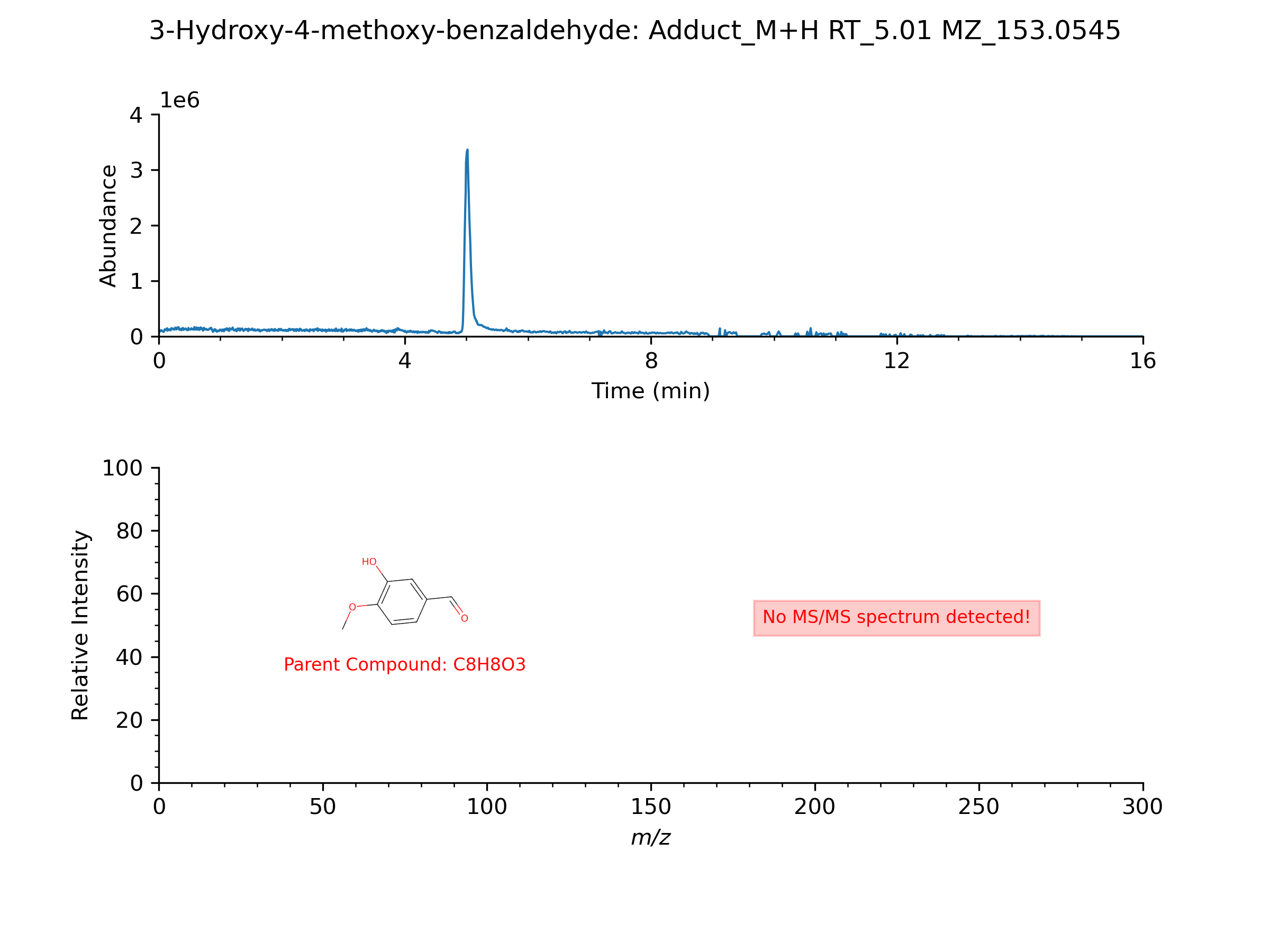

Supplement: Supplementary file 1 [file pharmaceuticals-18-01153-s001.zip › compound structures/M0149.png]

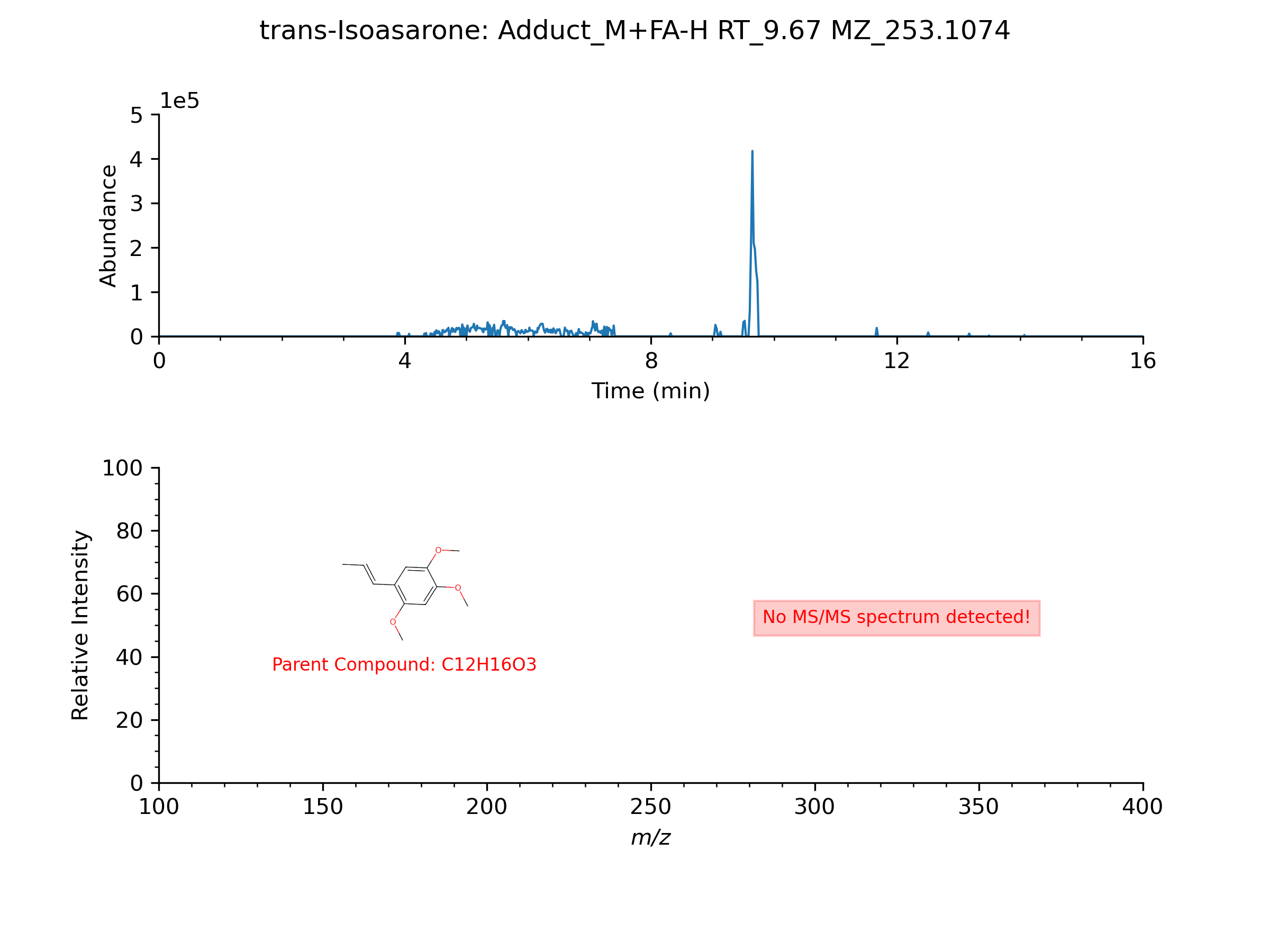

Supplement: Supplementary file 1 [file pharmaceuticals-18-01153-s001.zip › compound structures/M0150.png]

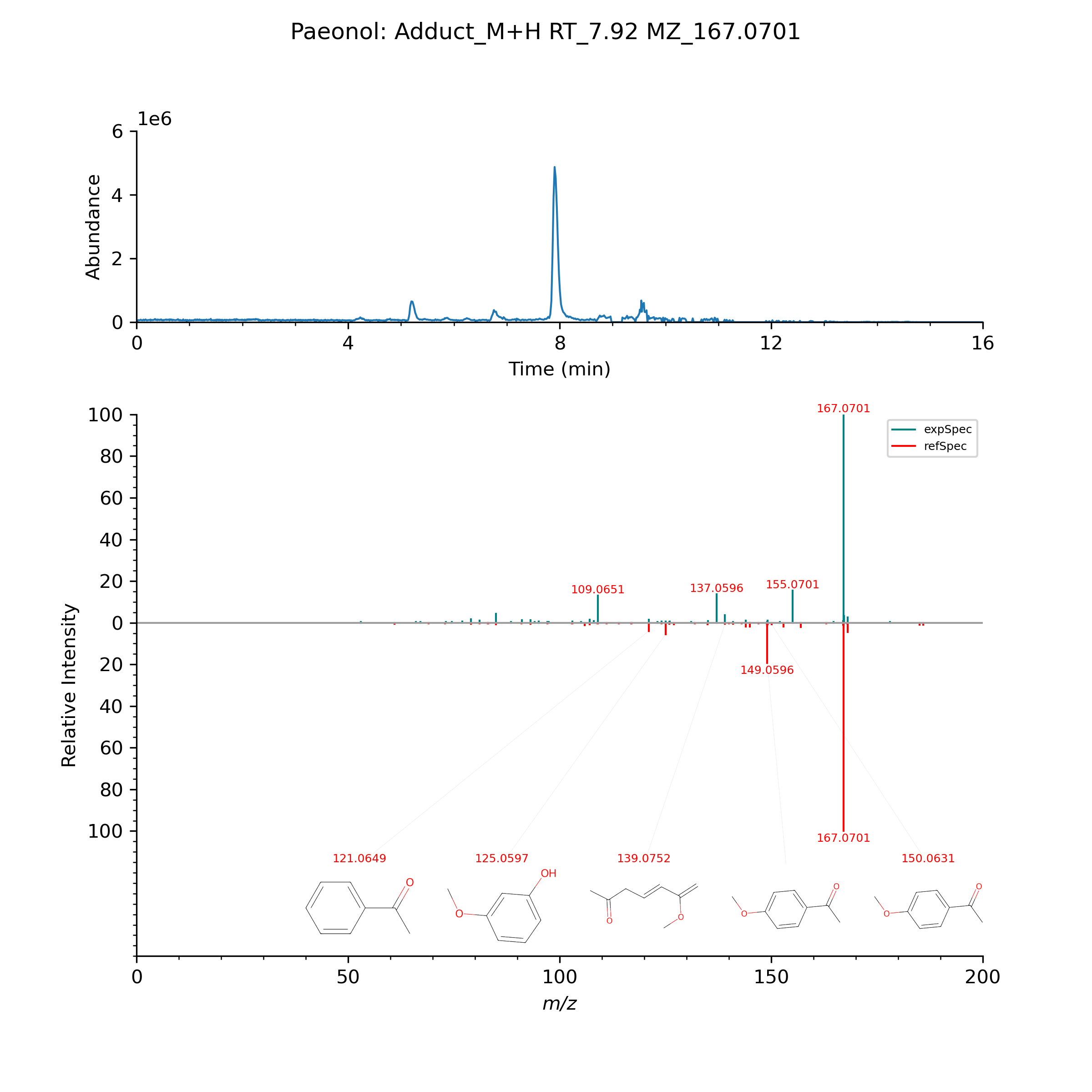

Supplement: Supplementary file 1 [file pharmaceuticals-18-01153-s001.zip › compound structures/M0151.png]

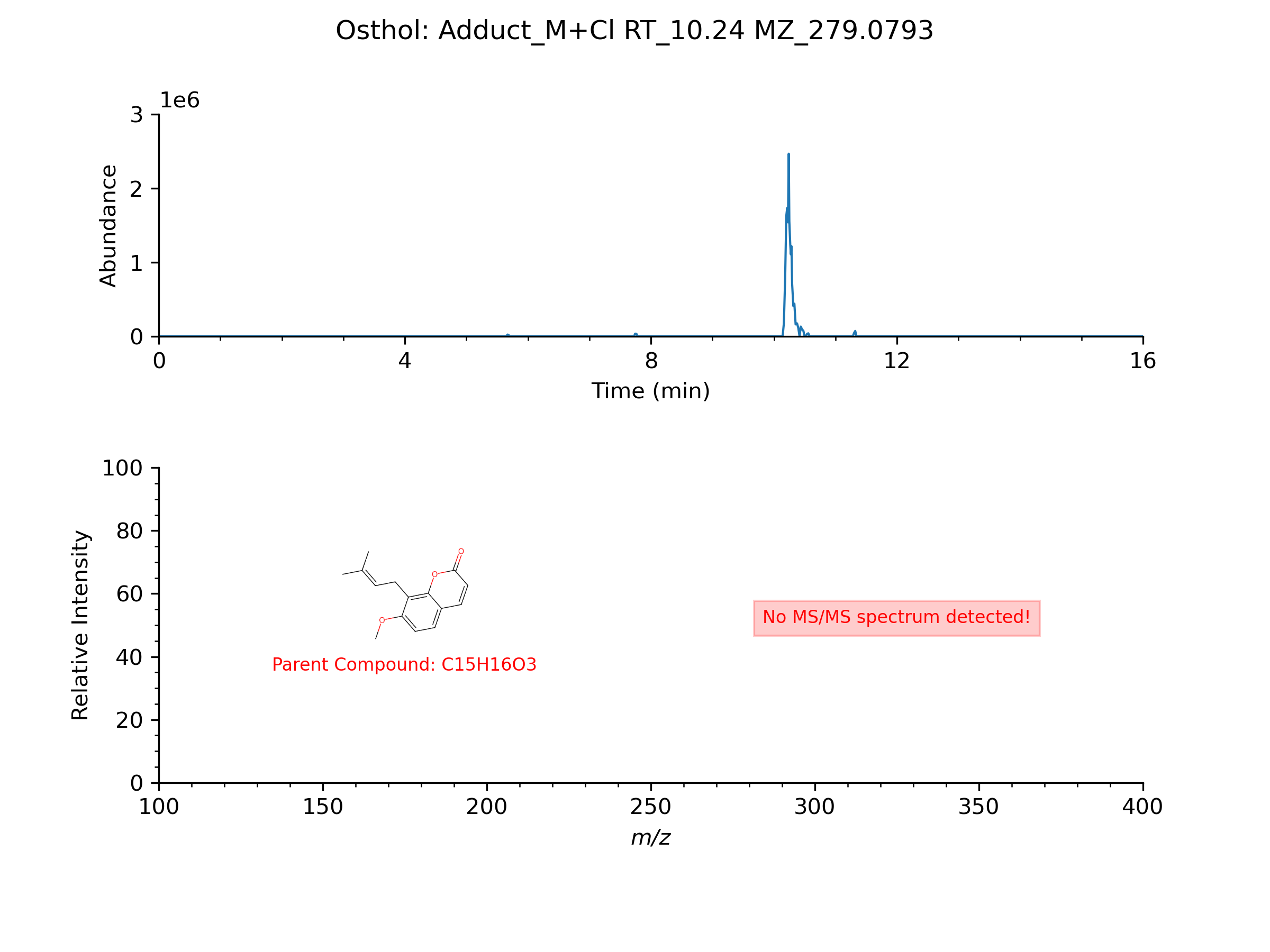

Supplement: Supplementary file 1 [file pharmaceuticals-18-01153-s001.zip › compound structures/M0152.png]

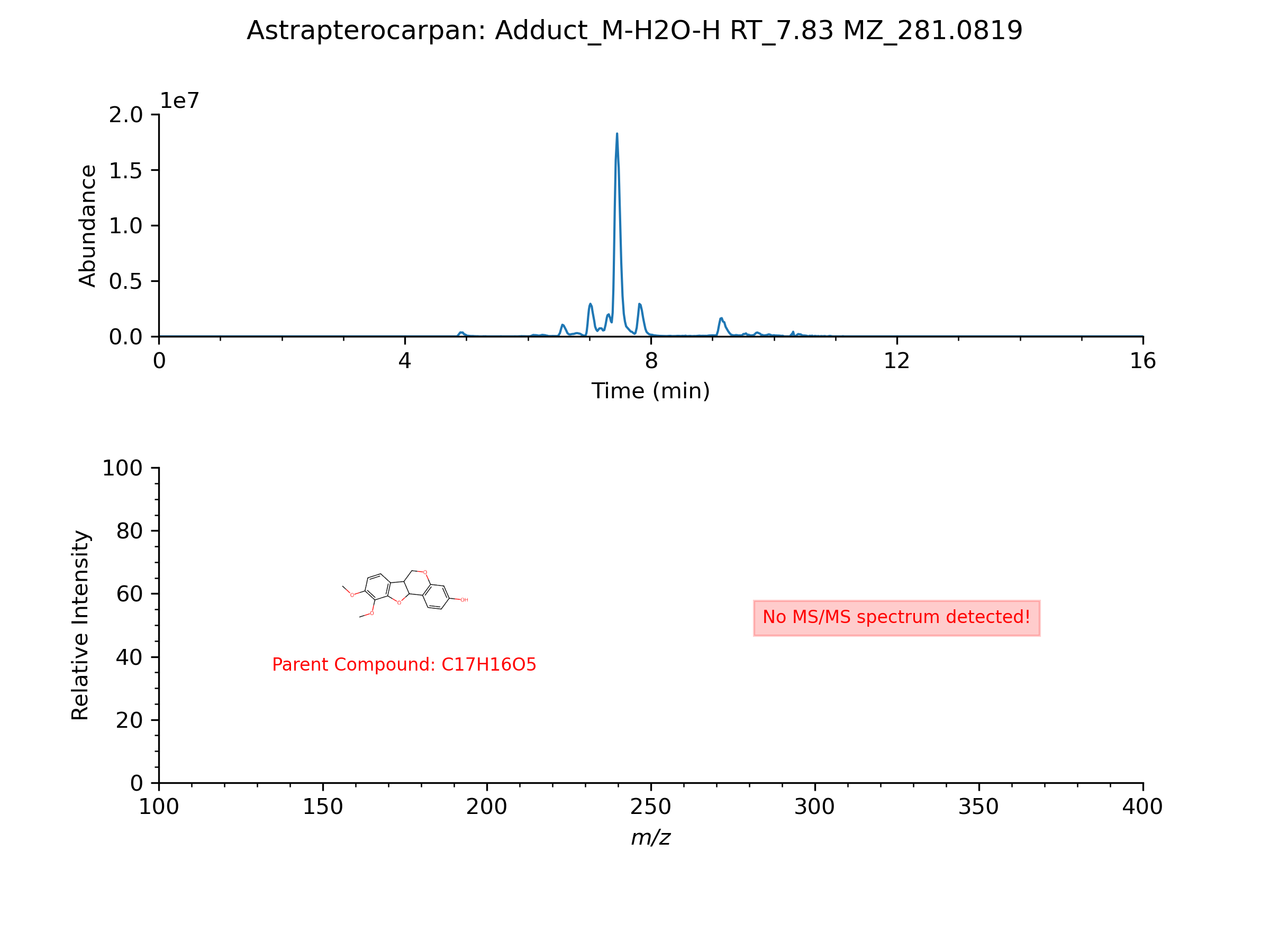

Supplement: Supplementary file 1 [file pharmaceuticals-18-01153-s001.zip › compound structures/M0153.png]

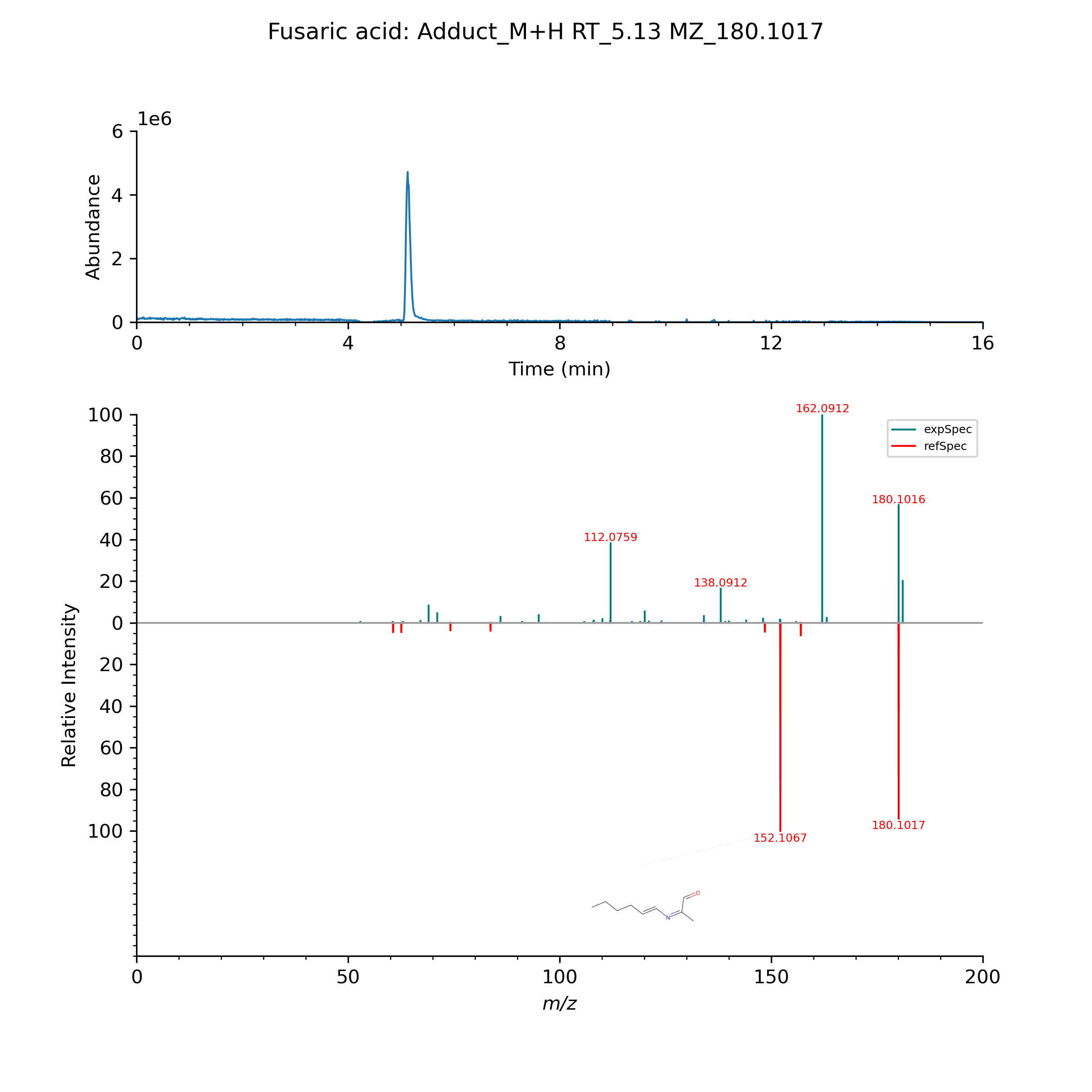

Supplement: Supplementary file 1 [file pharmaceuticals-18-01153-s001.zip › compound structures/M0154.png]

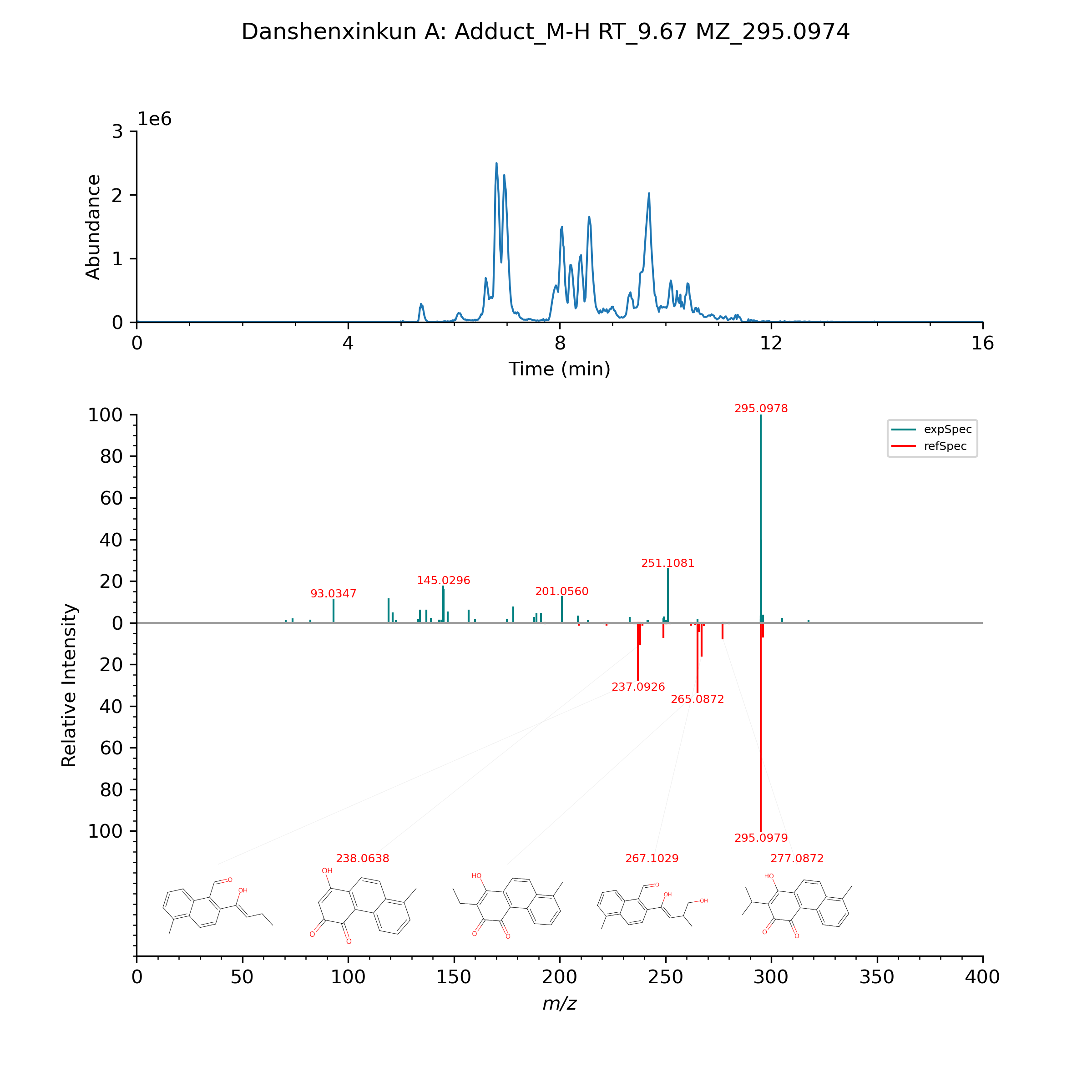

Supplement: Supplementary file 1 [file pharmaceuticals-18-01153-s001.zip › compound structures/M0155.png]

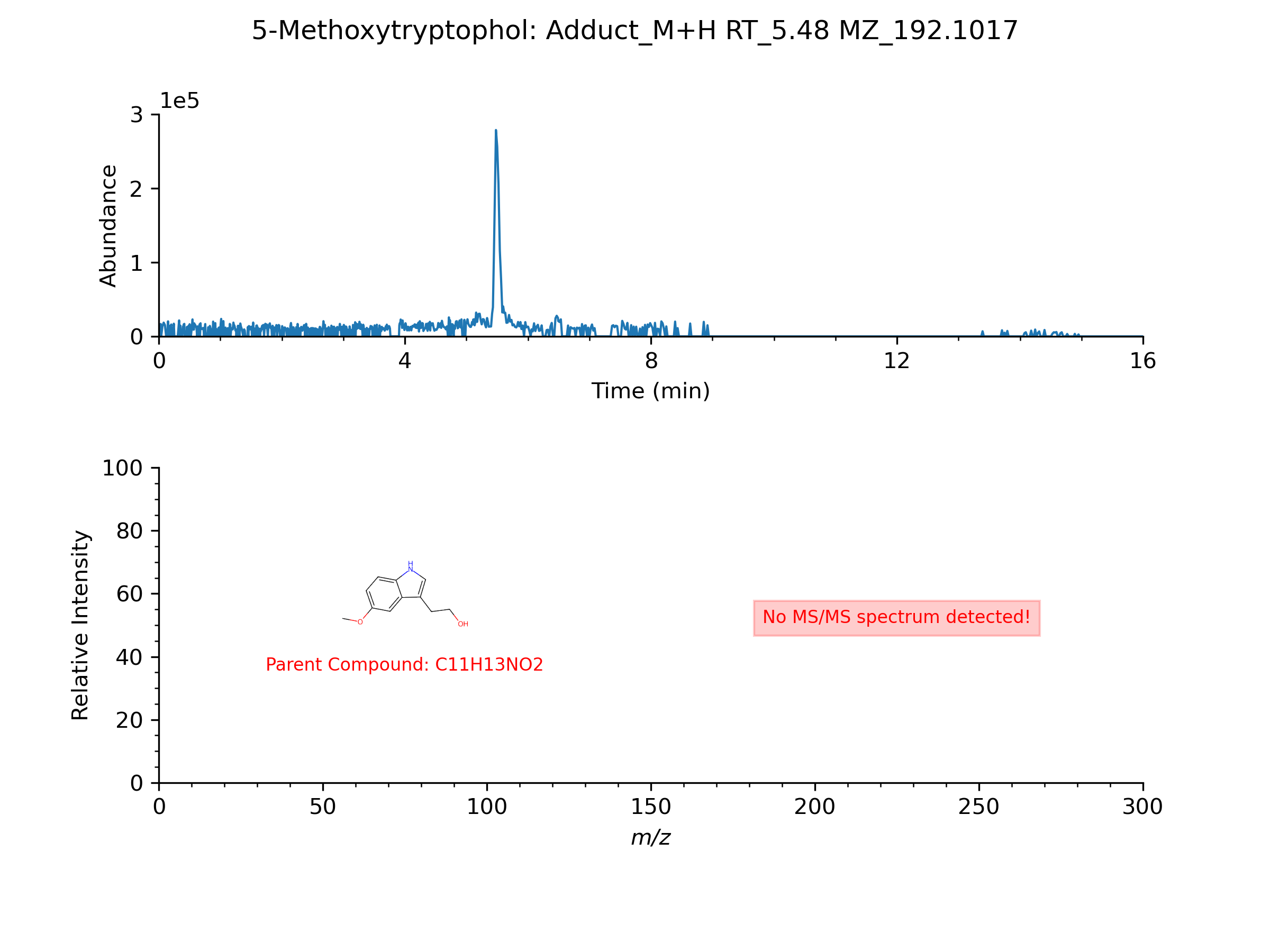

Supplement: Supplementary file 1 [file pharmaceuticals-18-01153-s001.zip › compound structures/M0156.png]

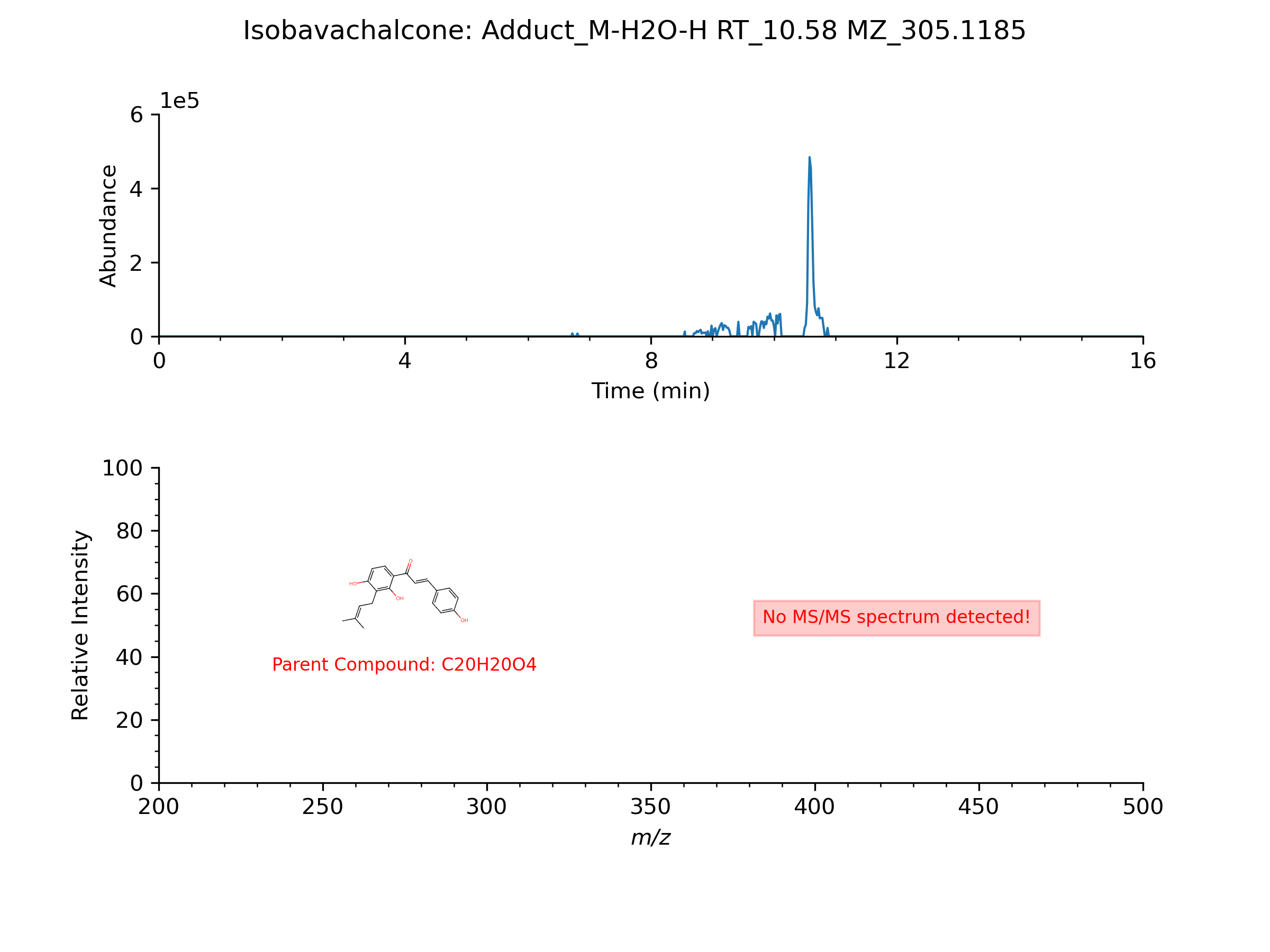

Supplement: Supplementary file 1 [file pharmaceuticals-18-01153-s001.zip › compound structures/M0157.png]

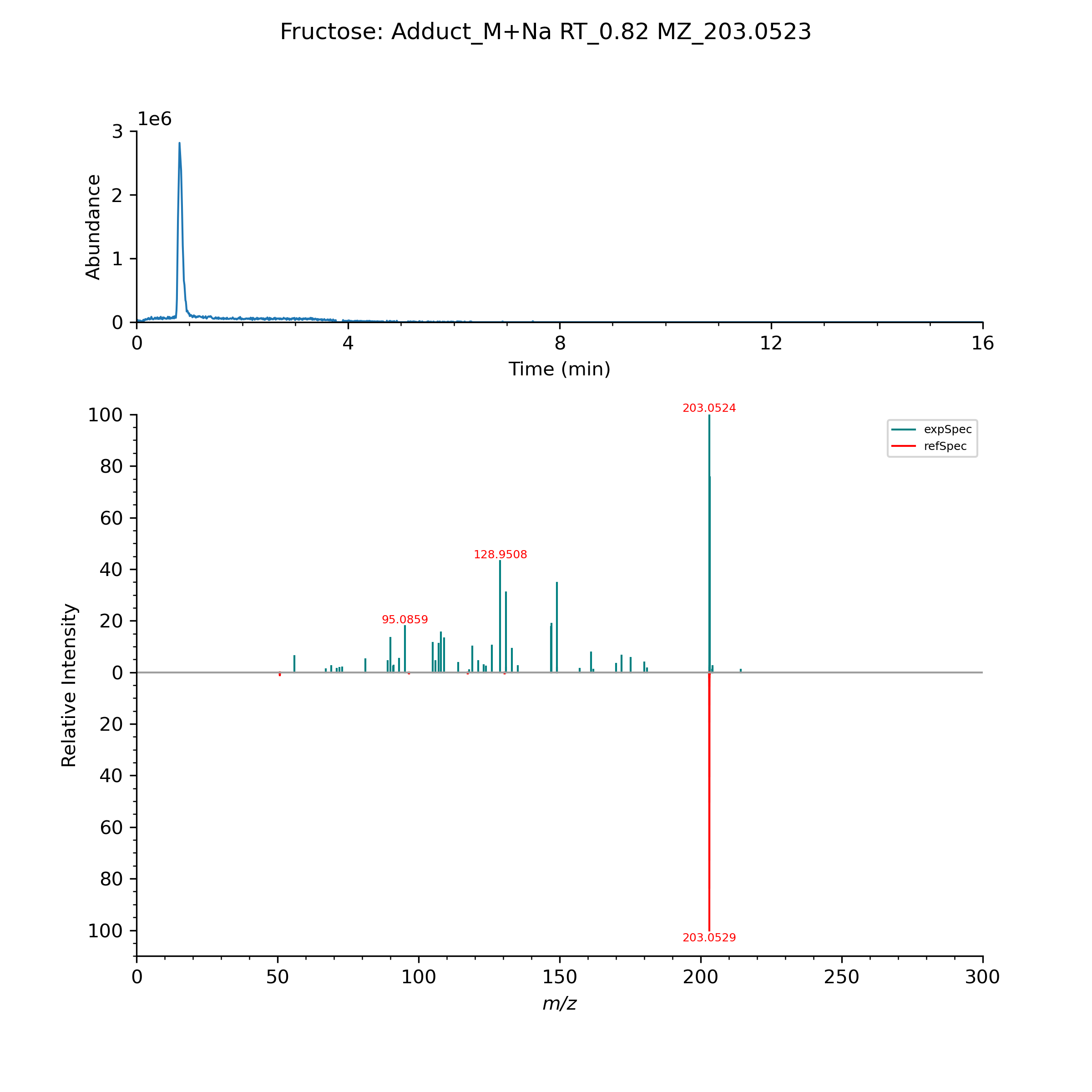

Supplement: Supplementary file 1 [file pharmaceuticals-18-01153-s001.zip › compound structures/M0158.png]

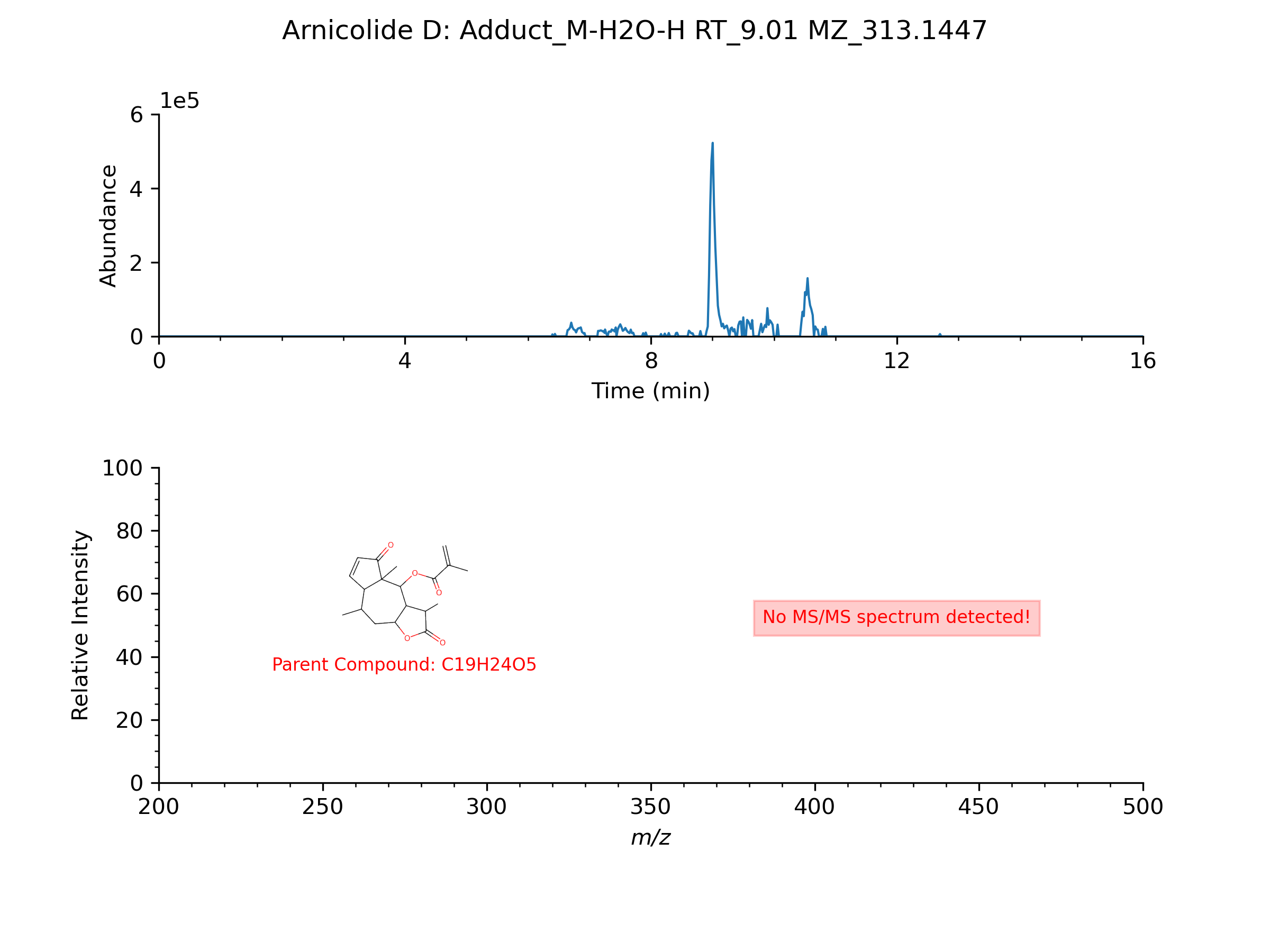

Supplement: Supplementary file 1 [file pharmaceuticals-18-01153-s001.zip › compound structures/M0159.png]

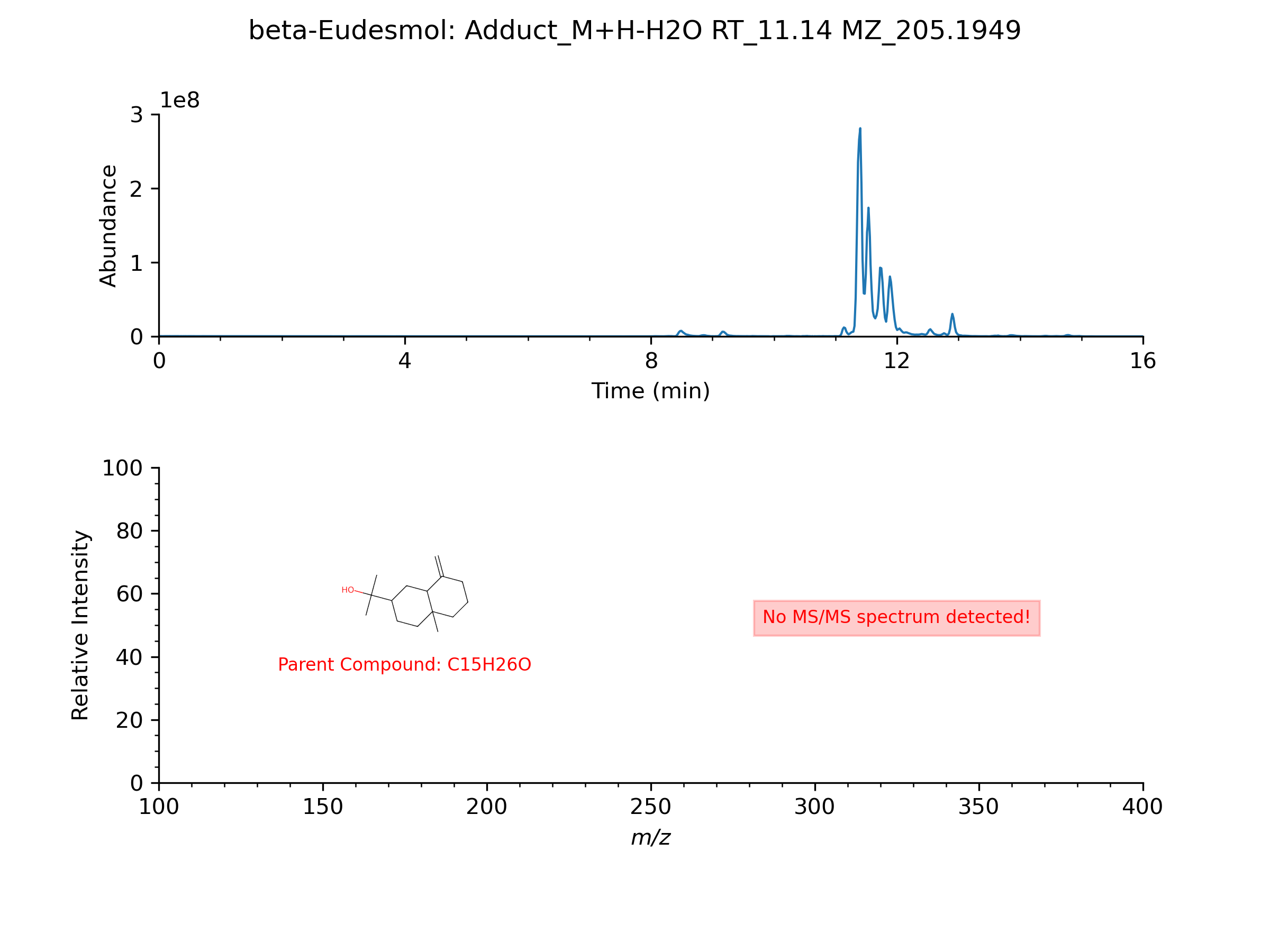

Supplement: Supplementary file 1 [file pharmaceuticals-18-01153-s001.zip › compound structures/M0160.png]

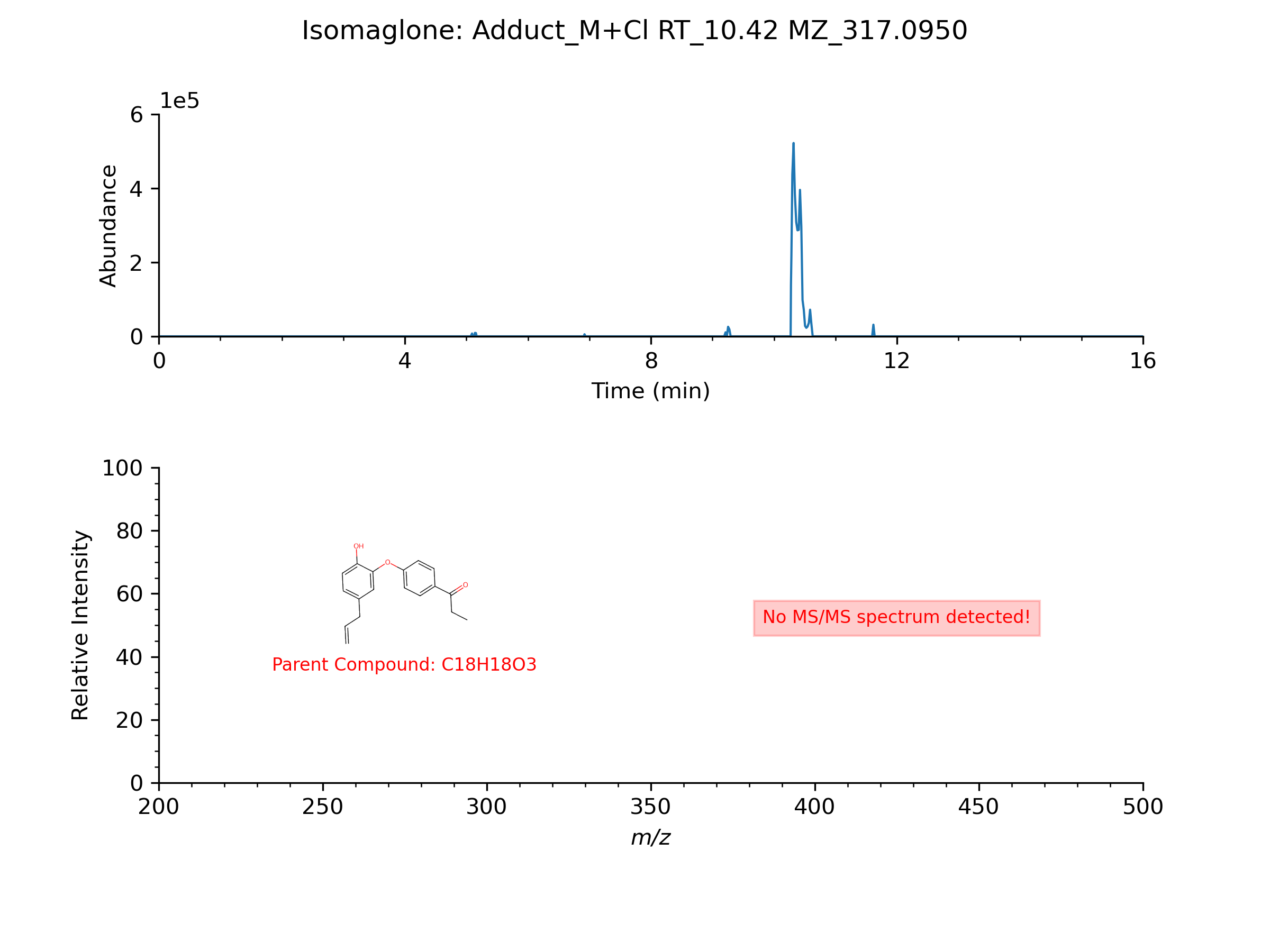

Supplement: Supplementary file 1 [file pharmaceuticals-18-01153-s001.zip › compound structures/M0161.png]

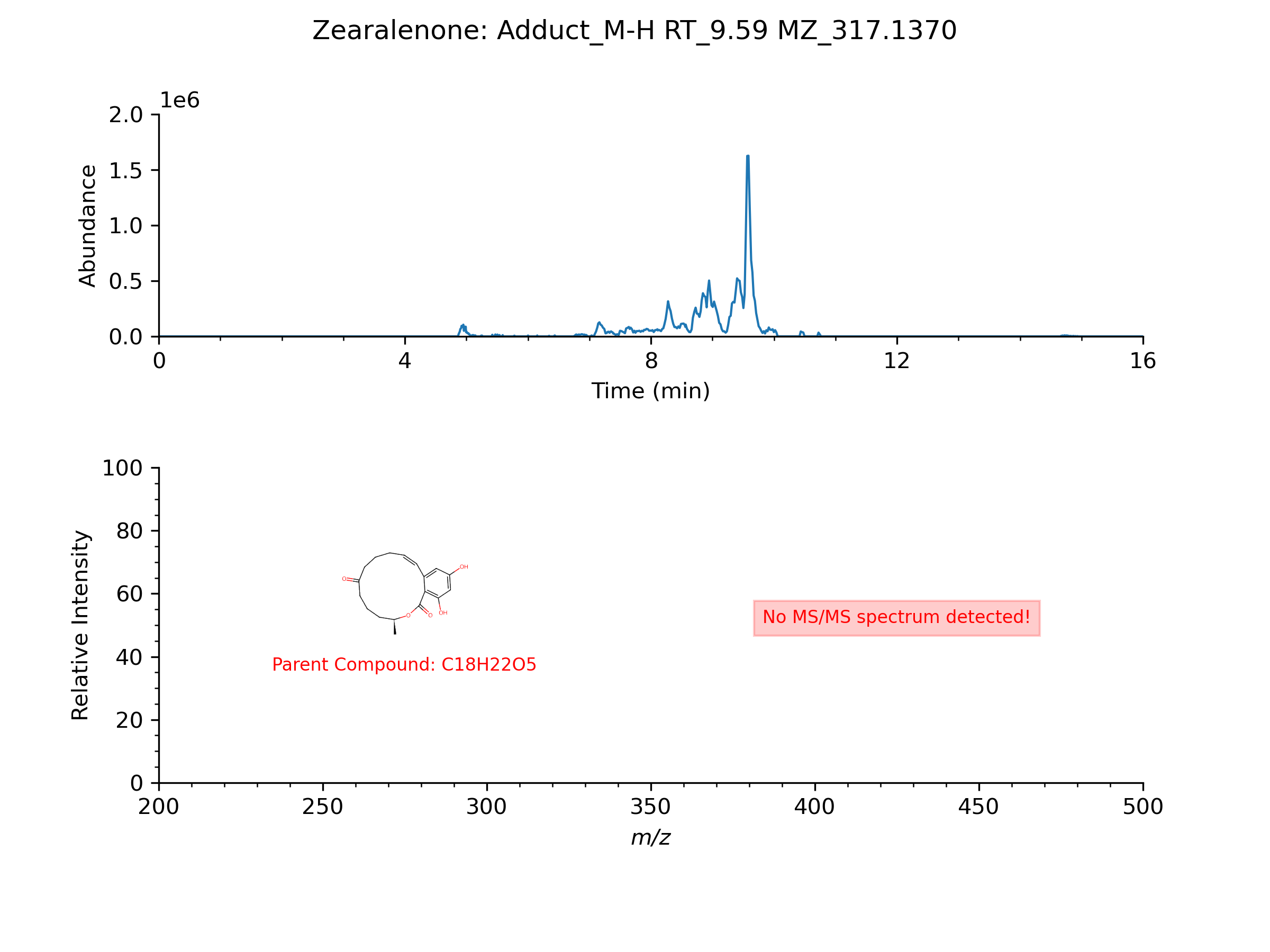

Supplement: Supplementary file 1 [file pharmaceuticals-18-01153-s001.zip › compound structures/M0162.png]

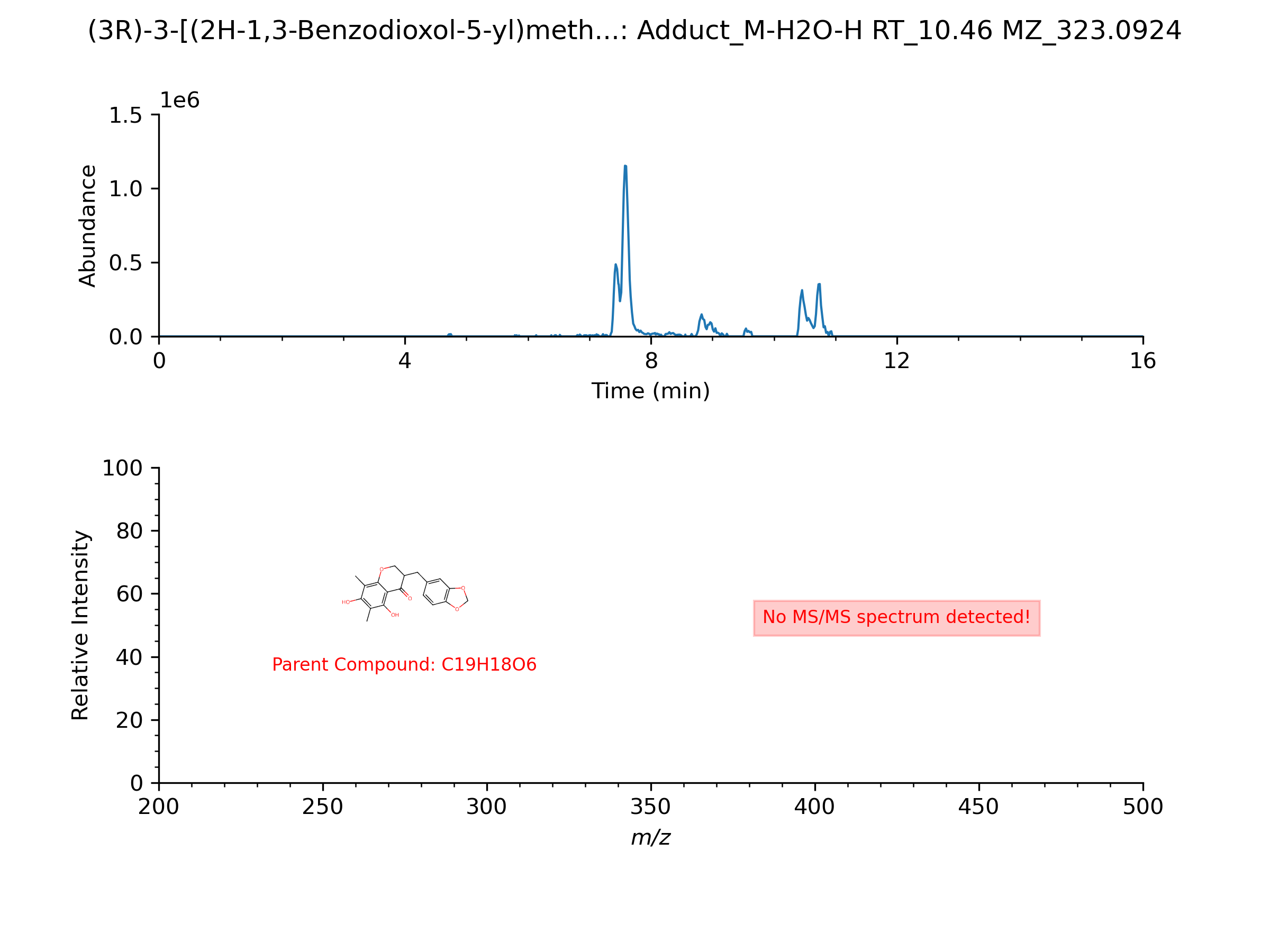

Supplement: Supplementary file 1 [file pharmaceuticals-18-01153-s001.zip › compound structures/M0163.png]

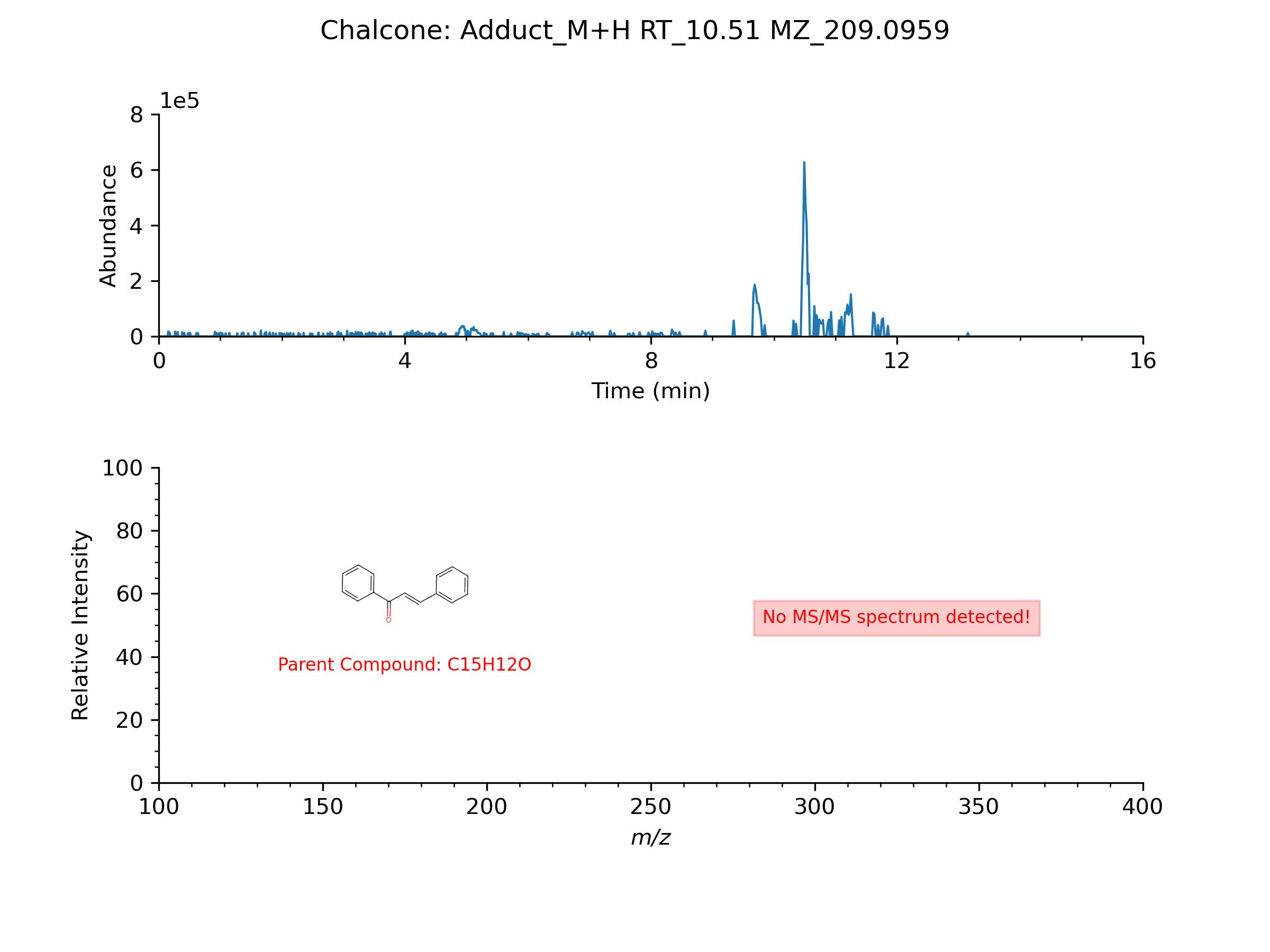

Supplement: Supplementary file 1 [file pharmaceuticals-18-01153-s001.zip › compound structures/M0164.png]

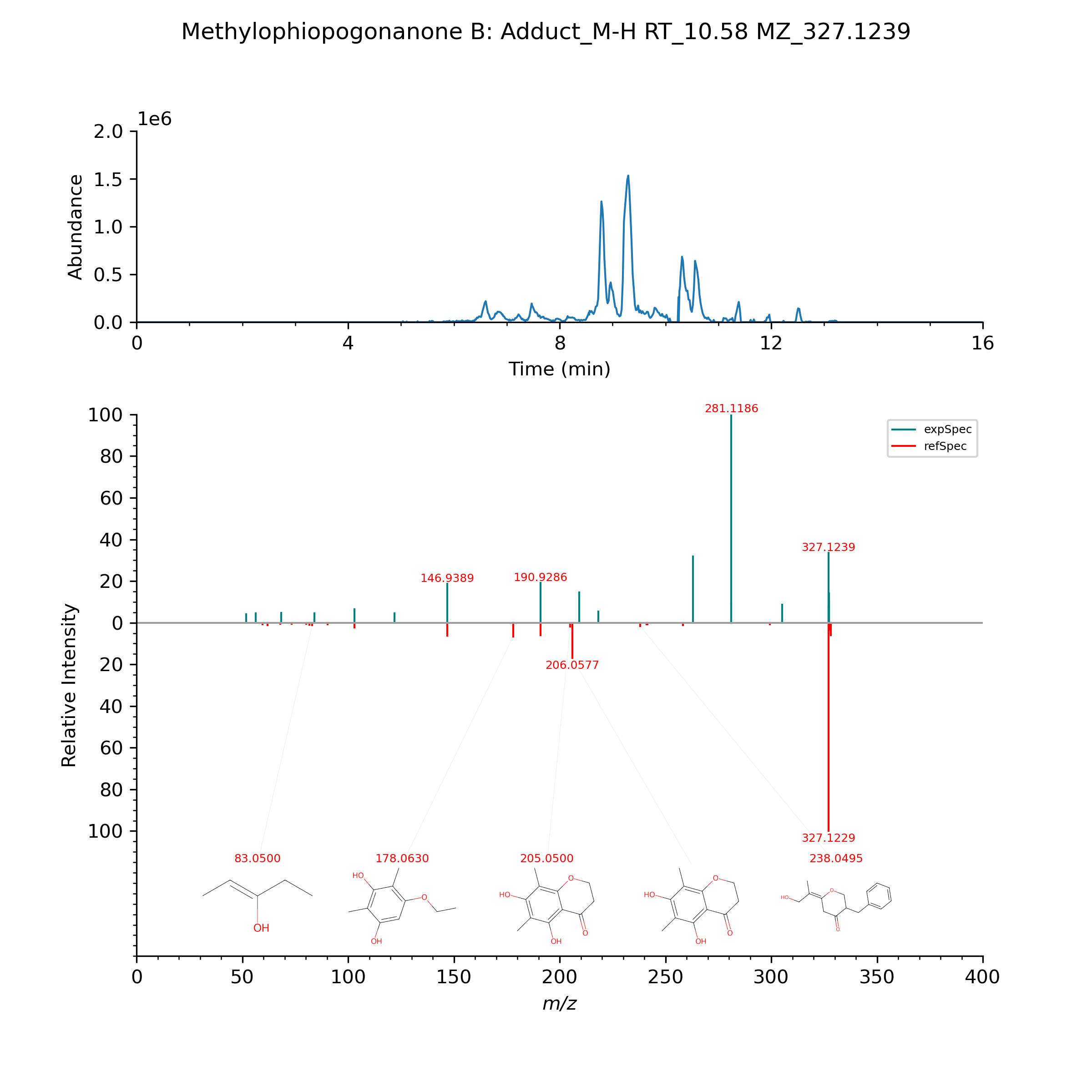

Supplement: Supplementary file 1 [file pharmaceuticals-18-01153-s001.zip › compound structures/M0165.png]

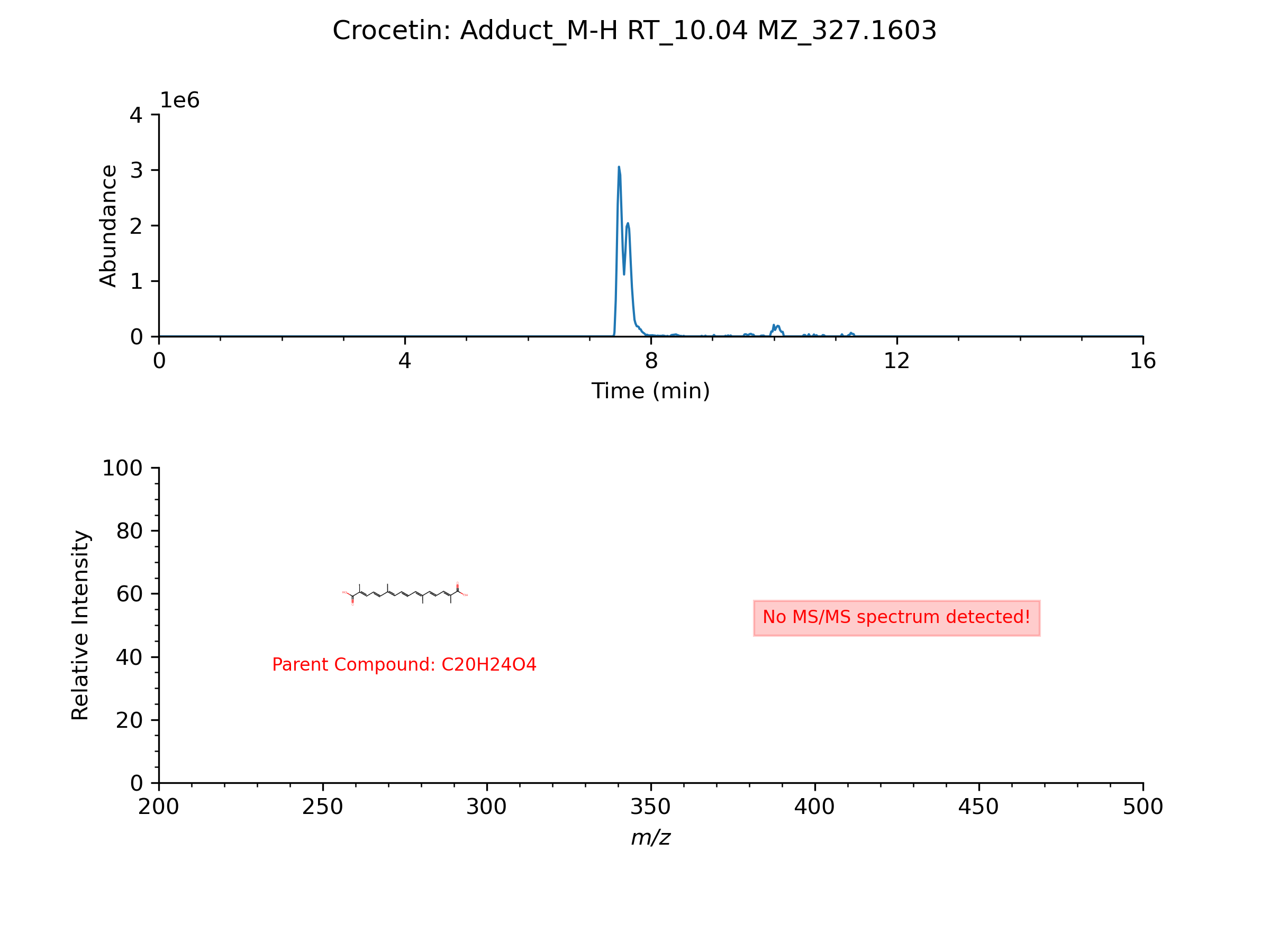

Supplement: Supplementary file 1 [file pharmaceuticals-18-01153-s001.zip › compound structures/M0166.png]

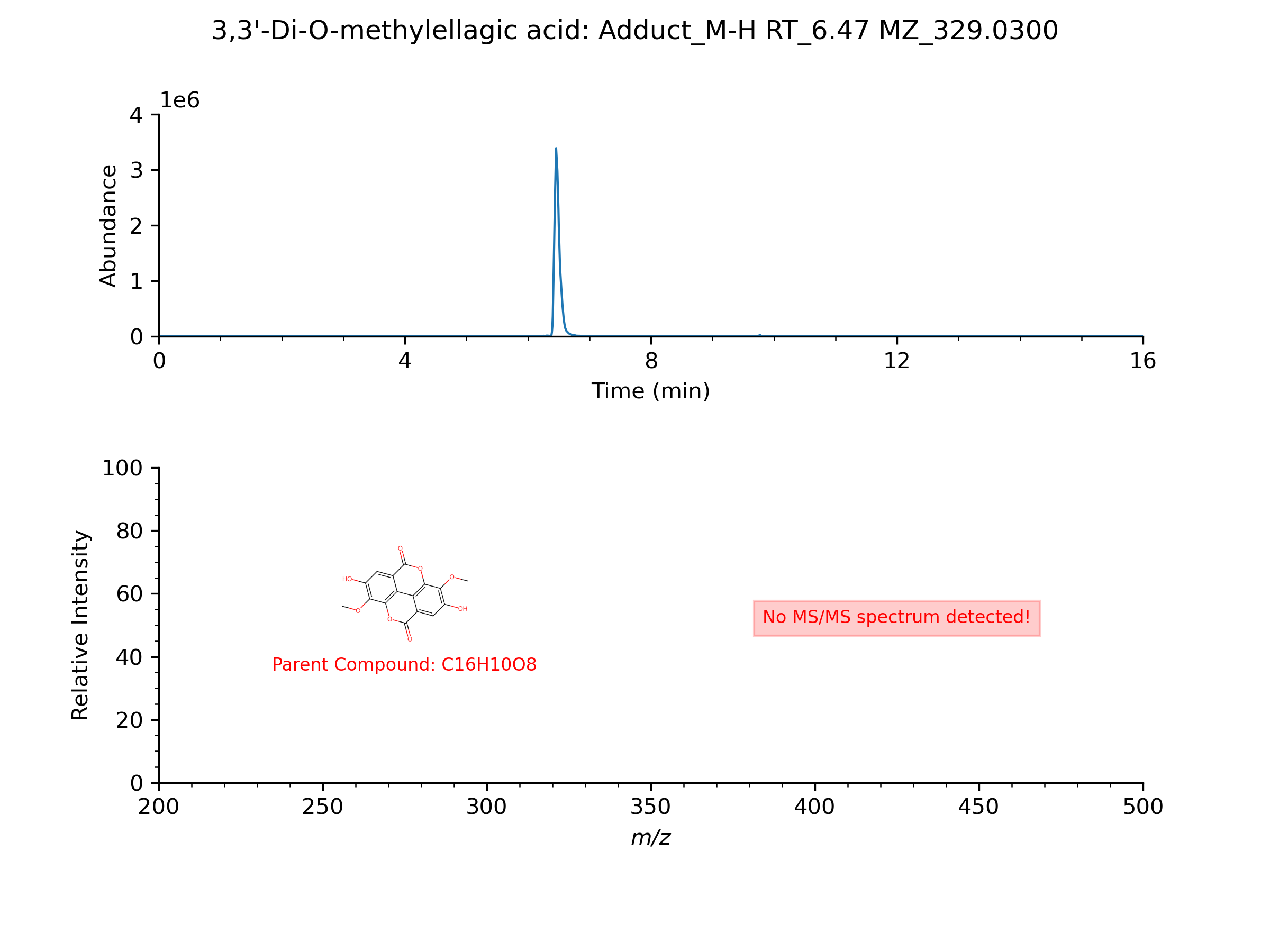

Supplement: Supplementary file 1 [file pharmaceuticals-18-01153-s001.zip › compound structures/M0167.png]

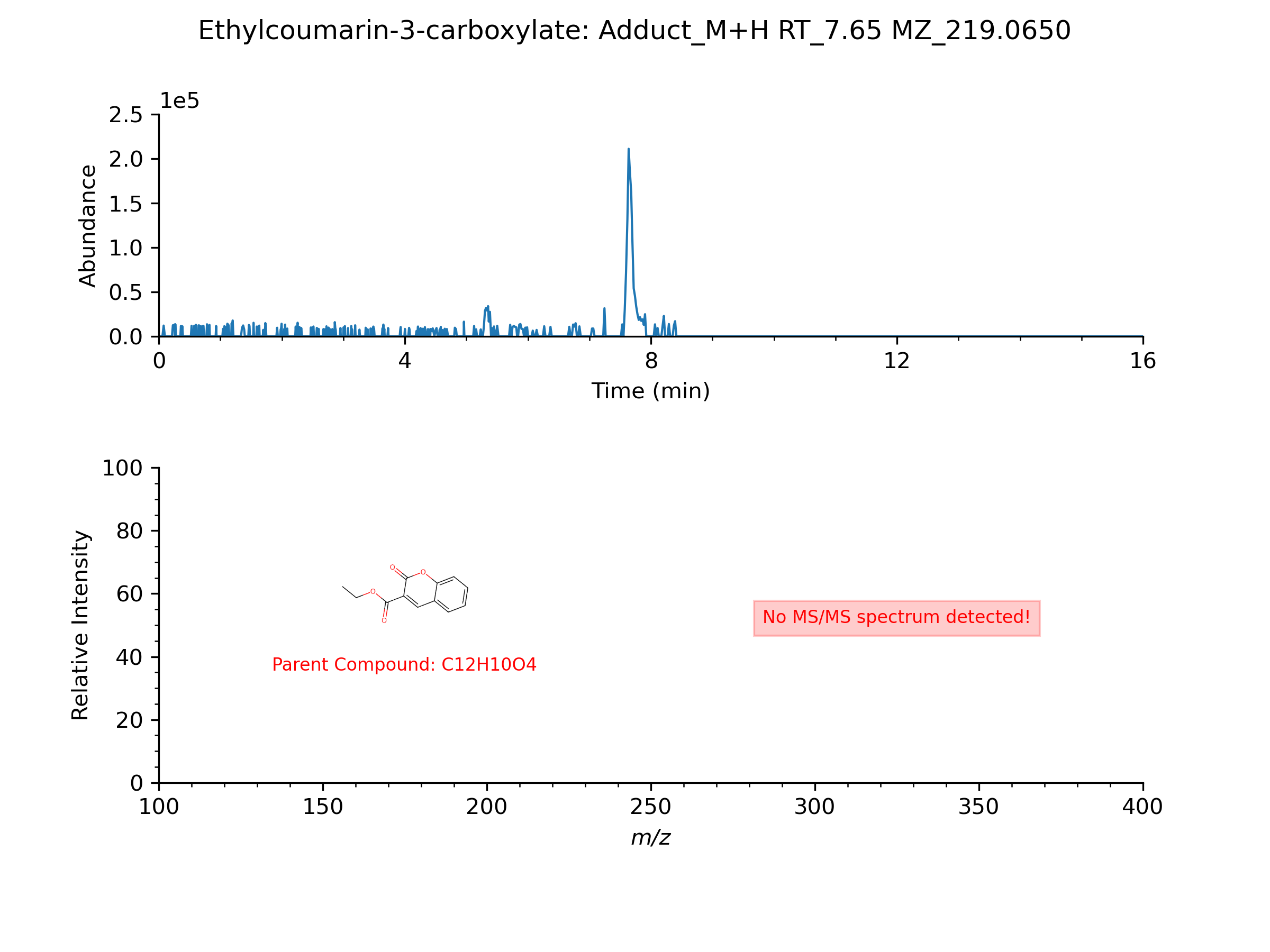

Supplement: Supplementary file 1 [file pharmaceuticals-18-01153-s001.zip › compound structures/M0168.png]

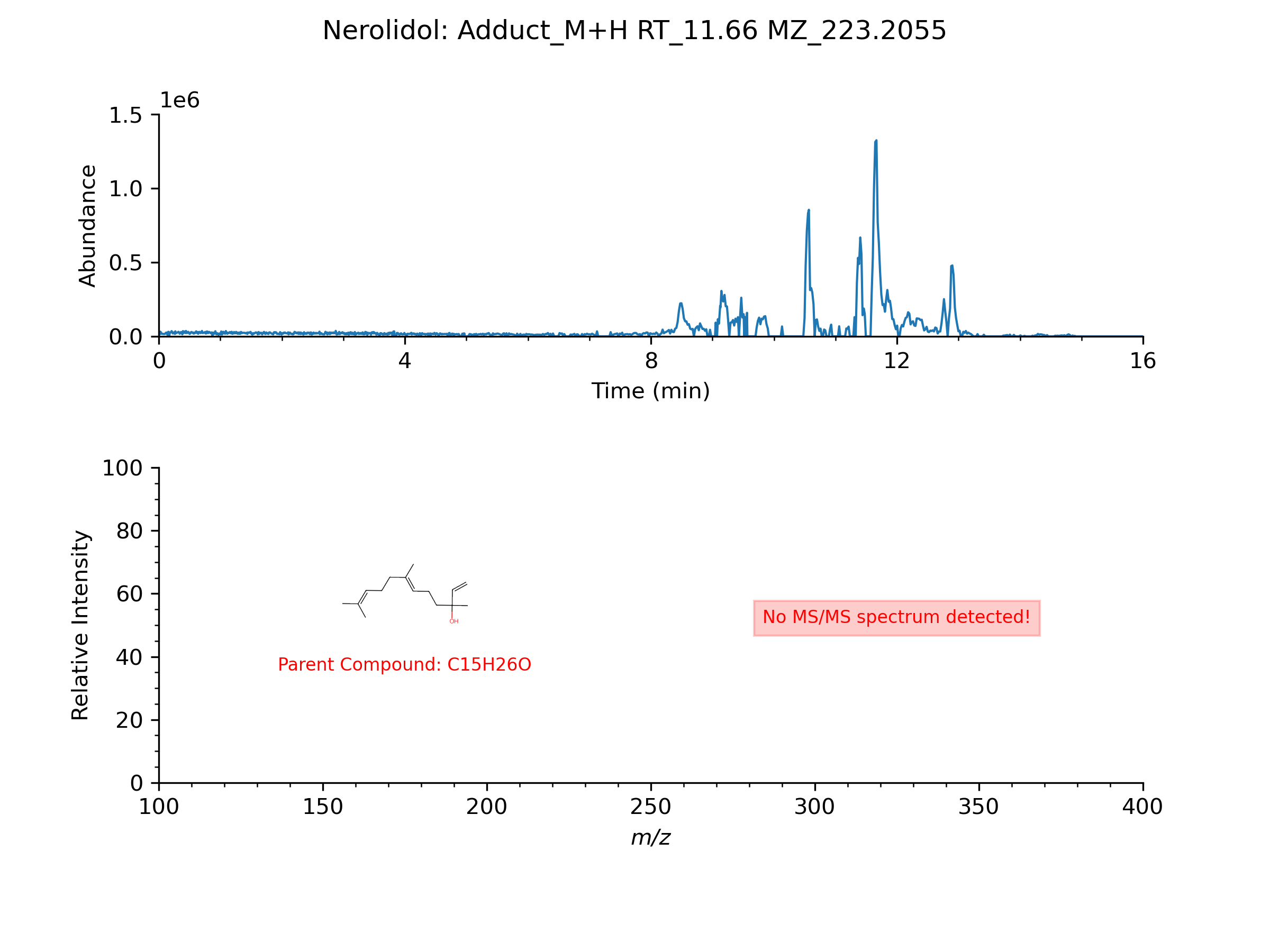

Supplement: Supplementary file 1 [file pharmaceuticals-18-01153-s001.zip › compound structures/M0169.png]

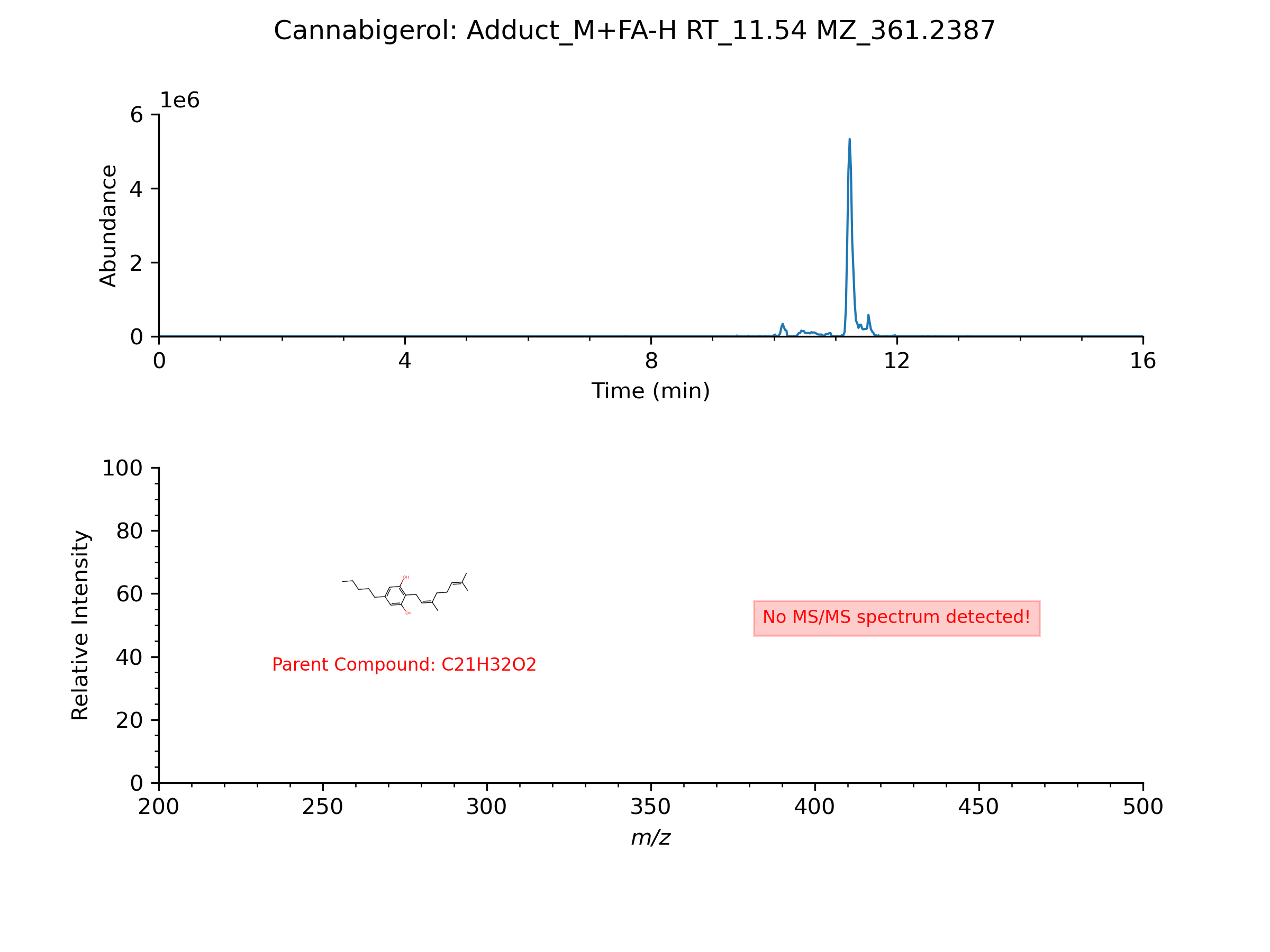

Supplement: Supplementary file 1 [file pharmaceuticals-18-01153-s001.zip › compound structures/M0170.png]

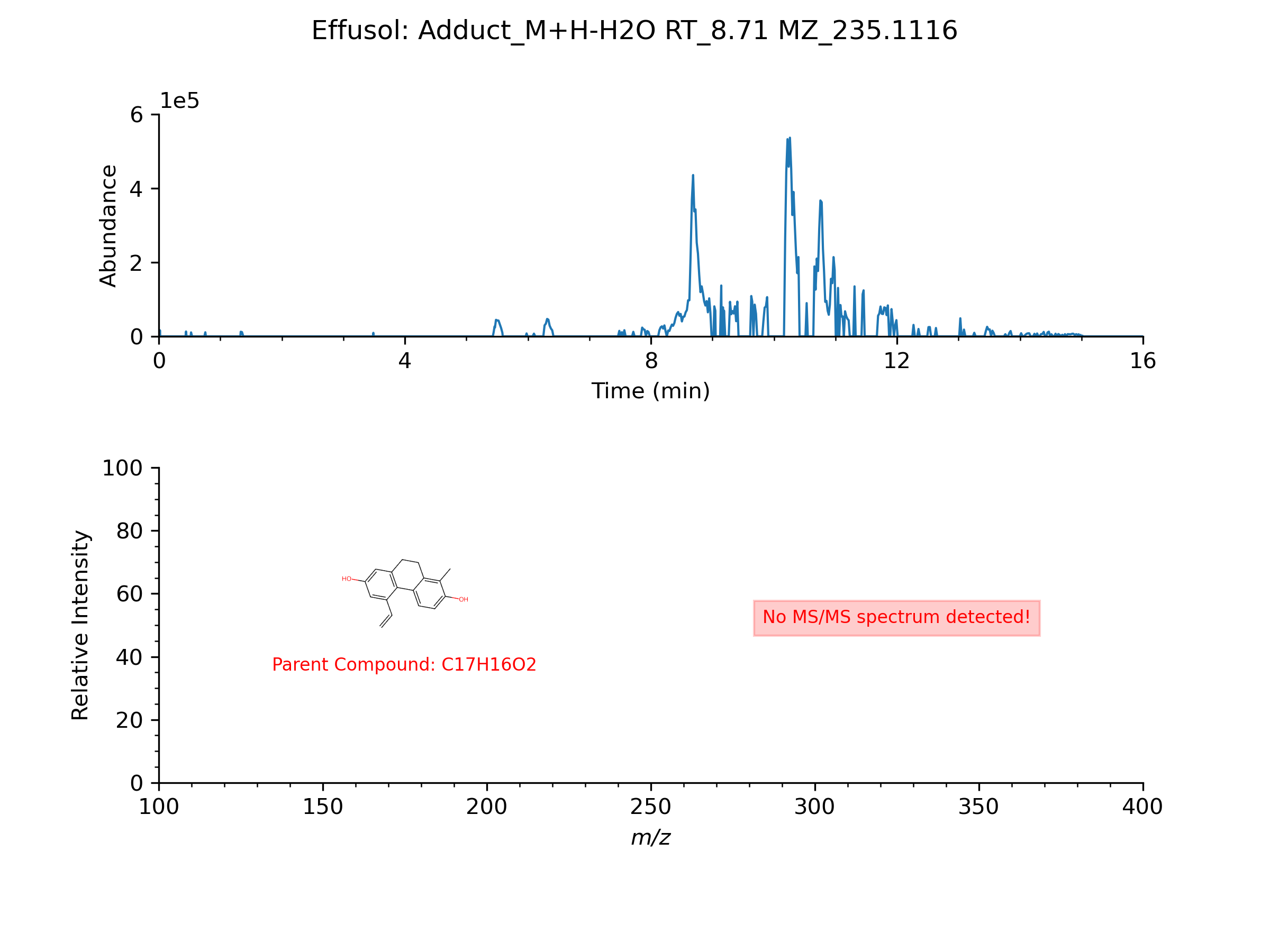

Supplement: Supplementary file 1 [file pharmaceuticals-18-01153-s001.zip › compound structures/M0171.png]

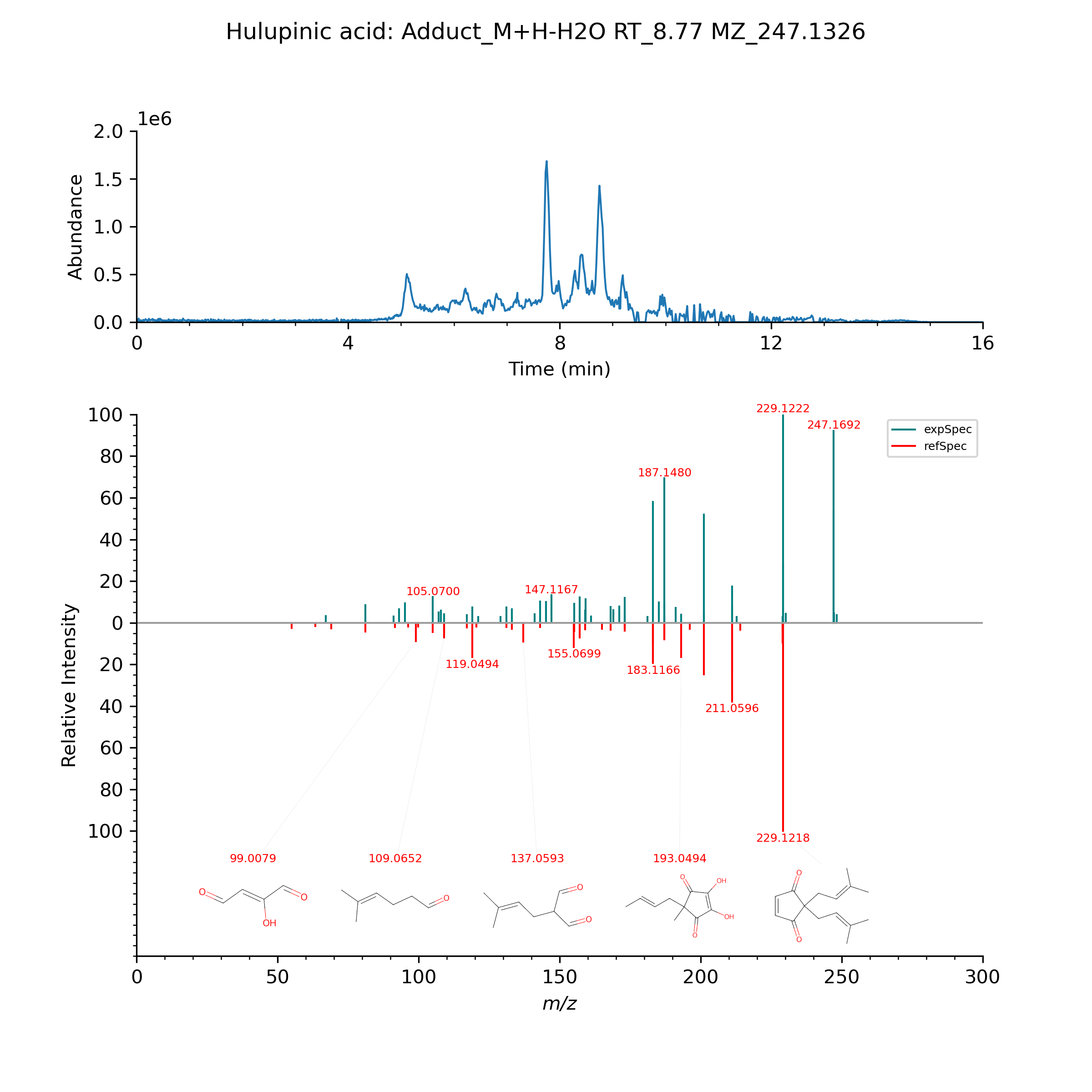

Supplement: Supplementary file 1 [file pharmaceuticals-18-01153-s001.zip › compound structures/M0172.png]

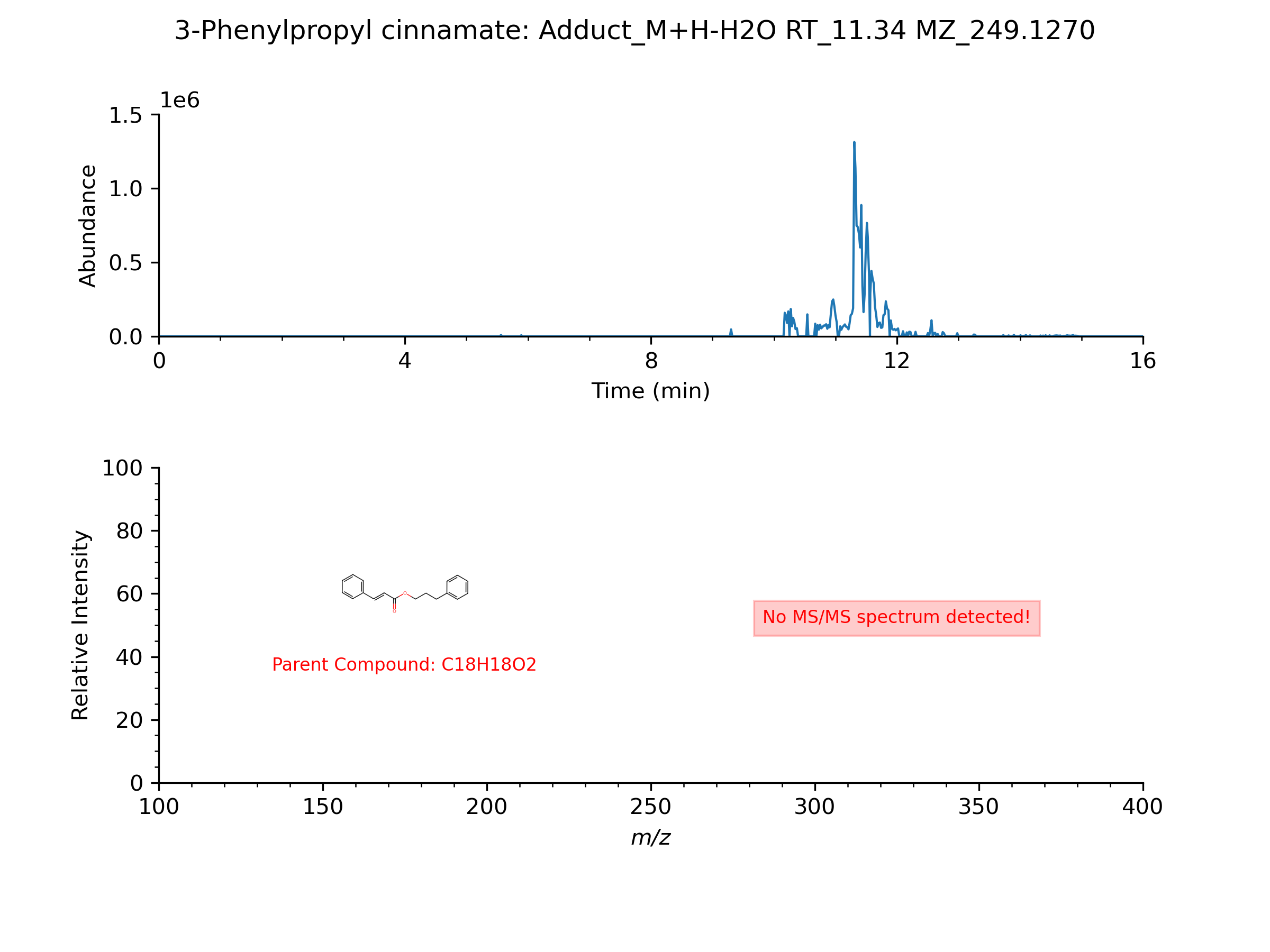

Supplement: Supplementary file 1 [file pharmaceuticals-18-01153-s001.zip › compound structures/M0173.png]

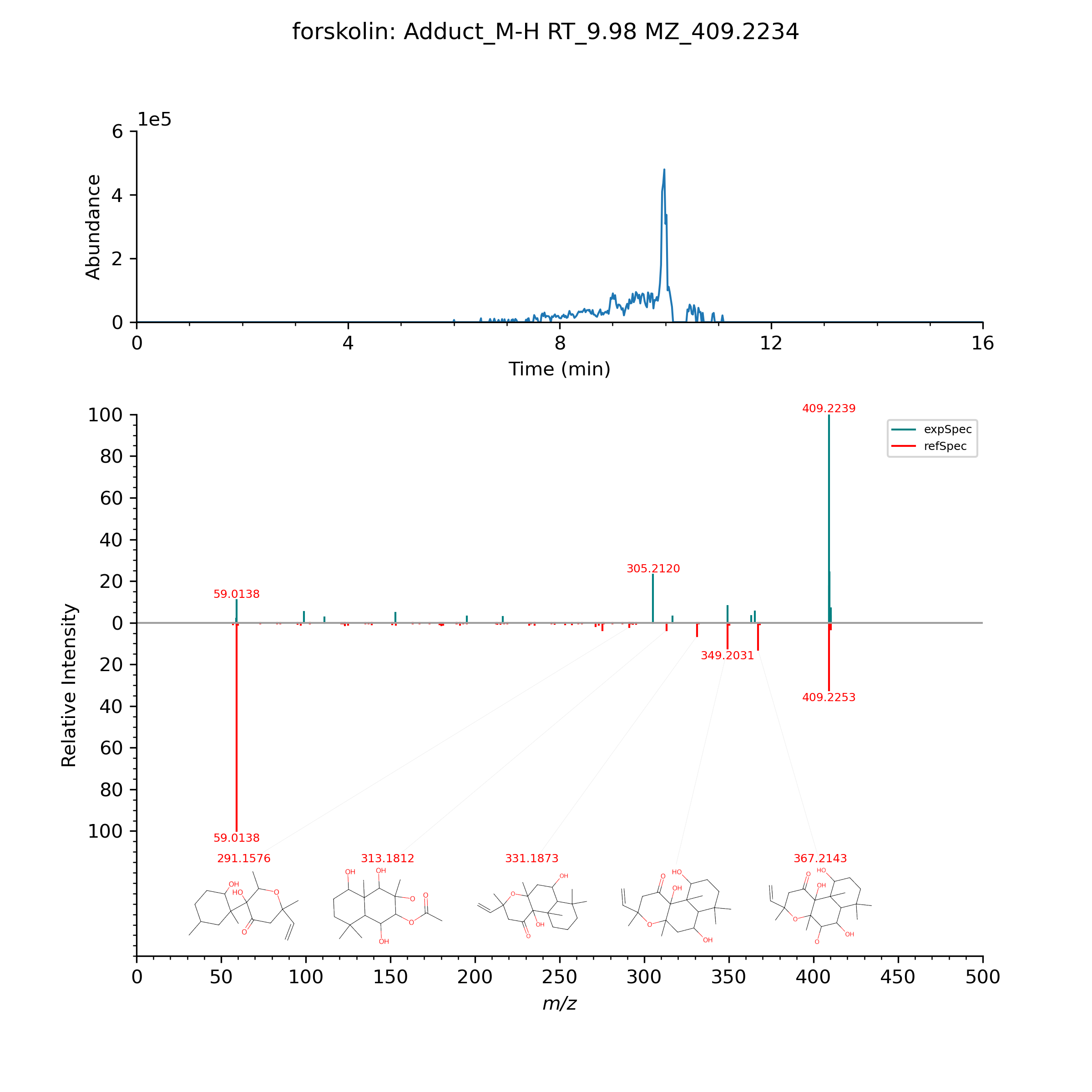

Supplement: Supplementary file 1 [file pharmaceuticals-18-01153-s001.zip › compound structures/M0174.png]

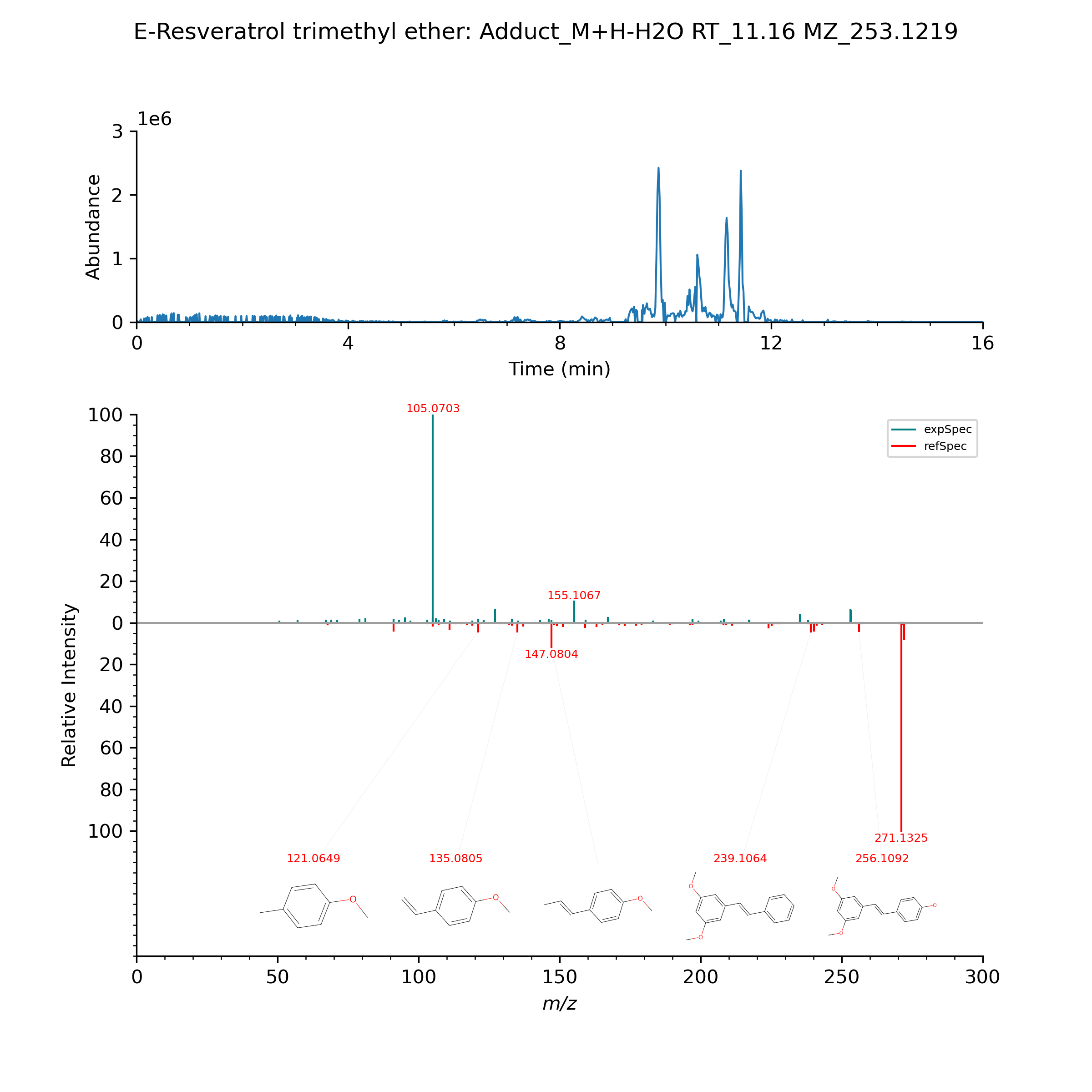

Supplement: Supplementary file 1 [file pharmaceuticals-18-01153-s001.zip › compound structures/M0175.png]

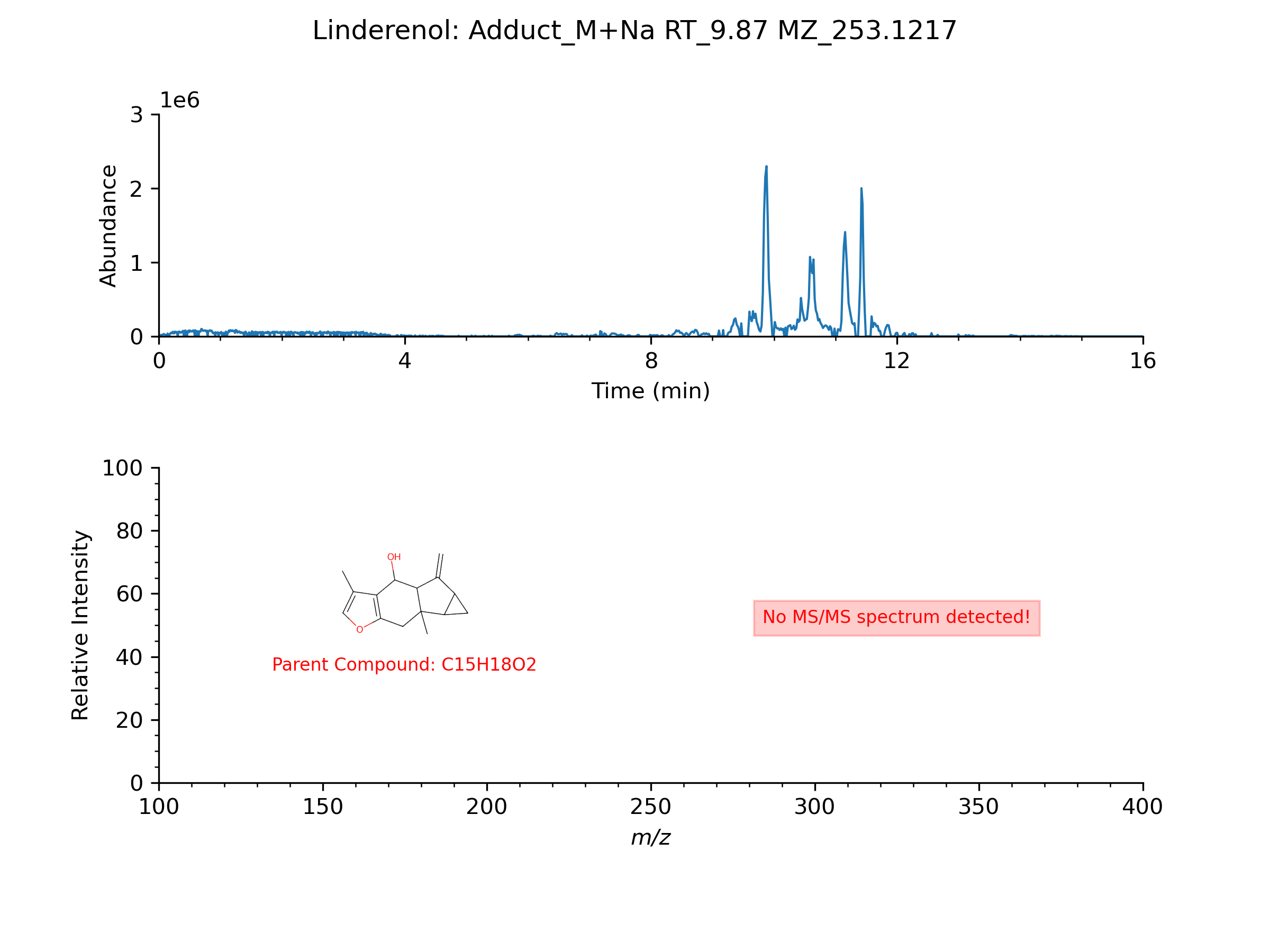

Supplement: Supplementary file 1 [file pharmaceuticals-18-01153-s001.zip › compound structures/M0176.png]

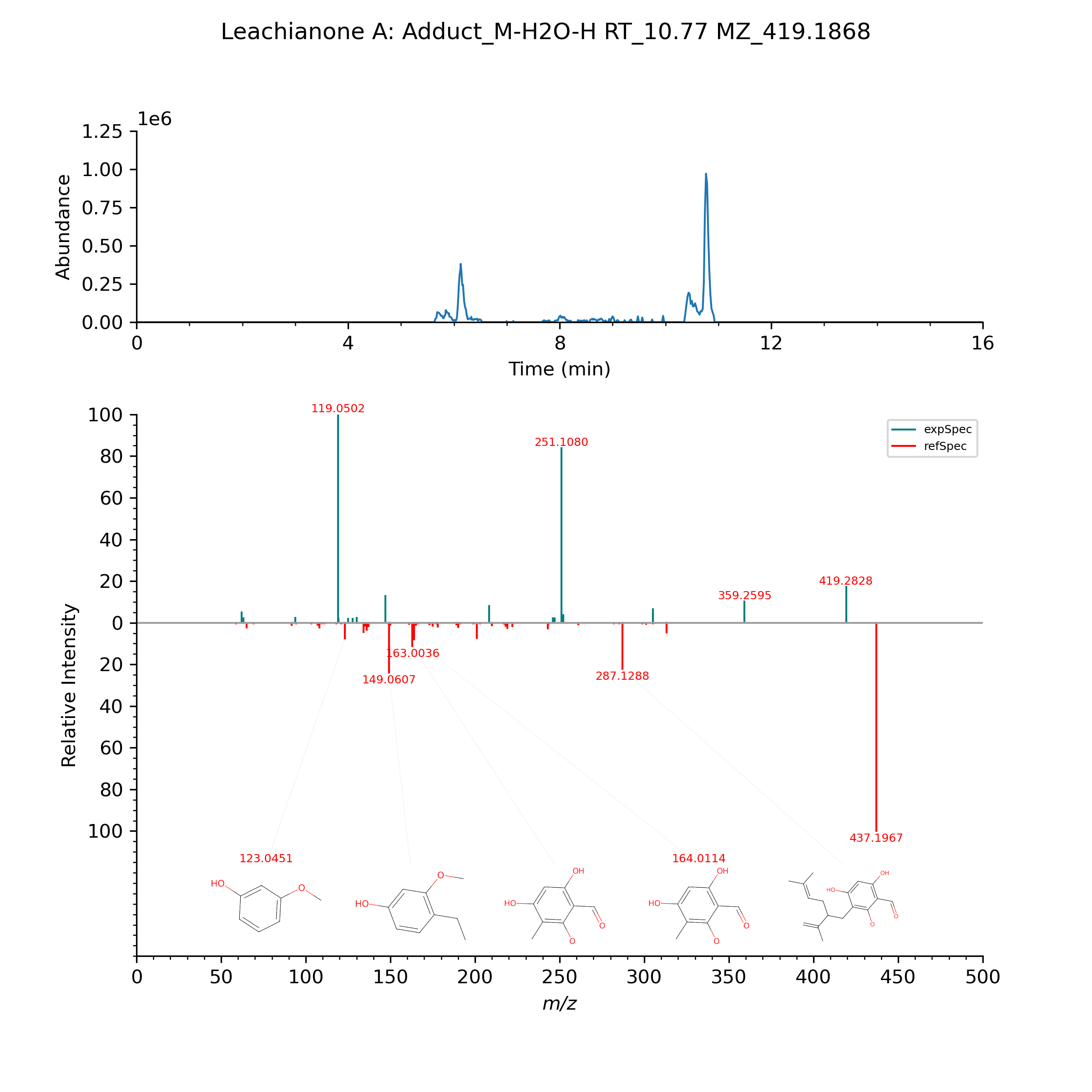

Supplement: Supplementary file 1 [file pharmaceuticals-18-01153-s001.zip › compound structures/M0177.png]

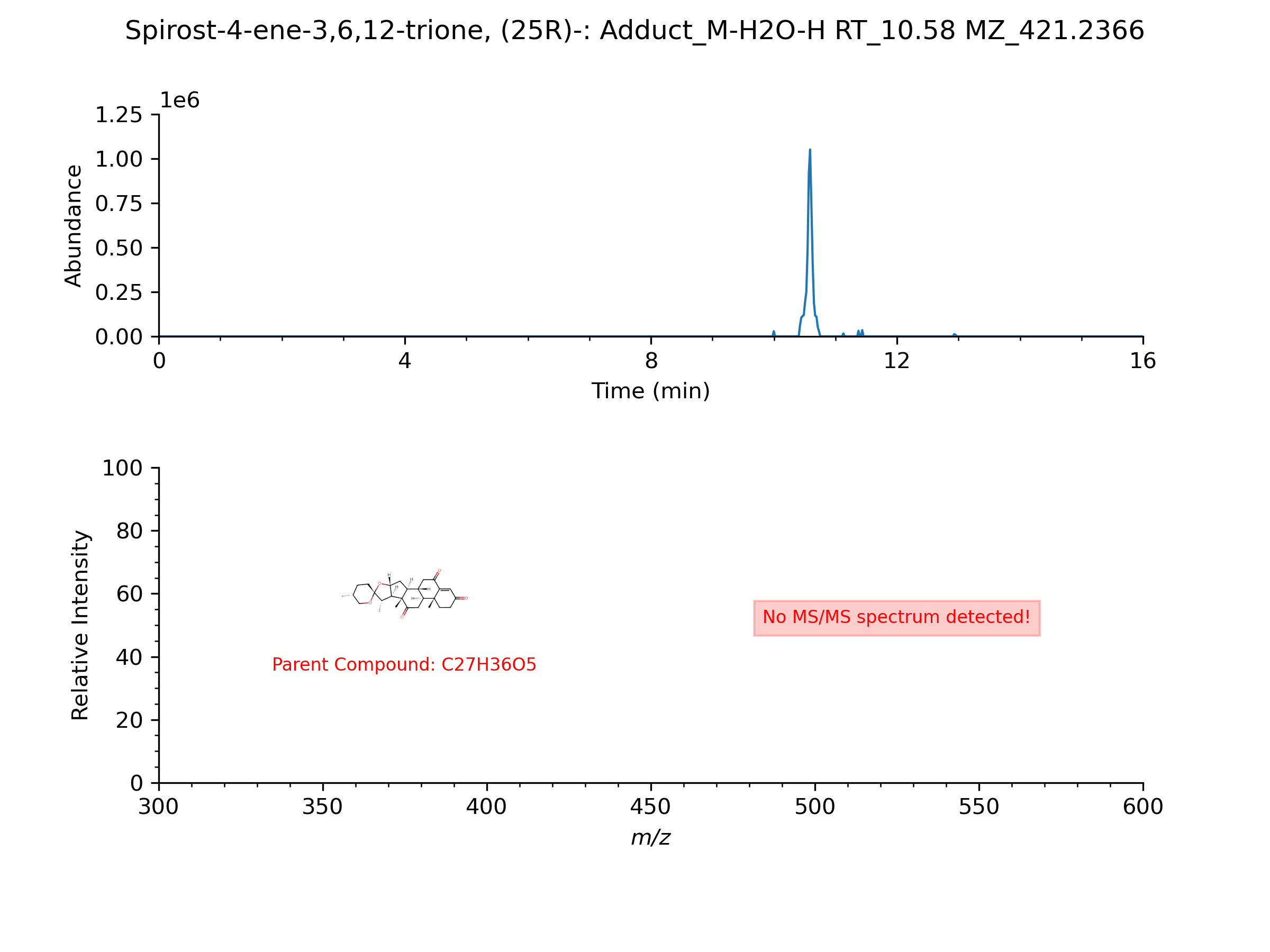

Supplement: Supplementary file 1 [file pharmaceuticals-18-01153-s001.zip › compound structures/M0178.png]

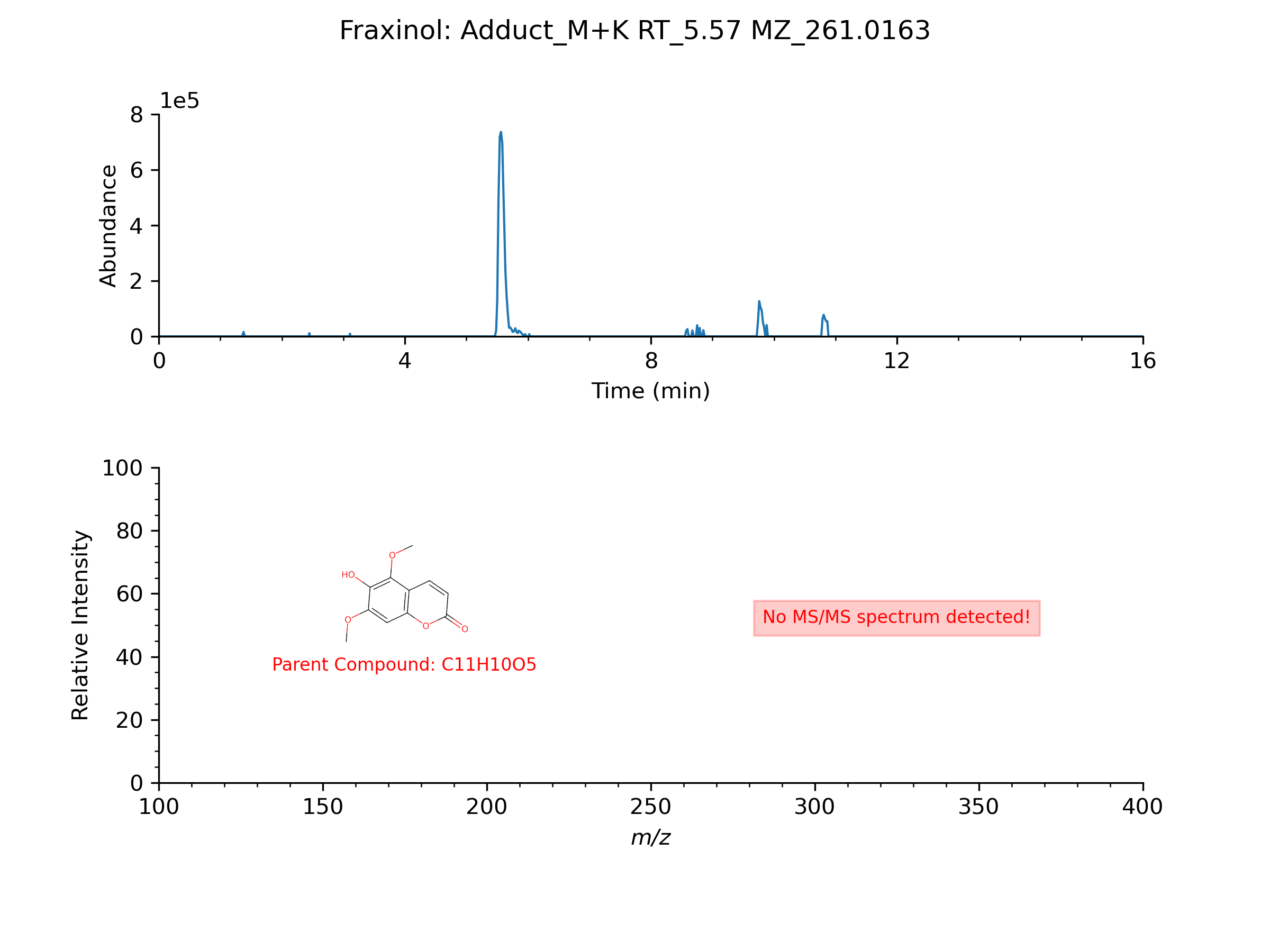

Supplement: Supplementary file 1 [file pharmaceuticals-18-01153-s001.zip › compound structures/M0179.png]

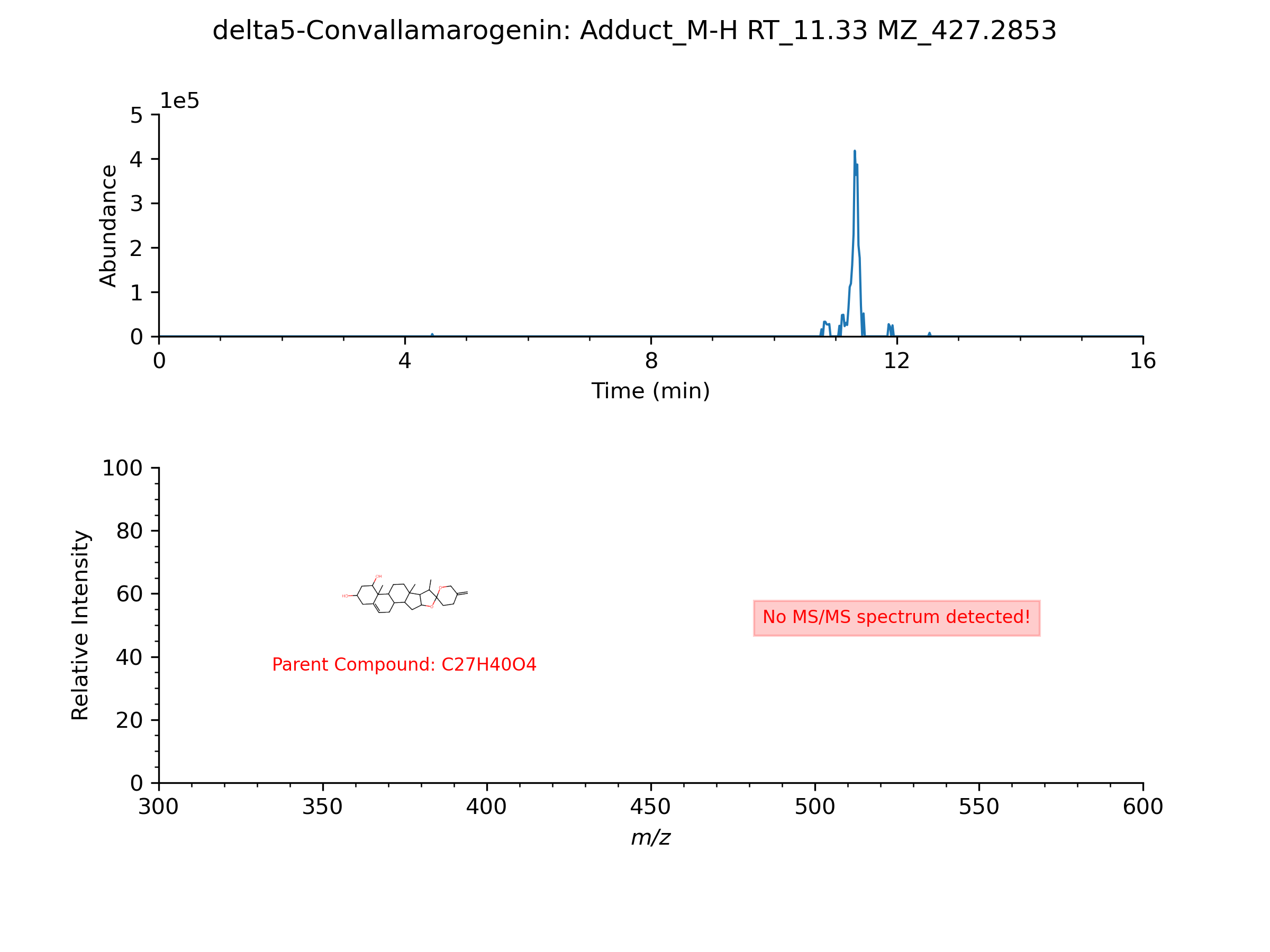

Supplement: Supplementary file 1 [file pharmaceuticals-18-01153-s001.zip › compound structures/M0180.png]

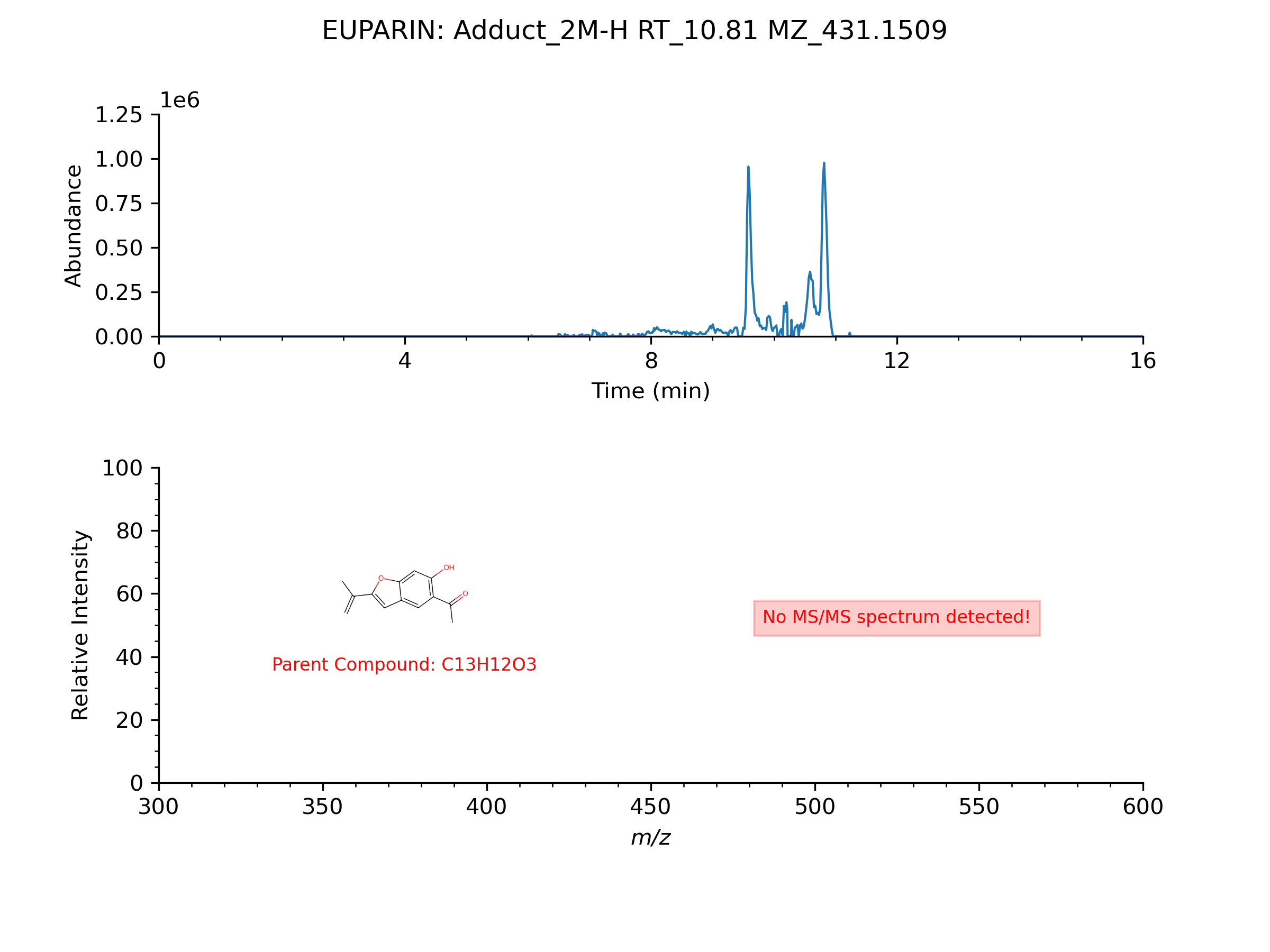

Supplement: Supplementary file 1 [file pharmaceuticals-18-01153-s001.zip › compound structures/M0181.png]

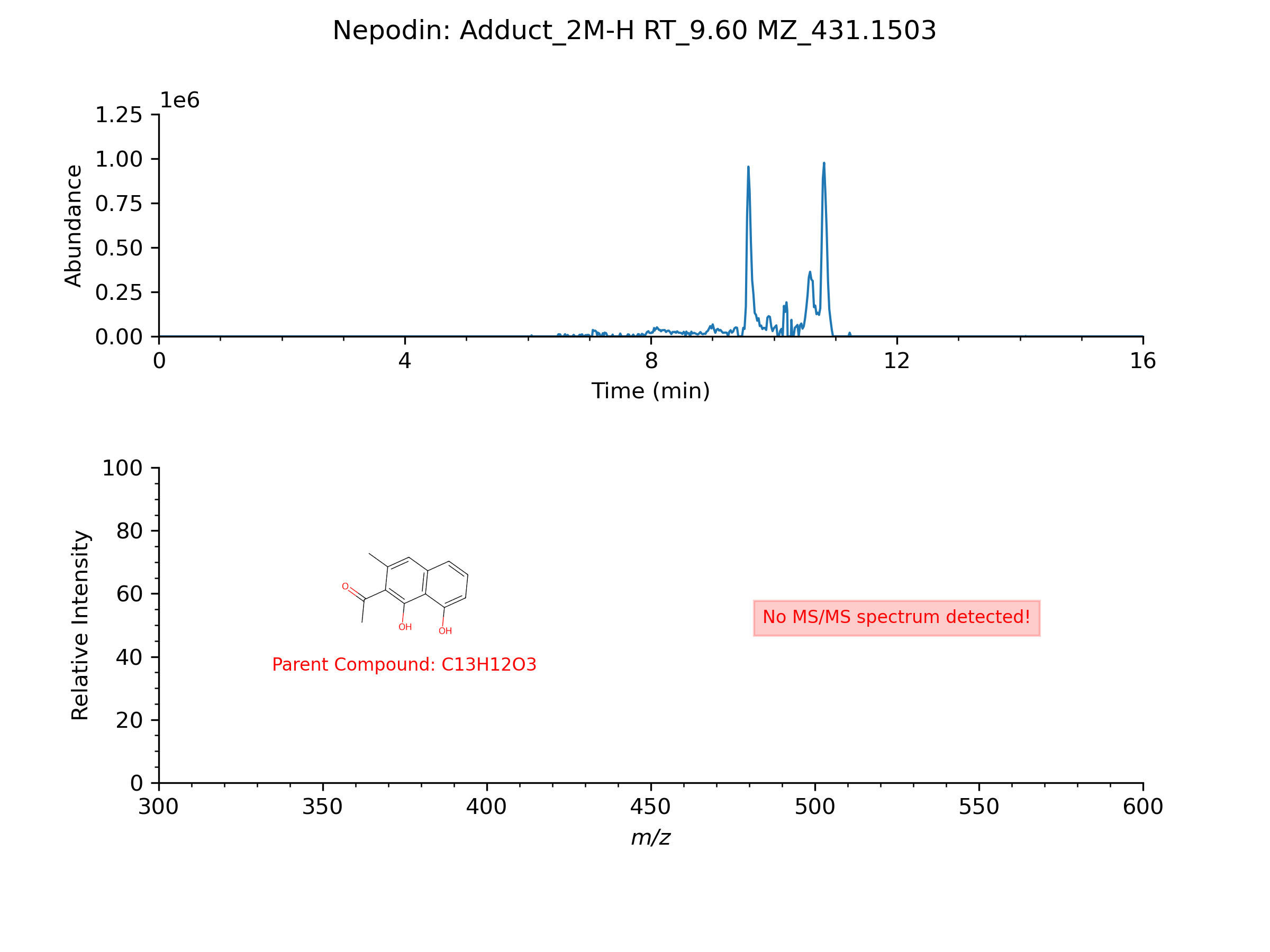

Supplement: Supplementary file 1 [file pharmaceuticals-18-01153-s001.zip › compound structures/M0182.png]

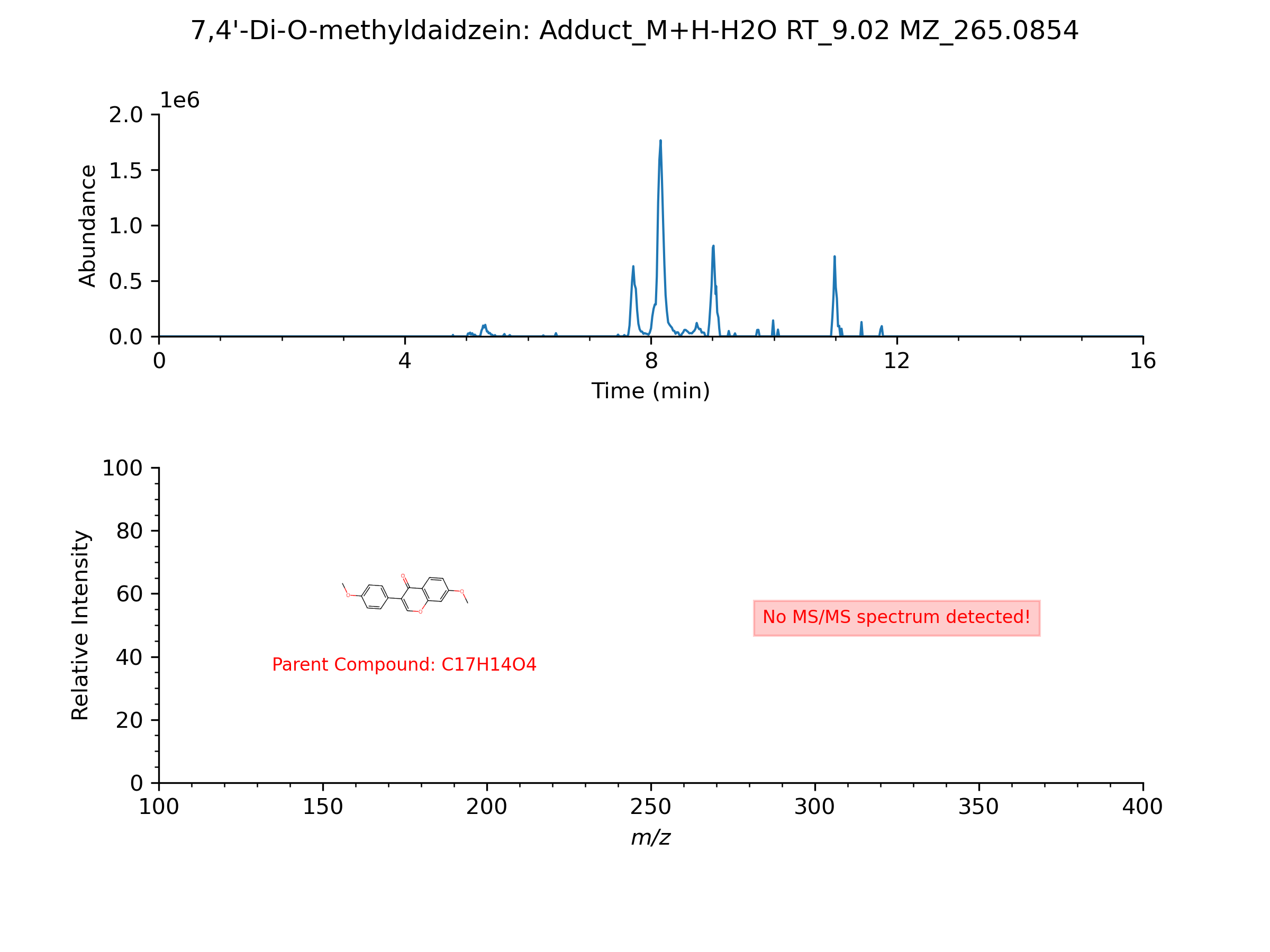

Supplement: Supplementary file 1 [file pharmaceuticals-18-01153-s001.zip › compound structures/M0183.png]

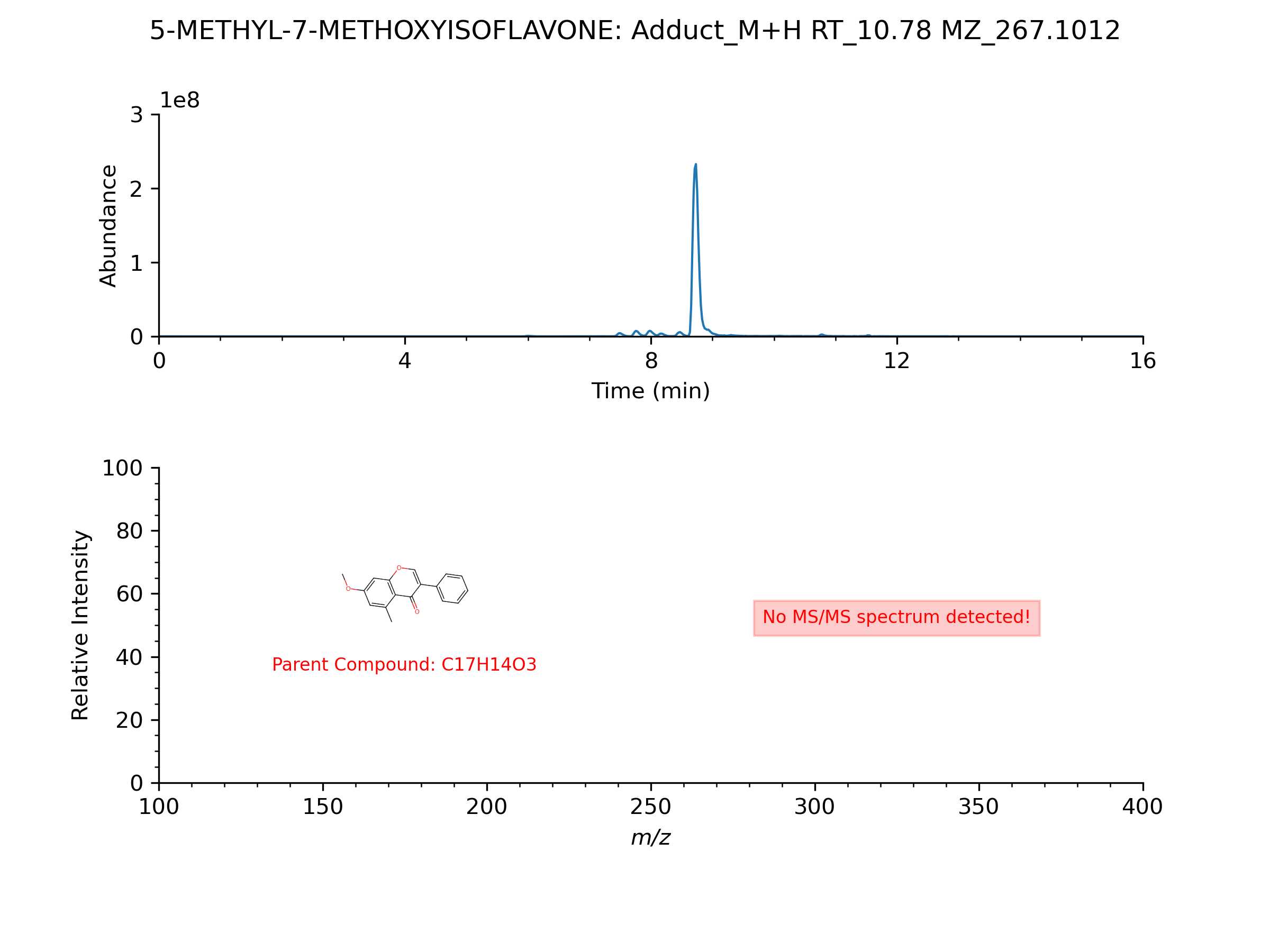

Supplement: Supplementary file 1 [file pharmaceuticals-18-01153-s001.zip › compound structures/M0184.png]

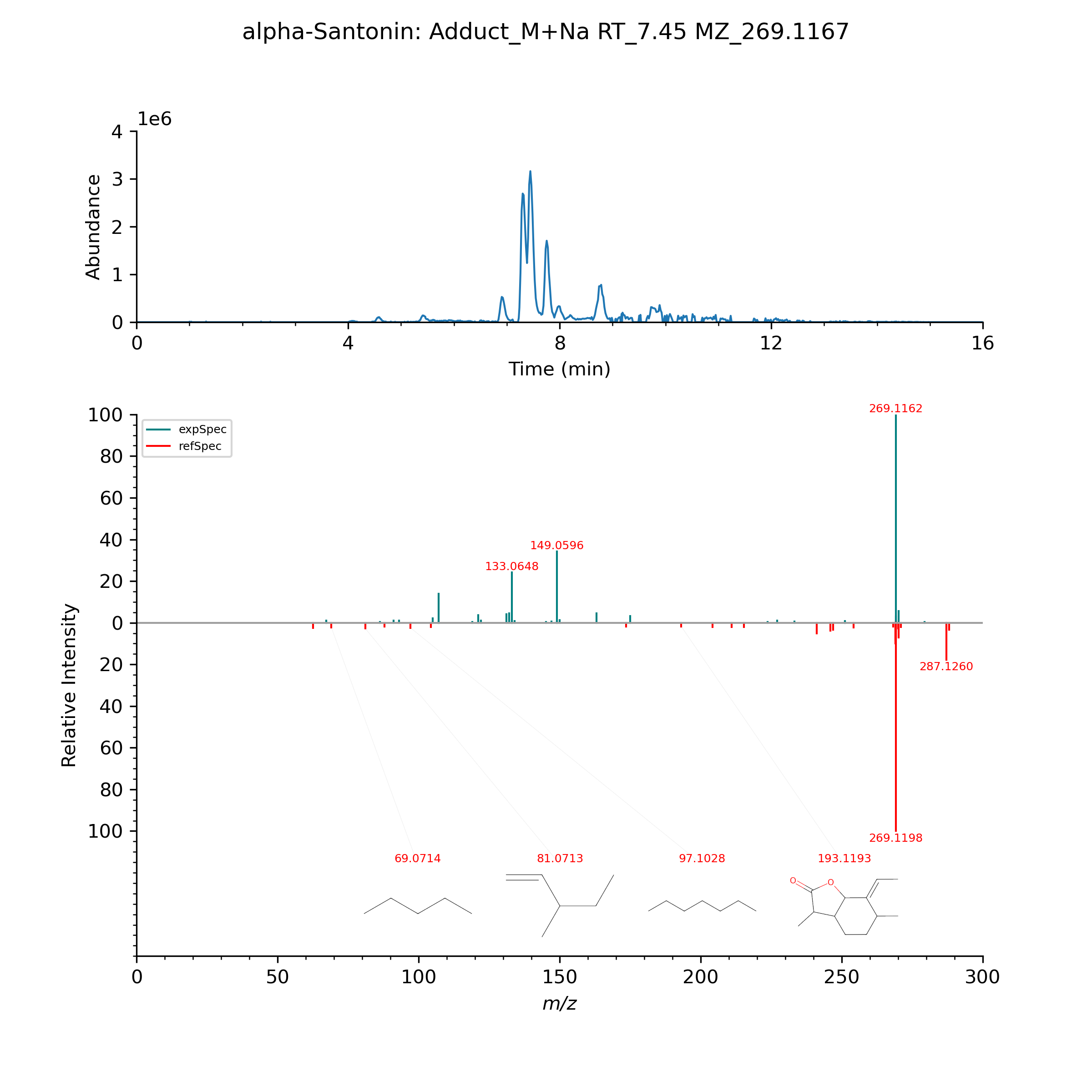

Supplement: Supplementary file 1 [file pharmaceuticals-18-01153-s001.zip › compound structures/M0185.png]

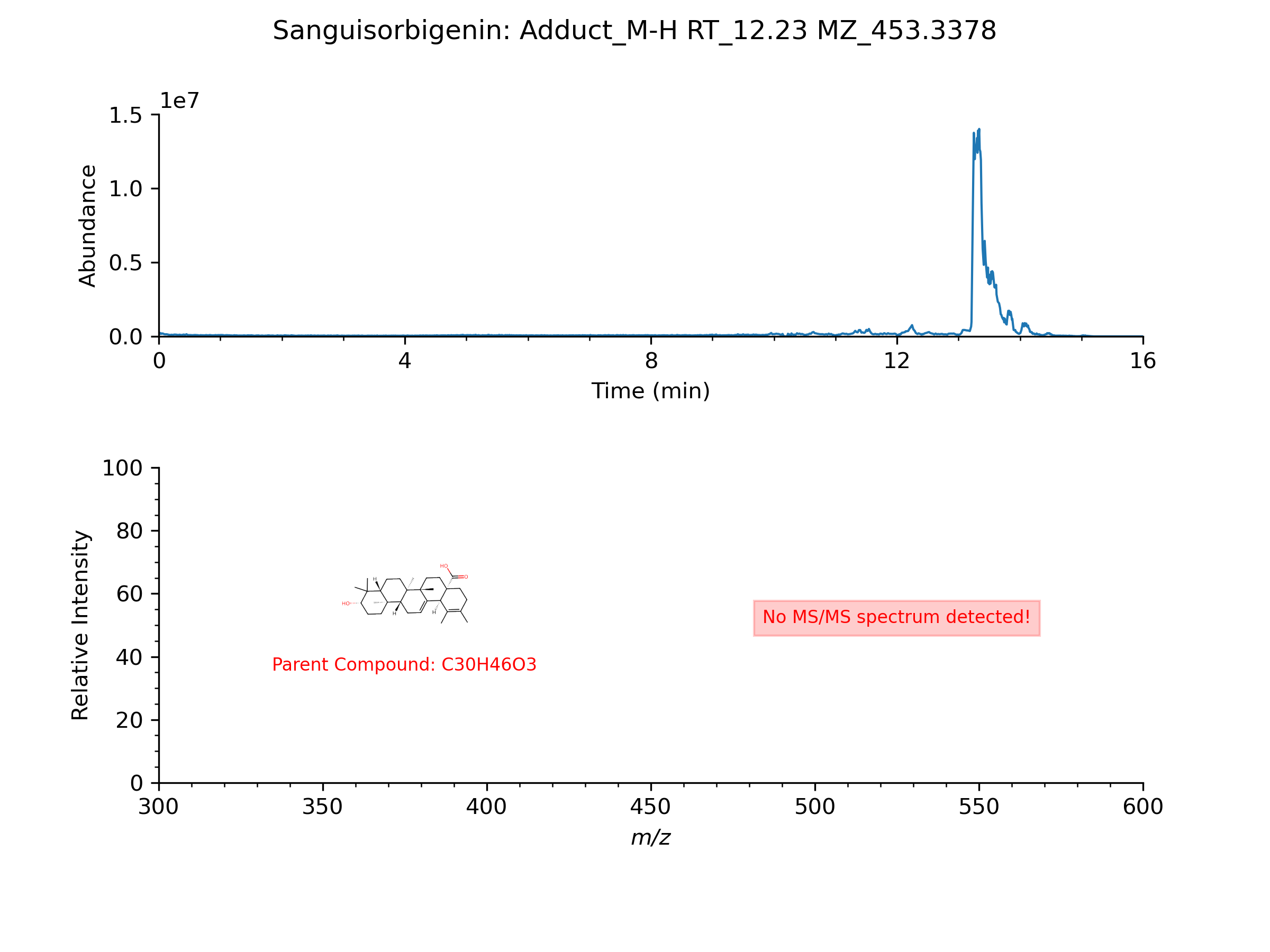

Supplement: Supplementary file 1 [file pharmaceuticals-18-01153-s001.zip › compound structures/M0186.png]

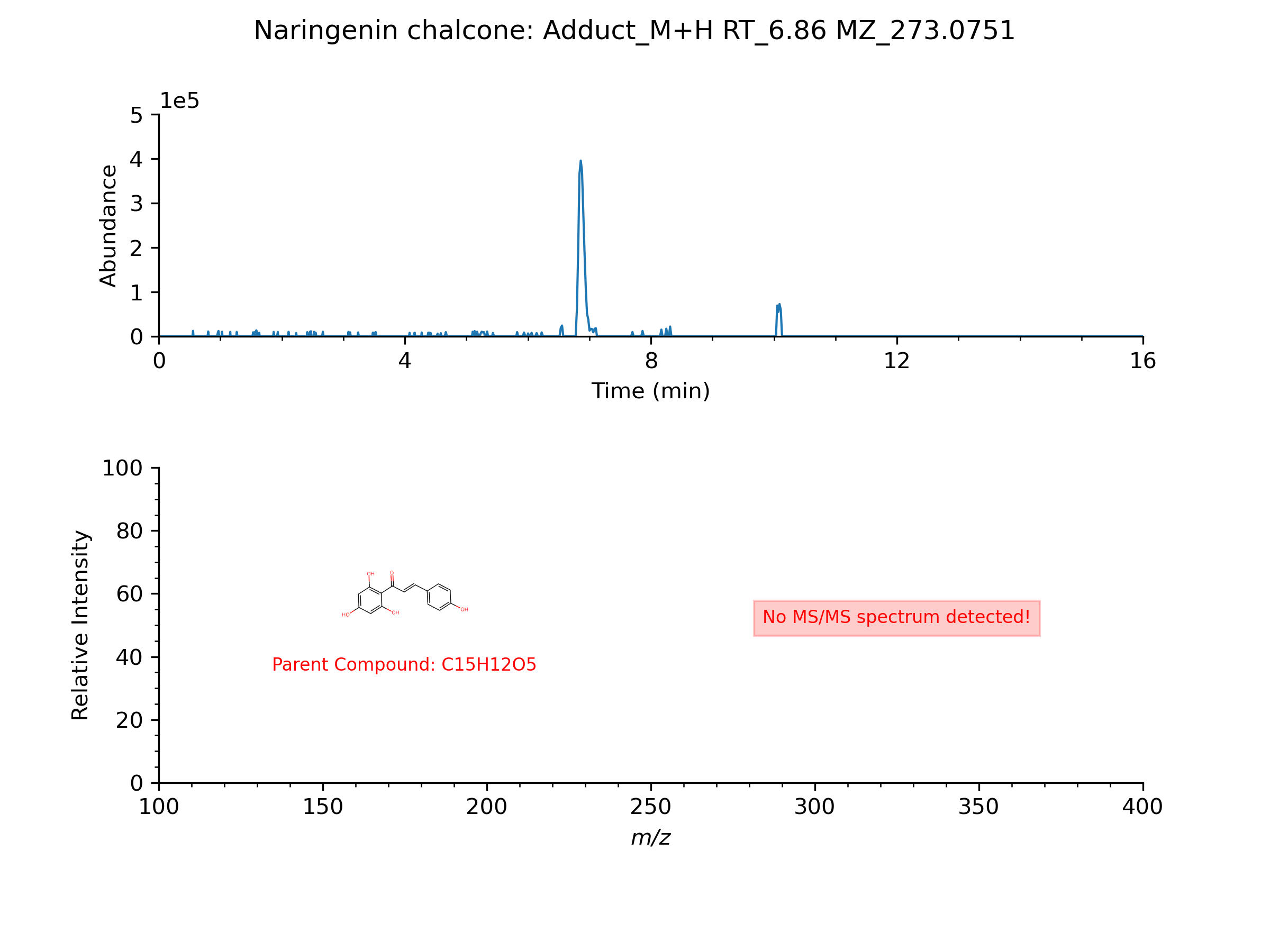

Supplement: Supplementary file 1 [file pharmaceuticals-18-01153-s001.zip › compound structures/M0187.png]

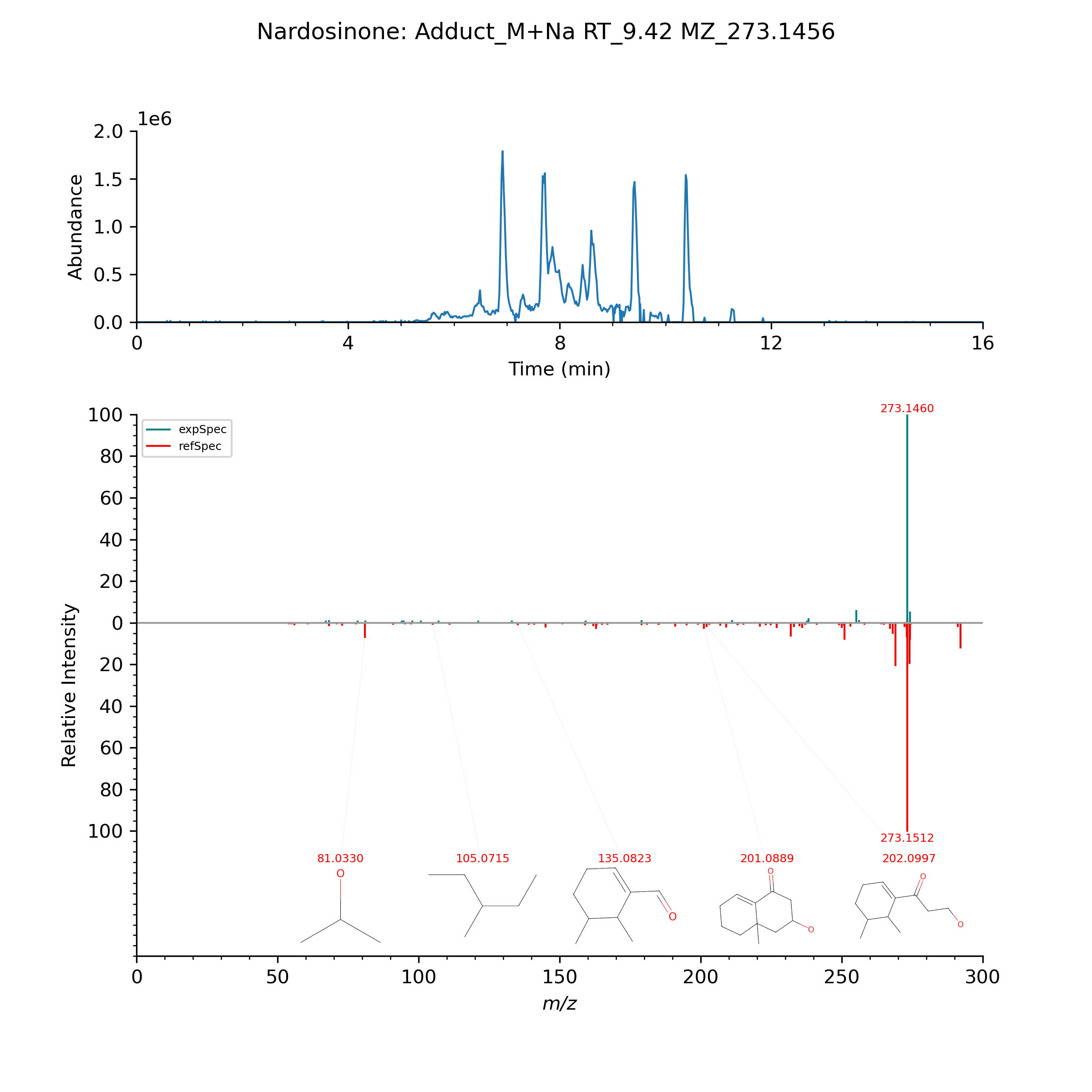

Supplement: Supplementary file 1 [file pharmaceuticals-18-01153-s001.zip › compound structures/M0188.png]

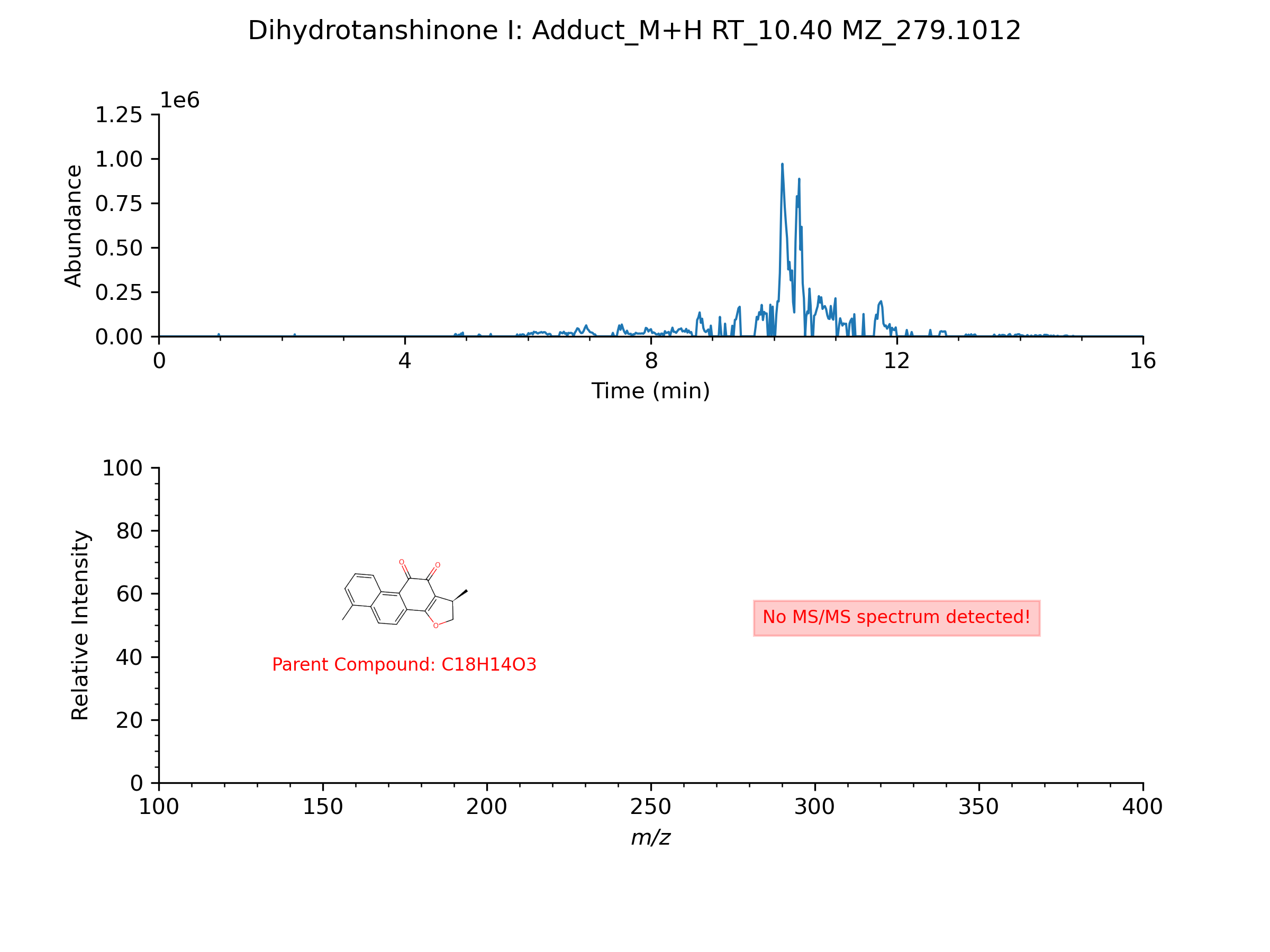

Supplement: Supplementary file 1 [file pharmaceuticals-18-01153-s001.zip › compound structures/M0189.png]

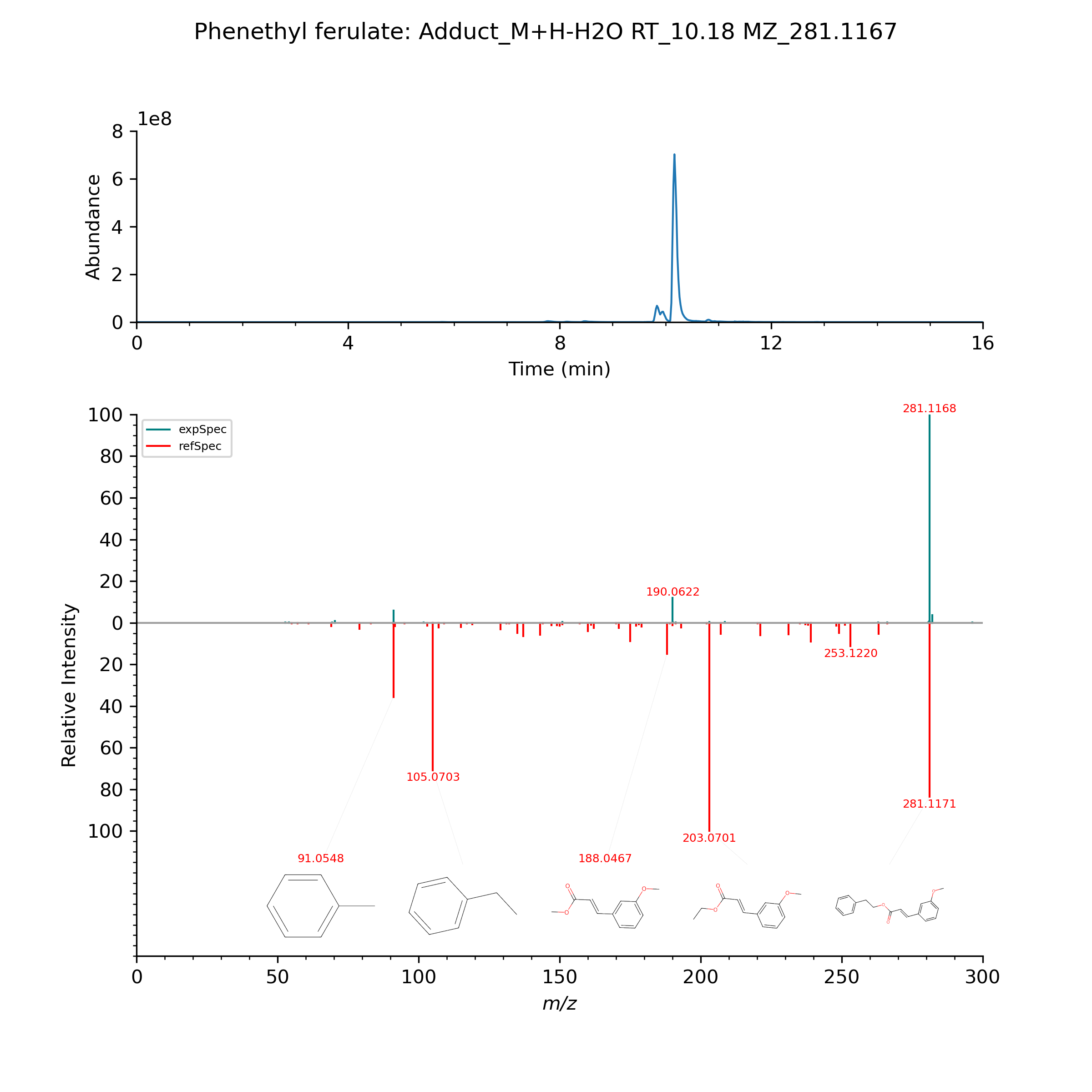

Supplement: Supplementary file 1 [file pharmaceuticals-18-01153-s001.zip › compound structures/M0190.png]

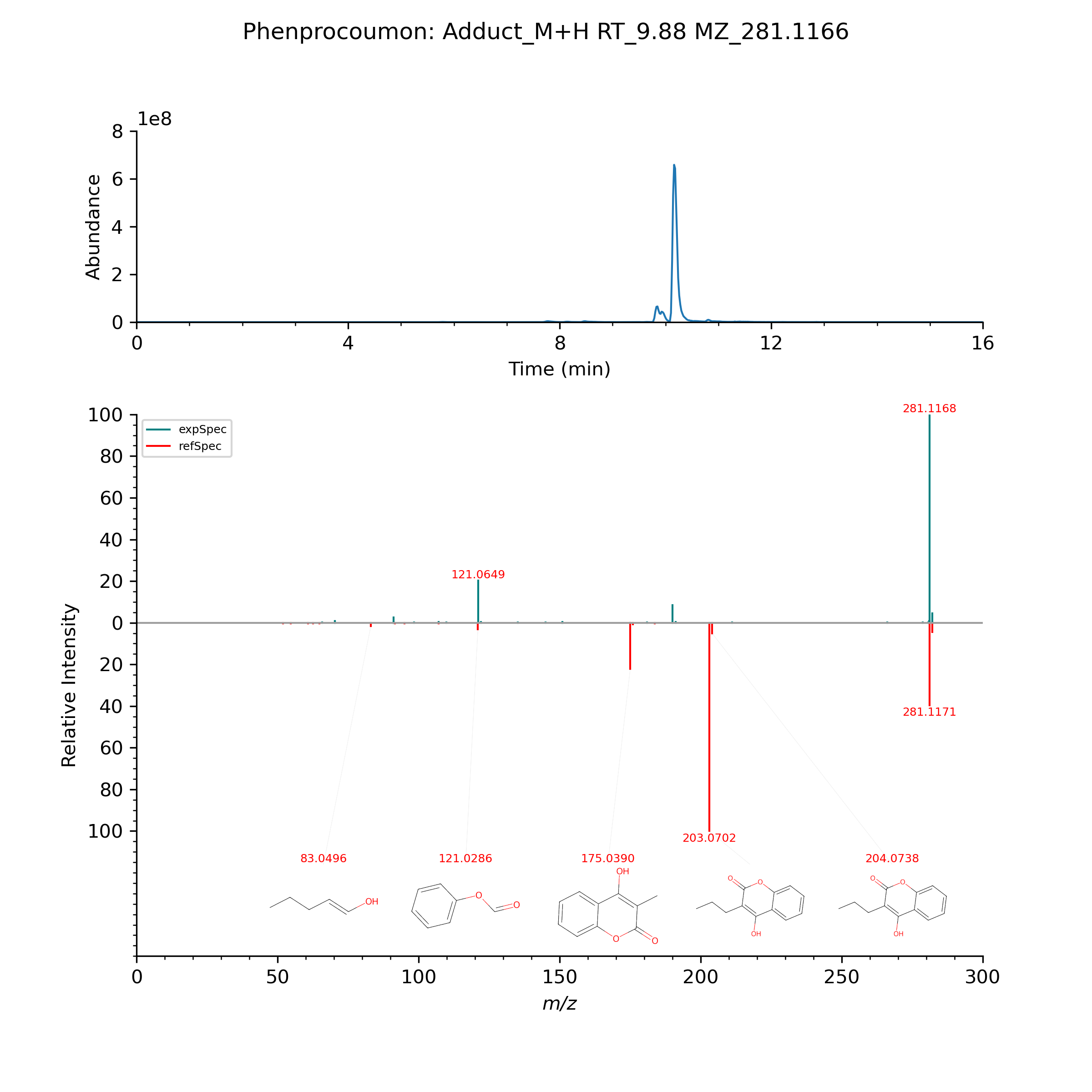

Supplement: Supplementary file 1 [file pharmaceuticals-18-01153-s001.zip › compound structures/M0191.png]

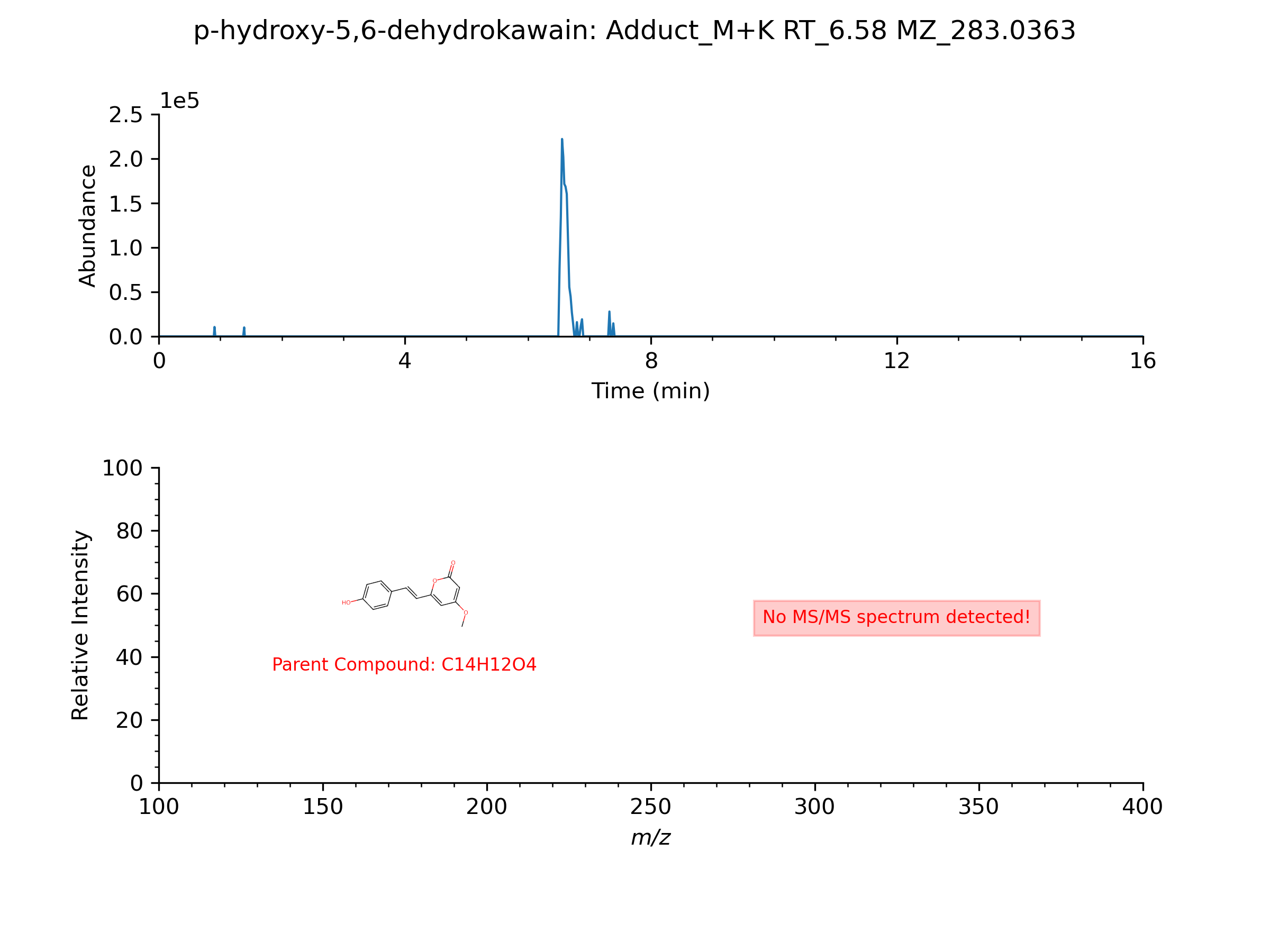

Supplement: Supplementary file 1 [file pharmaceuticals-18-01153-s001.zip › compound structures/M0192.png]

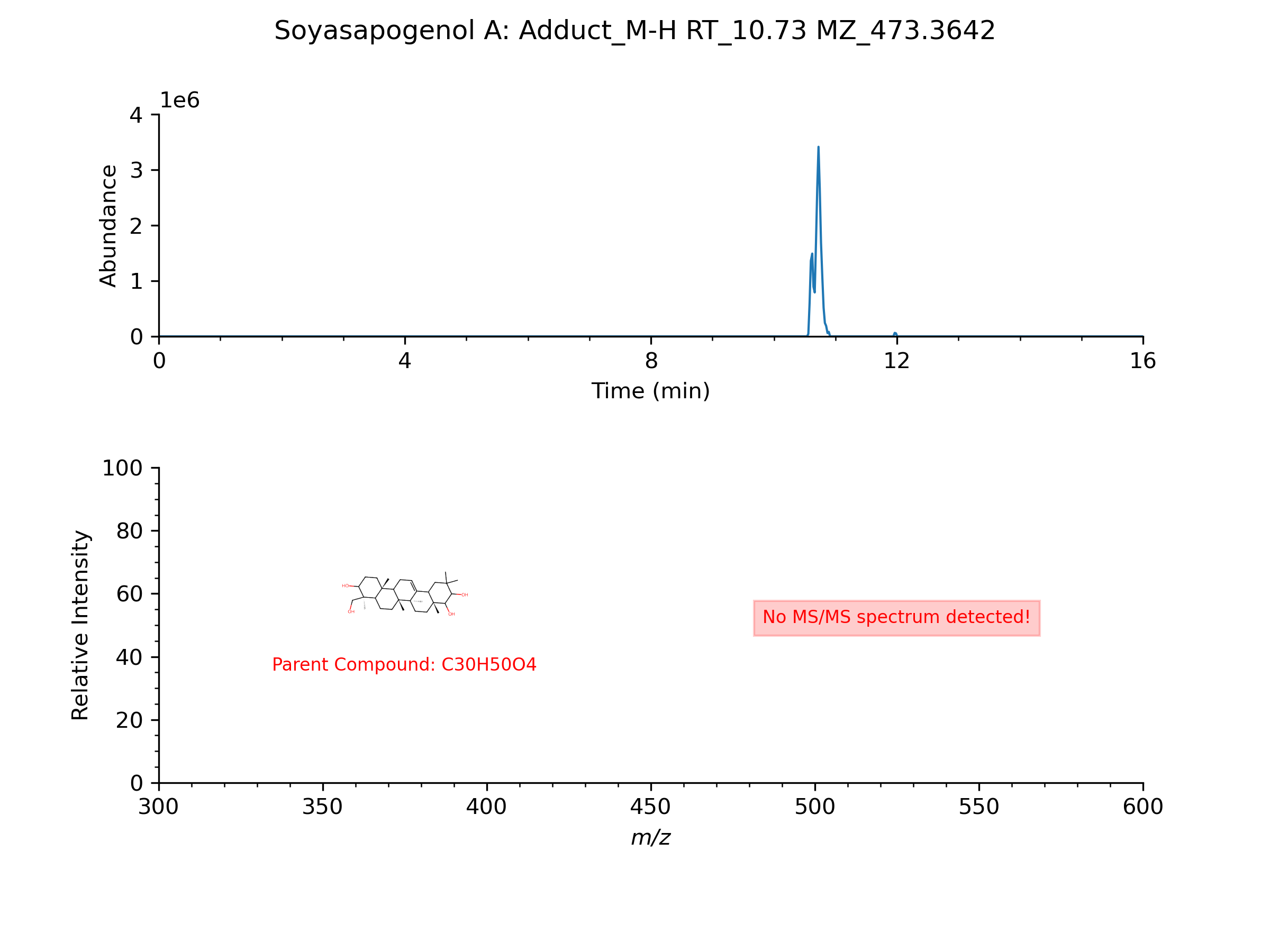

Supplement: Supplementary file 1 [file pharmaceuticals-18-01153-s001.zip › compound structures/M0193.png]

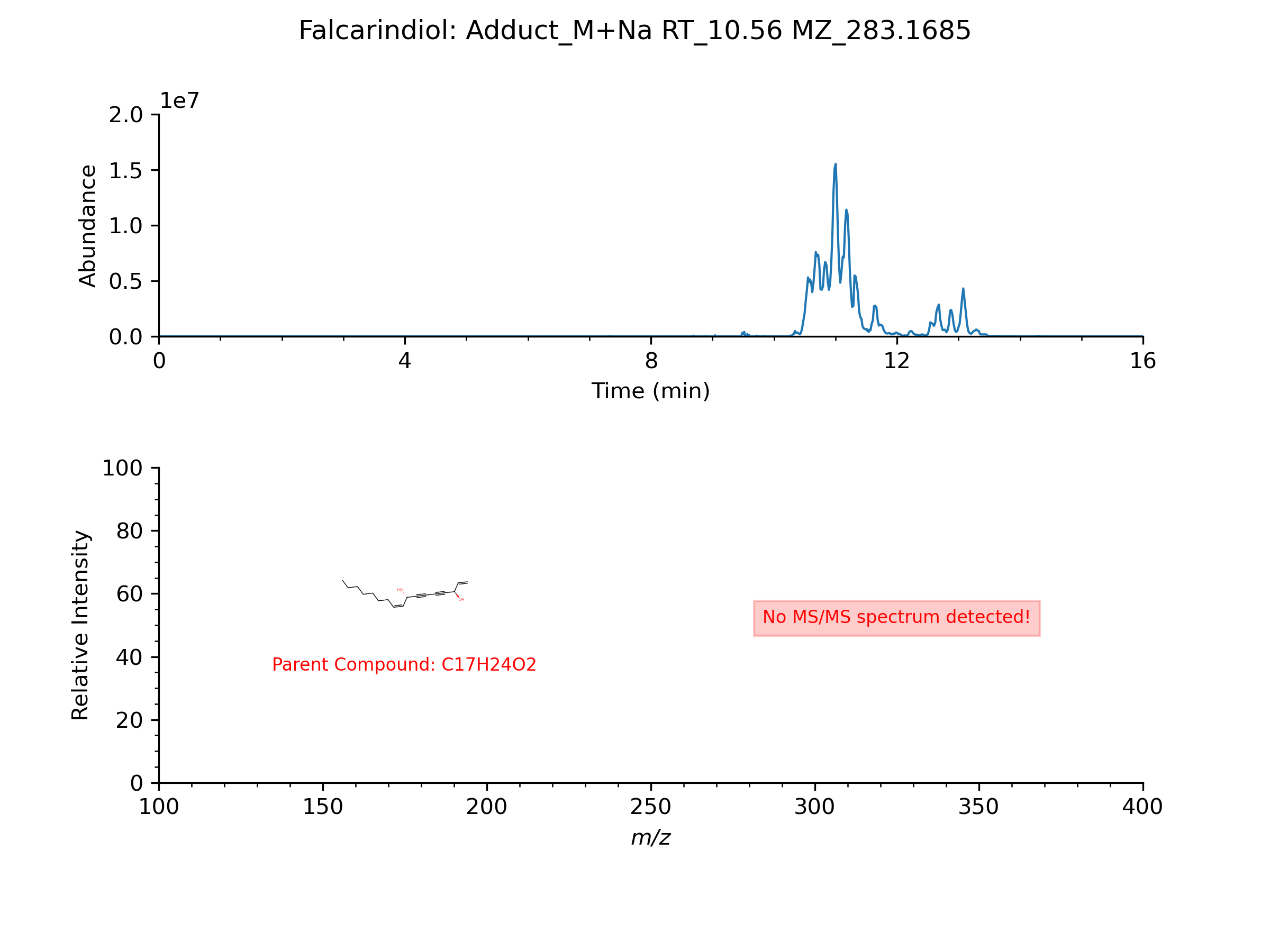

Supplement: Supplementary file 1 [file pharmaceuticals-18-01153-s001.zip › compound structures/M0194.png]

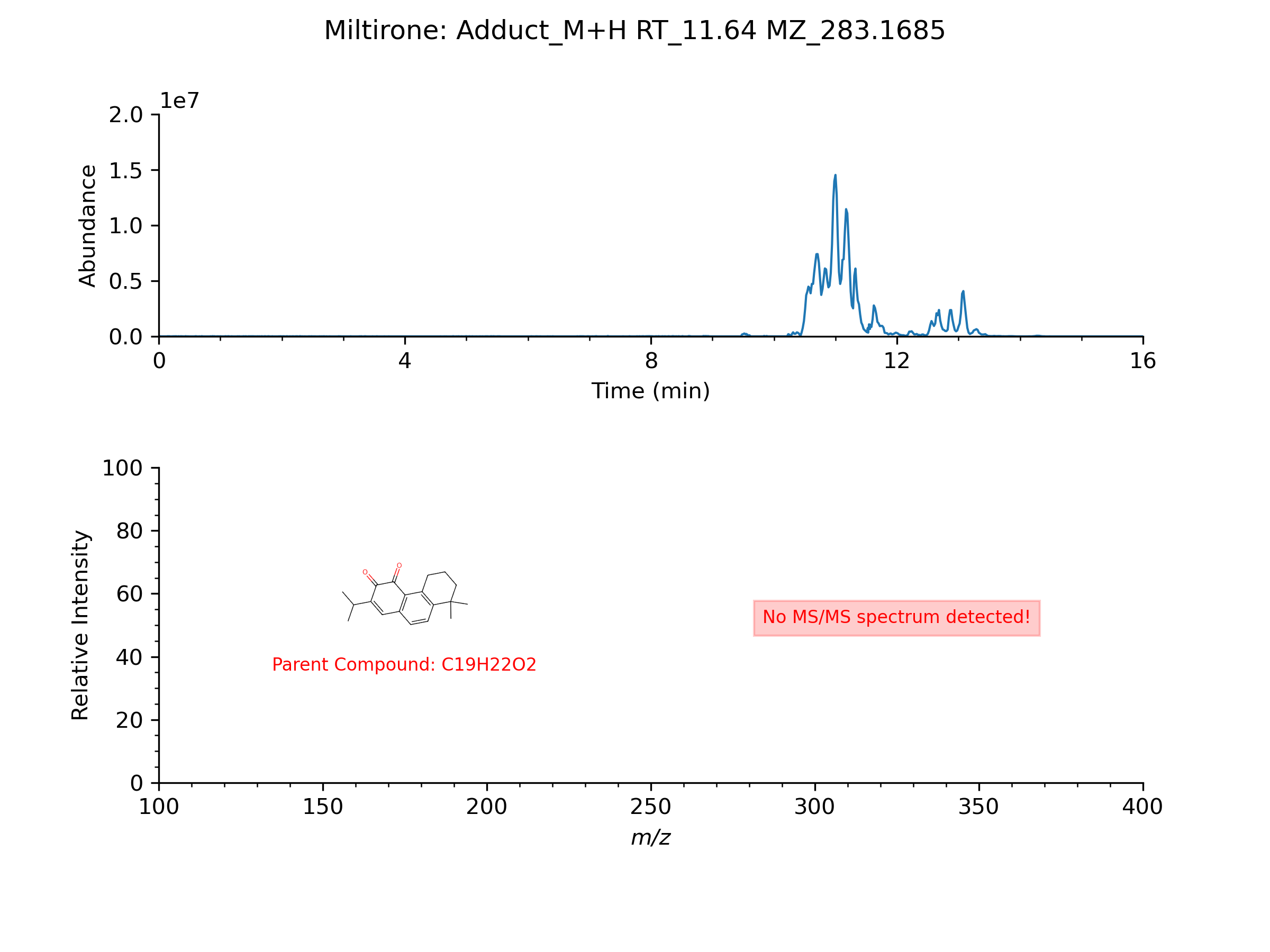

Supplement: Supplementary file 1 [file pharmaceuticals-18-01153-s001.zip › compound structures/M0195.png]

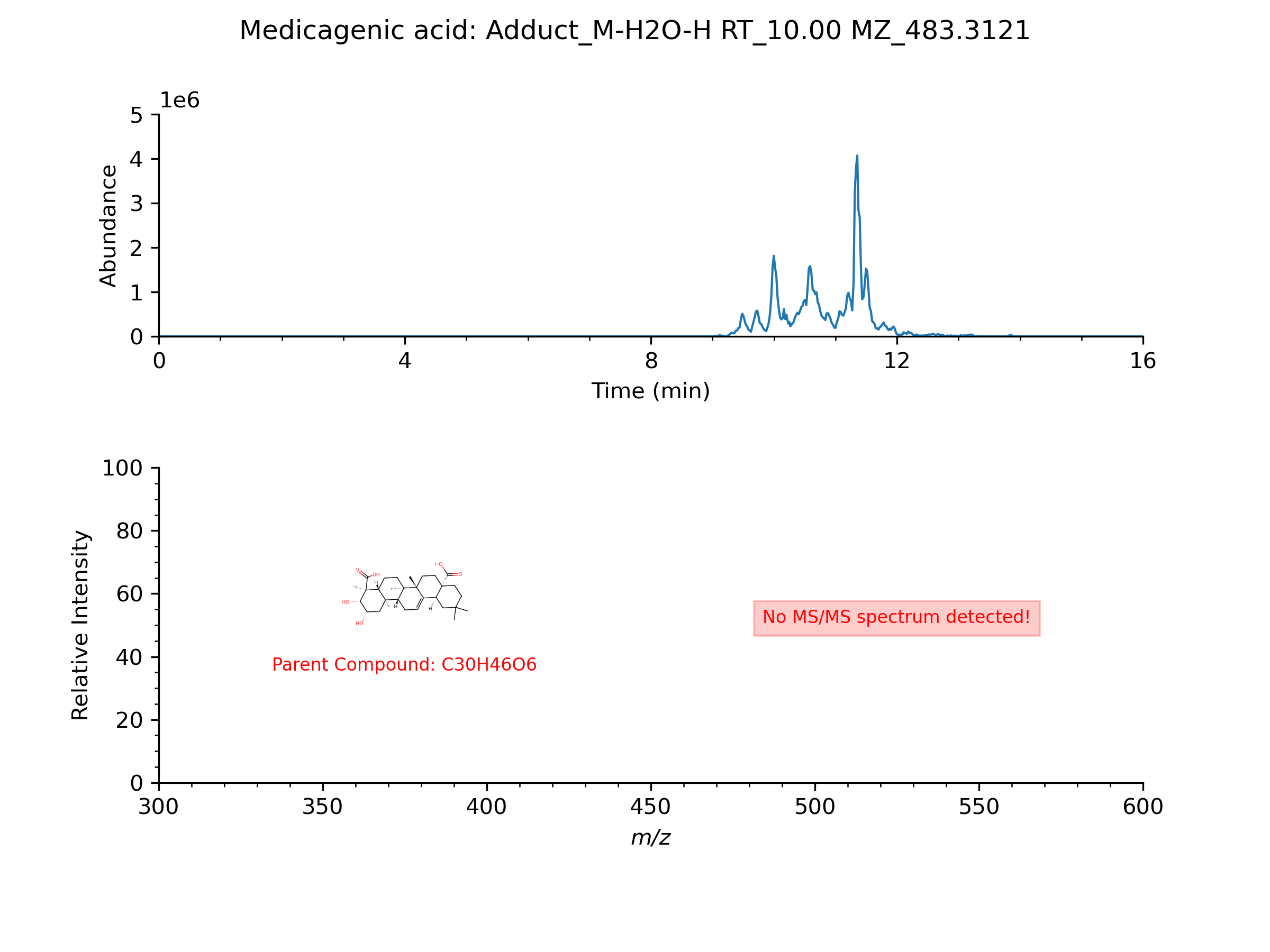

Supplement: Supplementary file 1 [file pharmaceuticals-18-01153-s001.zip › compound structures/M0196.png]

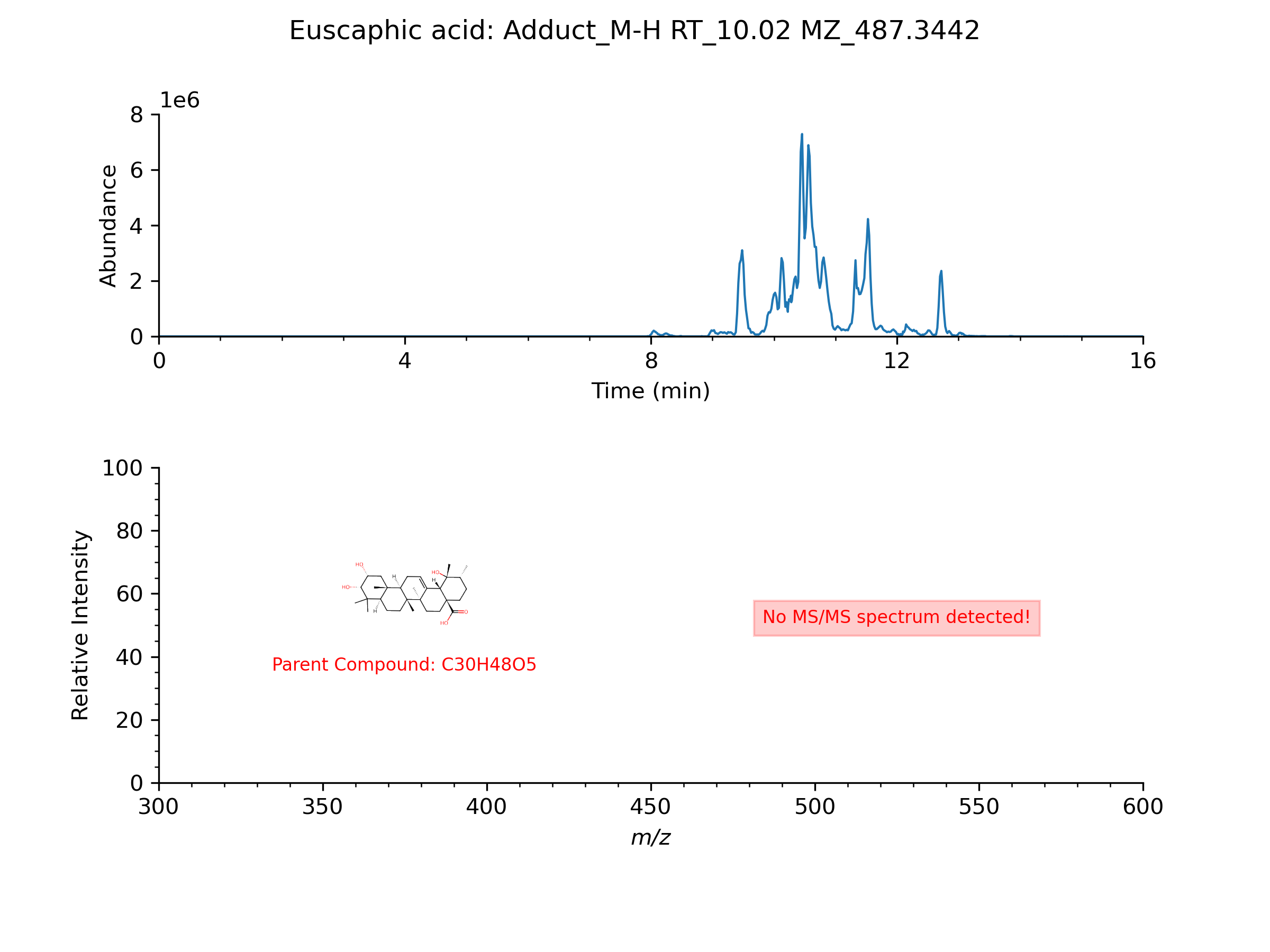

Supplement: Supplementary file 1 [file pharmaceuticals-18-01153-s001.zip › compound structures/M0197.png]

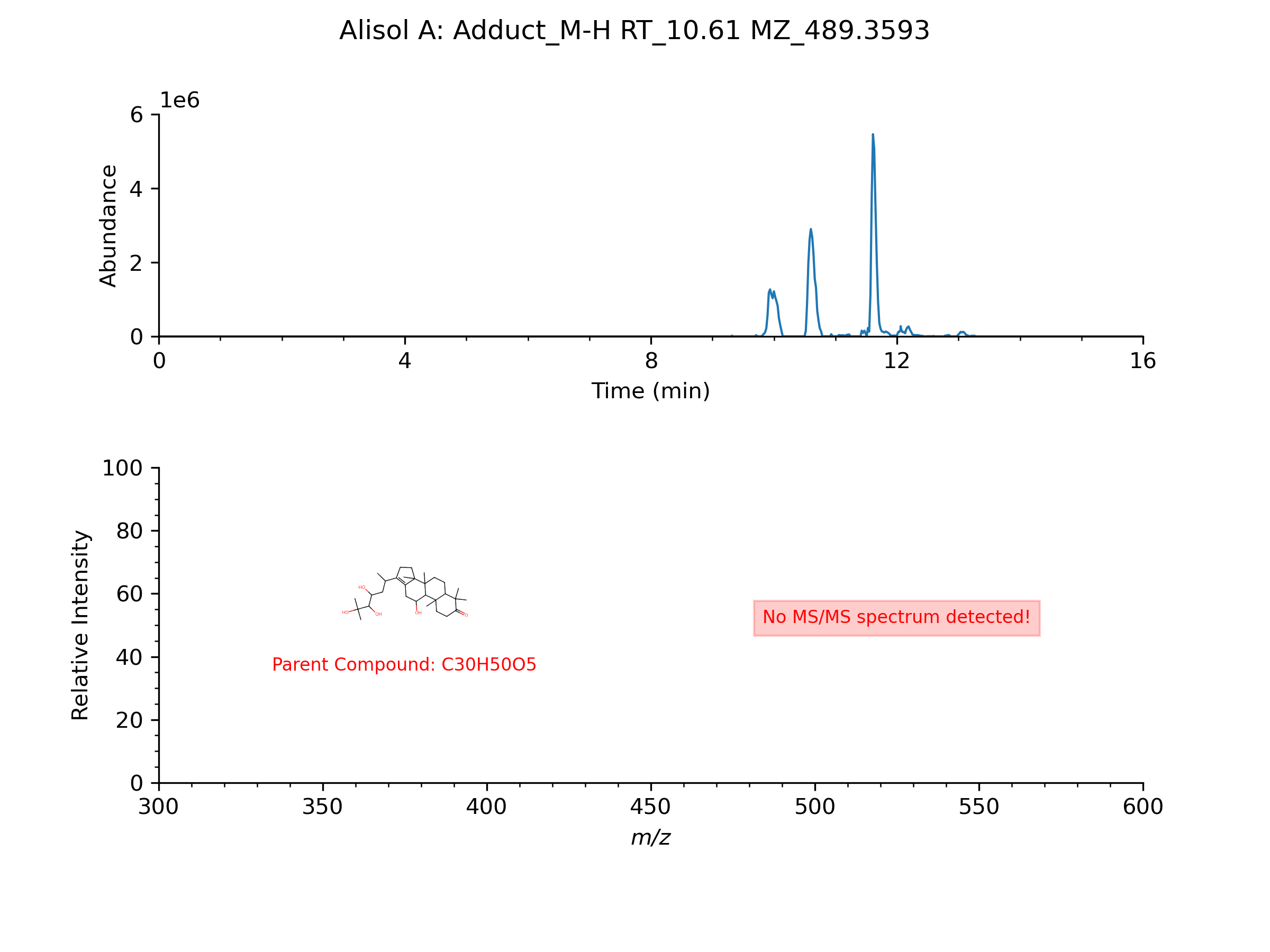

Supplement: Supplementary file 1 [file pharmaceuticals-18-01153-s001.zip › compound structures/M0198.png]

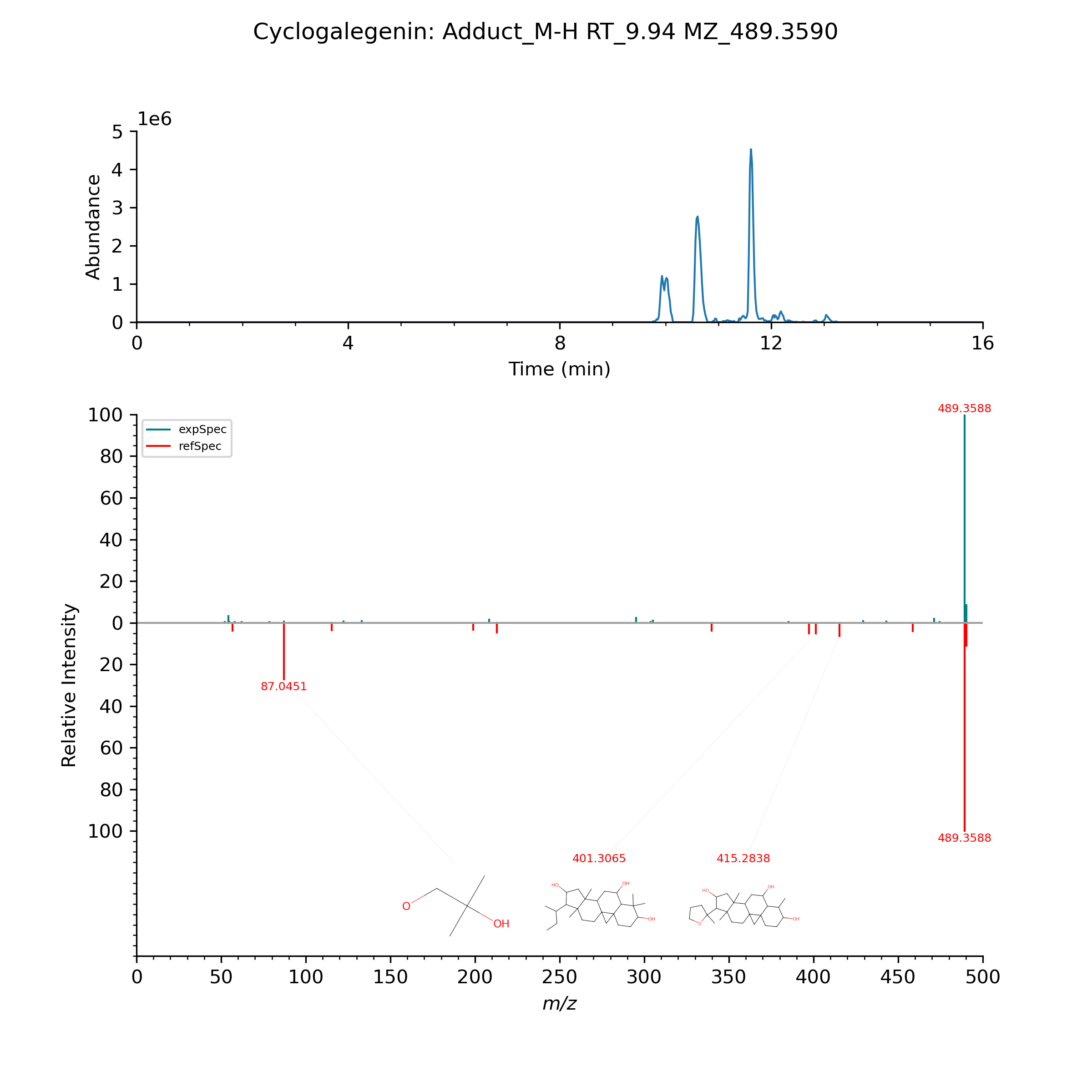

Supplement: Supplementary file 1 [file pharmaceuticals-18-01153-s001.zip › compound structures/M0199.png]

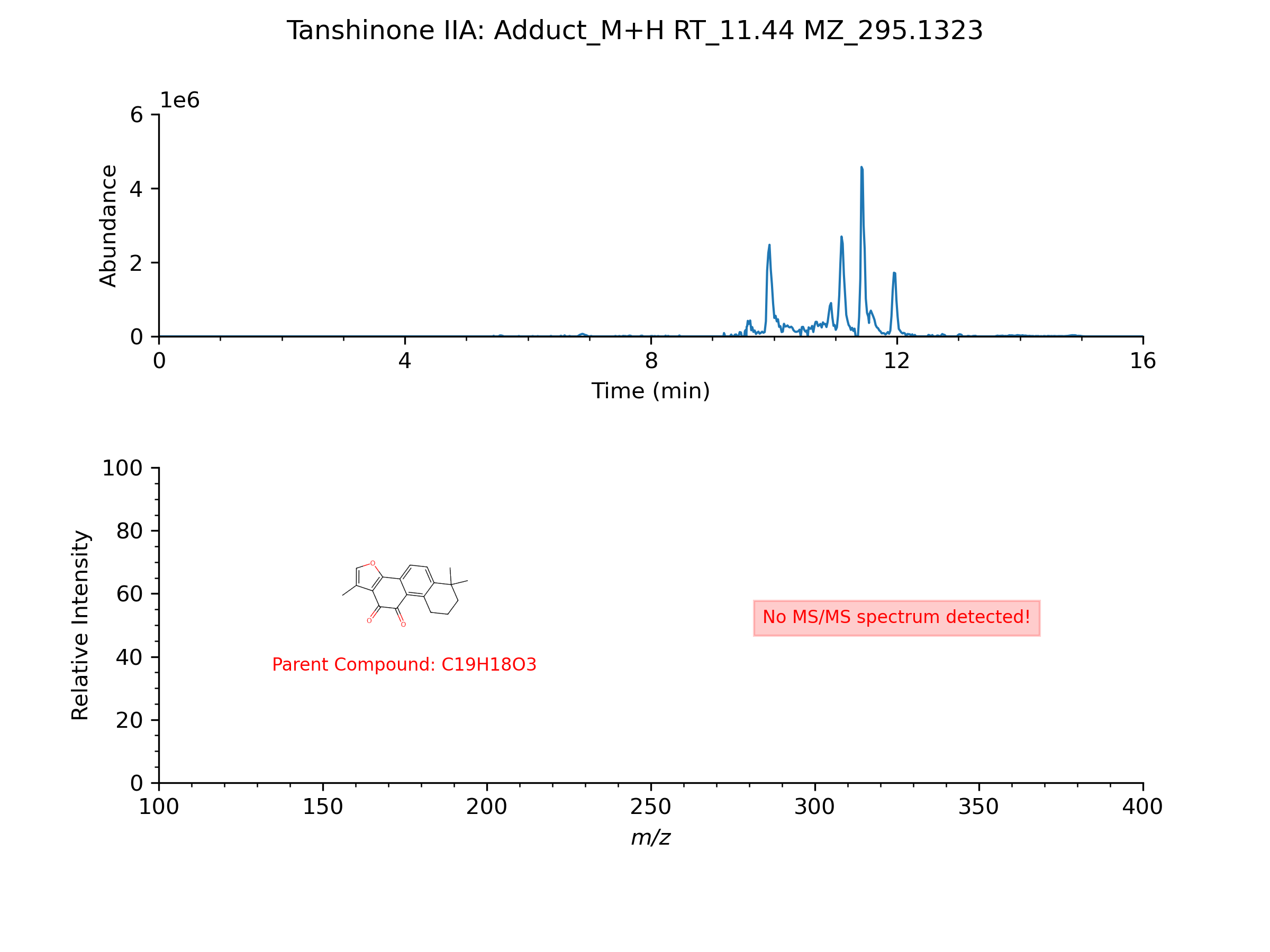

Supplement: Supplementary file 1 [file pharmaceuticals-18-01153-s001.zip › compound structures/M0200.png]
