# Supplementary figures and images for: Pharmacological Investigation of Tongqiao Jiuxin Oil Against High-Altitude Hypoxia: Integrating Chemical Profiling, Network Pharmacology, and Experimental Validation (part 1 of 2)
Source: Pharmaceuticals (Basel). 2025 Aug 2;18(8):1153. doi: 10.3390/ph18081153 (PMC12389502; doi:10.3390/ph18081153)

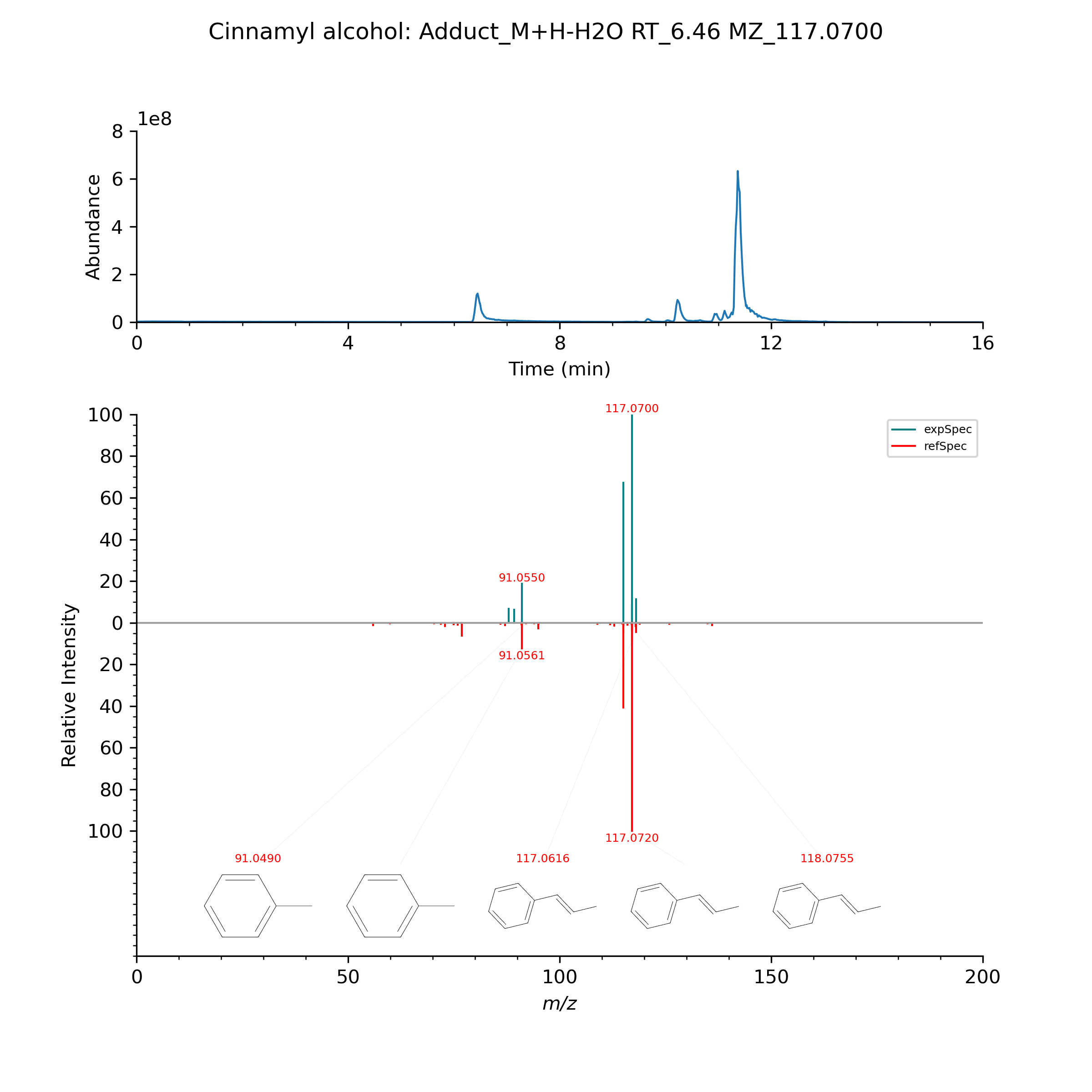

Supplement: Supplementary file 1 [file pharmaceuticals-18-01153-s001.zip › compound structures/M0001.png]

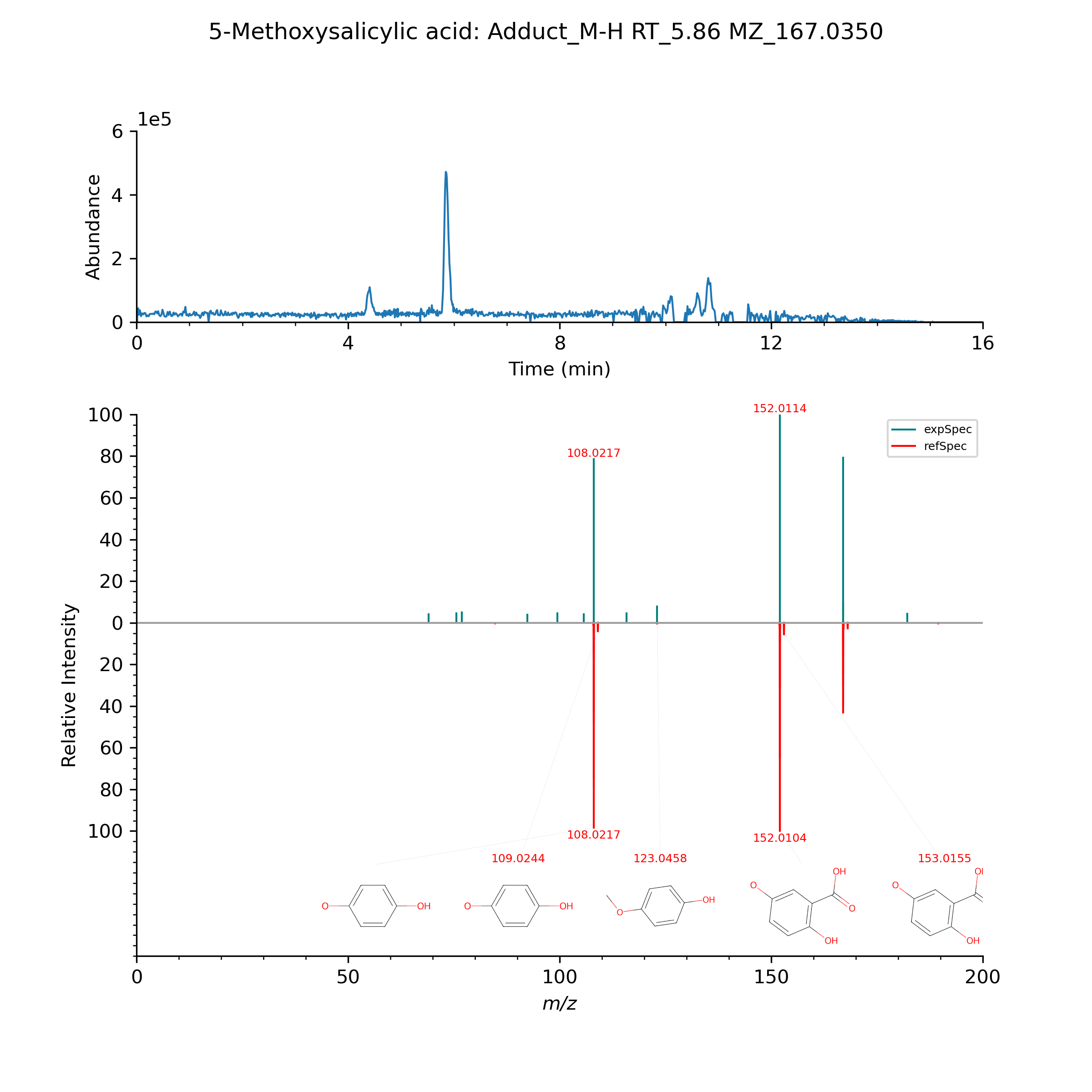

Supplement: Supplementary file 1 [file pharmaceuticals-18-01153-s001.zip › compound structures/M0002.png]

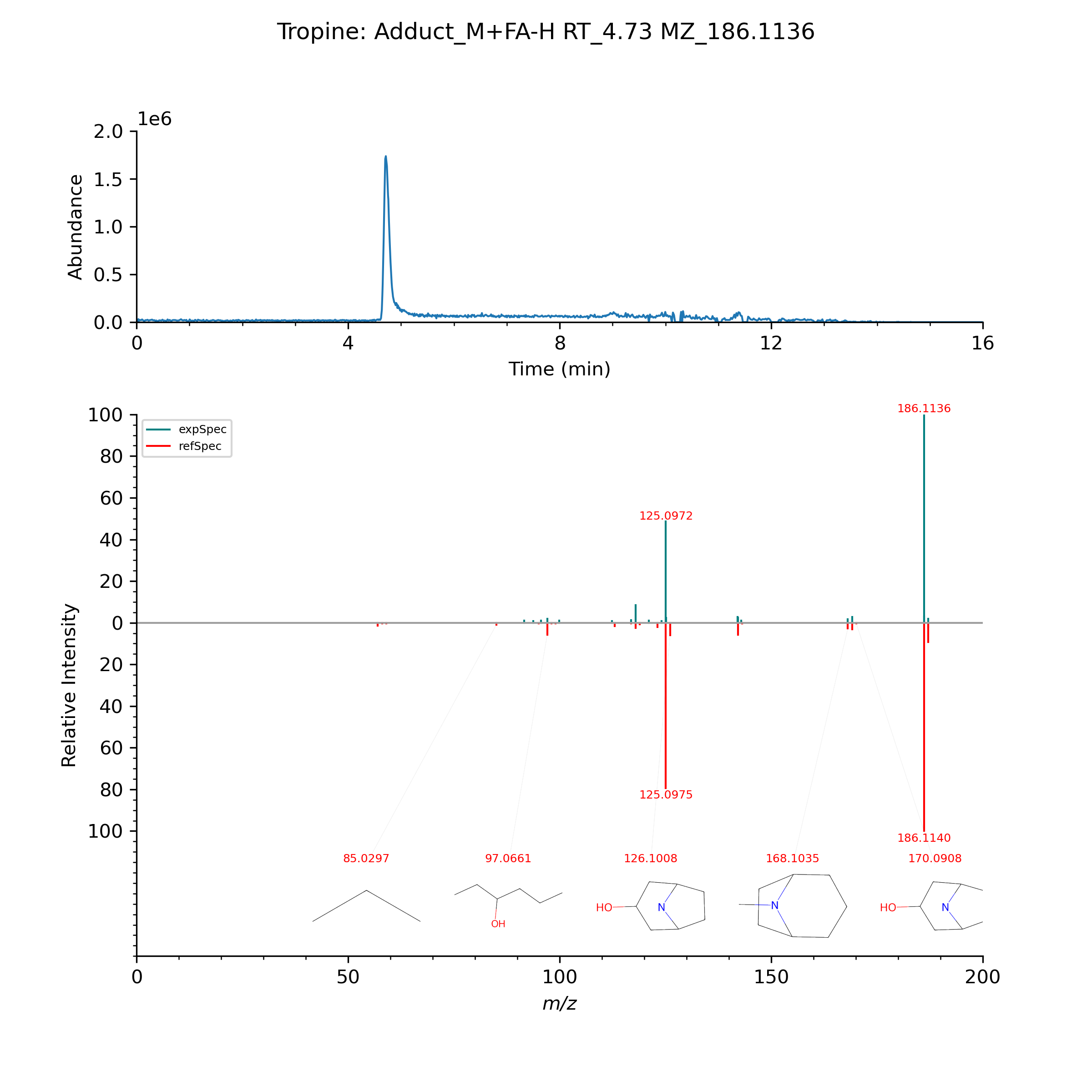

Supplement: Supplementary file 1 [file pharmaceuticals-18-01153-s001.zip › compound structures/M0003.png]

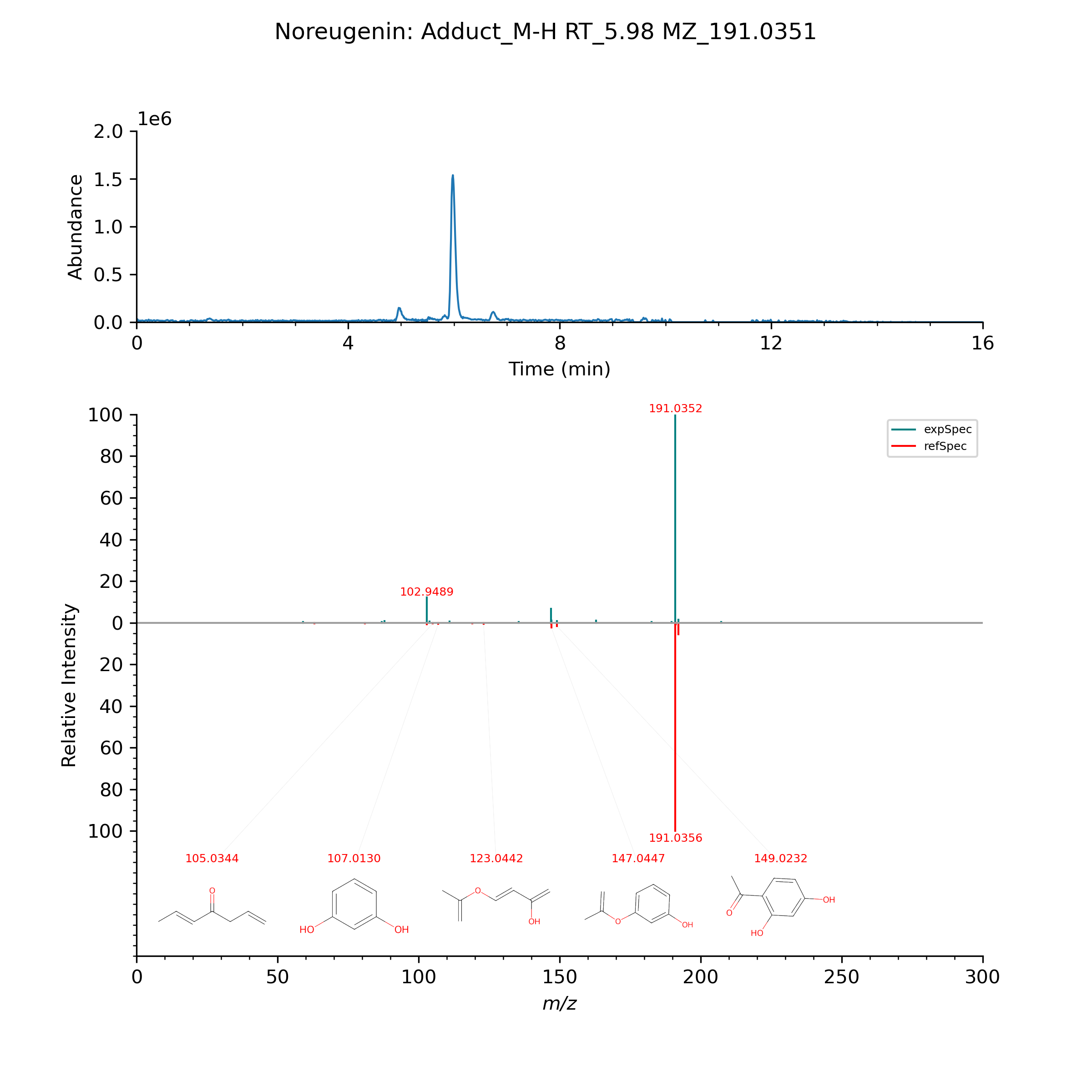

Supplement: Supplementary file 1 [file pharmaceuticals-18-01153-s001.zip › compound structures/M0004.png]

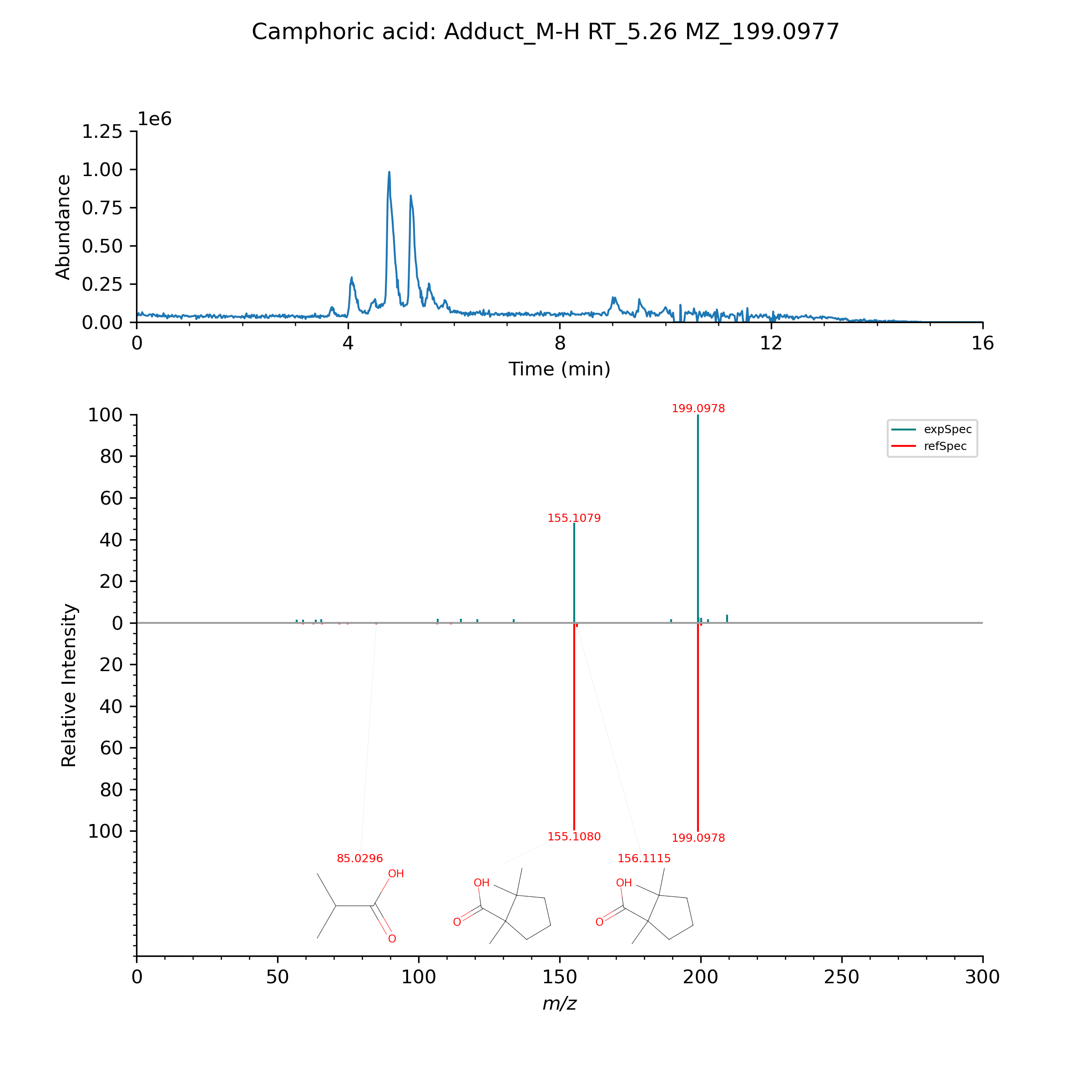

Supplement: Supplementary file 1 [file pharmaceuticals-18-01153-s001.zip › compound structures/M0005.png]

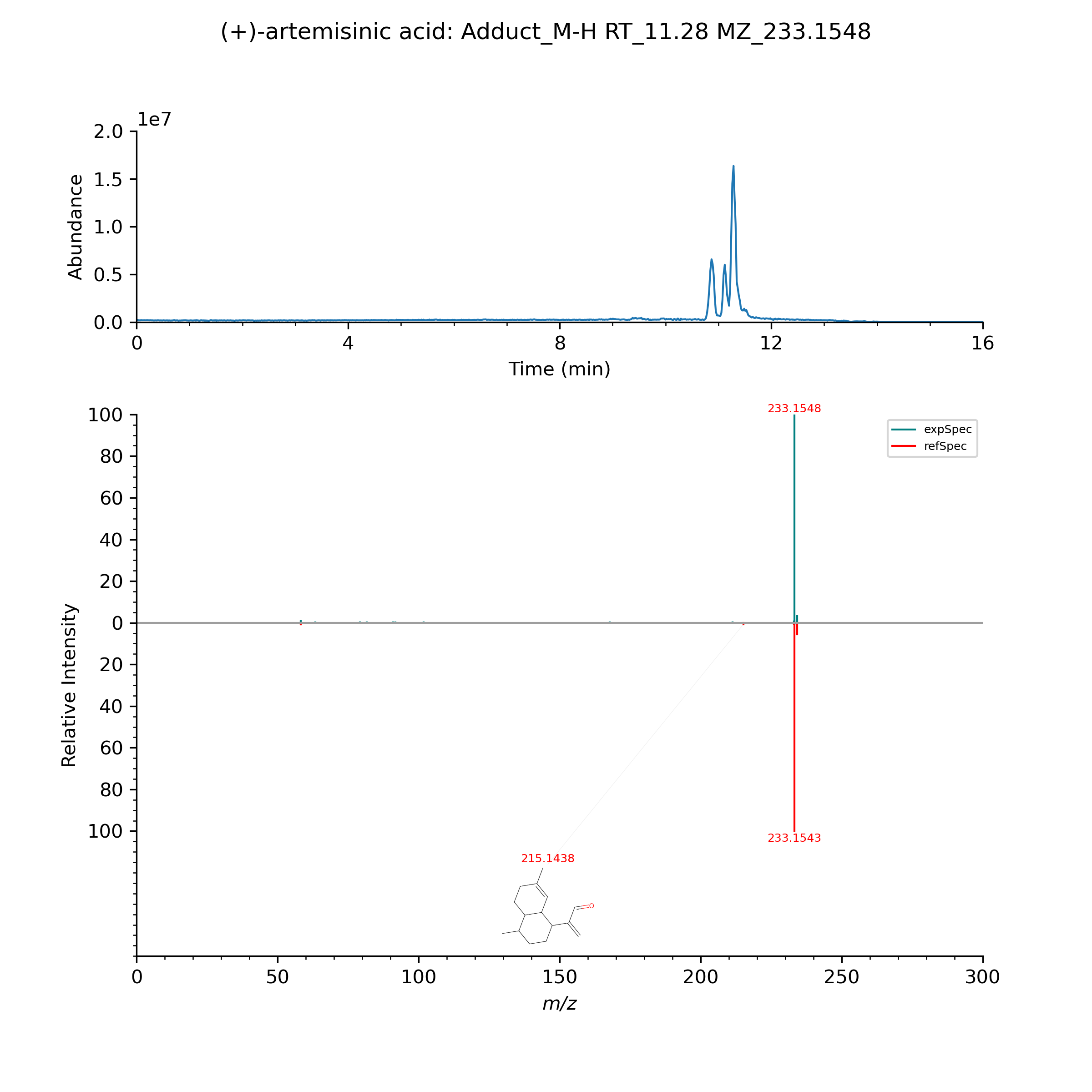

Supplement: Supplementary file 1 [file pharmaceuticals-18-01153-s001.zip › compound structures/M0006.png]

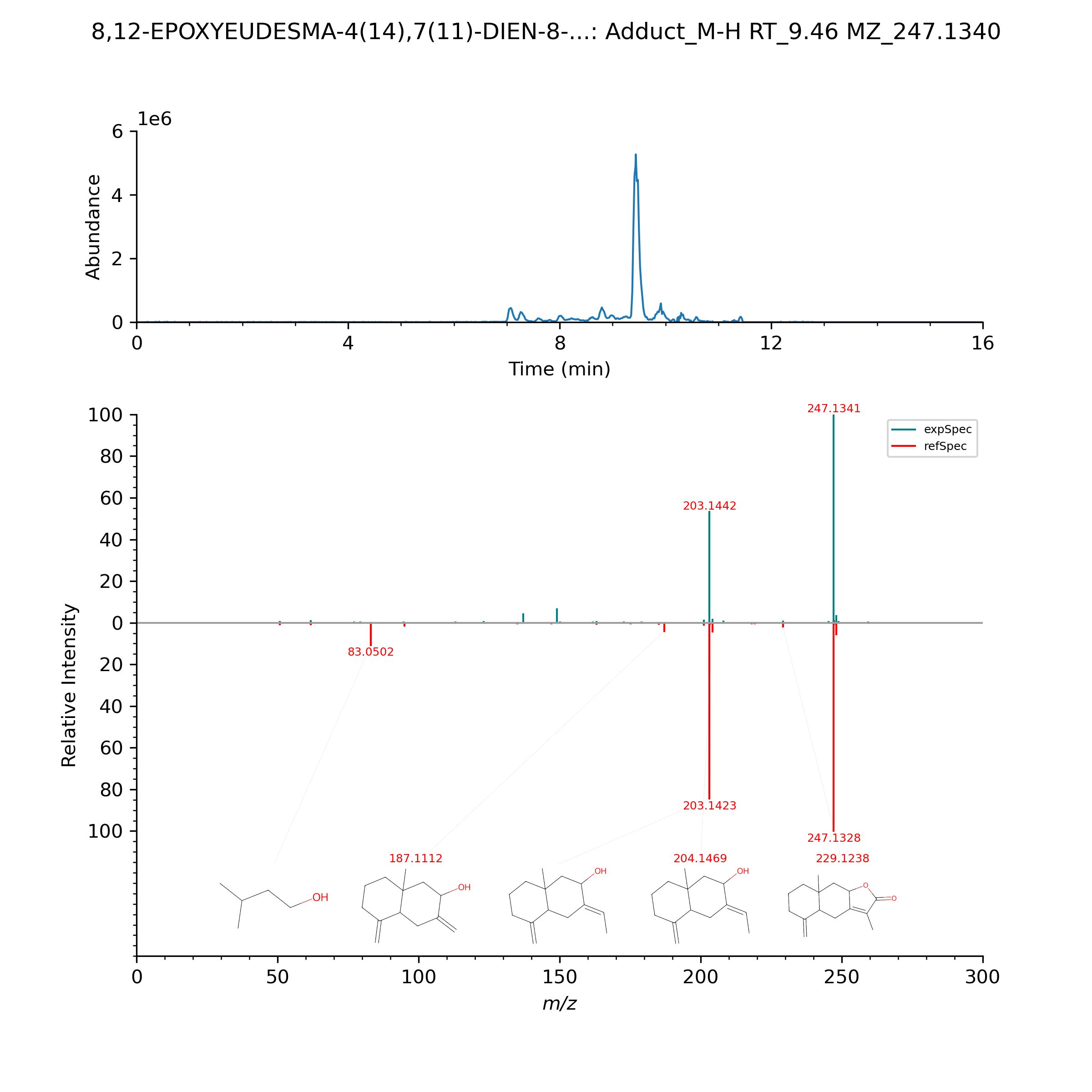

Supplement: Supplementary file 1 [file pharmaceuticals-18-01153-s001.zip › compound structures/M0007.png]

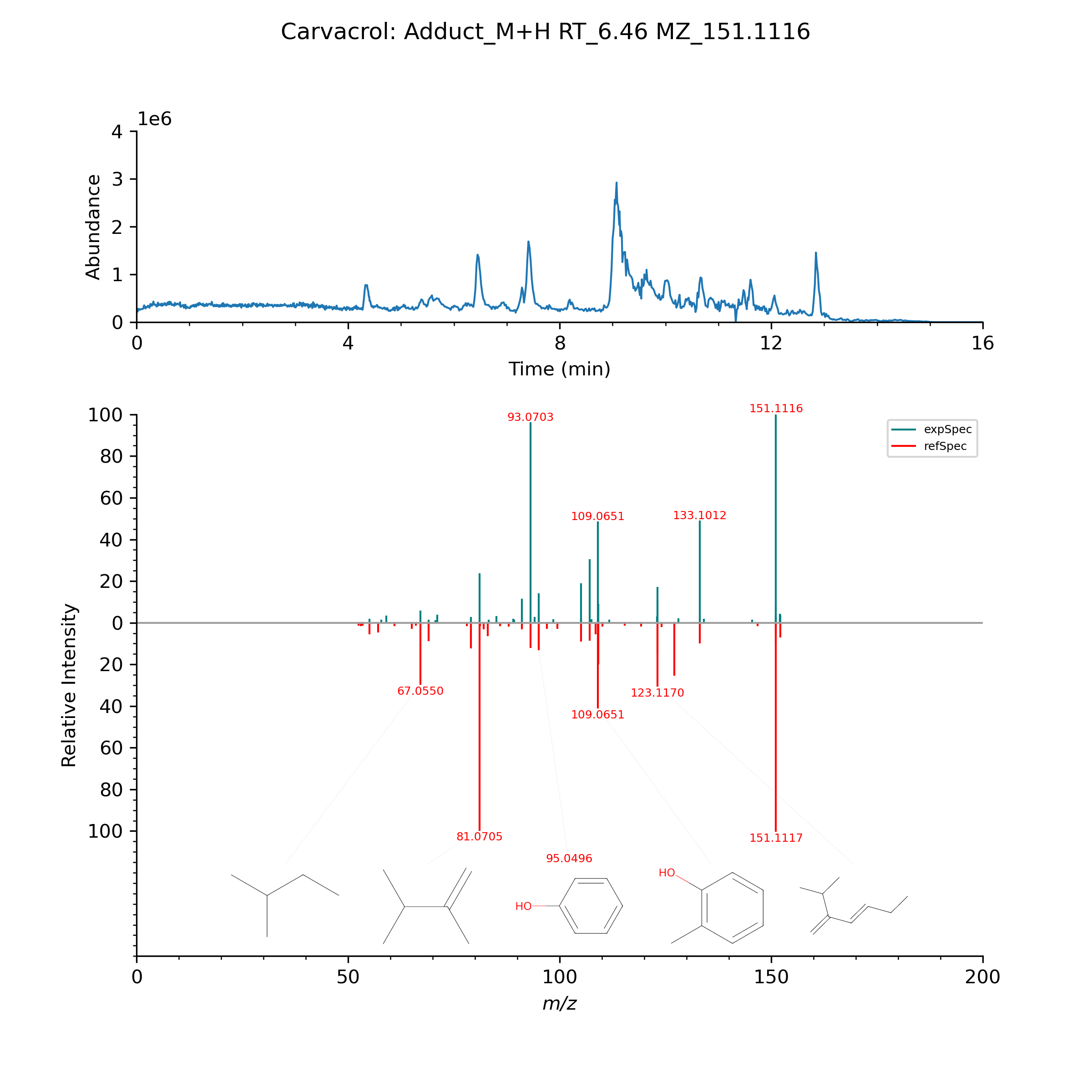

Supplement: Supplementary file 1 [file pharmaceuticals-18-01153-s001.zip › compound structures/M0008.png]

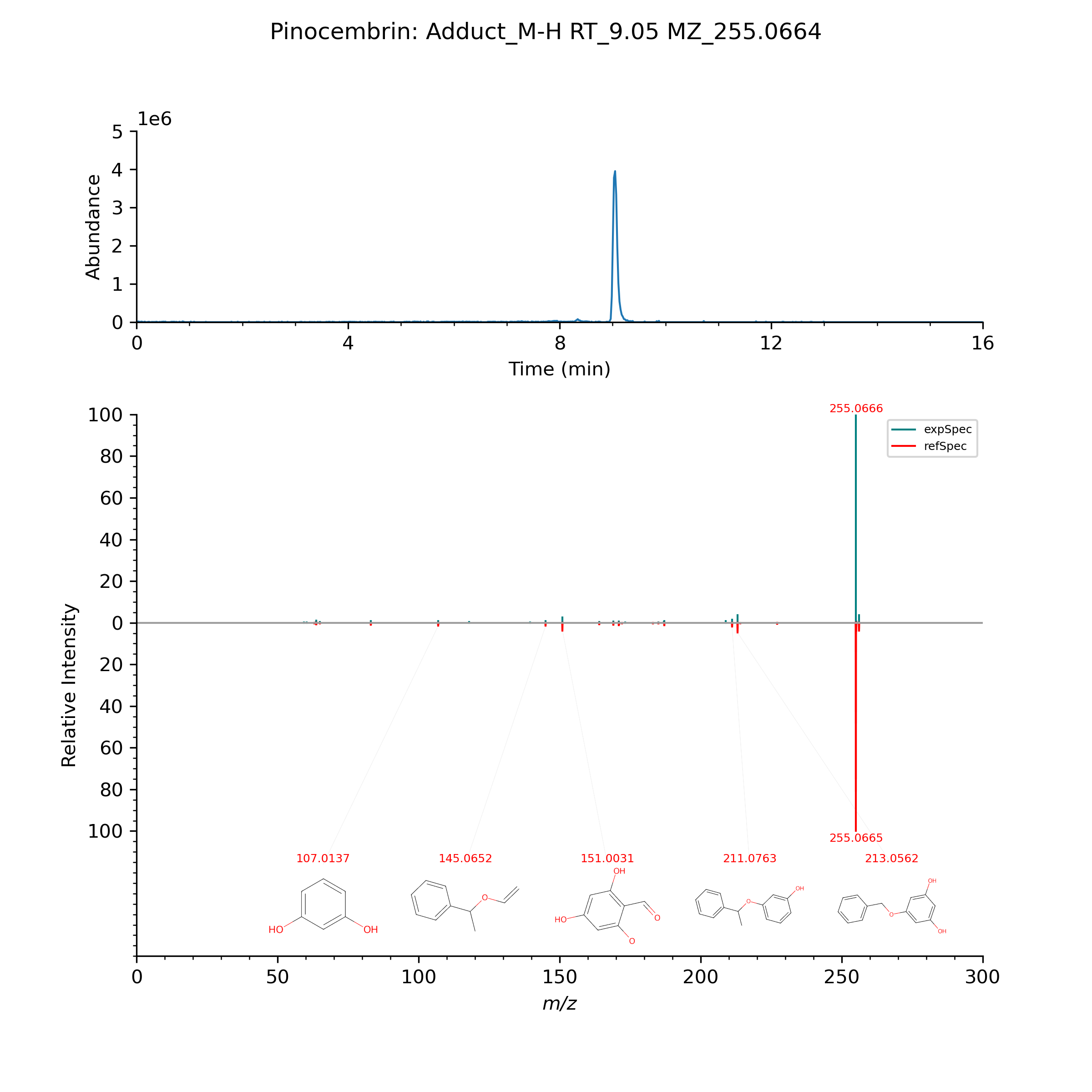

Supplement: Supplementary file 1 [file pharmaceuticals-18-01153-s001.zip › compound structures/M0009.png]

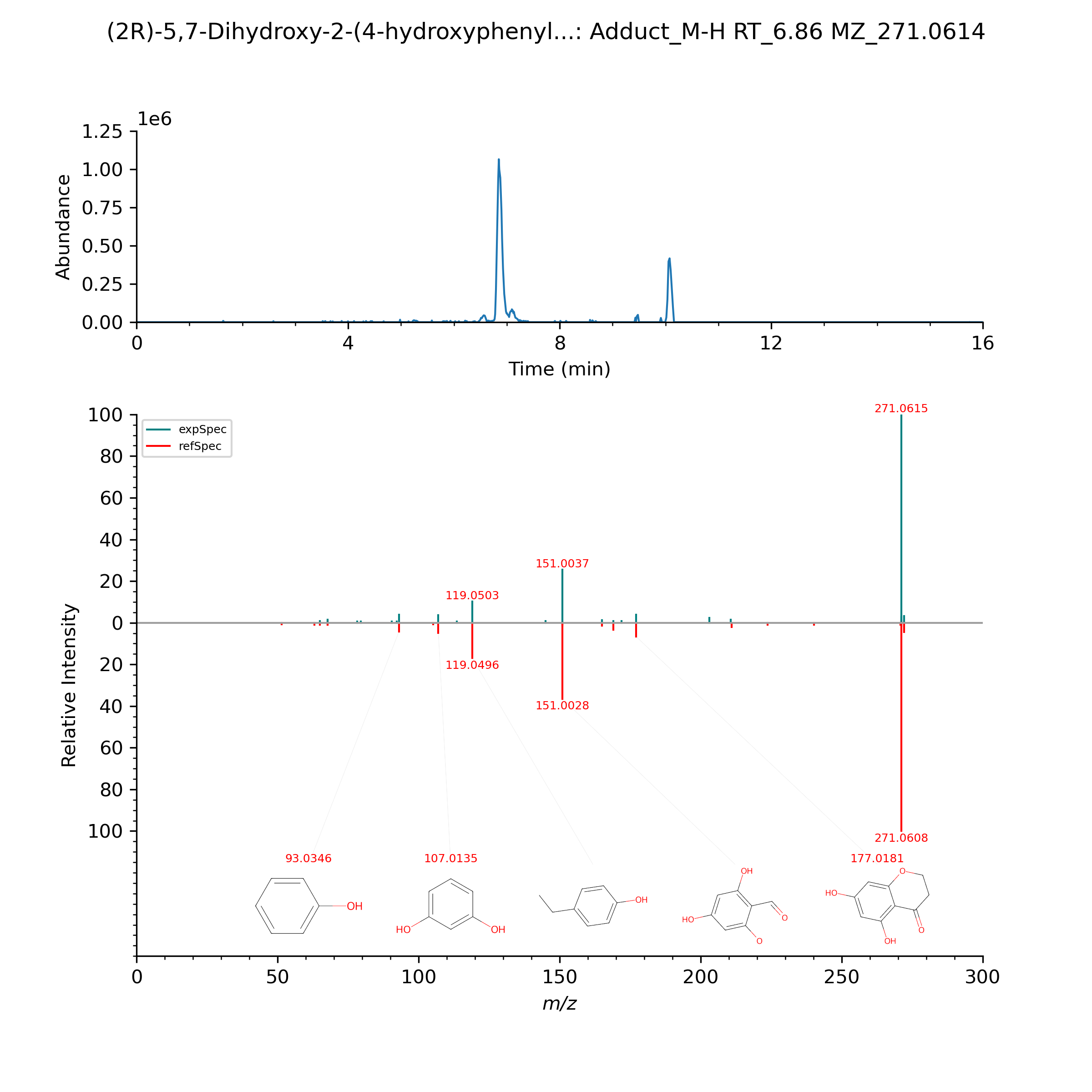

Supplement: Supplementary file 1 [file pharmaceuticals-18-01153-s001.zip › compound structures/M0010.png]

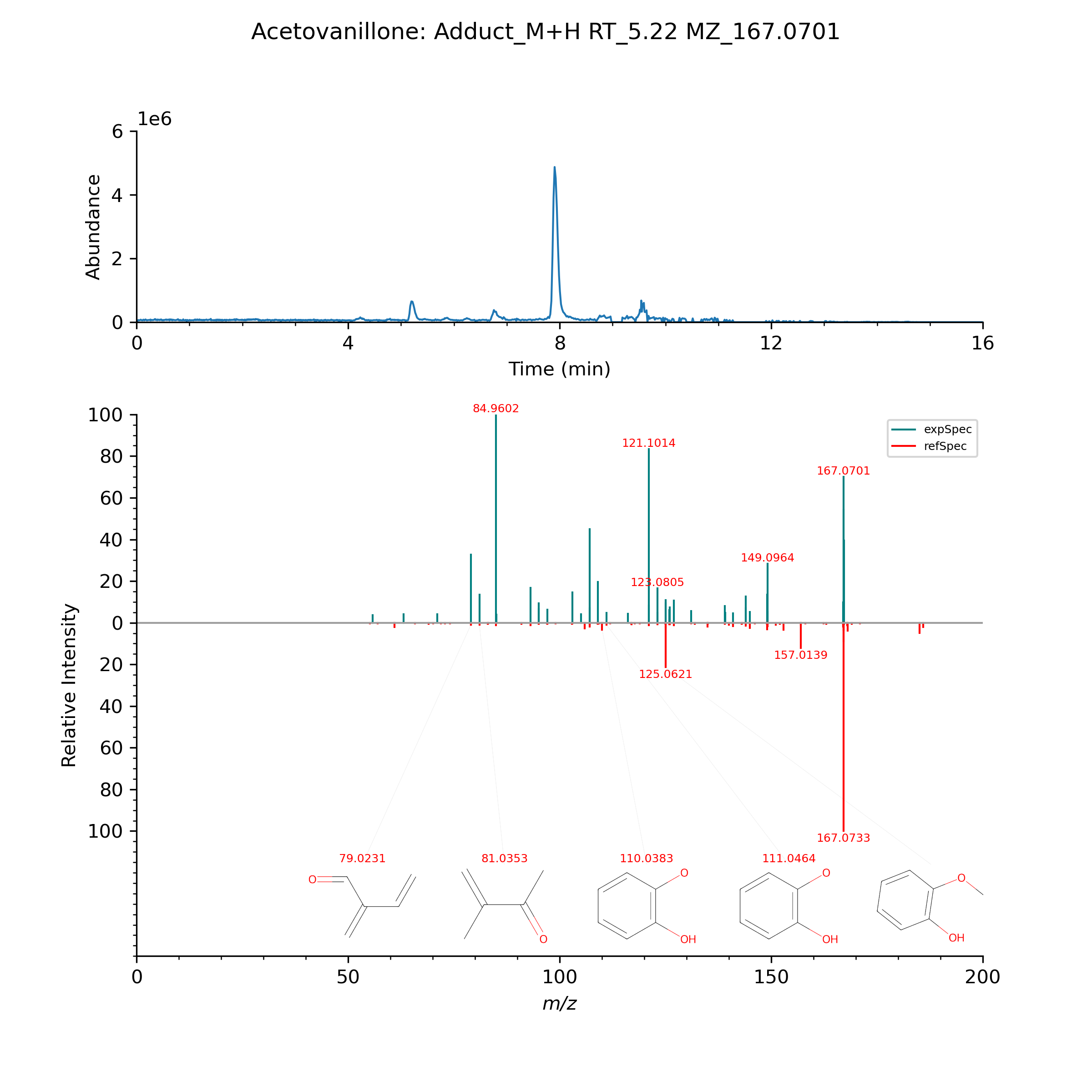

Supplement: Supplementary file 1 [file pharmaceuticals-18-01153-s001.zip › compound structures/M0011.png]

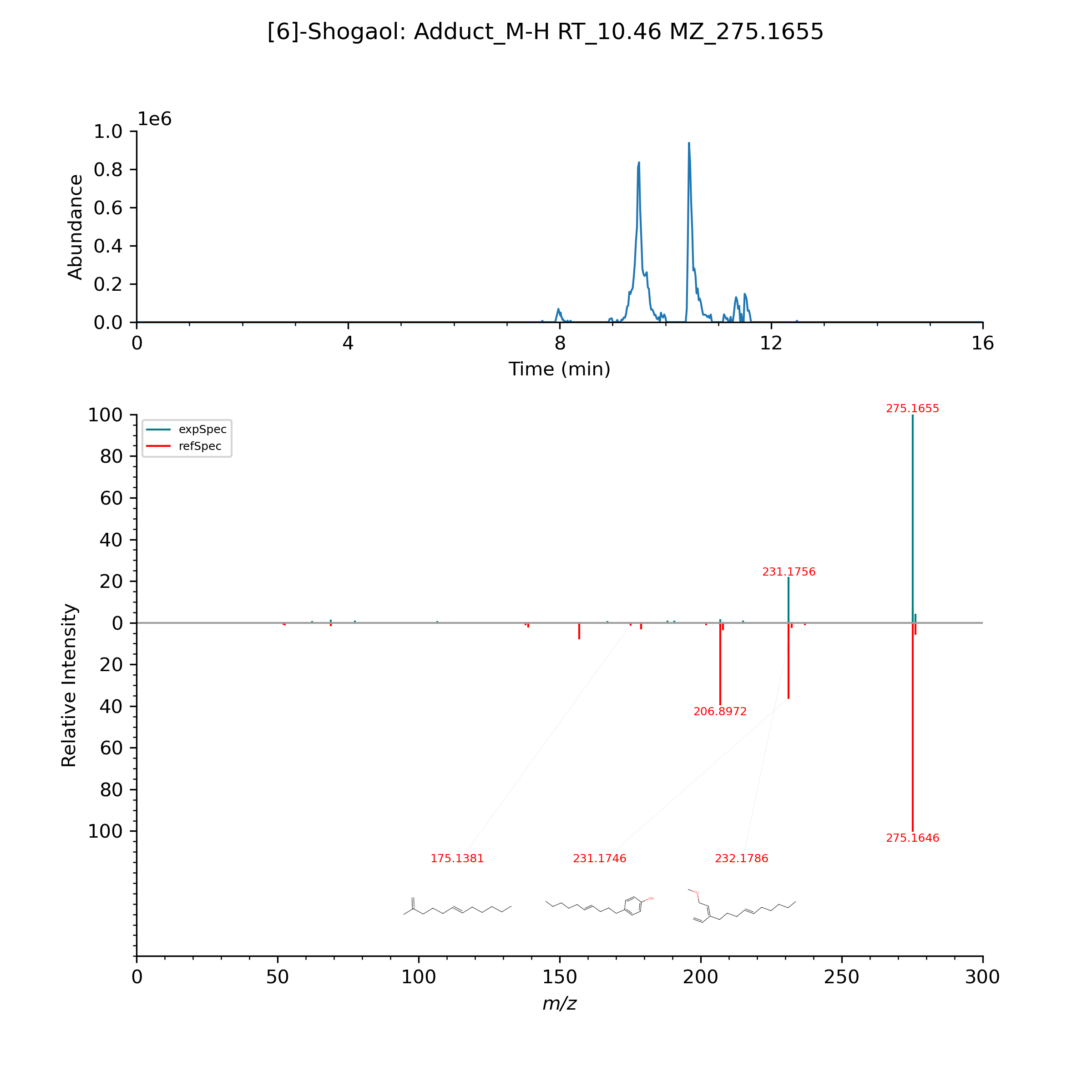

Supplement: Supplementary file 1 [file pharmaceuticals-18-01153-s001.zip › compound structures/M0012.png]

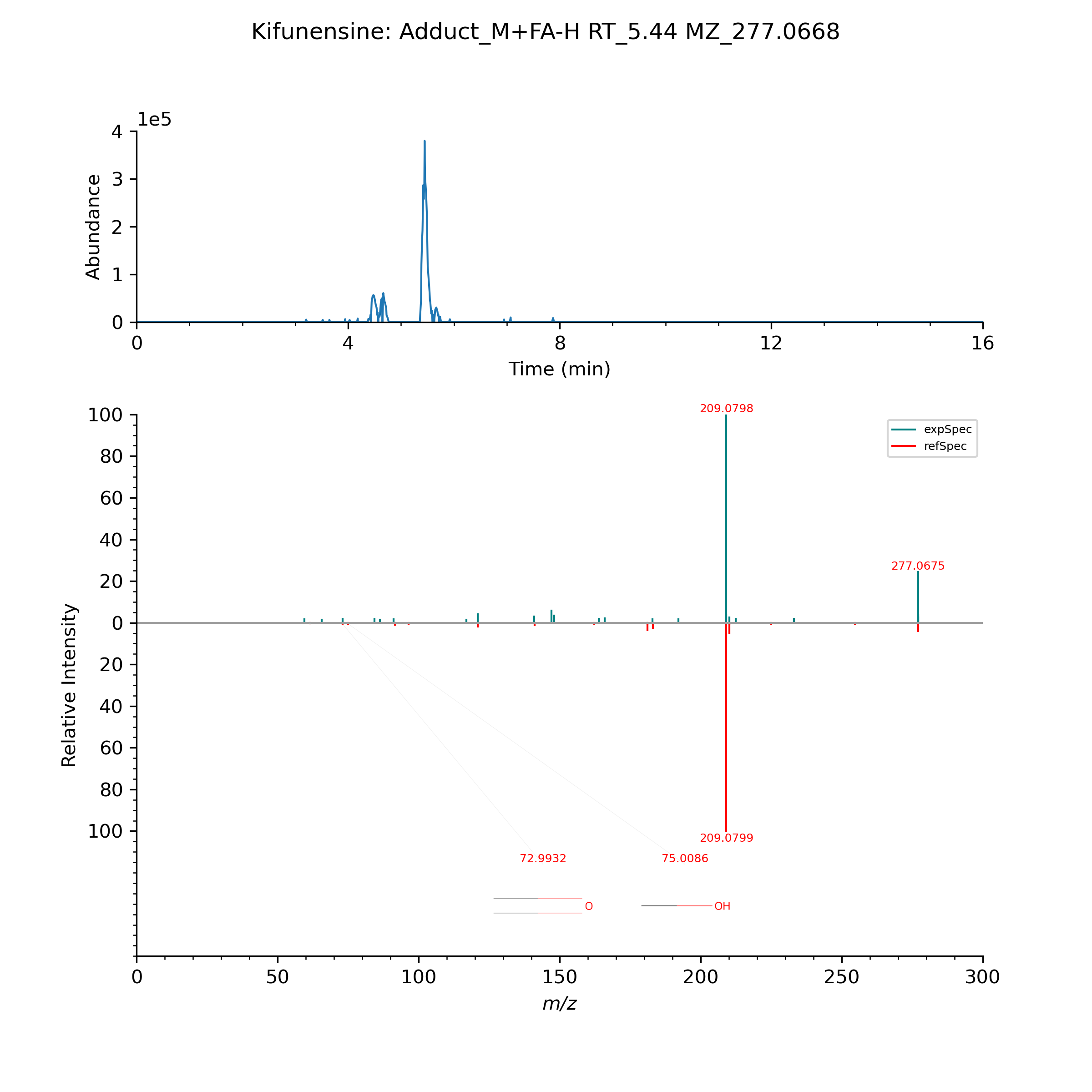

Supplement: Supplementary file 1 [file pharmaceuticals-18-01153-s001.zip › compound structures/M0013.png]

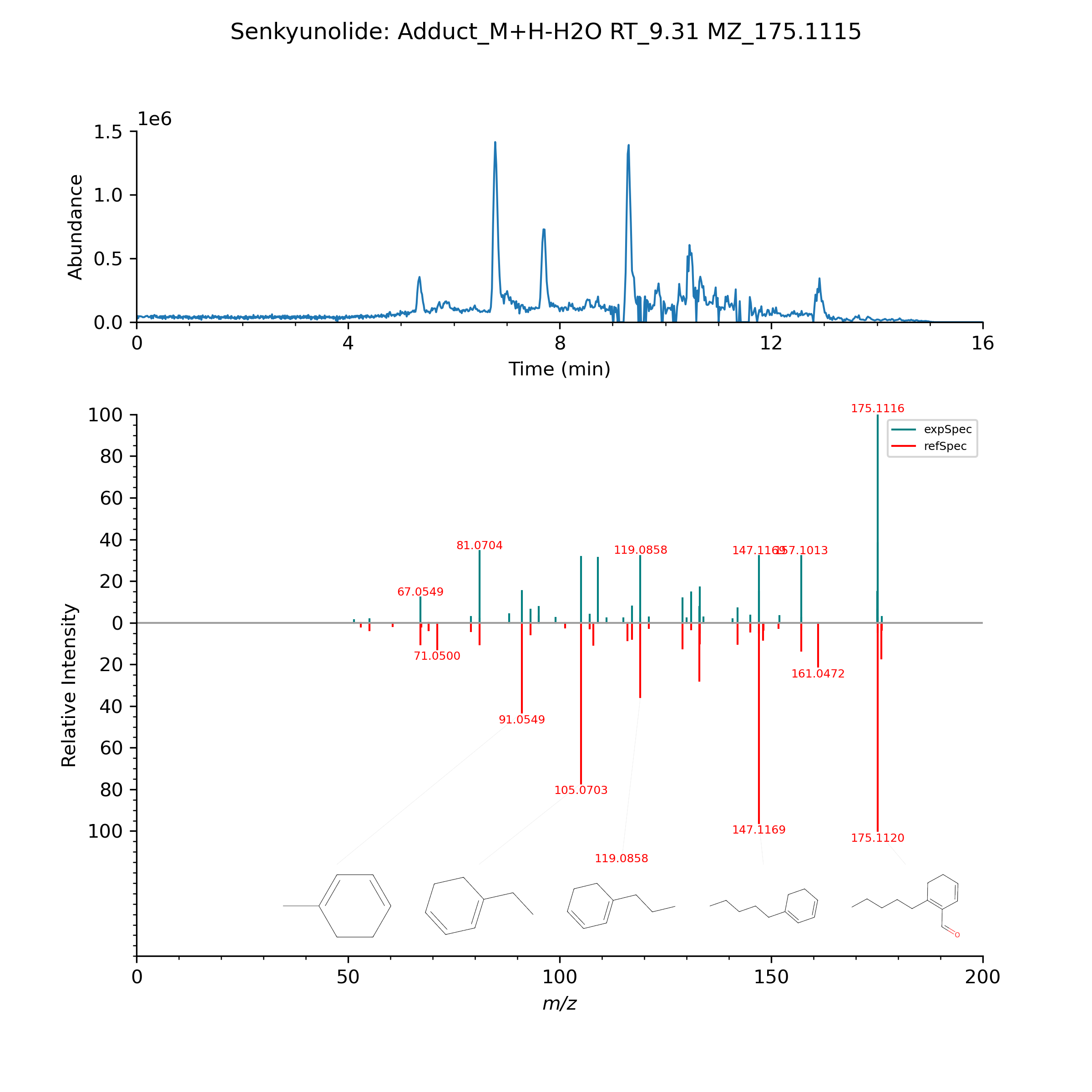

Supplement: Supplementary file 1 [file pharmaceuticals-18-01153-s001.zip › compound structures/M0014.png]

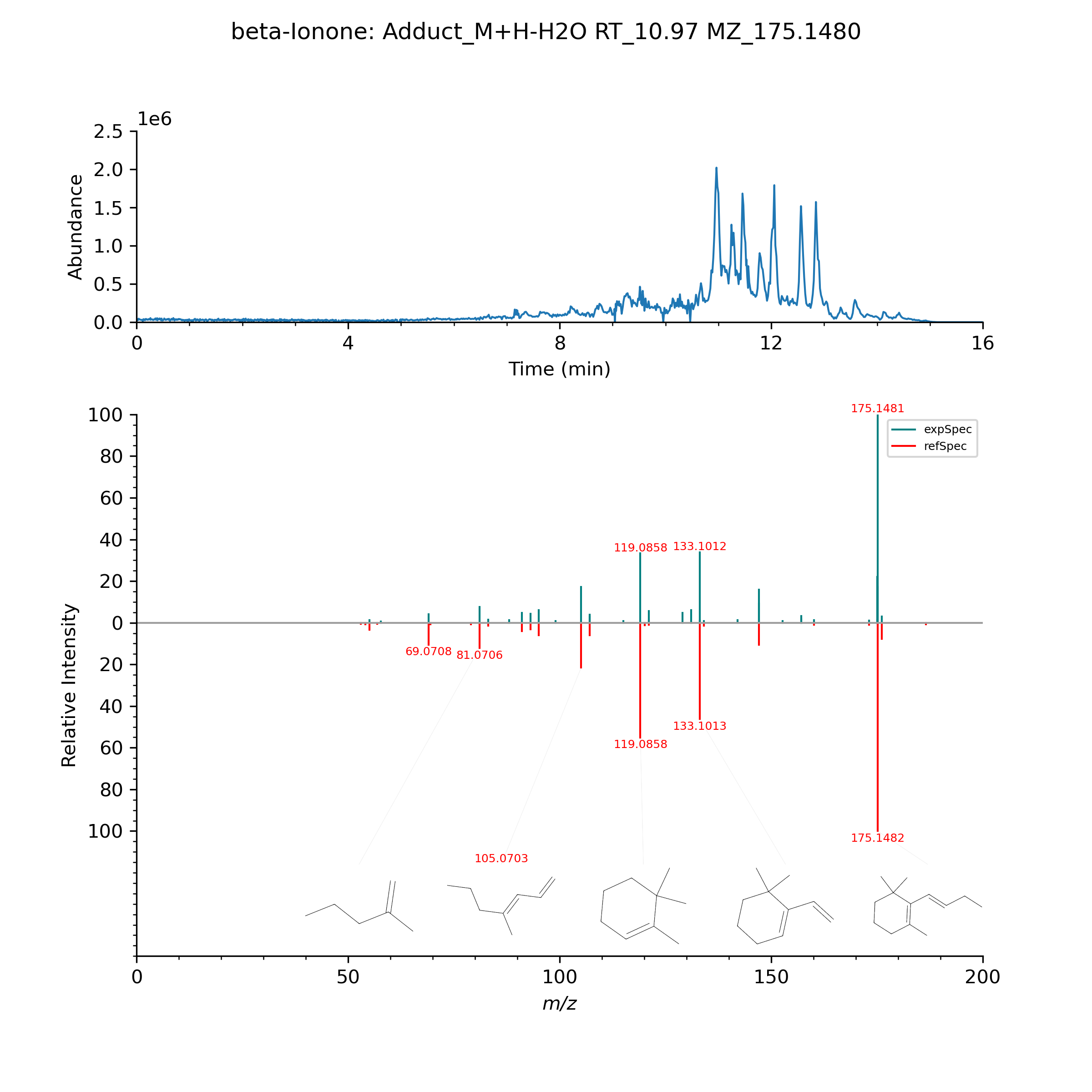

Supplement: Supplementary file 1 [file pharmaceuticals-18-01153-s001.zip › compound structures/M0015.png]

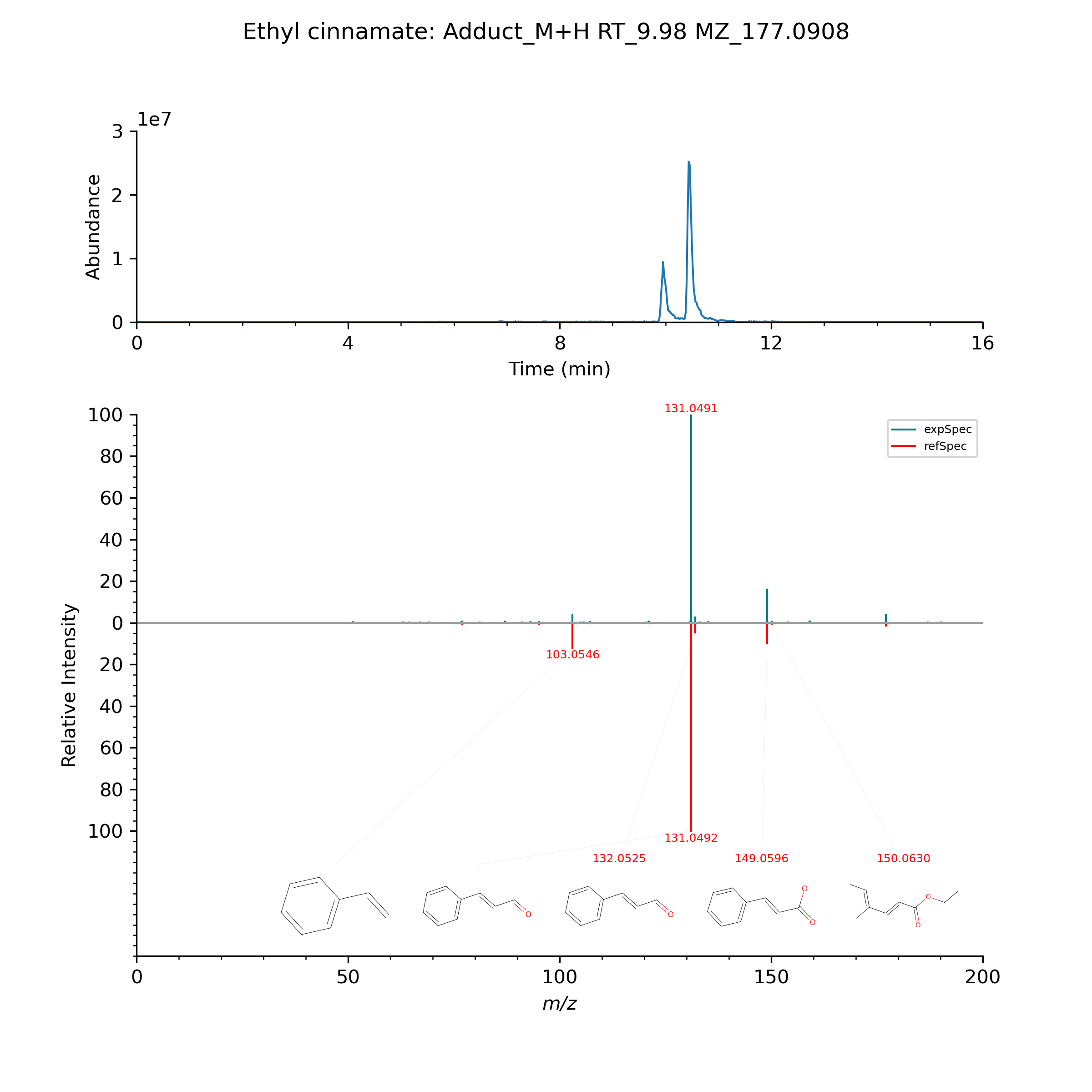

Supplement: Supplementary file 1 [file pharmaceuticals-18-01153-s001.zip › compound structures/M0016.png]

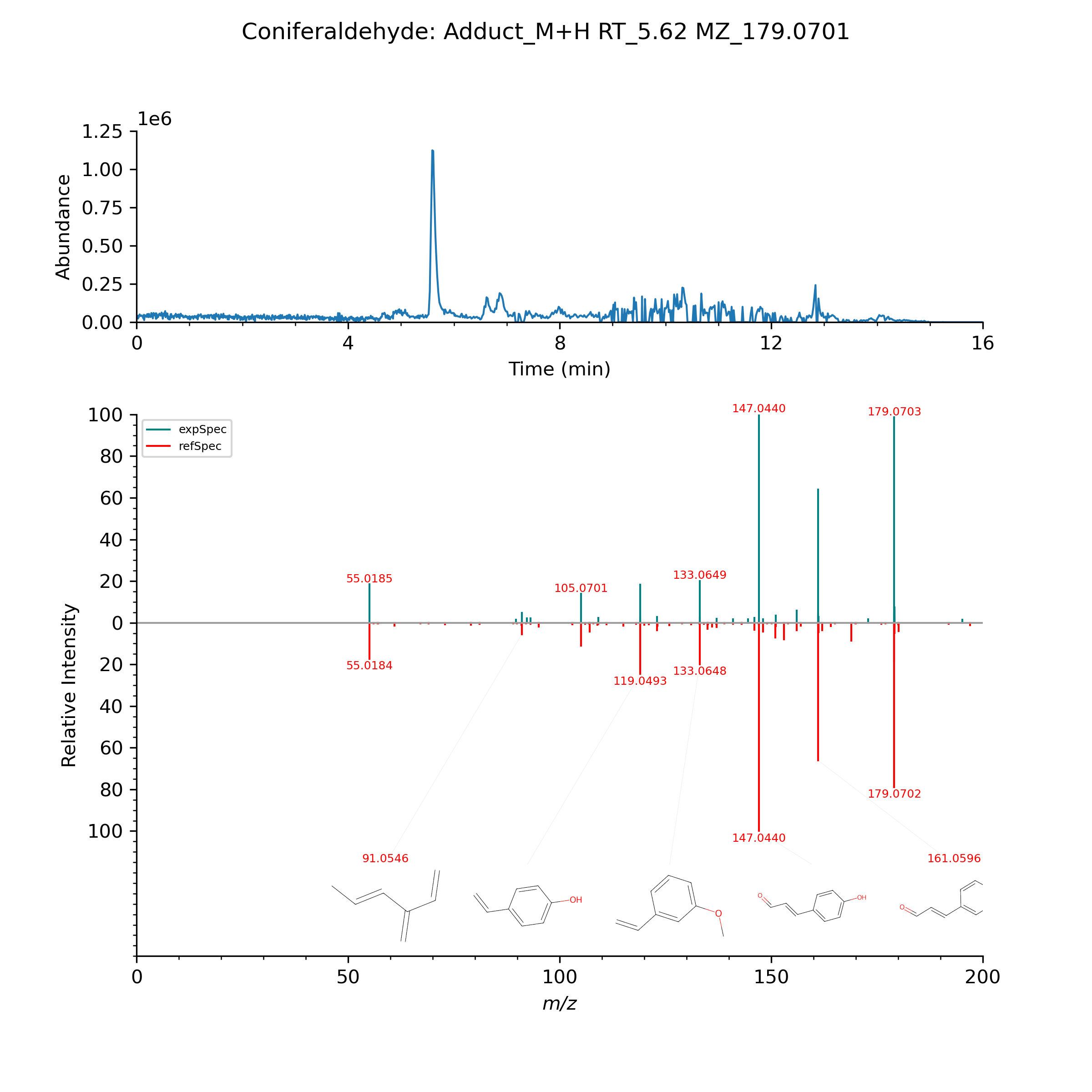

Supplement: Supplementary file 1 [file pharmaceuticals-18-01153-s001.zip › compound structures/M0017.png]

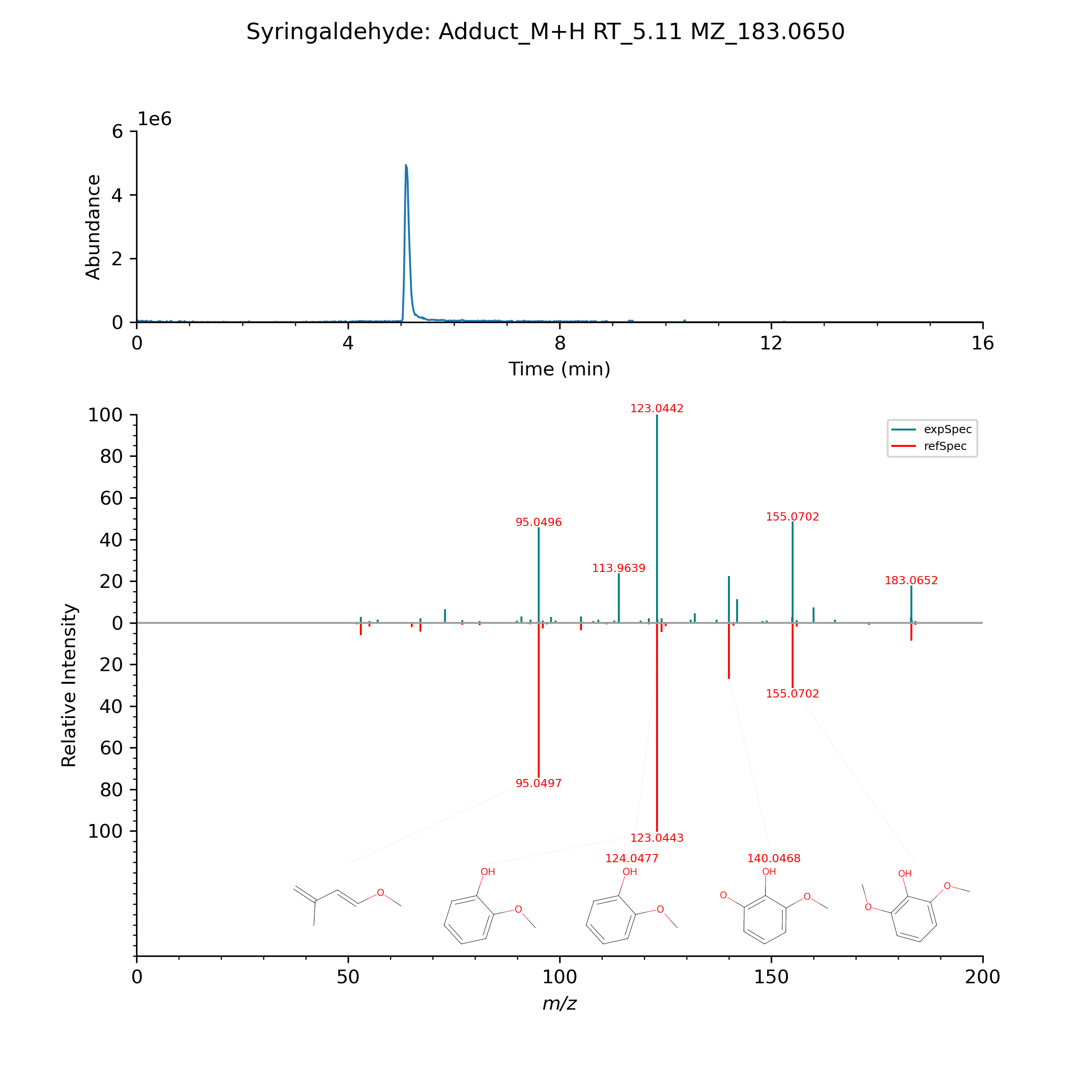

Supplement: Supplementary file 1 [file pharmaceuticals-18-01153-s001.zip › compound structures/M0018.png]

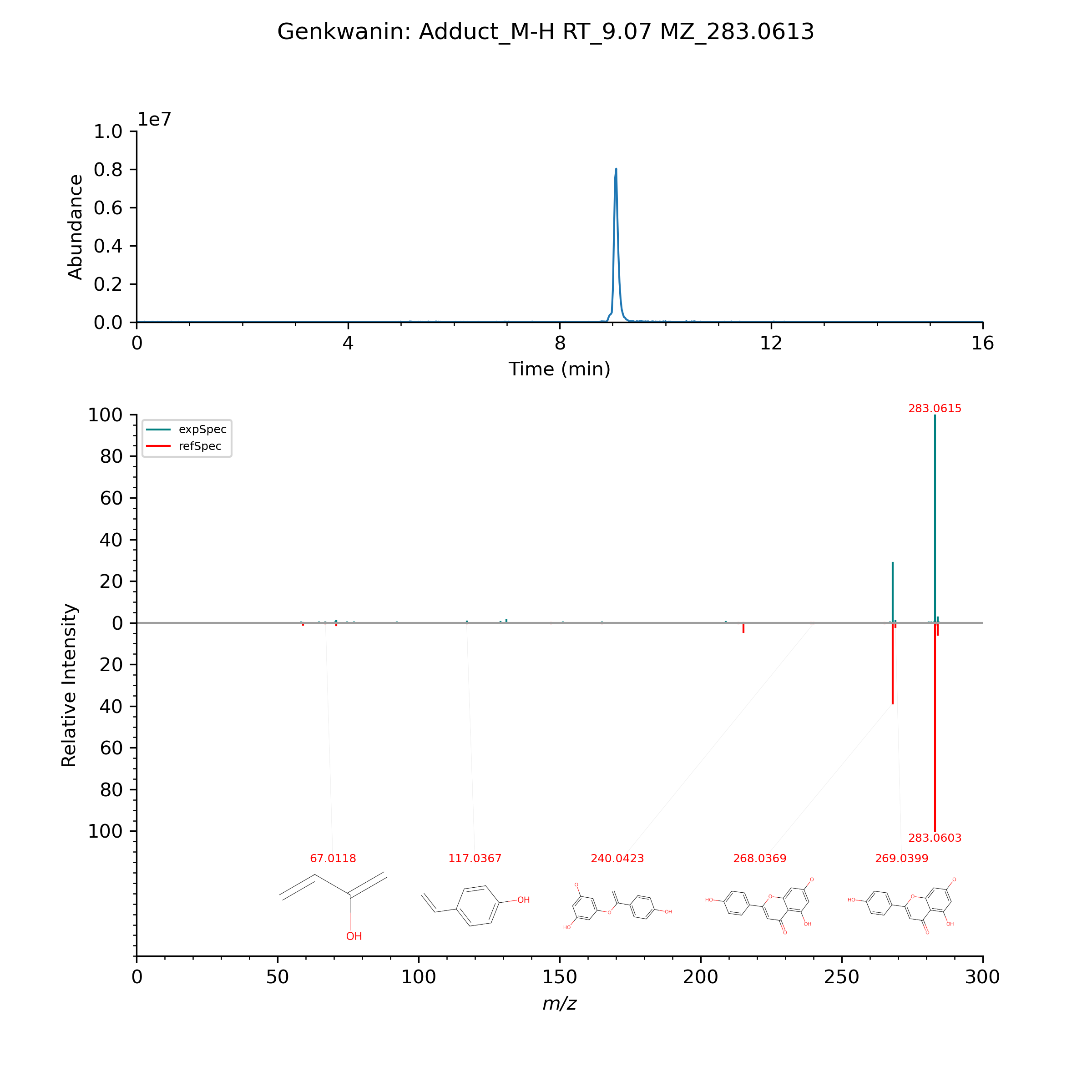

Supplement: Supplementary file 1 [file pharmaceuticals-18-01153-s001.zip › compound structures/M0019.png]

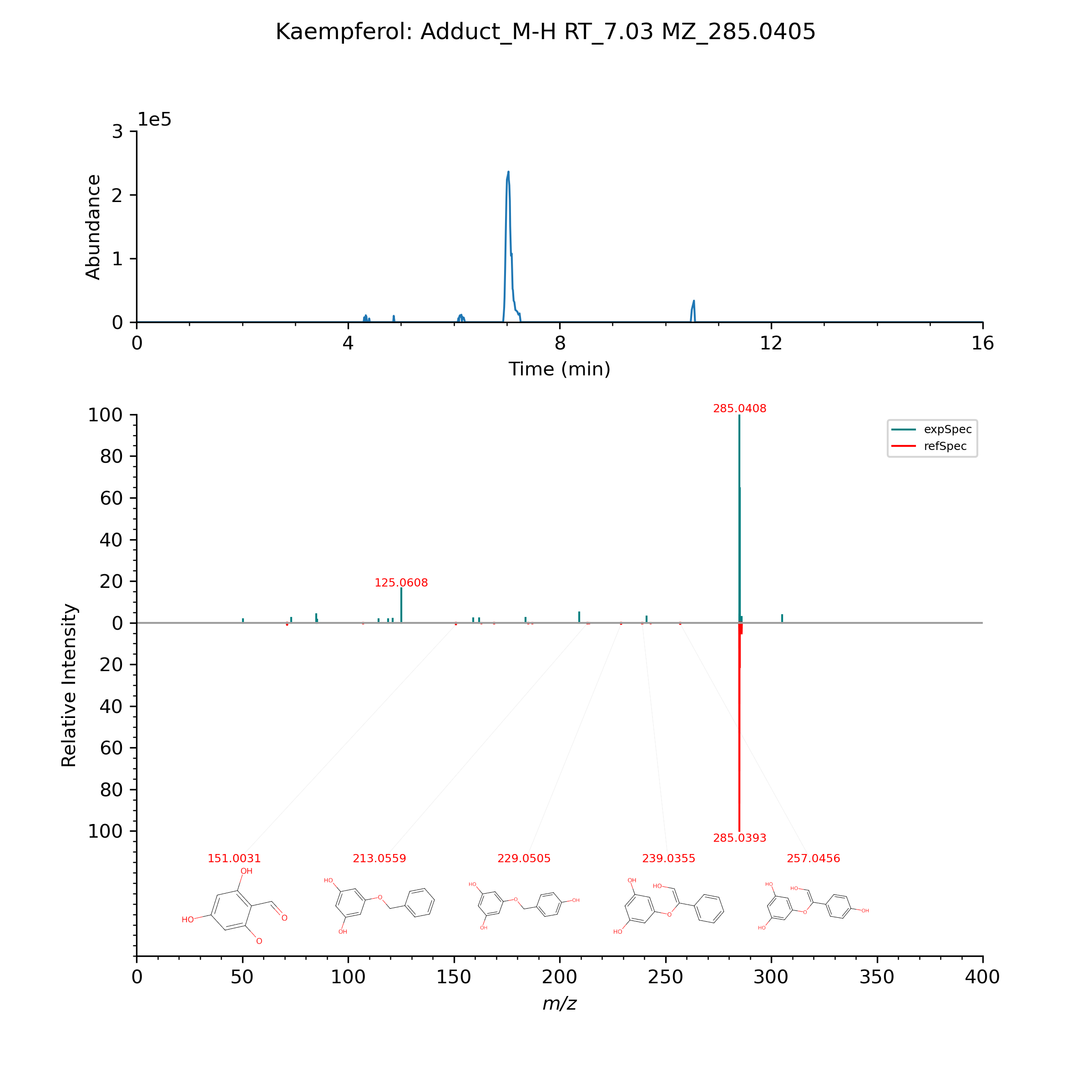

Supplement: Supplementary file 1 [file pharmaceuticals-18-01153-s001.zip › compound structures/M0020.png]

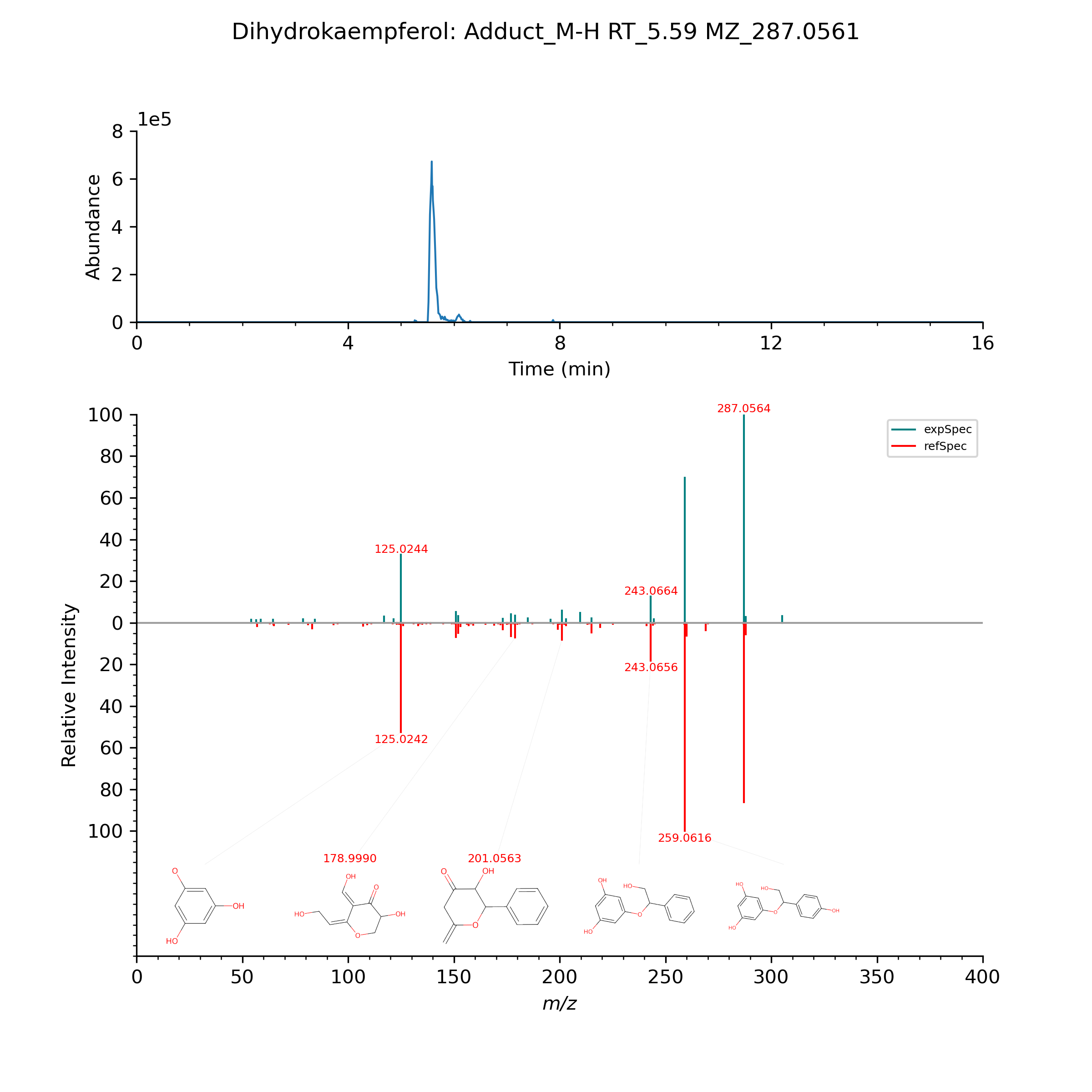

Supplement: Supplementary file 1 [file pharmaceuticals-18-01153-s001.zip › compound structures/M0021.png]

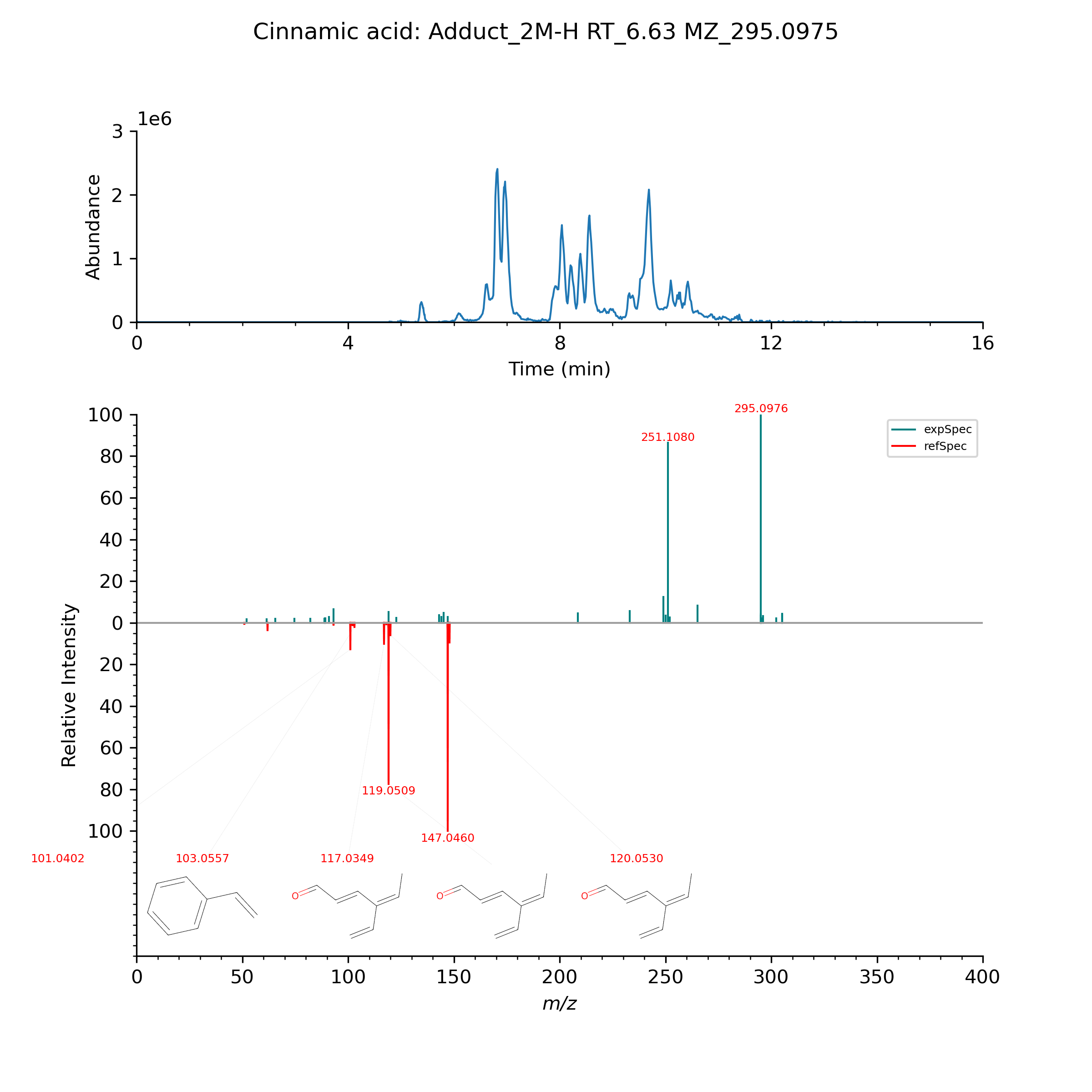

Supplement: Supplementary file 1 [file pharmaceuticals-18-01153-s001.zip › compound structures/M0022.png]

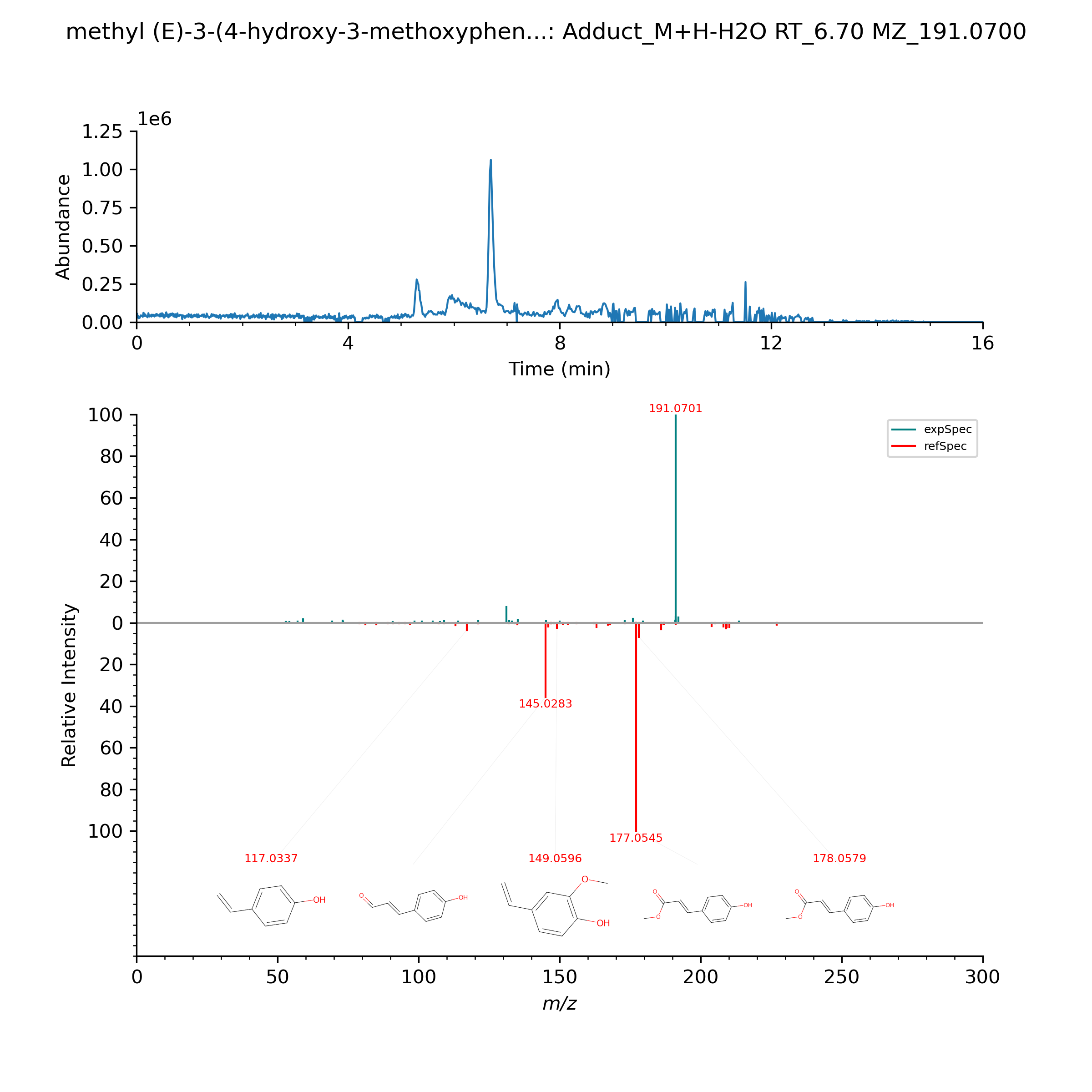

Supplement: Supplementary file 1 [file pharmaceuticals-18-01153-s001.zip › compound structures/M0023.png]

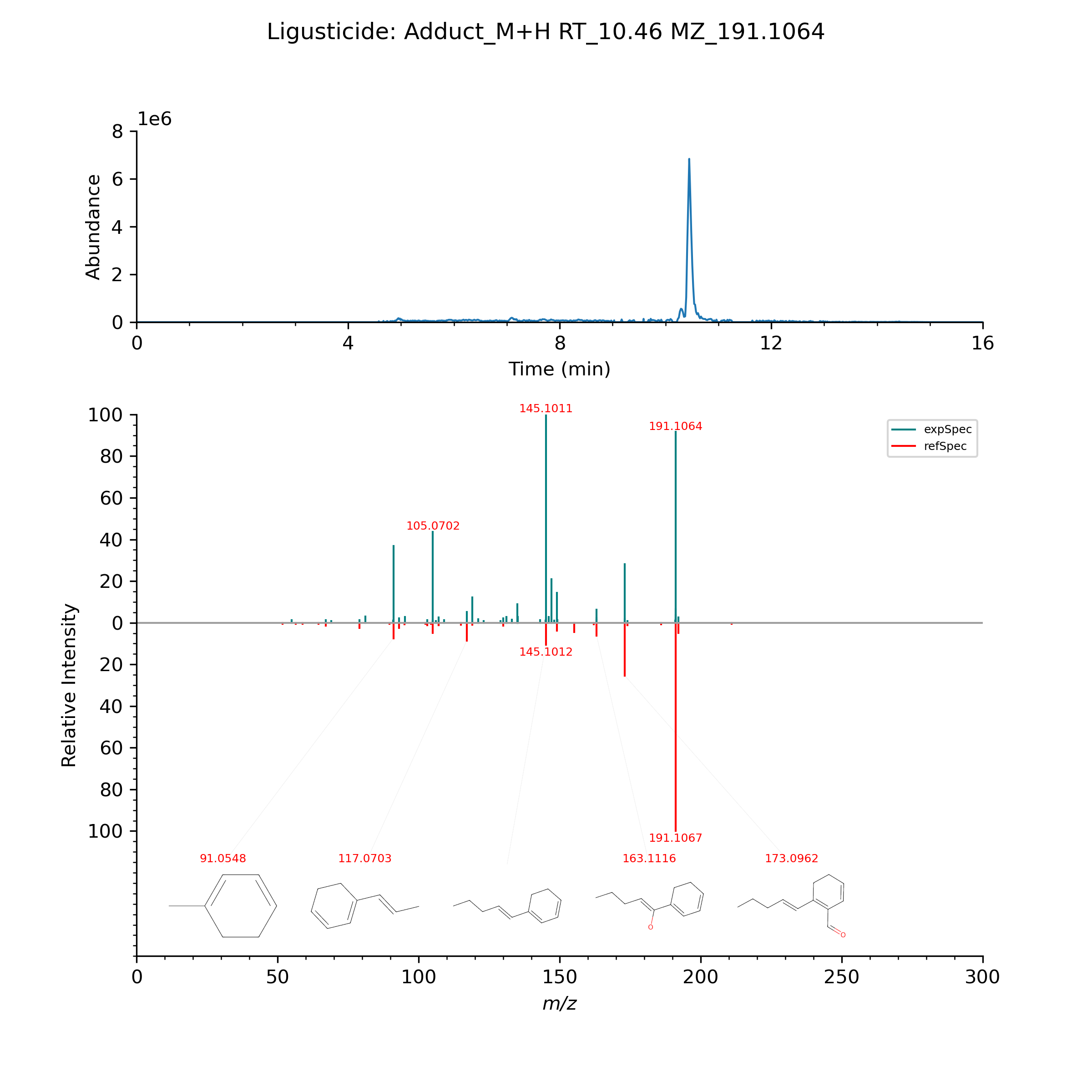

Supplement: Supplementary file 1 [file pharmaceuticals-18-01153-s001.zip › compound structures/M0024.png]

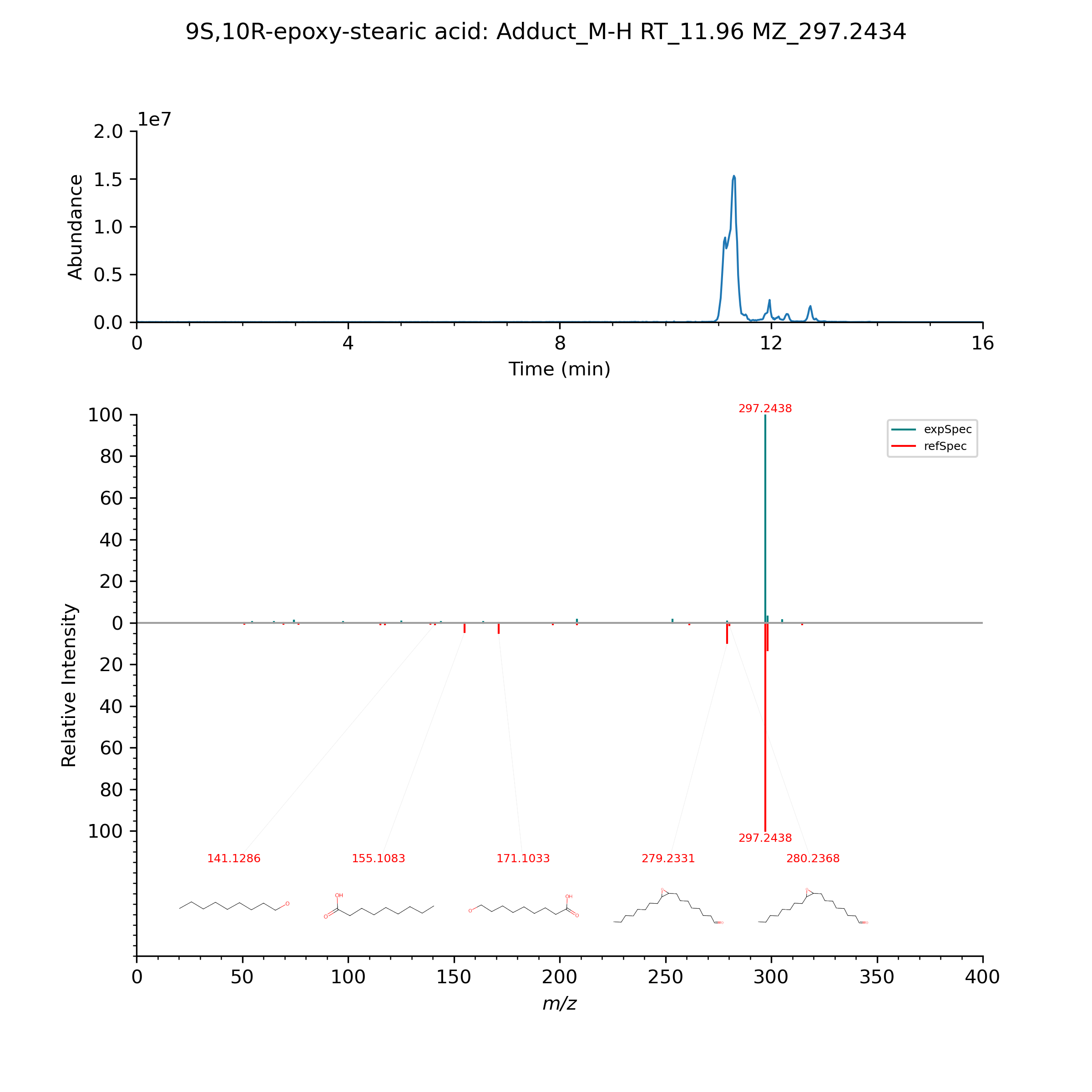

Supplement: Supplementary file 1 [file pharmaceuticals-18-01153-s001.zip › compound structures/M0025.png]

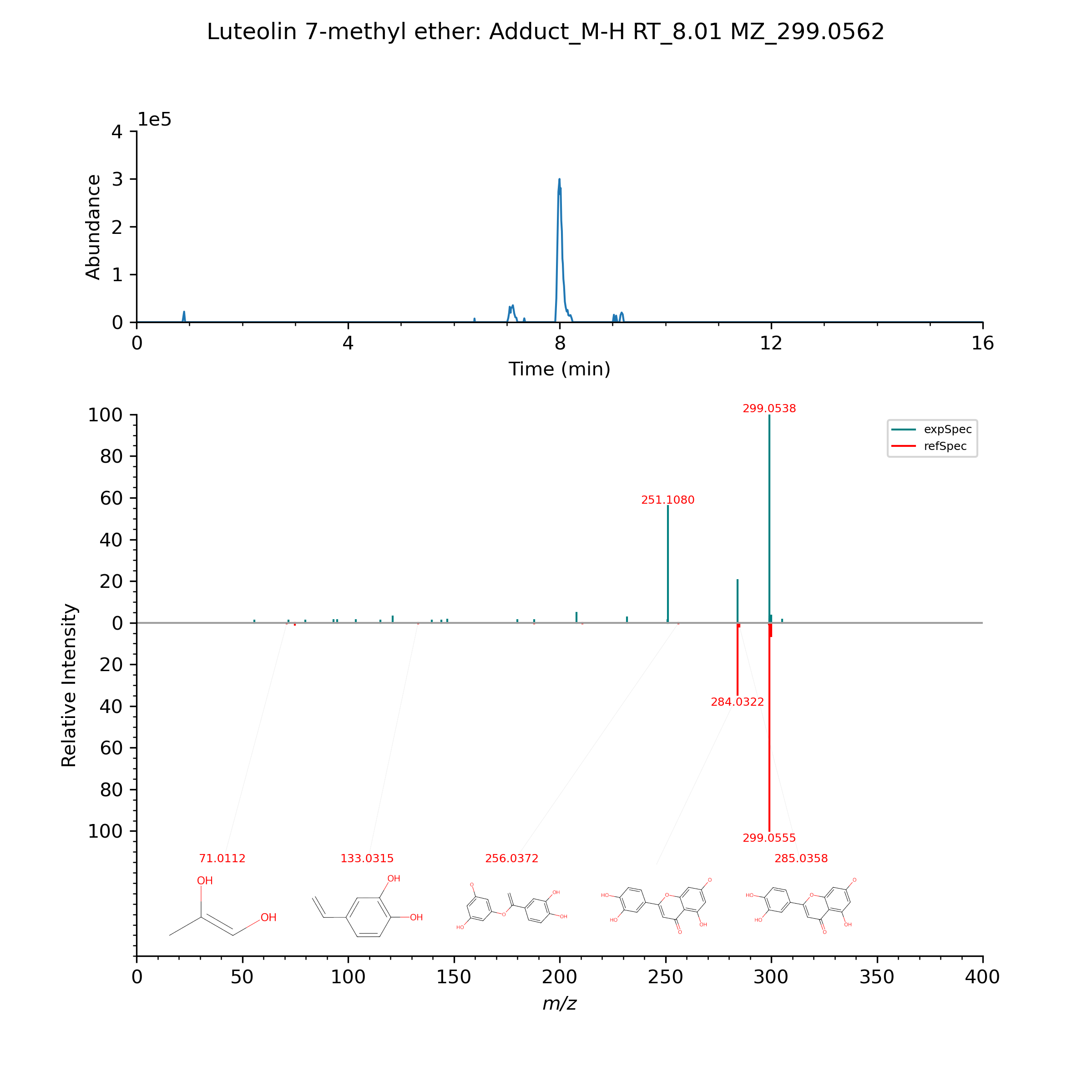

Supplement: Supplementary file 1 [file pharmaceuticals-18-01153-s001.zip › compound structures/M0026.png]

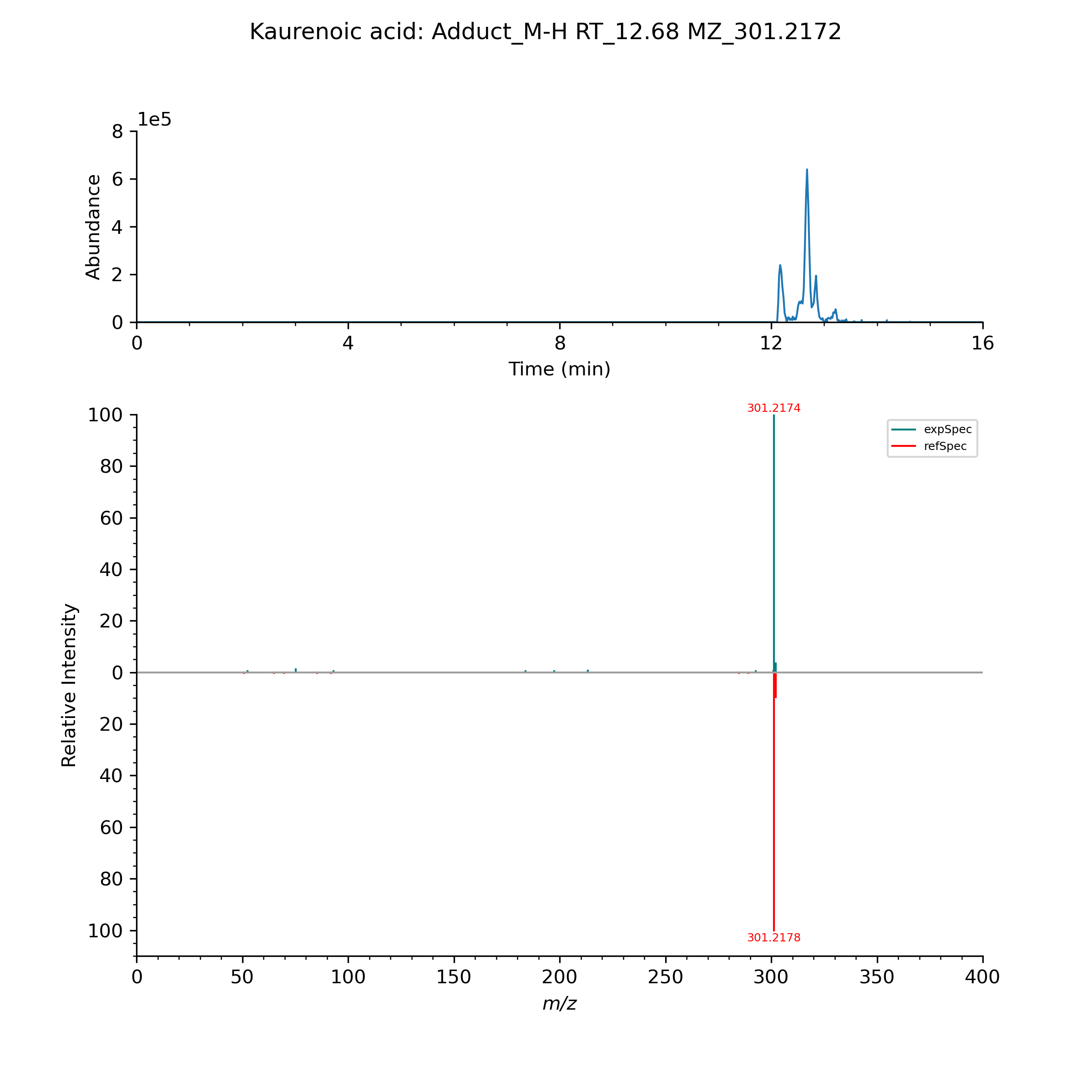

Supplement: Supplementary file 1 [file pharmaceuticals-18-01153-s001.zip › compound structures/M0027.png]

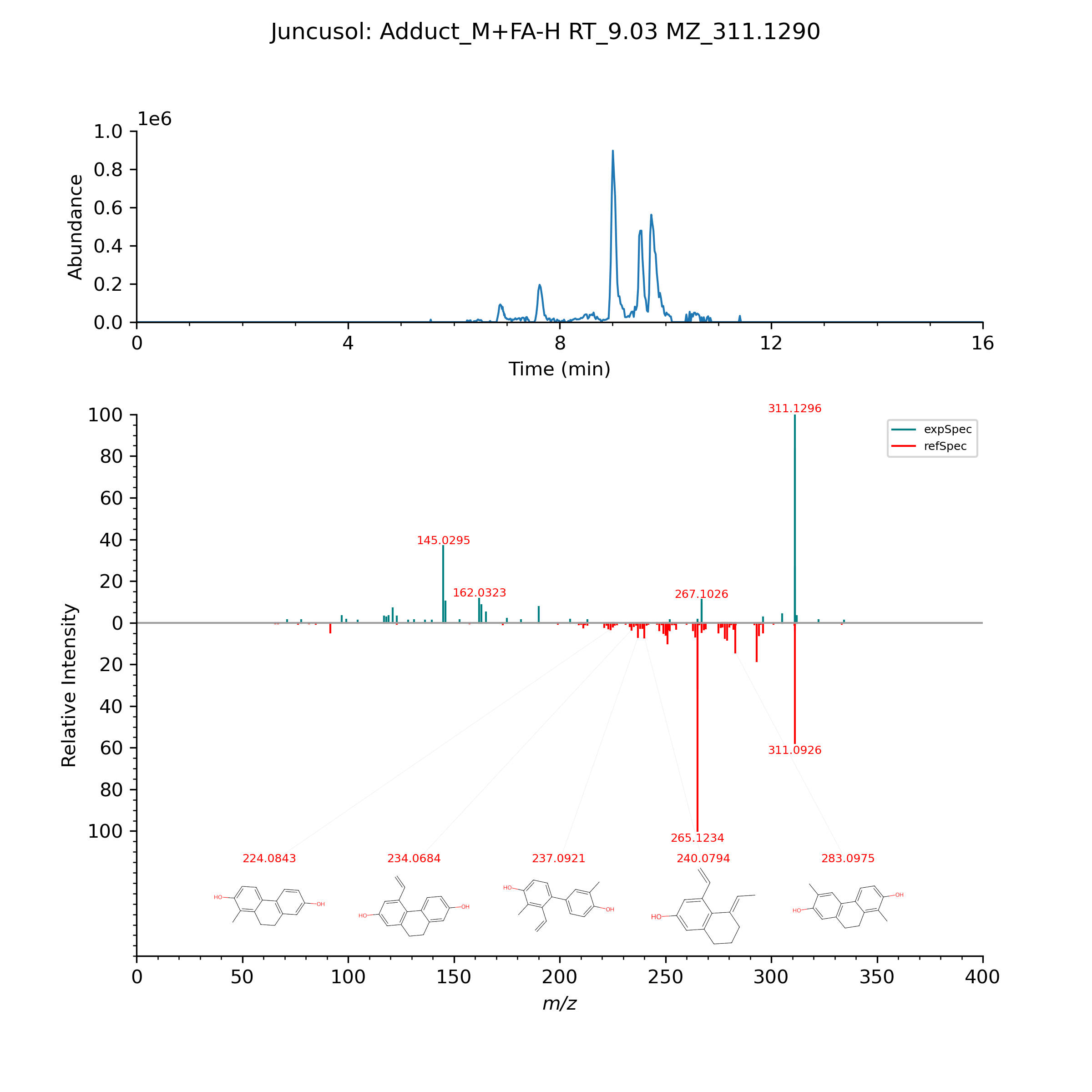

Supplement: Supplementary file 1 [file pharmaceuticals-18-01153-s001.zip › compound structures/M0028.png]

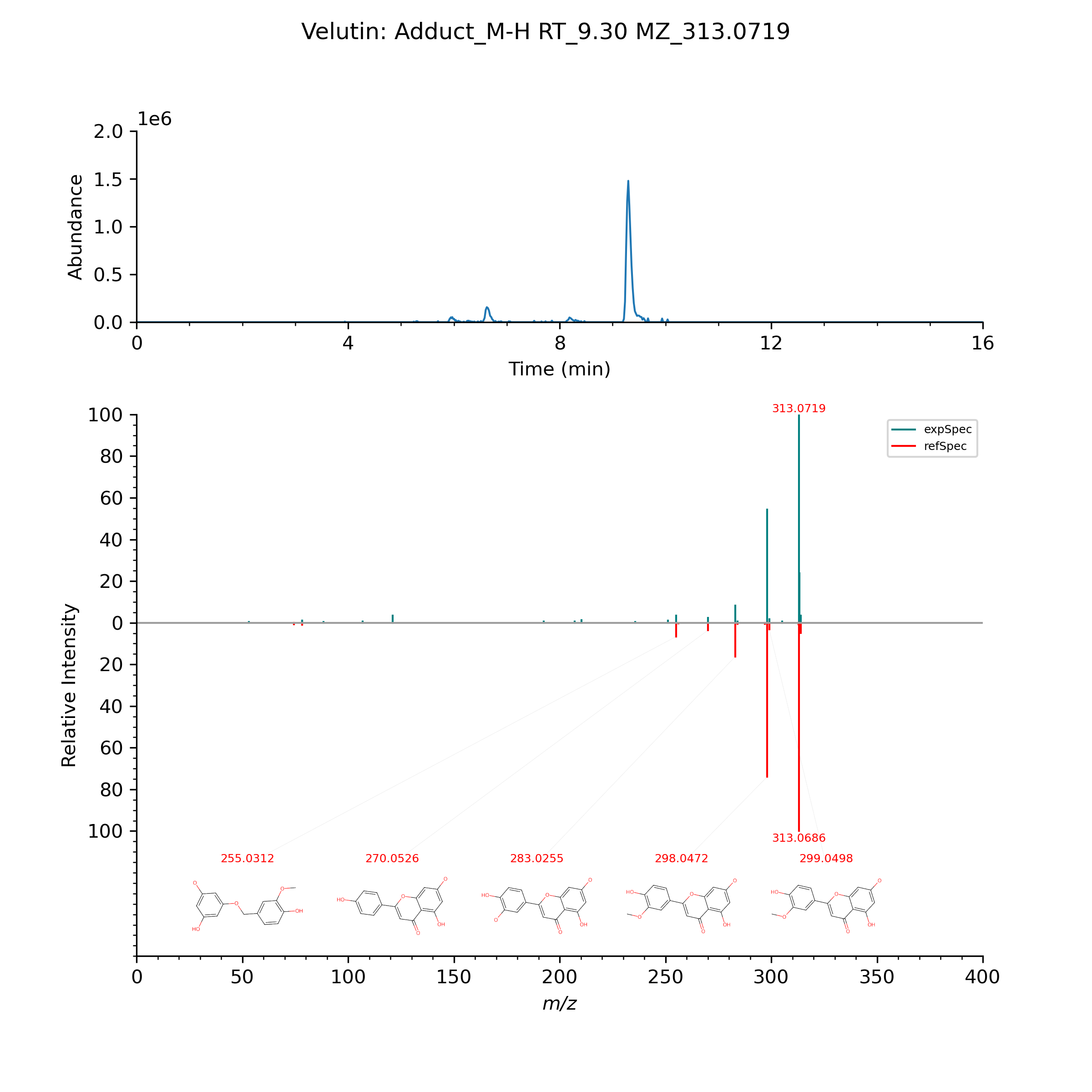

Supplement: Supplementary file 1 [file pharmaceuticals-18-01153-s001.zip › compound structures/M0029.png]

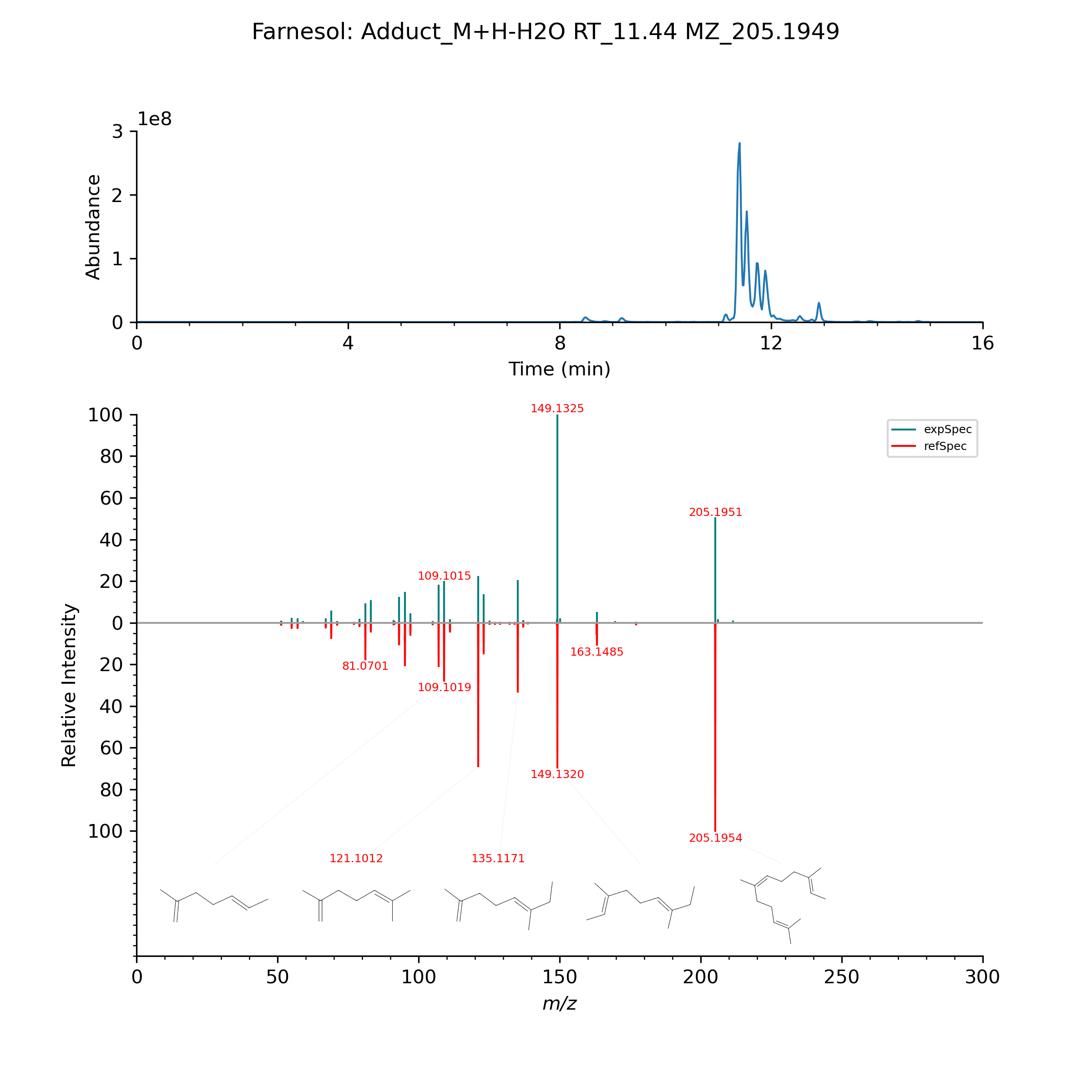

Supplement: Supplementary file 1 [file pharmaceuticals-18-01153-s001.zip › compound structures/M0030.png]

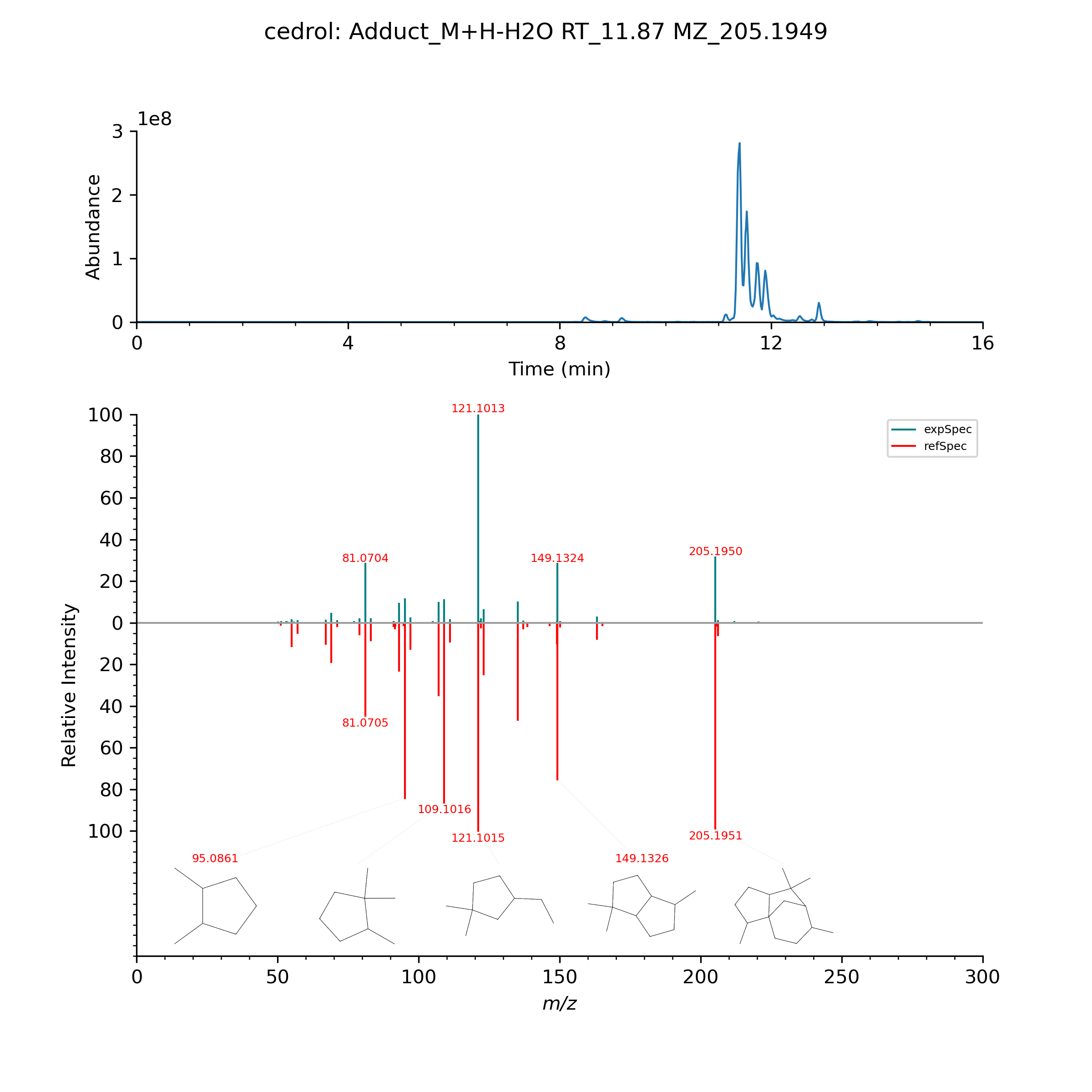

Supplement: Supplementary file 1 [file pharmaceuticals-18-01153-s001.zip › compound structures/M0031.png]

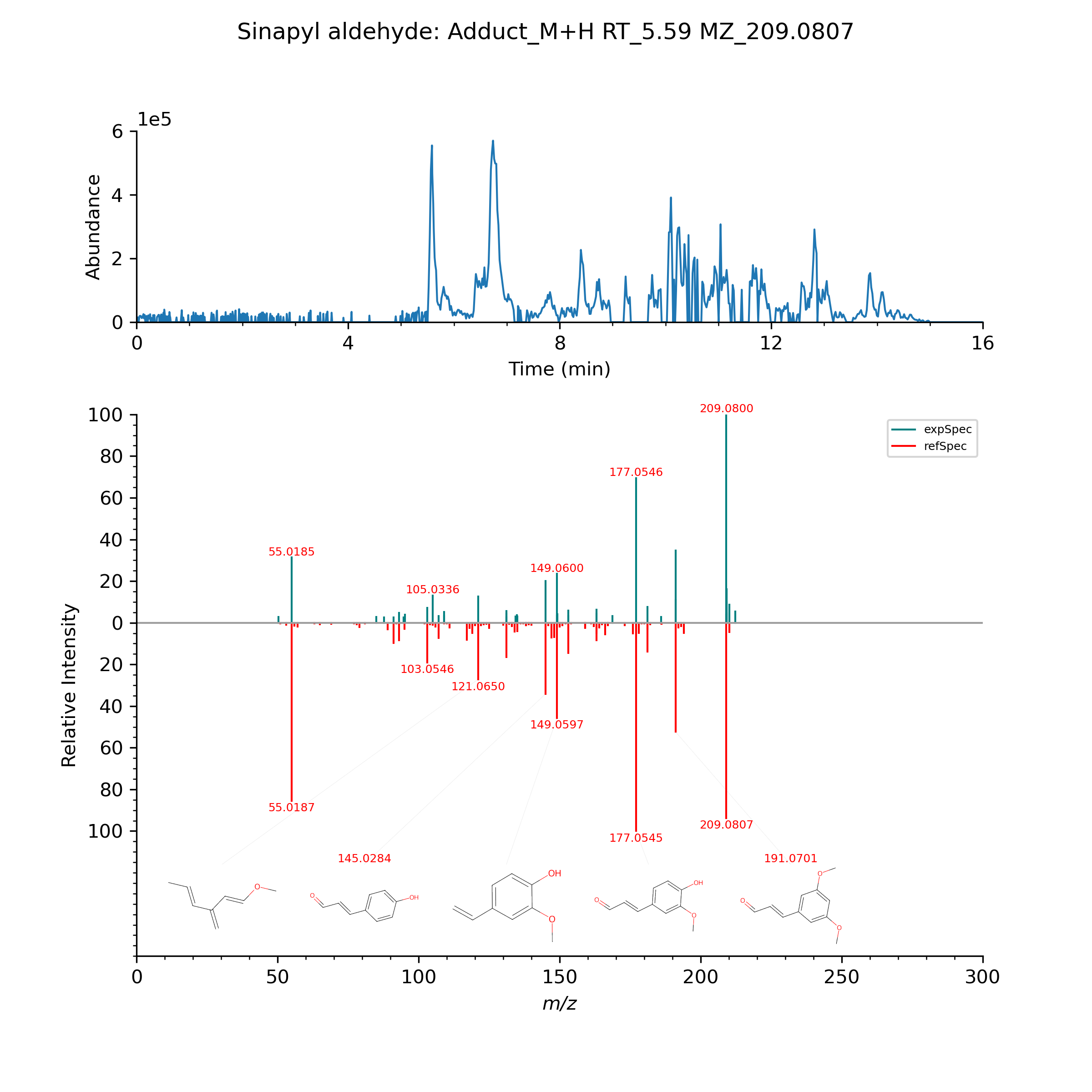

Supplement: Supplementary file 1 [file pharmaceuticals-18-01153-s001.zip › compound structures/M0032.png]

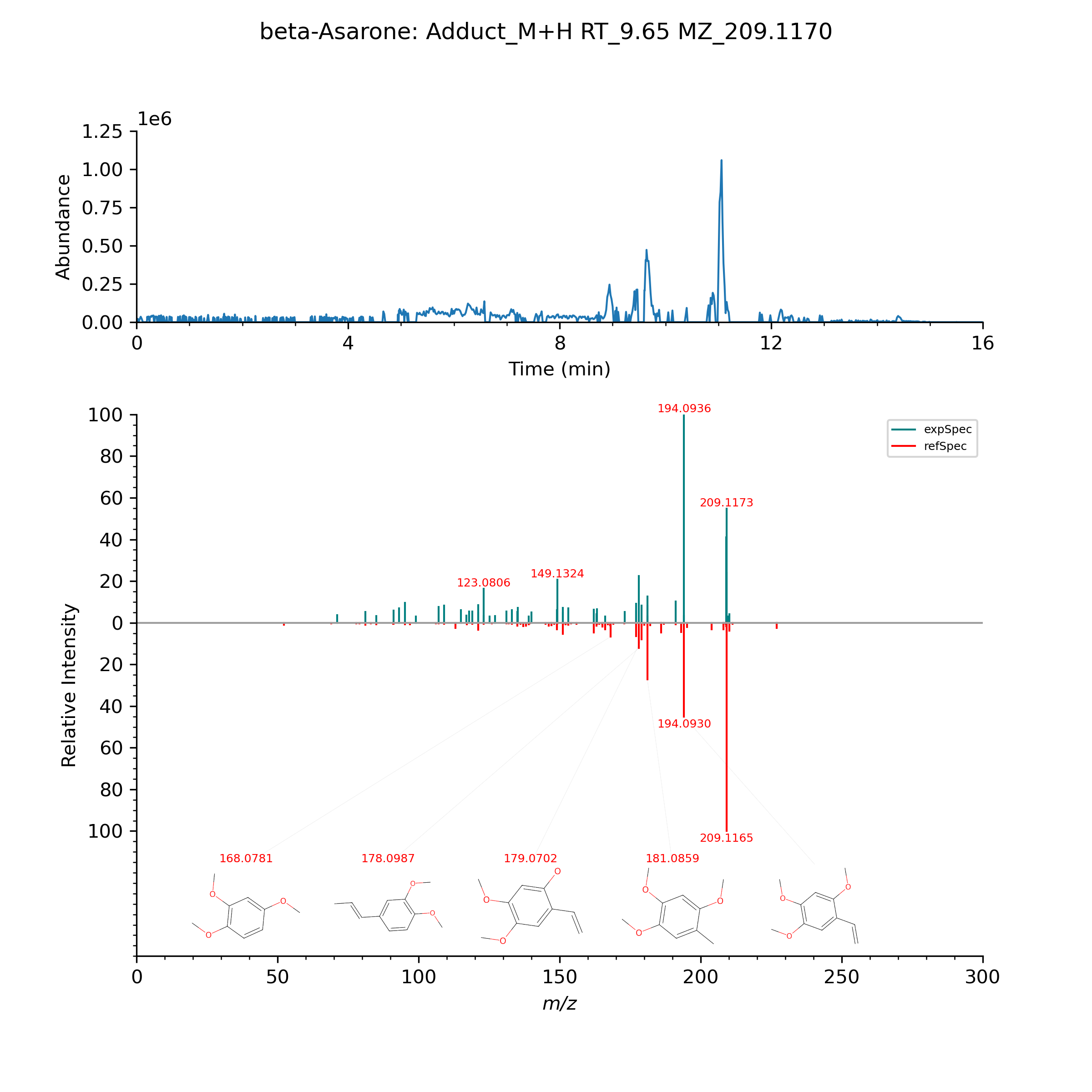

Supplement: Supplementary file 1 [file pharmaceuticals-18-01153-s001.zip › compound structures/M0033.png]

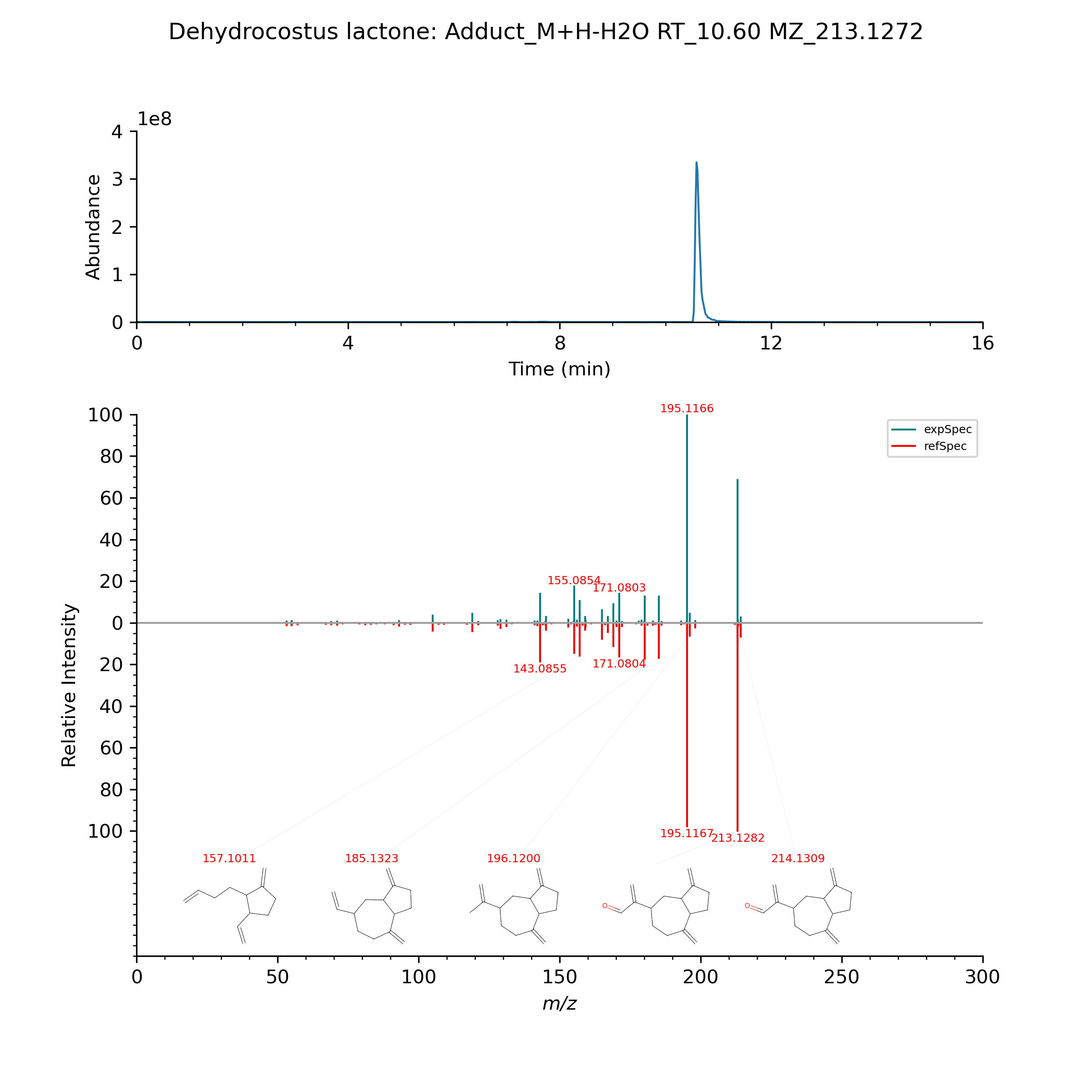

Supplement: Supplementary file 1 [file pharmaceuticals-18-01153-s001.zip › compound structures/M0034.png]

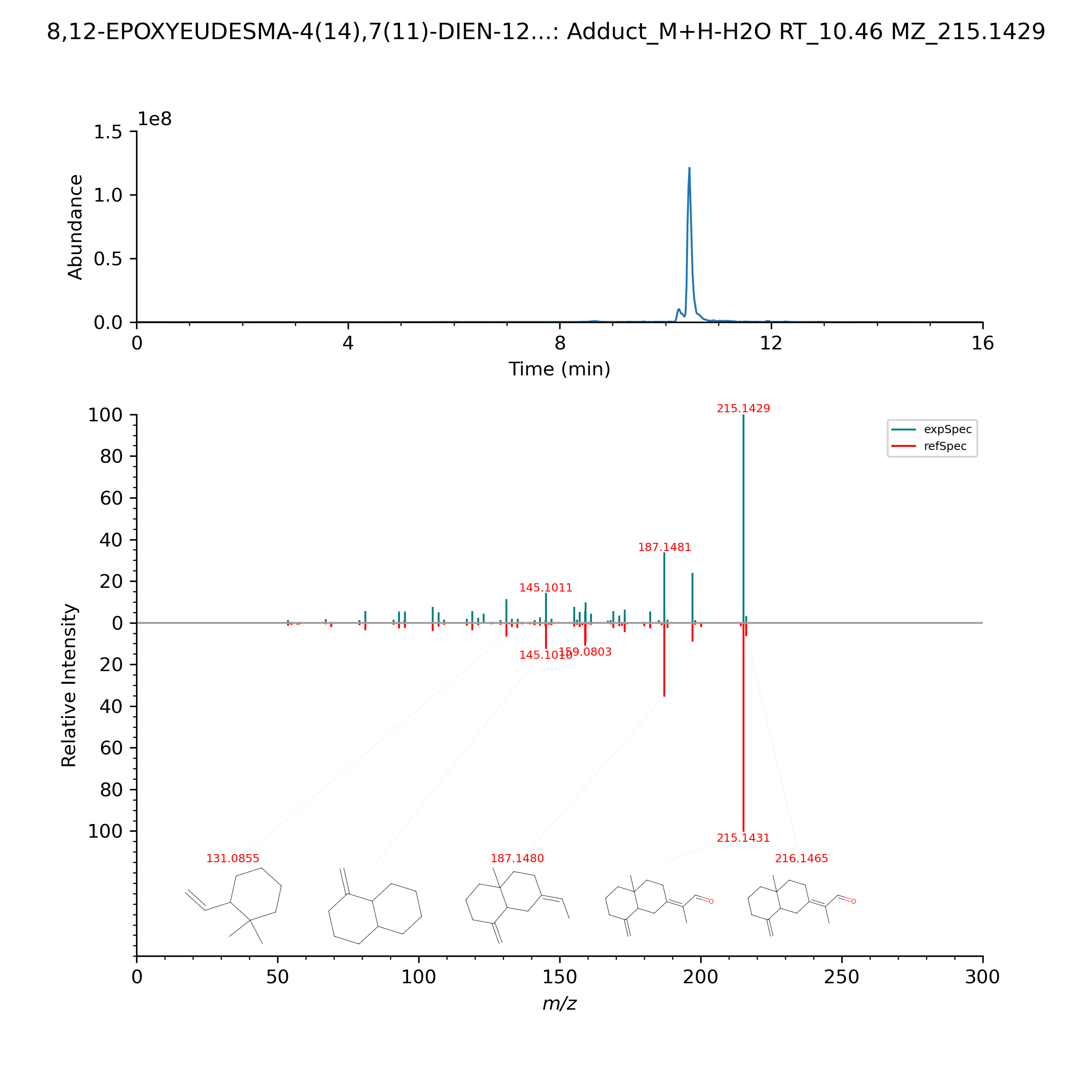

Supplement: Supplementary file 1 [file pharmaceuticals-18-01153-s001.zip › compound structures/M0035.png]

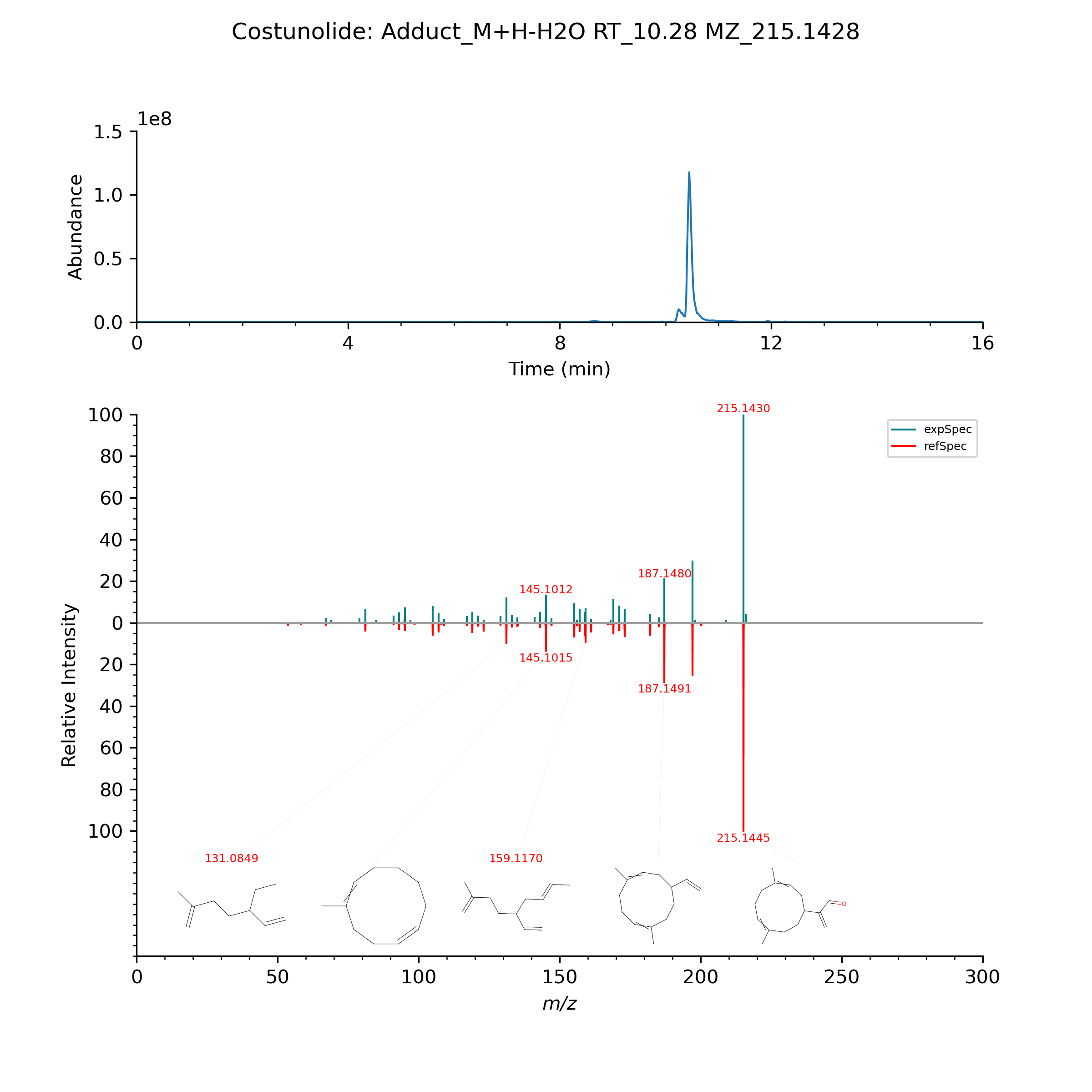

Supplement: Supplementary file 1 [file pharmaceuticals-18-01153-s001.zip › compound structures/M0036.png]

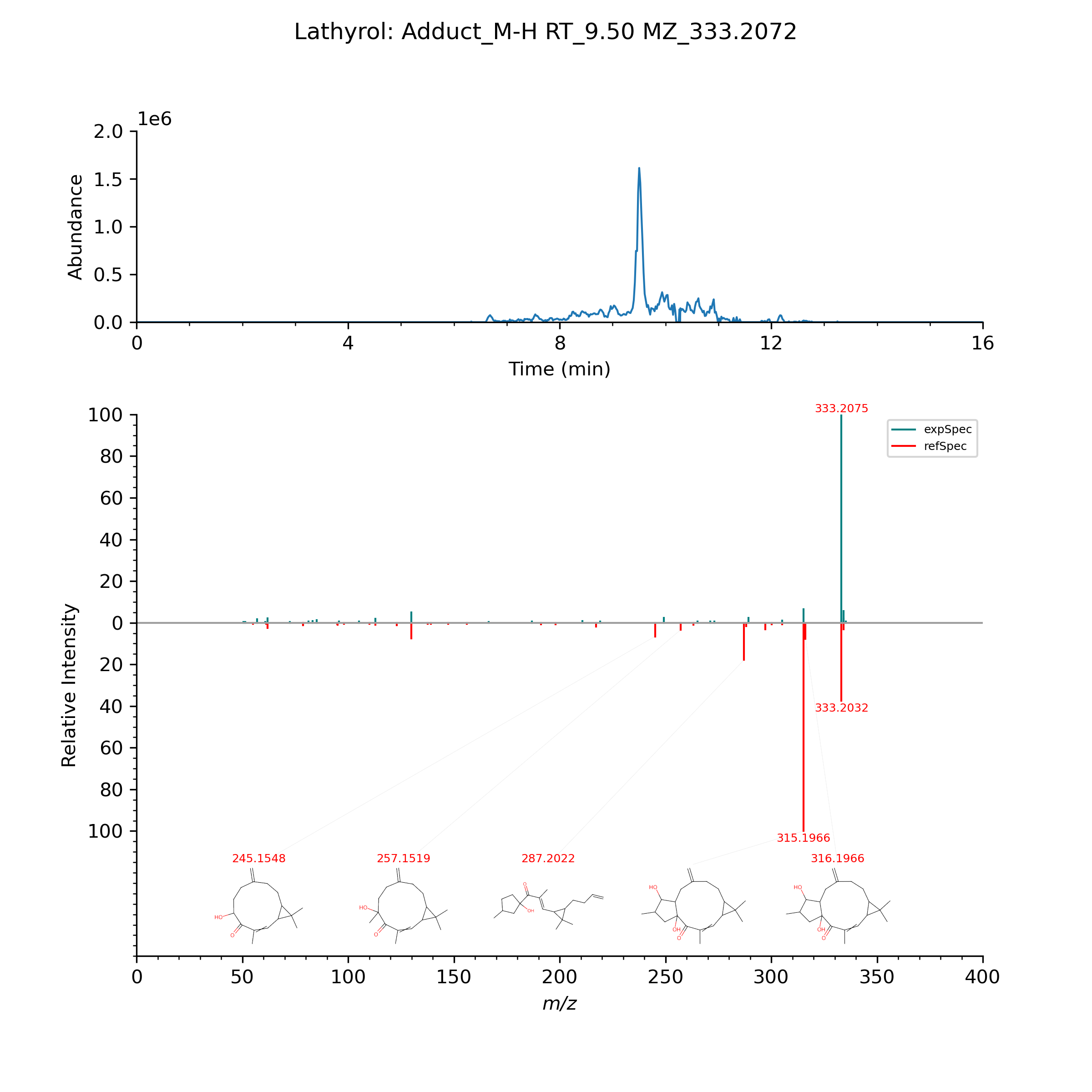

Supplement: Supplementary file 1 [file pharmaceuticals-18-01153-s001.zip › compound structures/M0037.png]

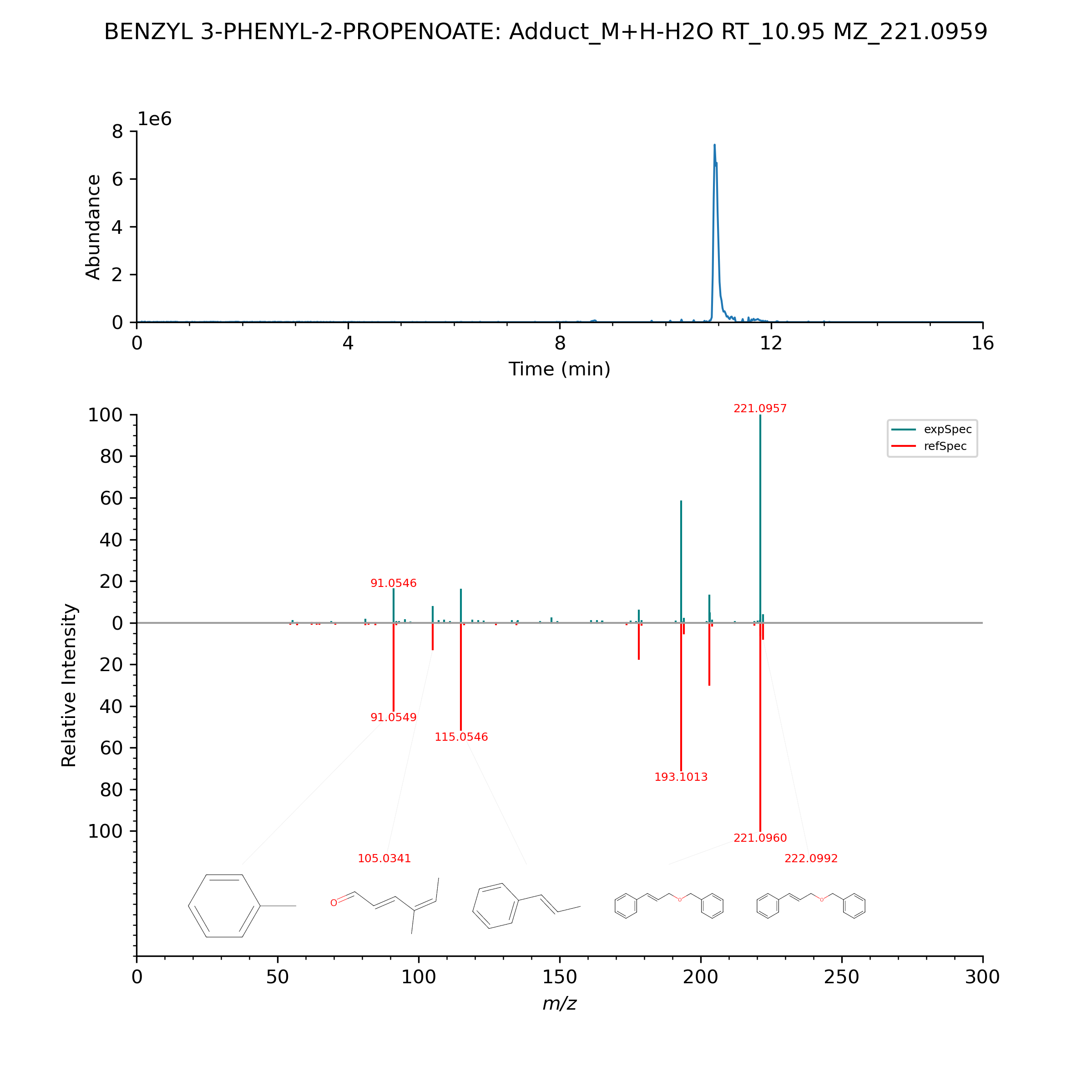

Supplement: Supplementary file 1 [file pharmaceuticals-18-01153-s001.zip › compound structures/M0038.png]

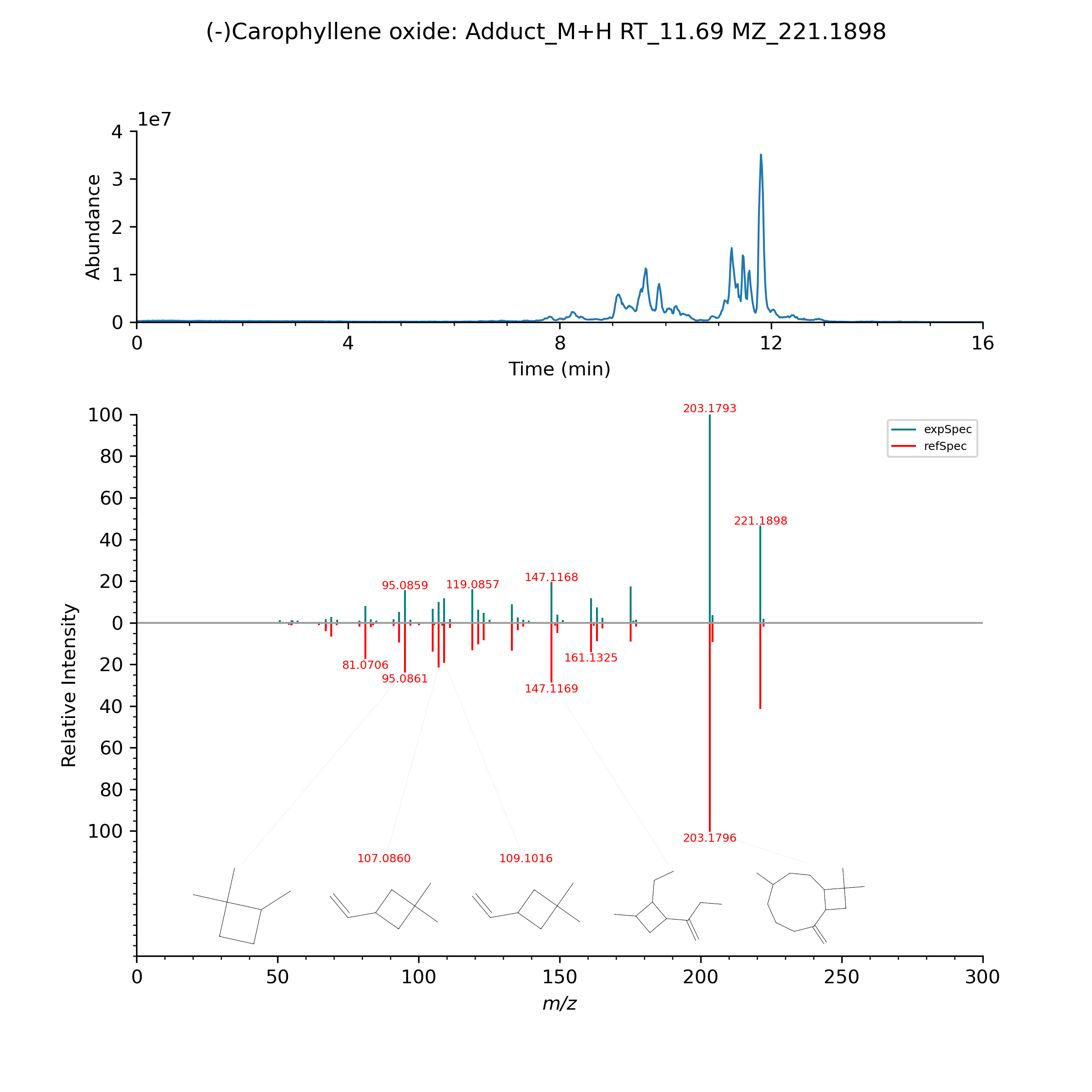

Supplement: Supplementary file 1 [file pharmaceuticals-18-01153-s001.zip › compound structures/M0039.png]

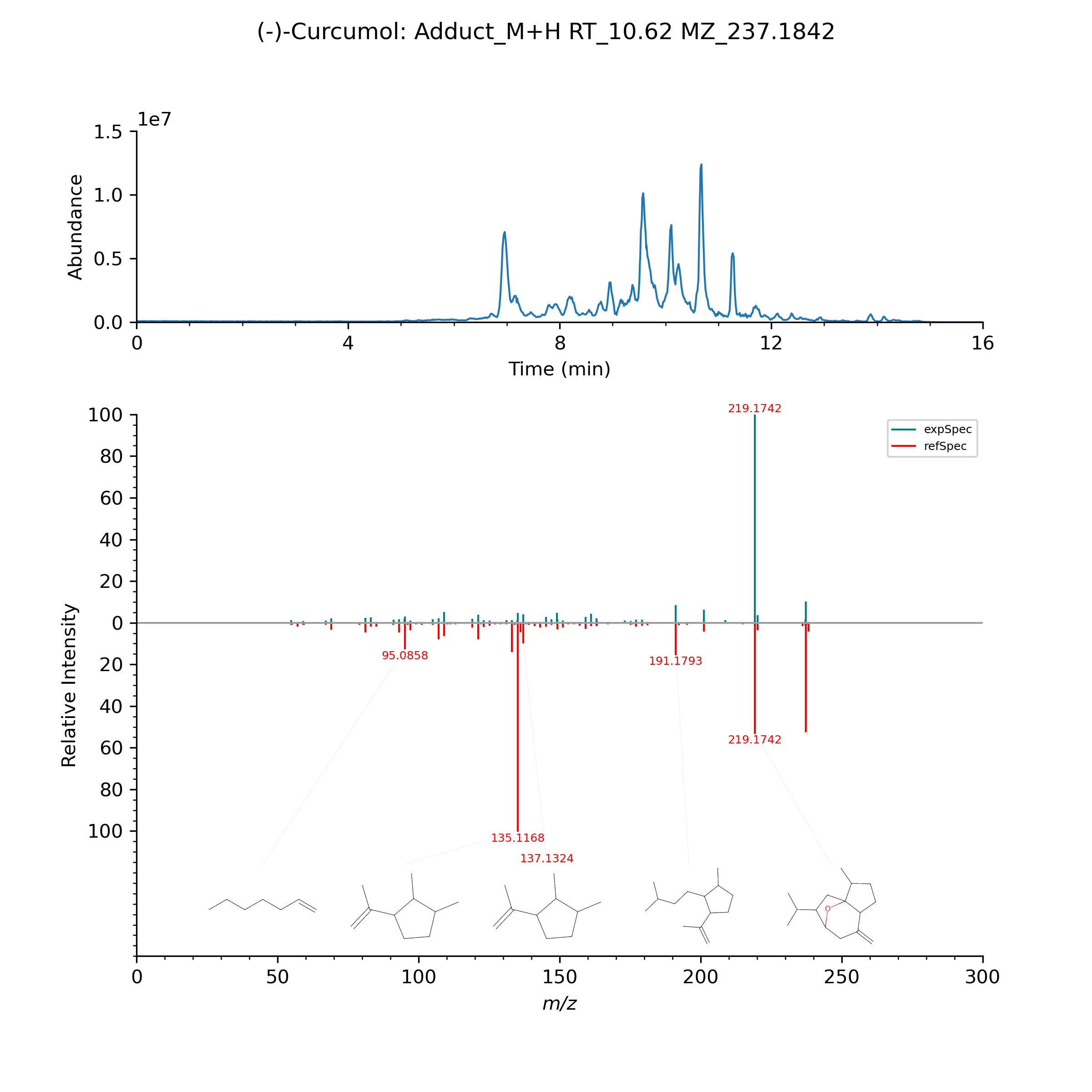

Supplement: Supplementary file 1 [file pharmaceuticals-18-01153-s001.zip › compound structures/M0040.png]

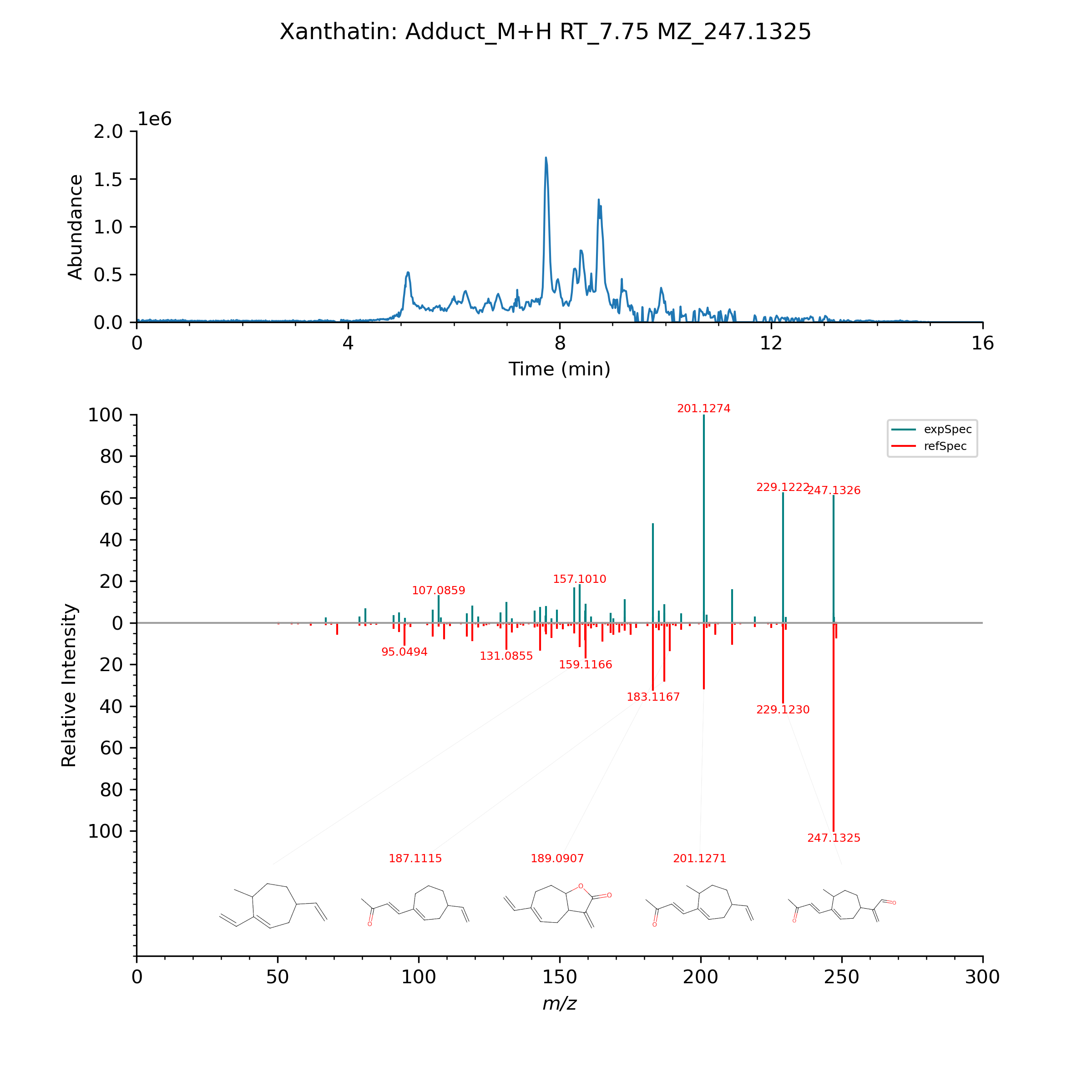

Supplement: Supplementary file 1 [file pharmaceuticals-18-01153-s001.zip › compound structures/M0041.png]

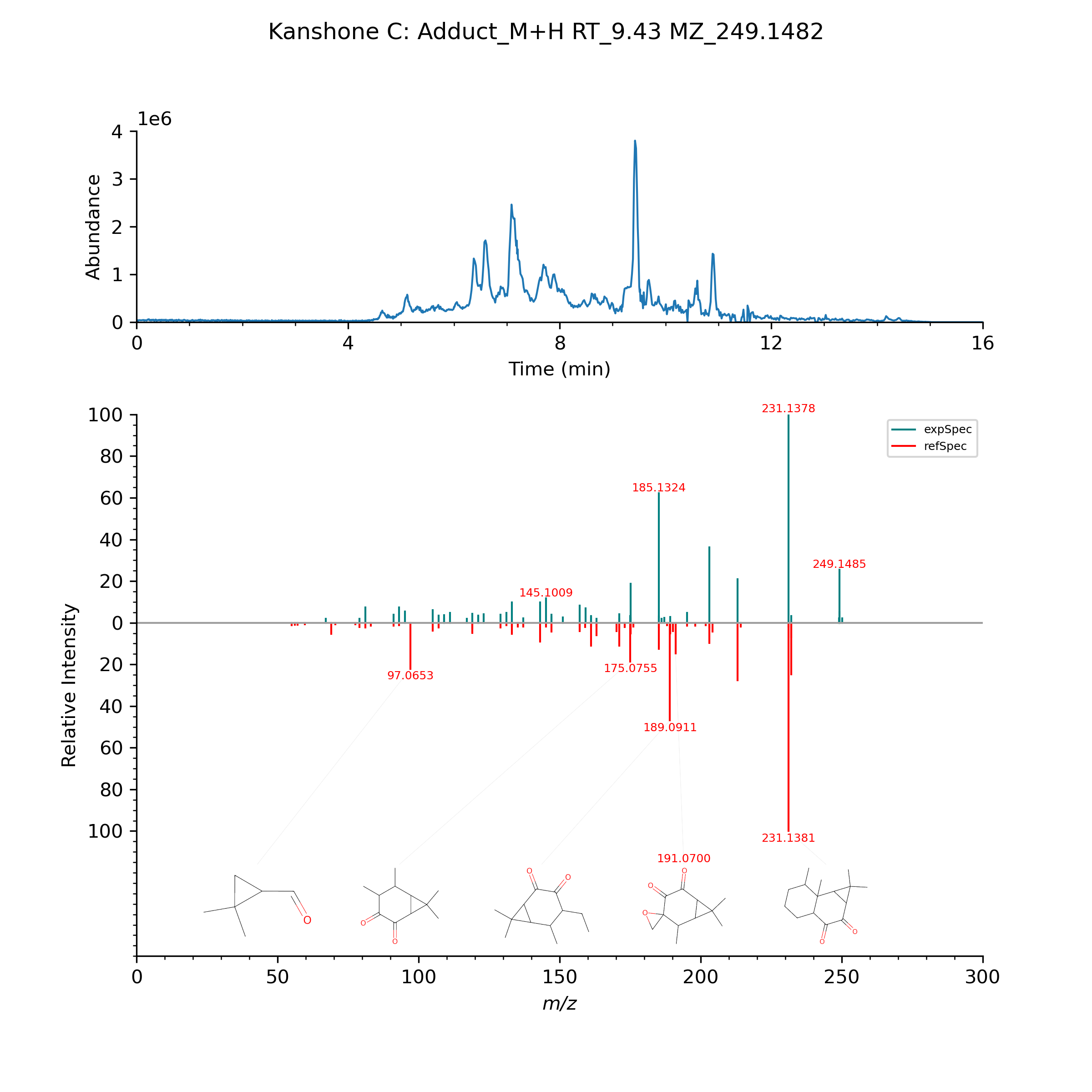

Supplement: Supplementary file 1 [file pharmaceuticals-18-01153-s001.zip › compound structures/M0042.png]

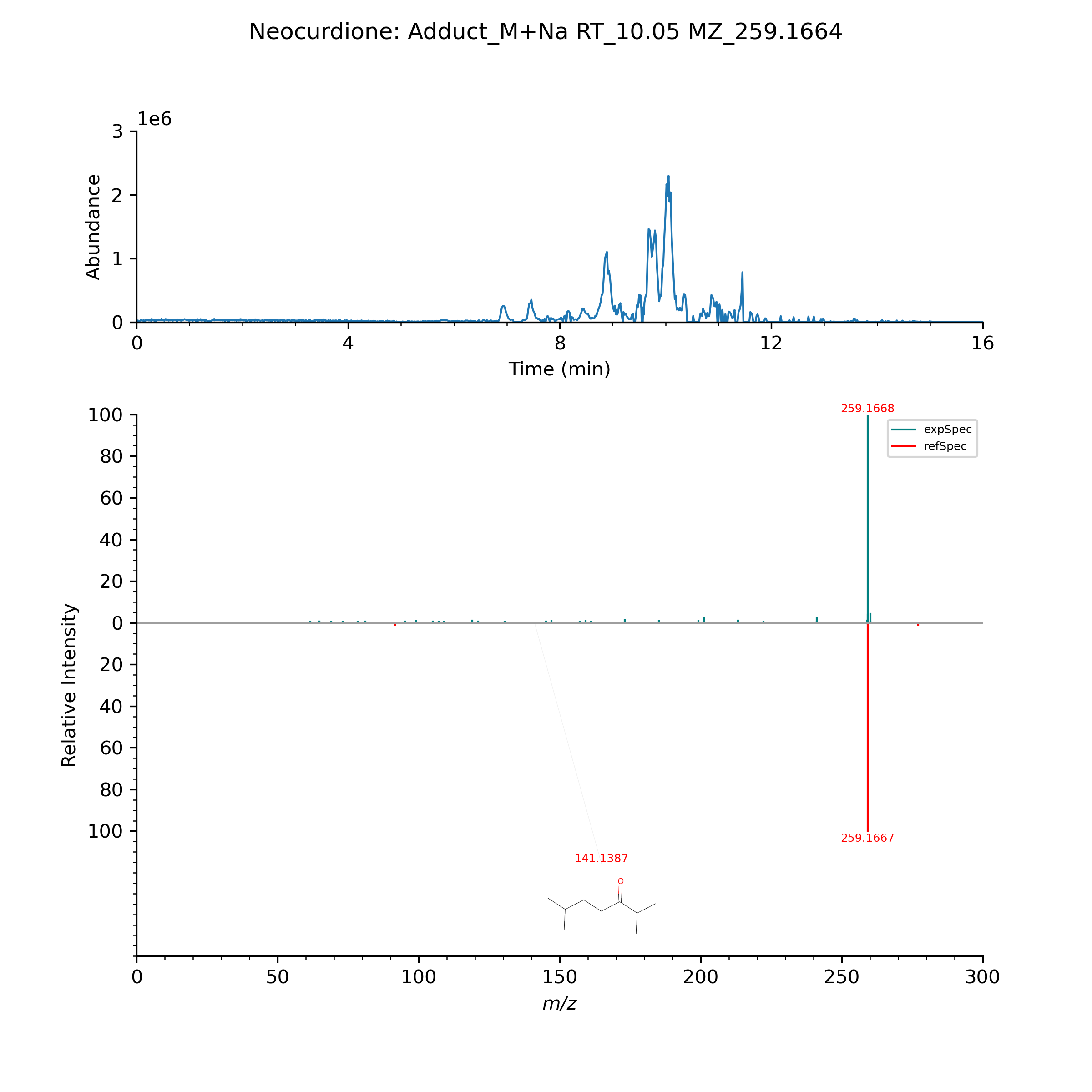

Supplement: Supplementary file 1 [file pharmaceuticals-18-01153-s001.zip › compound structures/M0043.png]

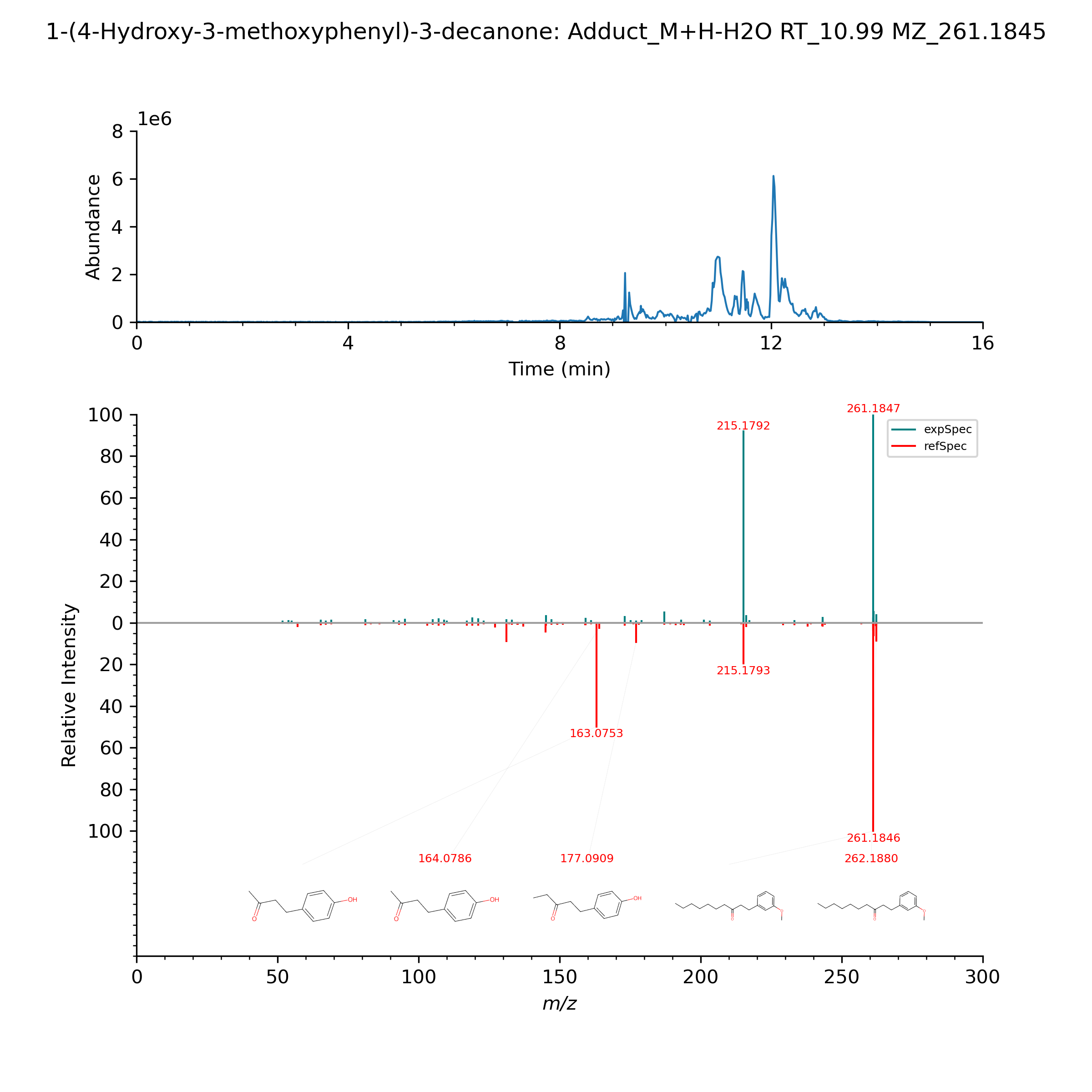

Supplement: Supplementary file 1 [file pharmaceuticals-18-01153-s001.zip › compound structures/M0044.png]

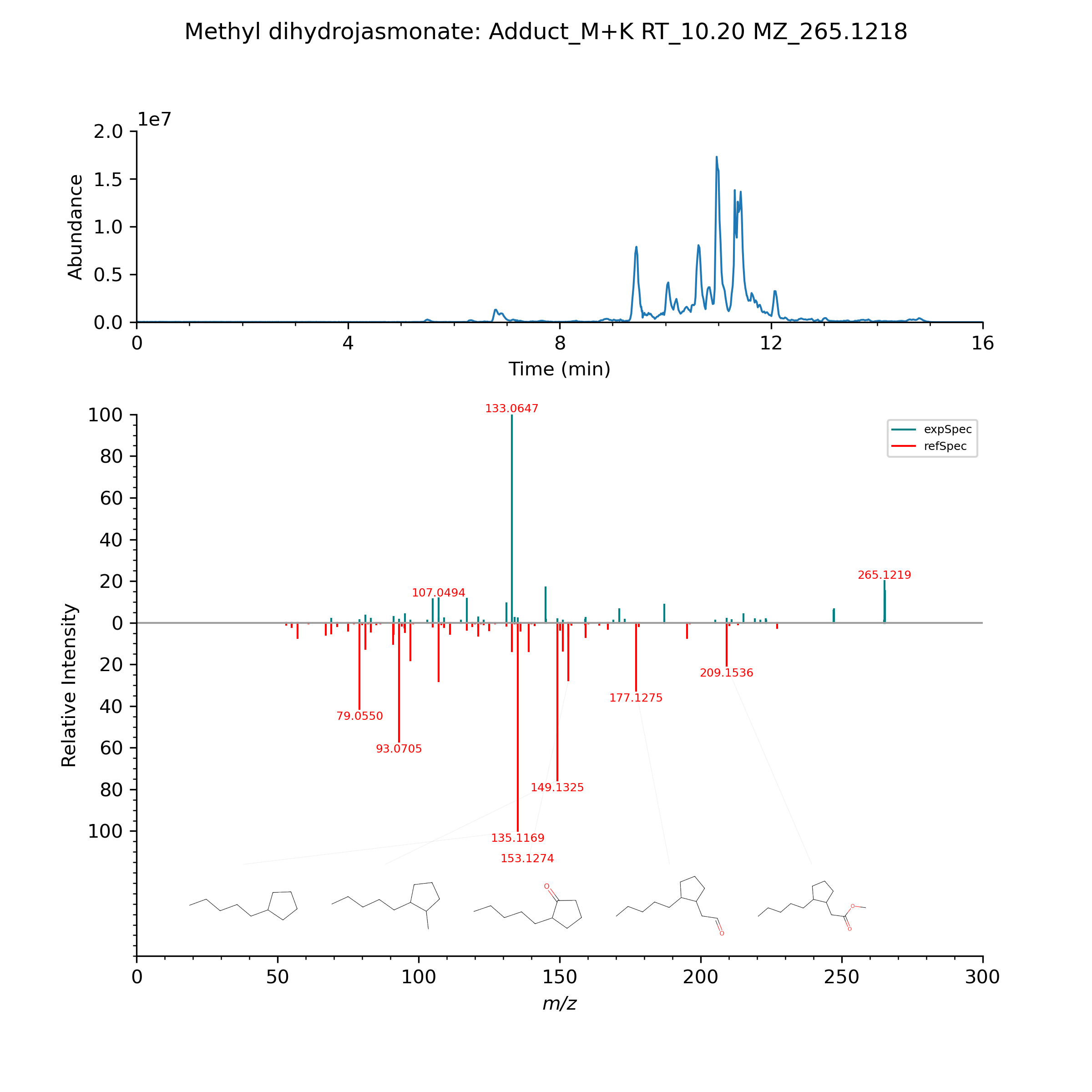

Supplement: Supplementary file 1 [file pharmaceuticals-18-01153-s001.zip › compound structures/M0045.png]

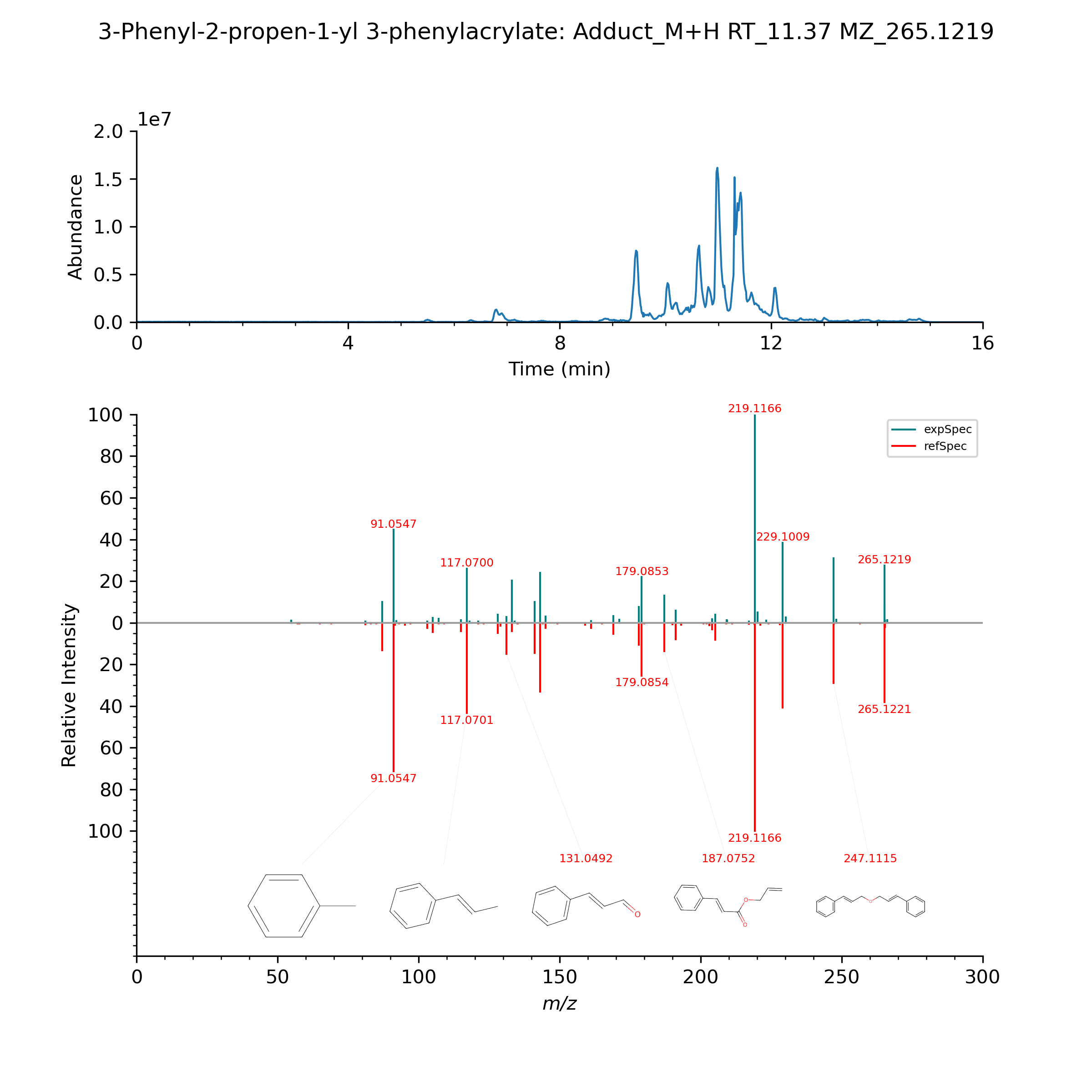

Supplement: Supplementary file 1 [file pharmaceuticals-18-01153-s001.zip › compound structures/M0046.png]

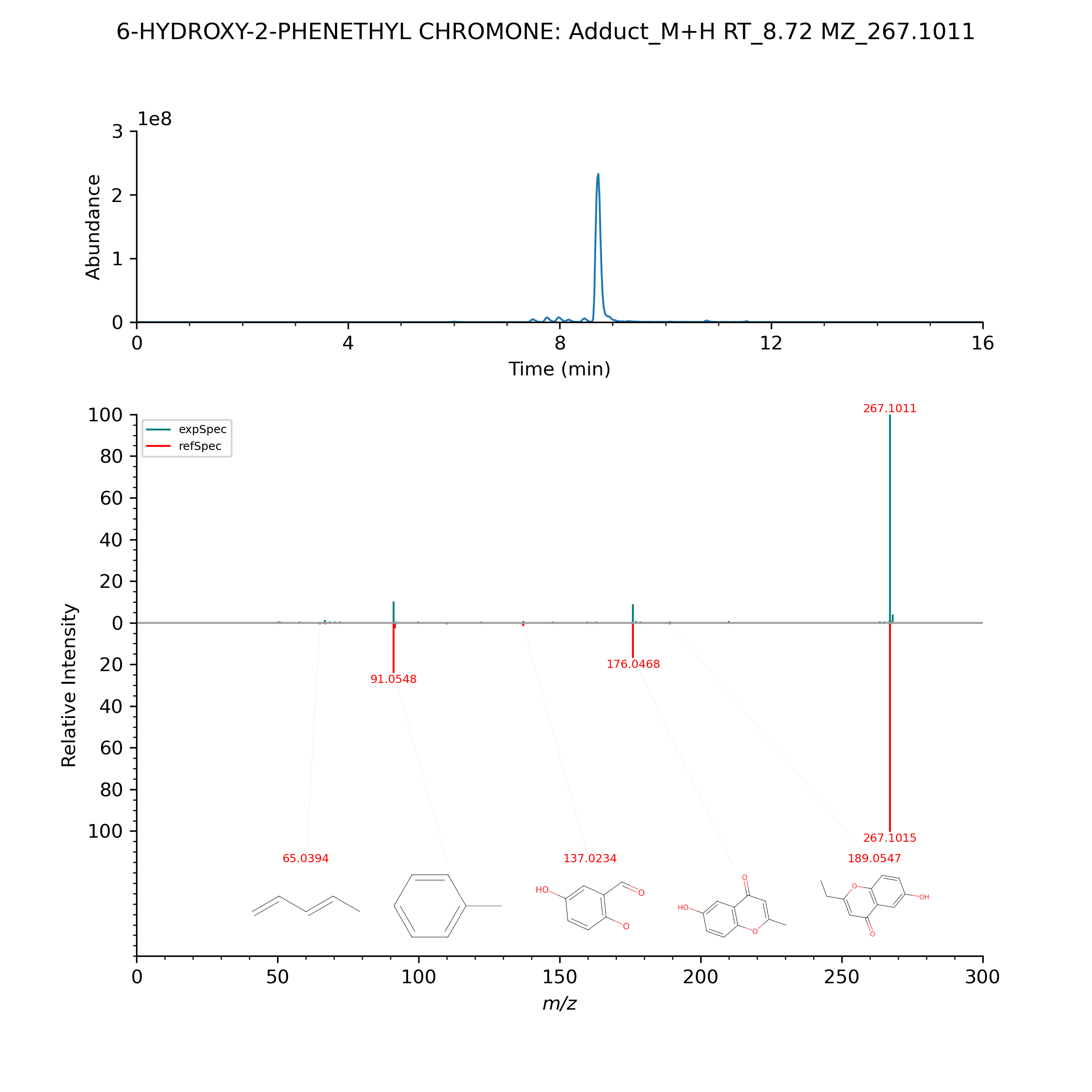

Supplement: Supplementary file 1 [file pharmaceuticals-18-01153-s001.zip › compound structures/M0047.png]

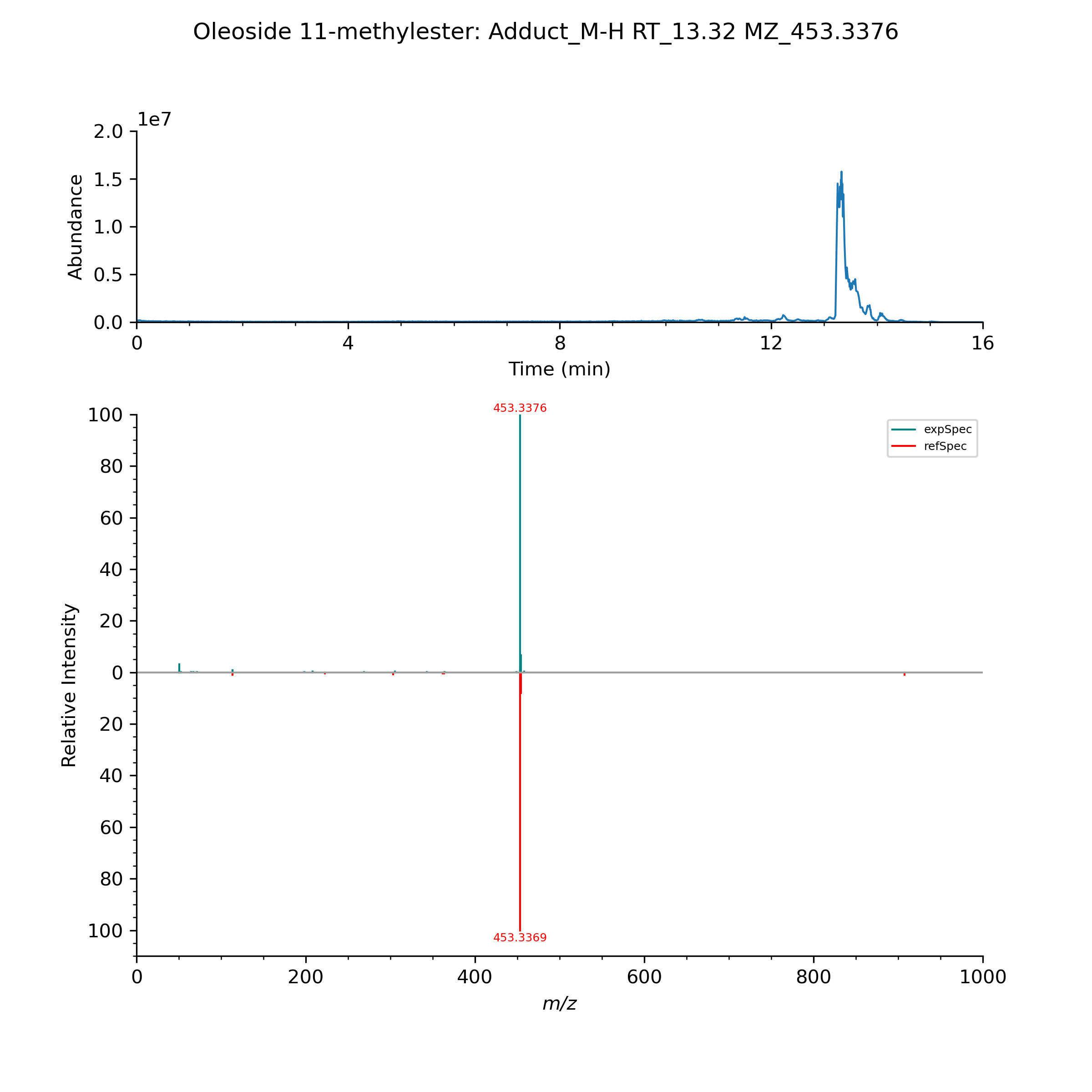

Supplement: Supplementary file 1 [file pharmaceuticals-18-01153-s001.zip › compound structures/M0048.png]

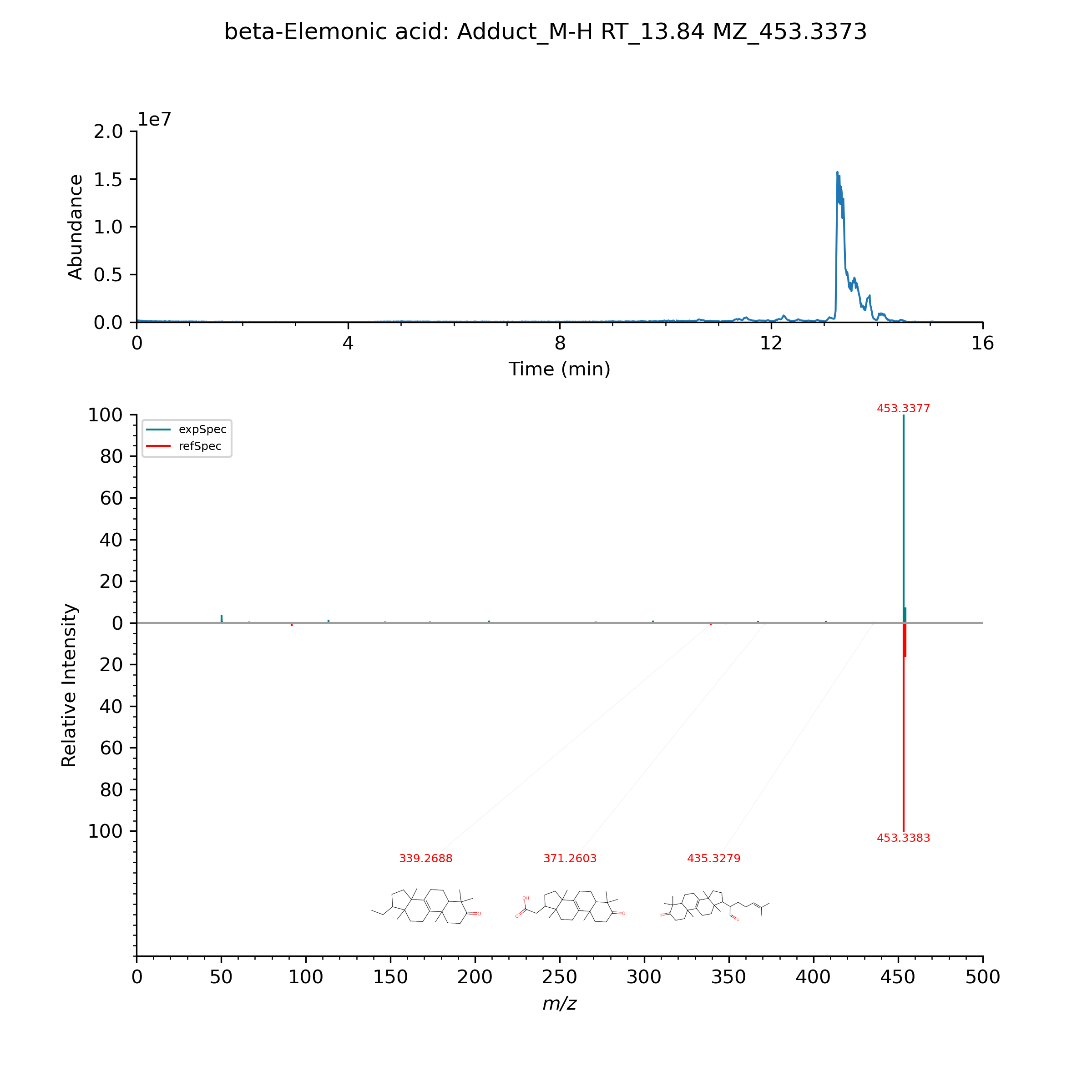

Supplement: Supplementary file 1 [file pharmaceuticals-18-01153-s001.zip › compound structures/M0049.png]

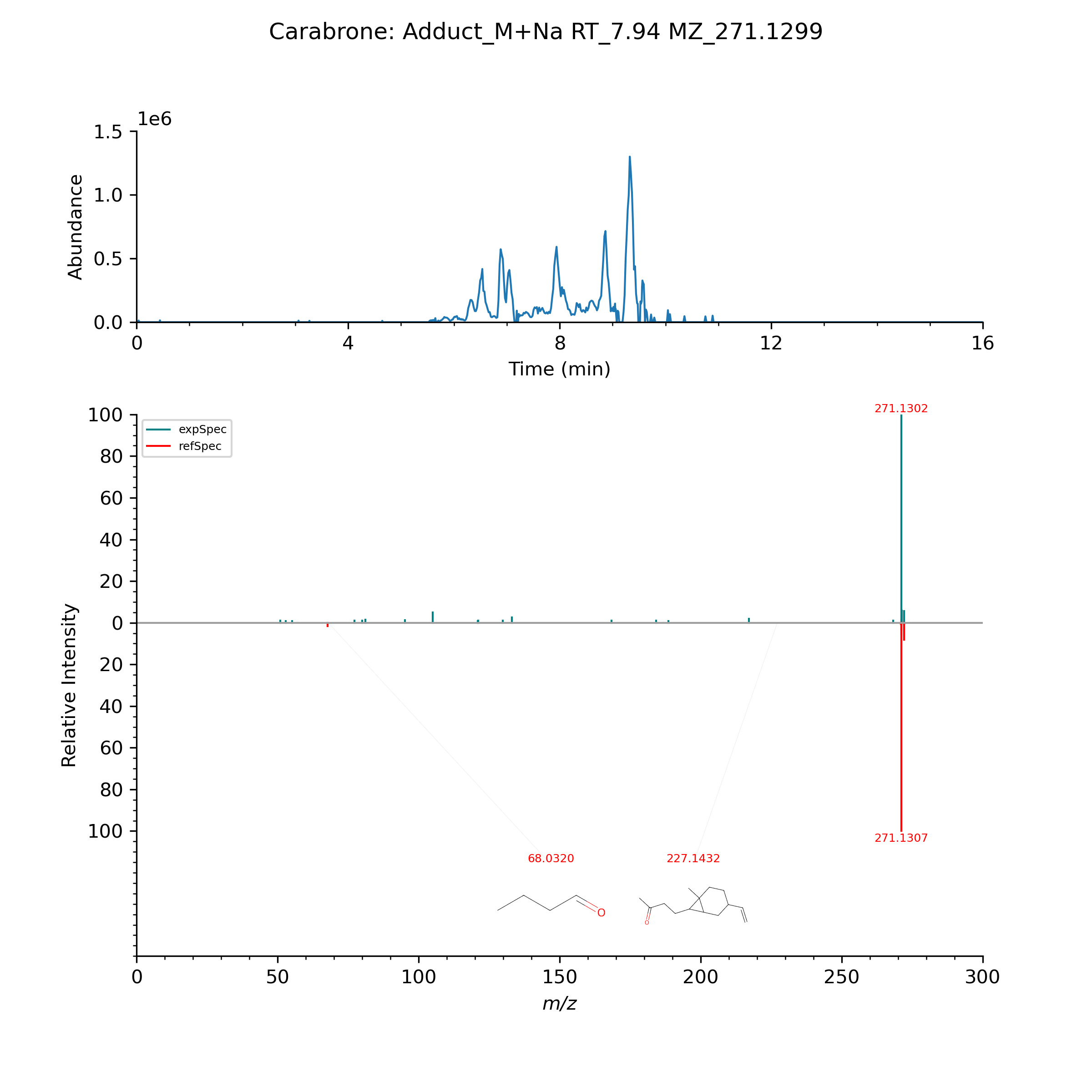

Supplement: Supplementary file 1 [file pharmaceuticals-18-01153-s001.zip › compound structures/M0050.png]

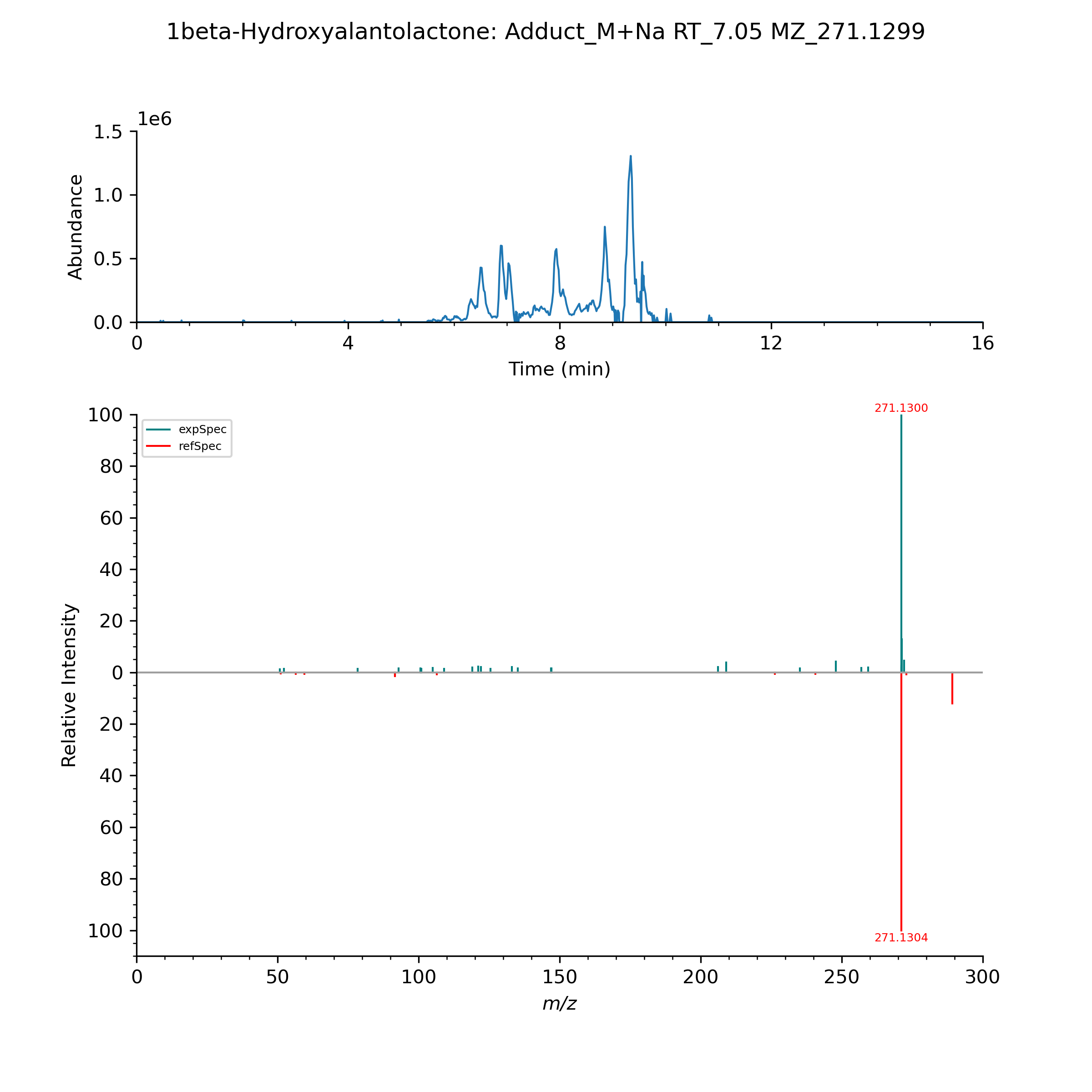

Supplement: Supplementary file 1 [file pharmaceuticals-18-01153-s001.zip › compound structures/M0051.png]

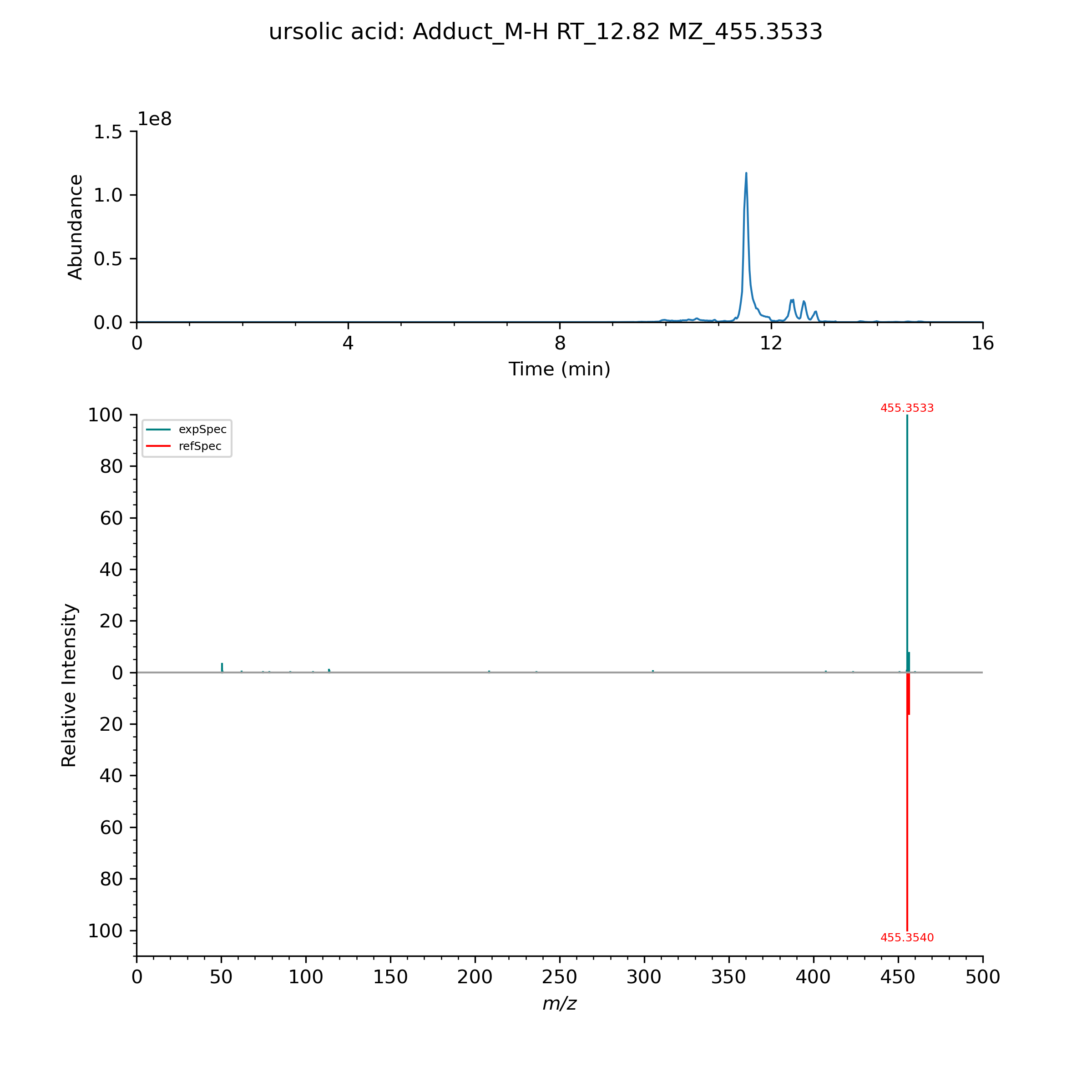

Supplement: Supplementary file 1 [file pharmaceuticals-18-01153-s001.zip › compound structures/M0052.png]

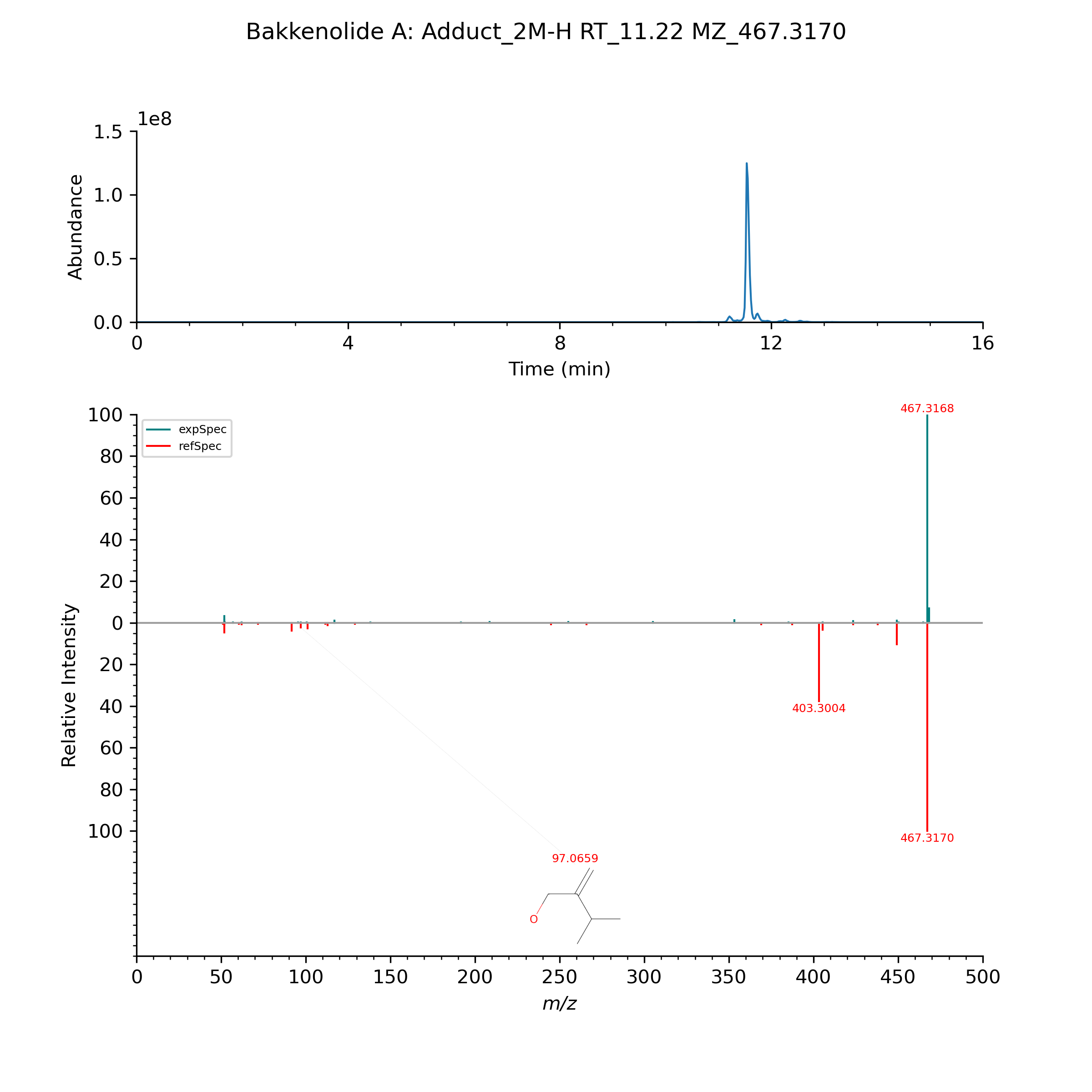

Supplement: Supplementary file 1 [file pharmaceuticals-18-01153-s001.zip › compound structures/M0053.png]

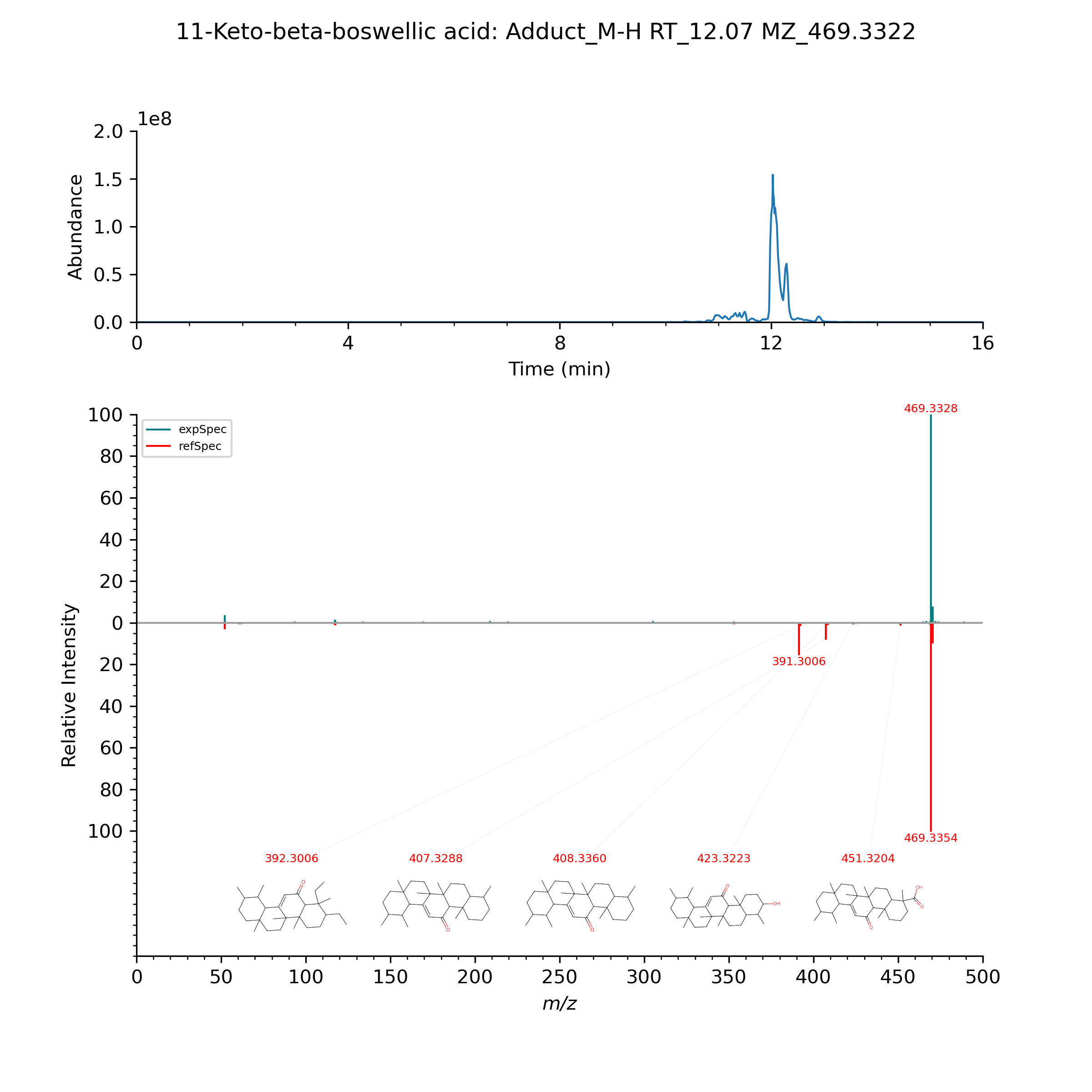

Supplement: Supplementary file 1 [file pharmaceuticals-18-01153-s001.zip › compound structures/M0054.png]

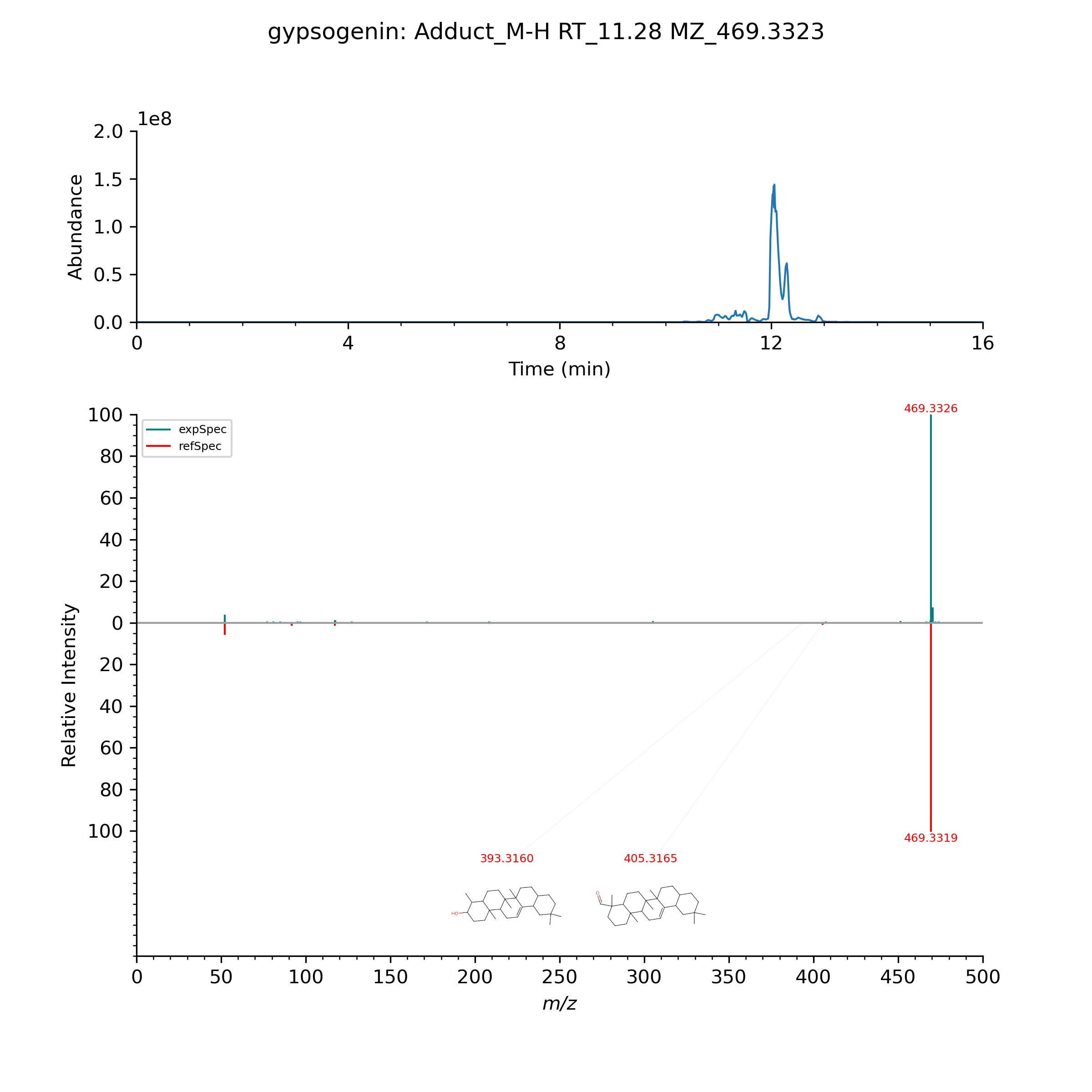

Supplement: Supplementary file 1 [file pharmaceuticals-18-01153-s001.zip › compound structures/M0055.png]

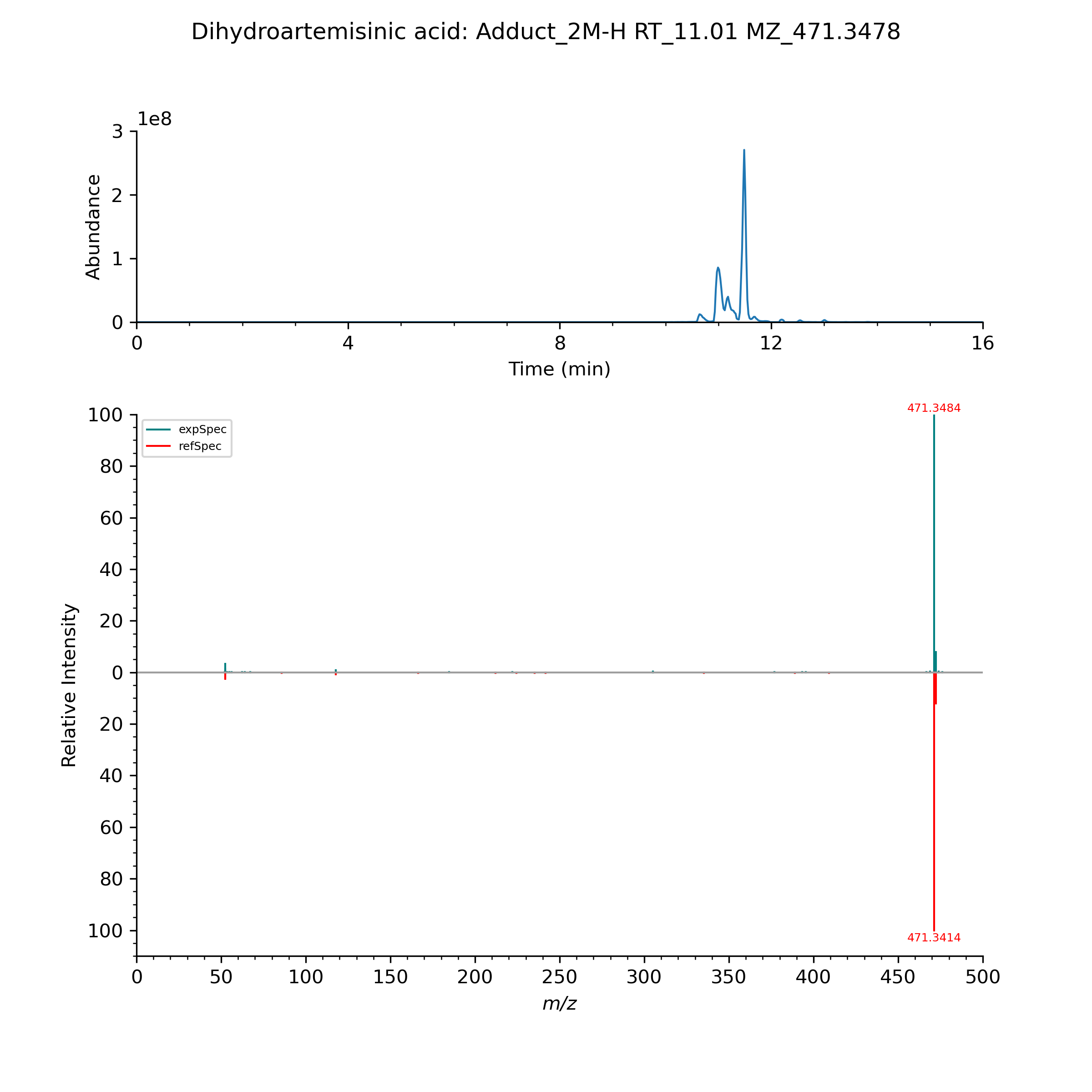

Supplement: Supplementary file 1 [file pharmaceuticals-18-01153-s001.zip › compound structures/M0056.png]

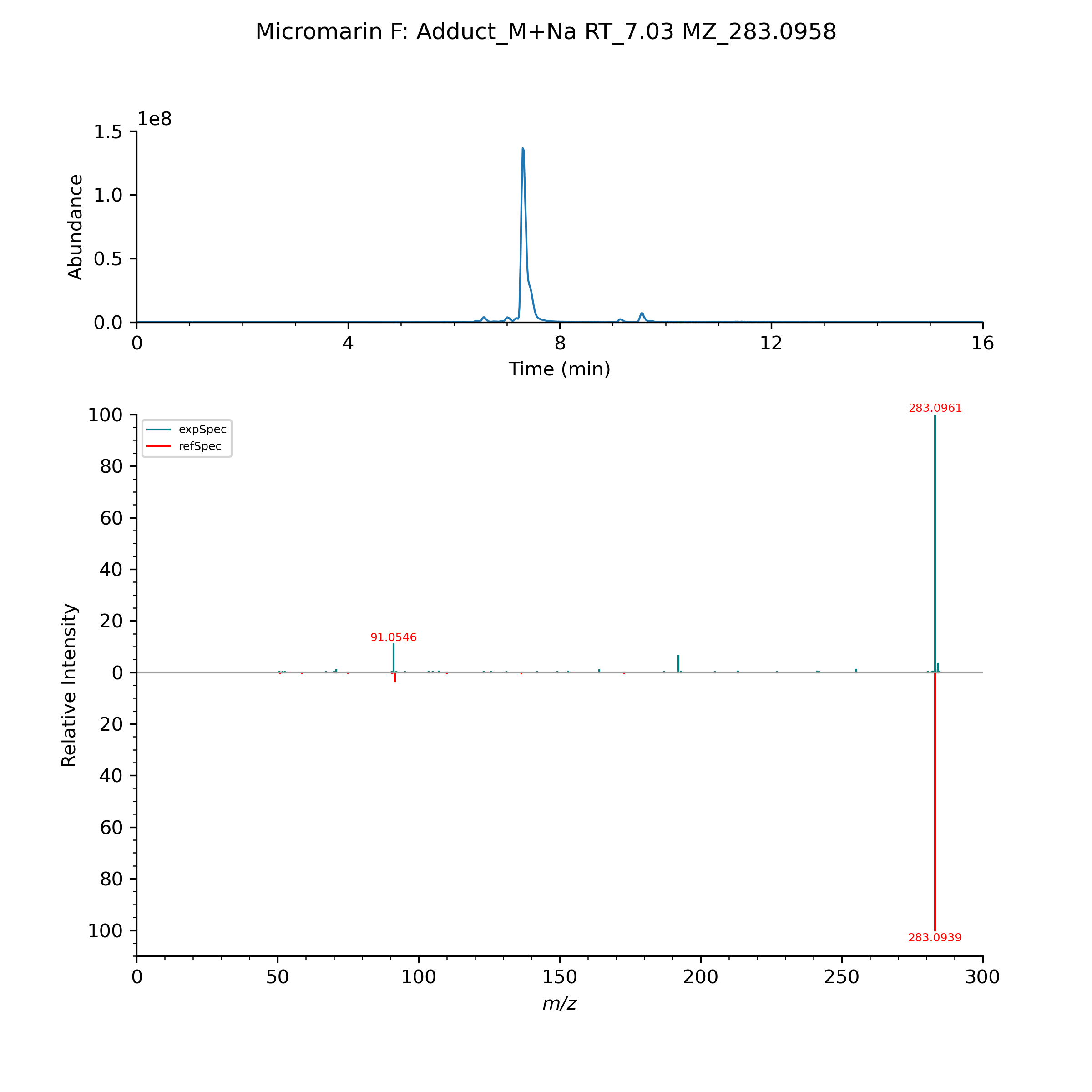

Supplement: Supplementary file 1 [file pharmaceuticals-18-01153-s001.zip › compound structures/M0057.png]

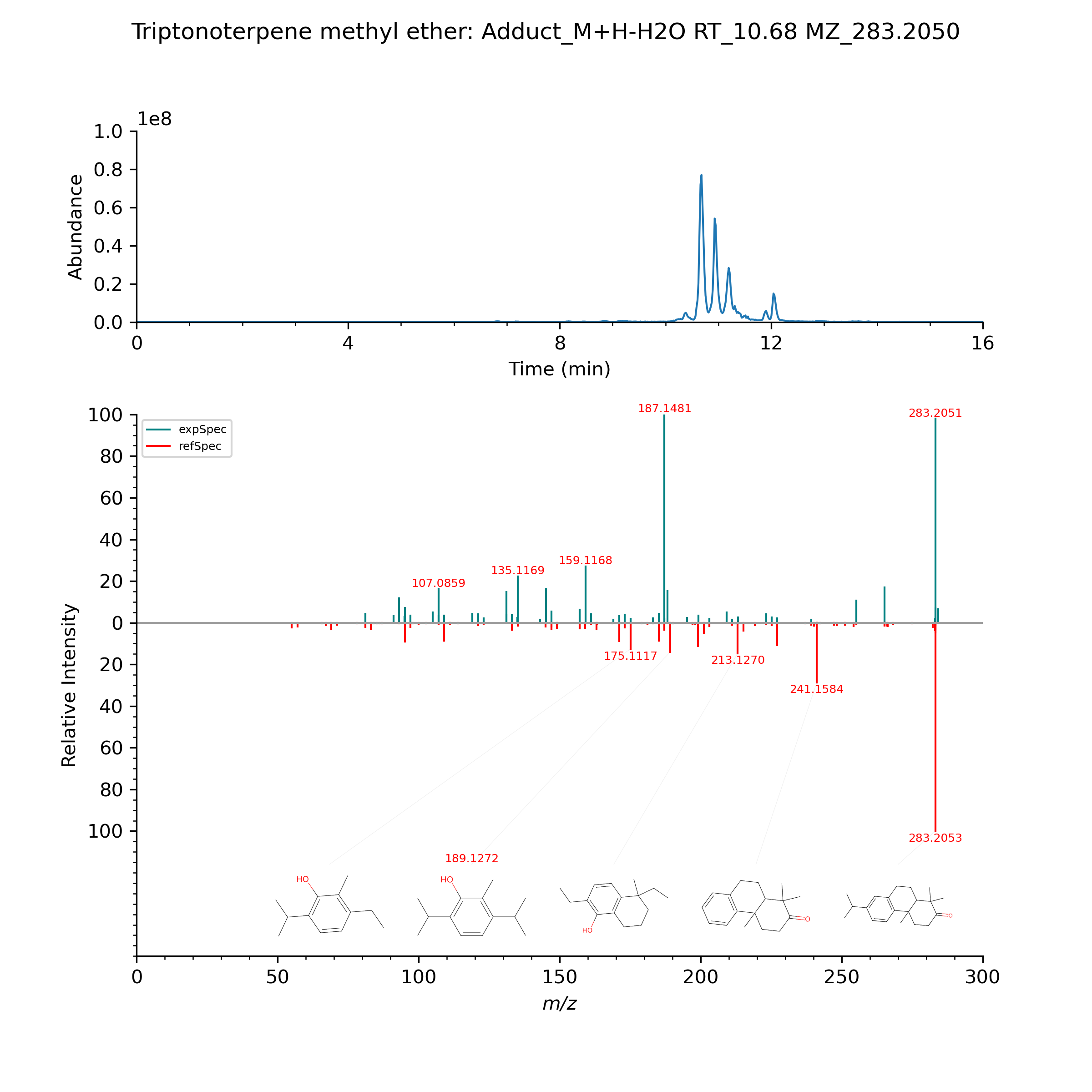

Supplement: Supplementary file 1 [file pharmaceuticals-18-01153-s001.zip › compound structures/M0058.png]

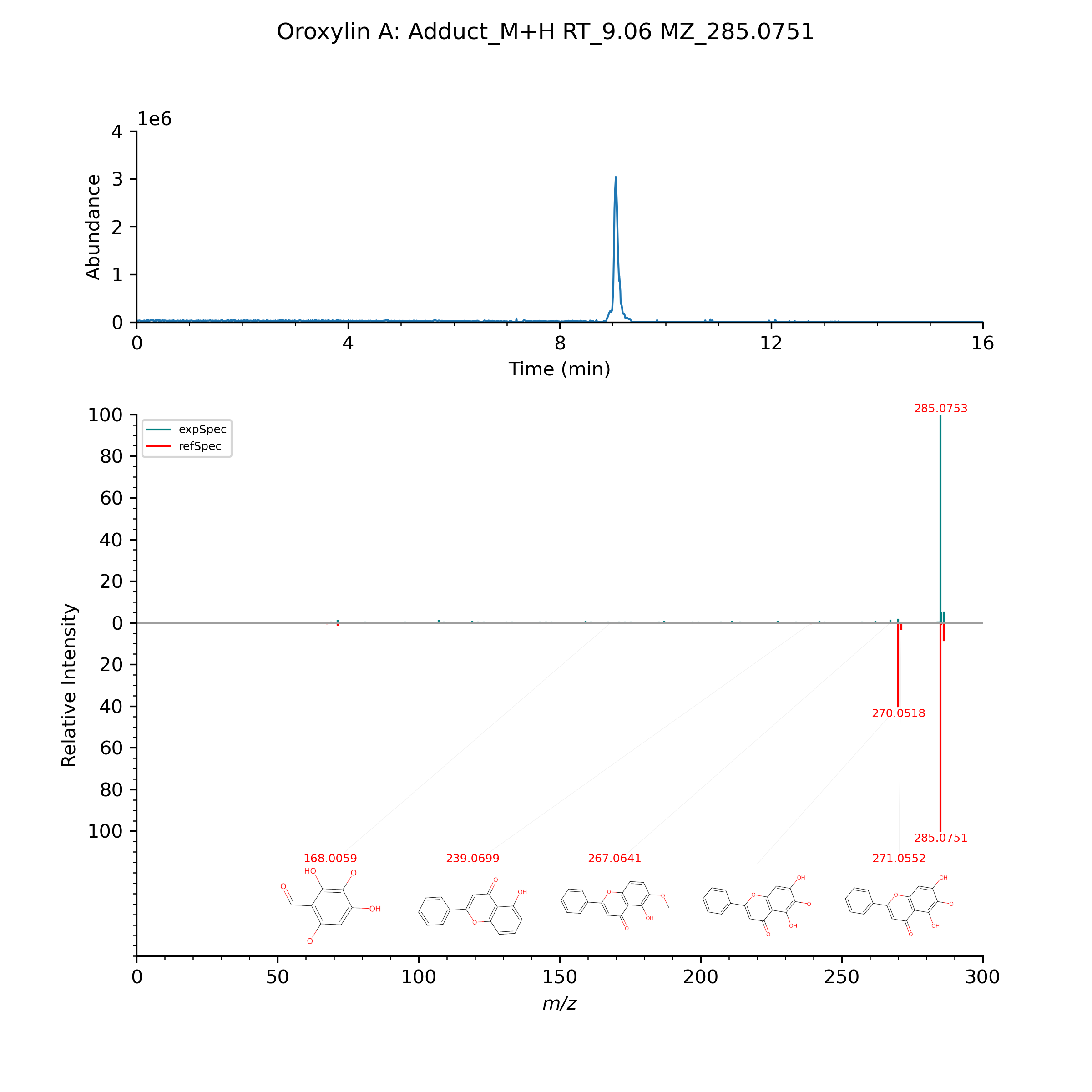

Supplement: Supplementary file 1 [file pharmaceuticals-18-01153-s001.zip › compound structures/M0059.png]

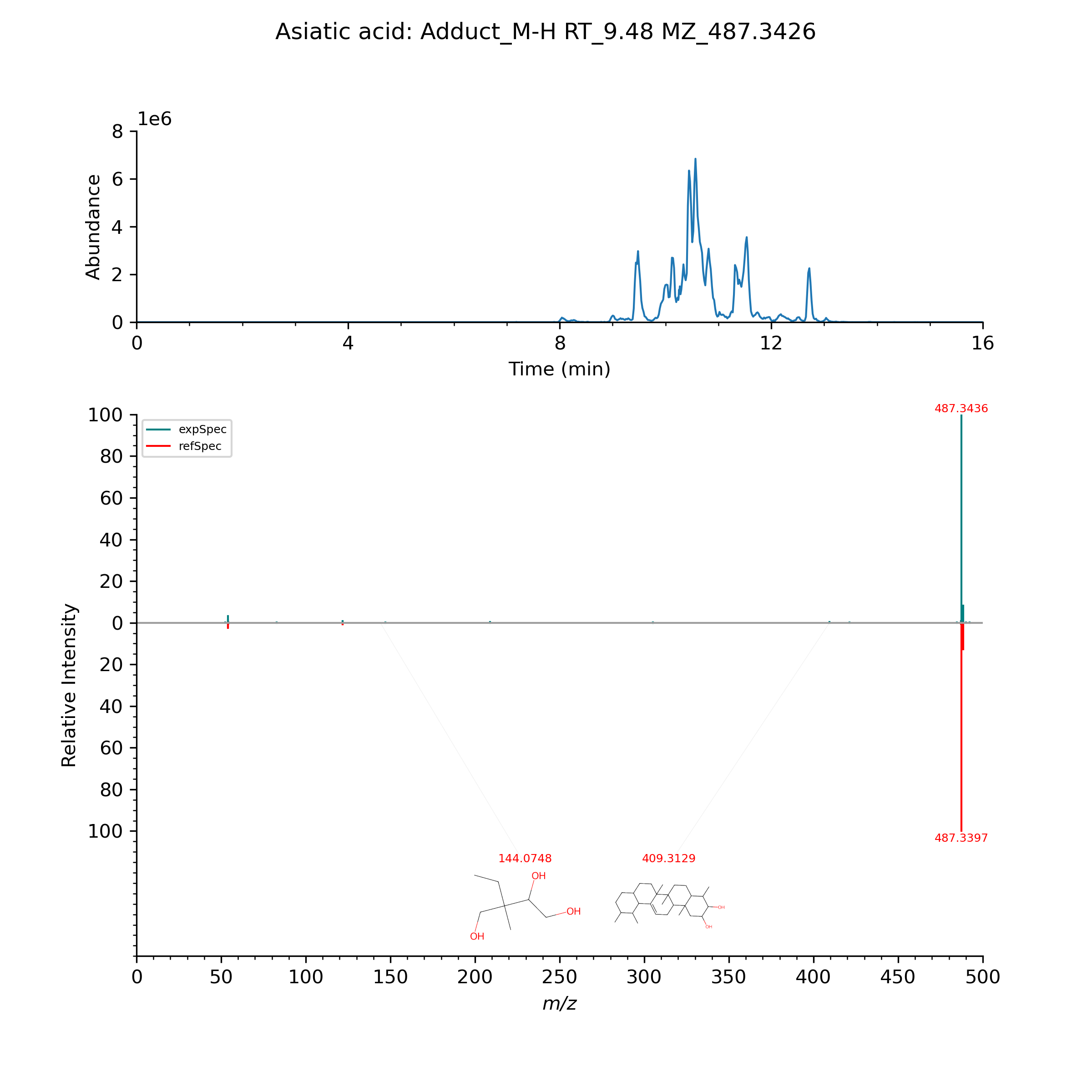

Supplement: Supplementary file 1 [file pharmaceuticals-18-01153-s001.zip › compound structures/M0060.png]

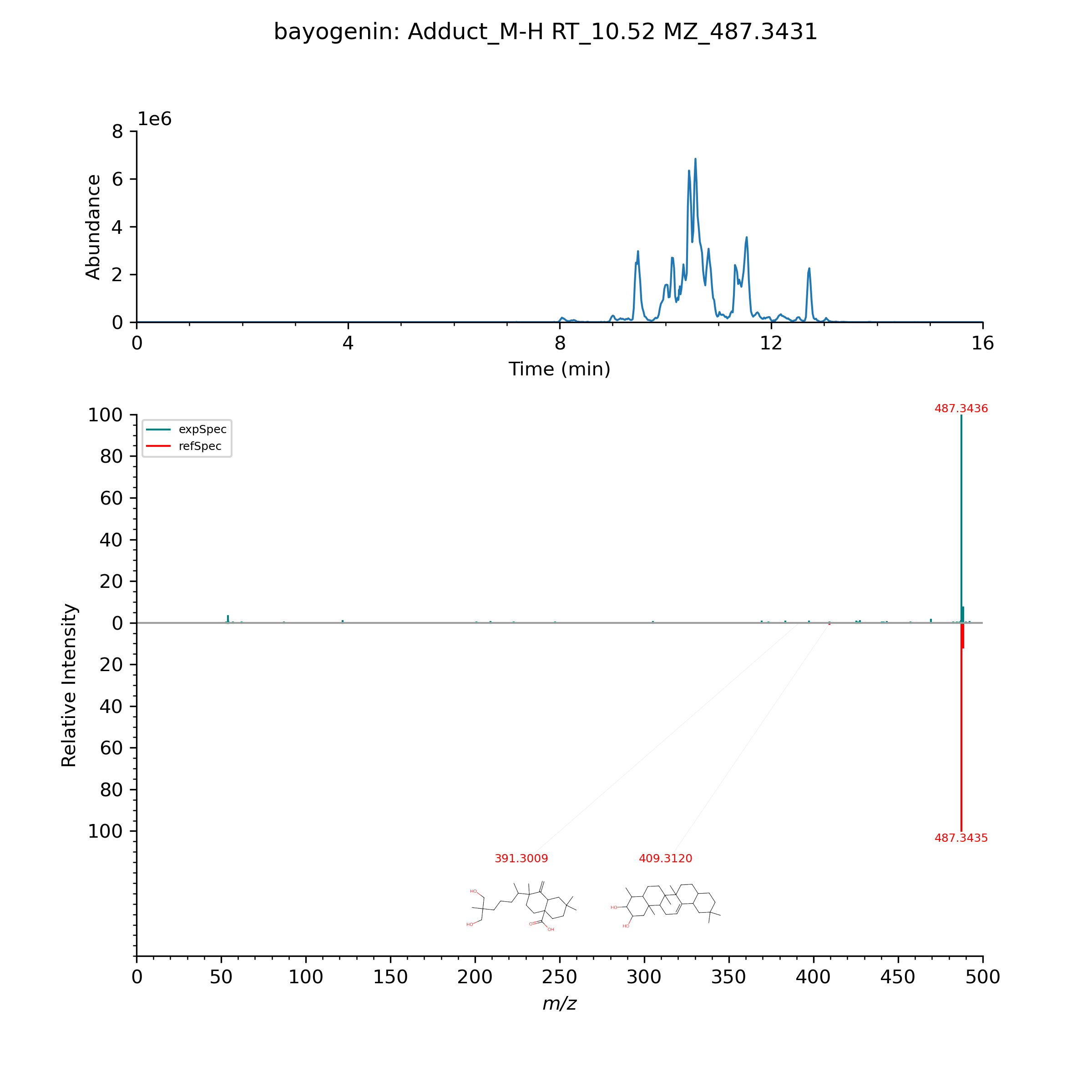

Supplement: Supplementary file 1 [file pharmaceuticals-18-01153-s001.zip › compound structures/M0061.png]

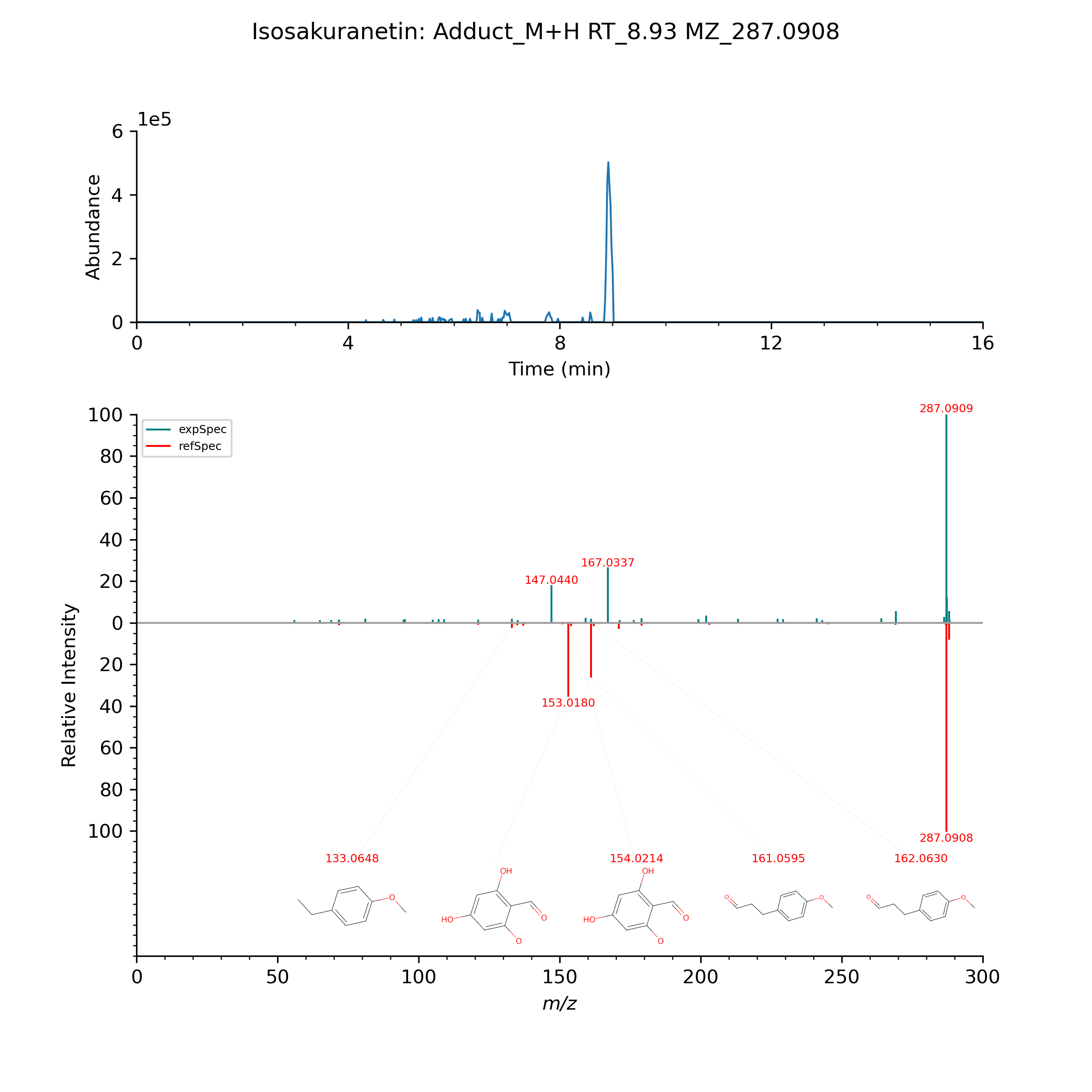

Supplement: Supplementary file 1 [file pharmaceuticals-18-01153-s001.zip › compound structures/M0062.png]

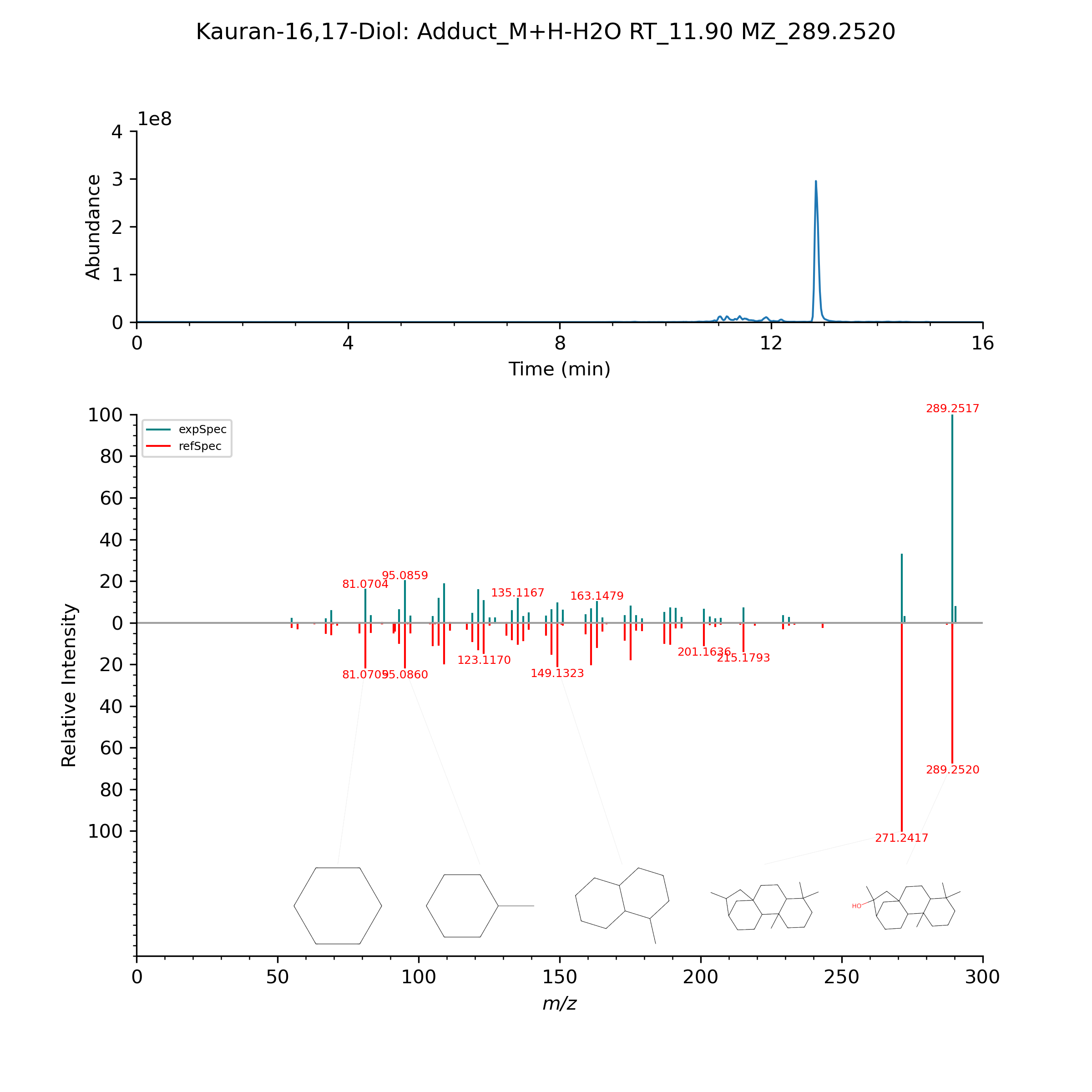

Supplement: Supplementary file 1 [file pharmaceuticals-18-01153-s001.zip › compound structures/M0063.png]

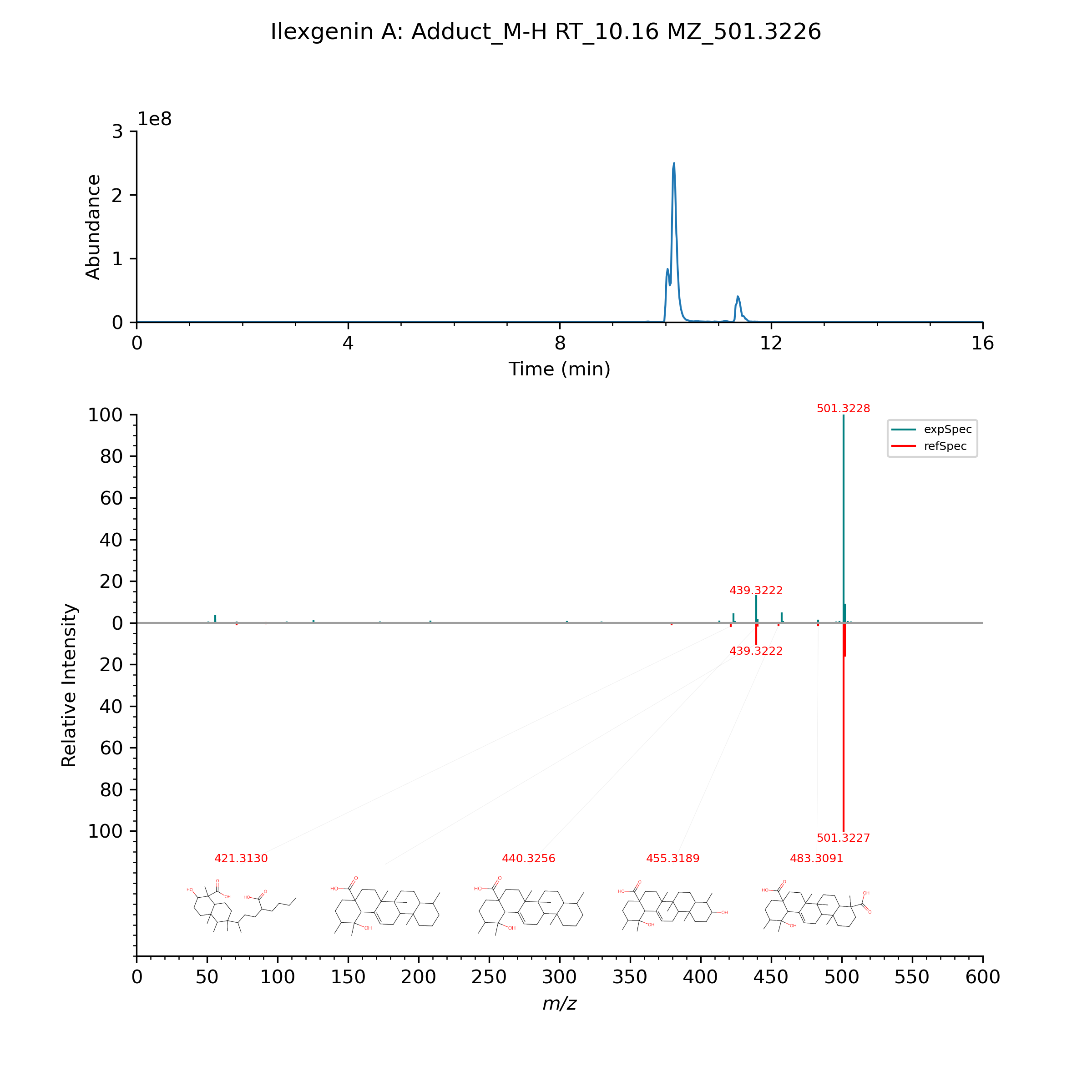

Supplement: Supplementary file 1 [file pharmaceuticals-18-01153-s001.zip › compound structures/M0064.png]

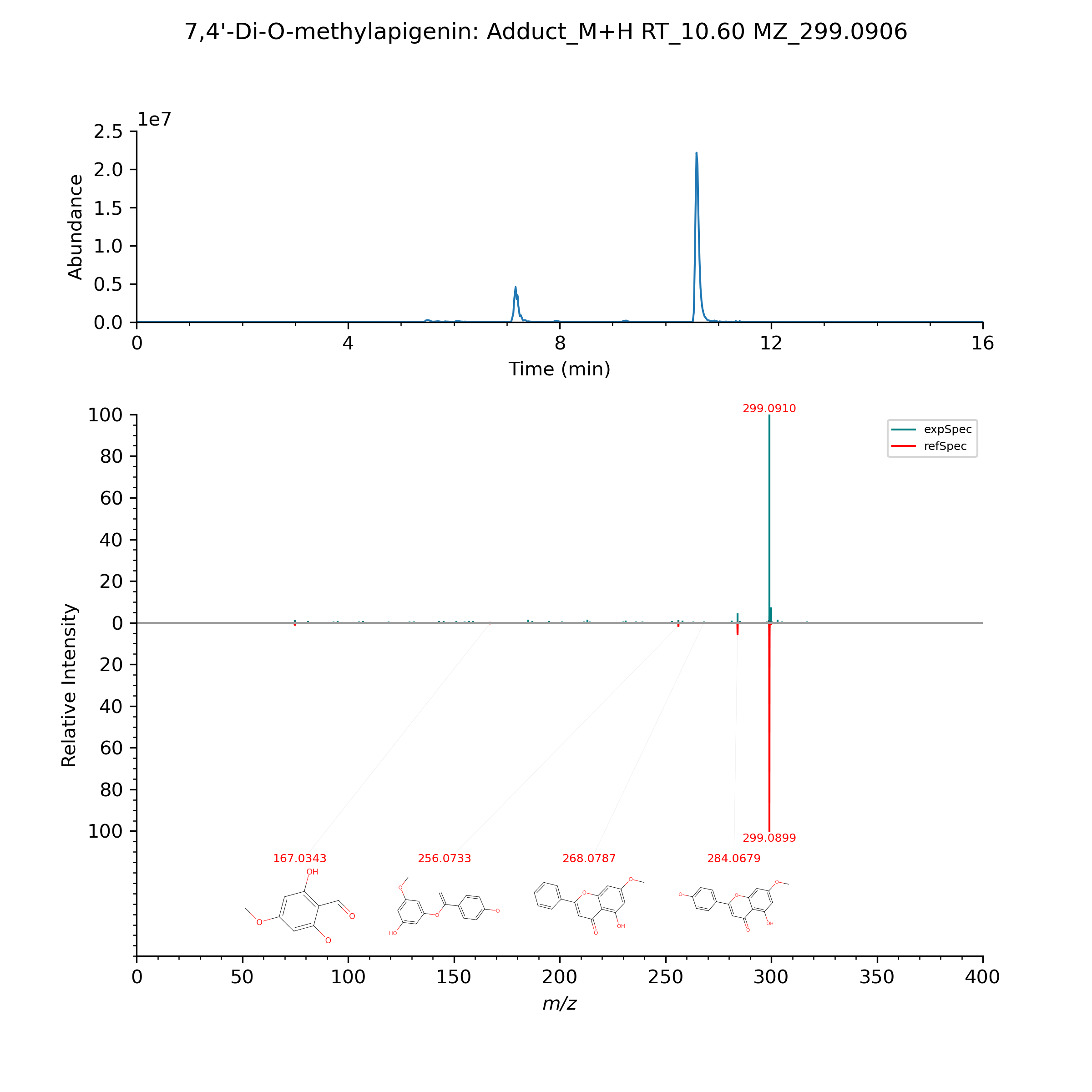

Supplement: Supplementary file 1 [file pharmaceuticals-18-01153-s001.zip › compound structures/M0065.png]

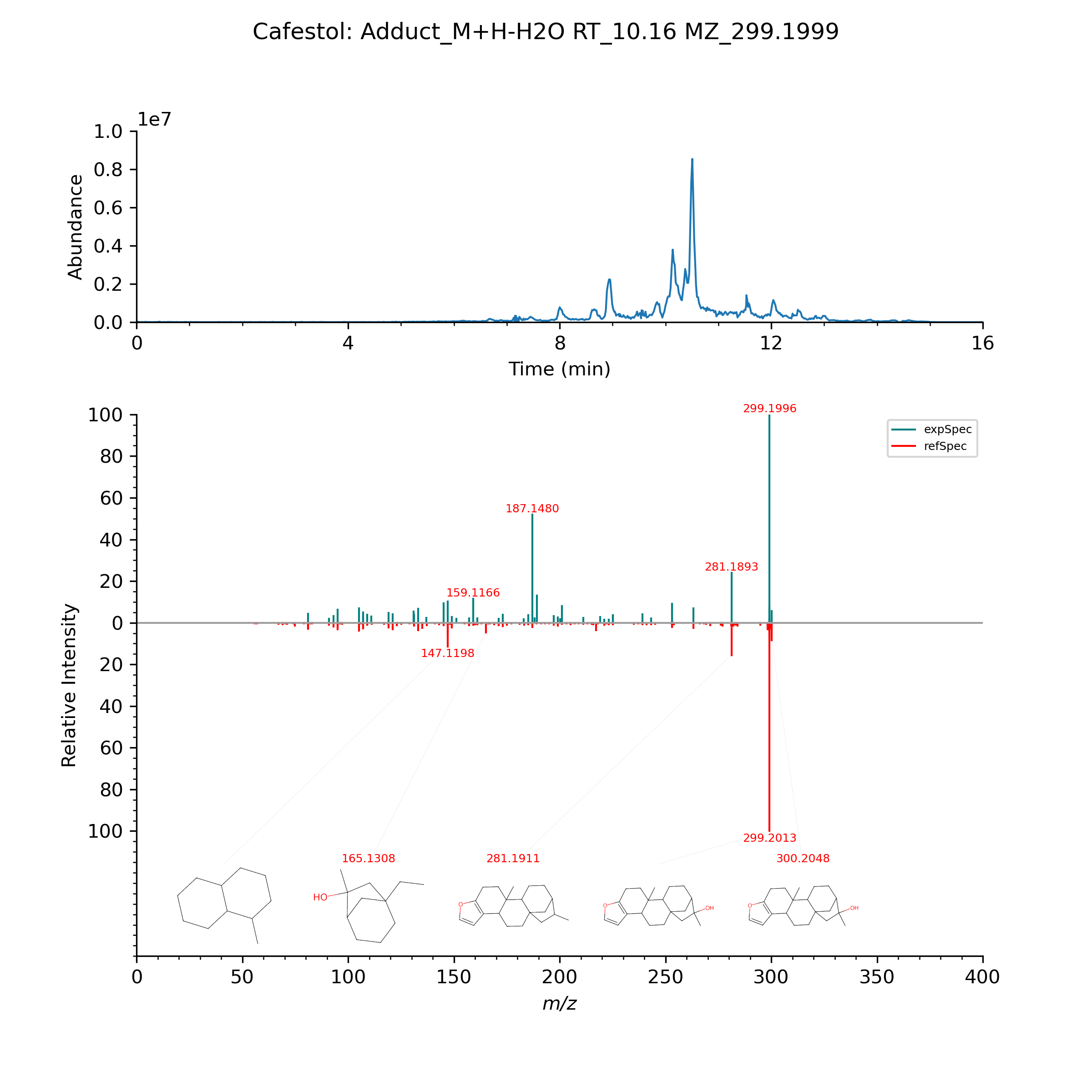

Supplement: Supplementary file 1 [file pharmaceuticals-18-01153-s001.zip › compound structures/M0066.png]

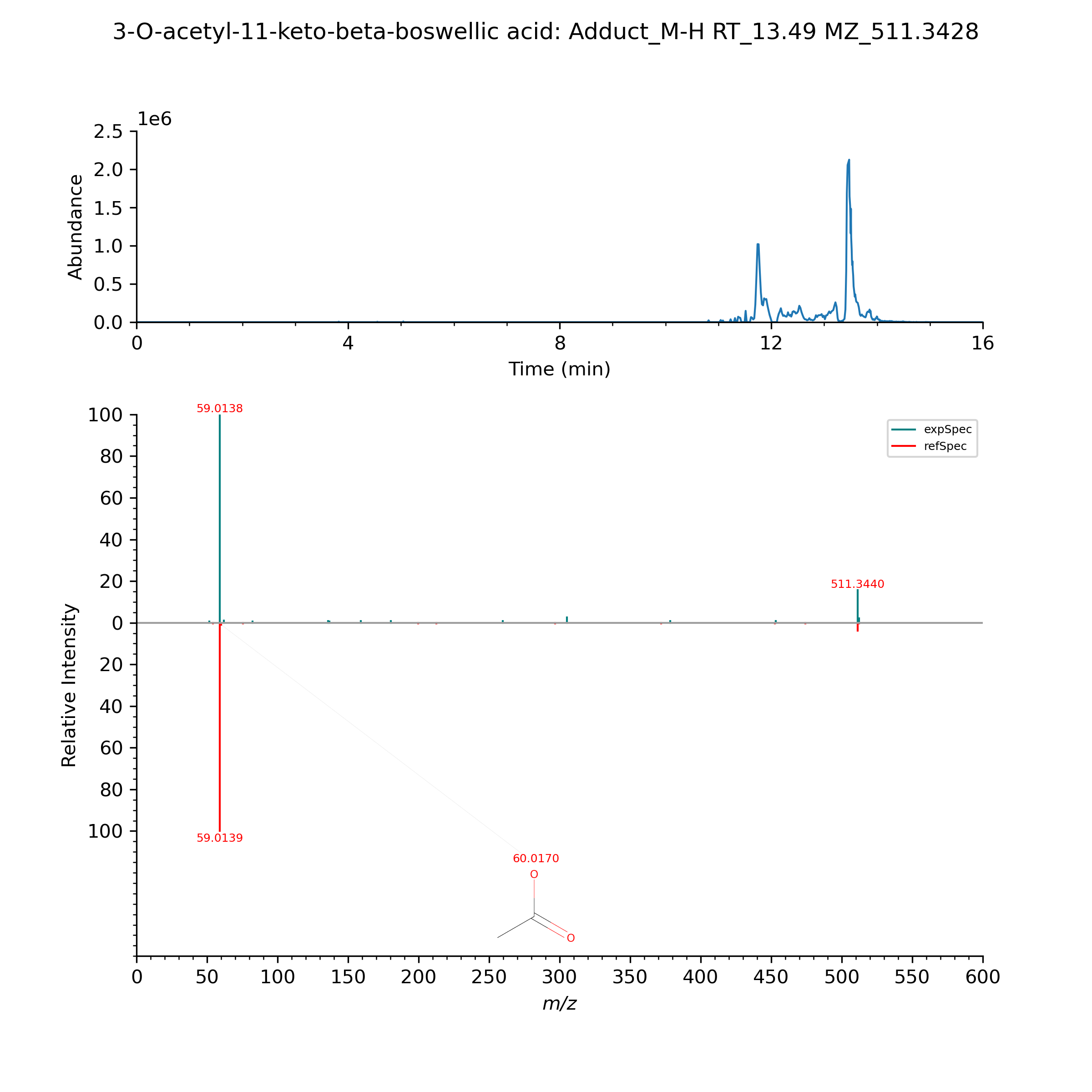

Supplement: Supplementary file 1 [file pharmaceuticals-18-01153-s001.zip › compound structures/M0067.png]

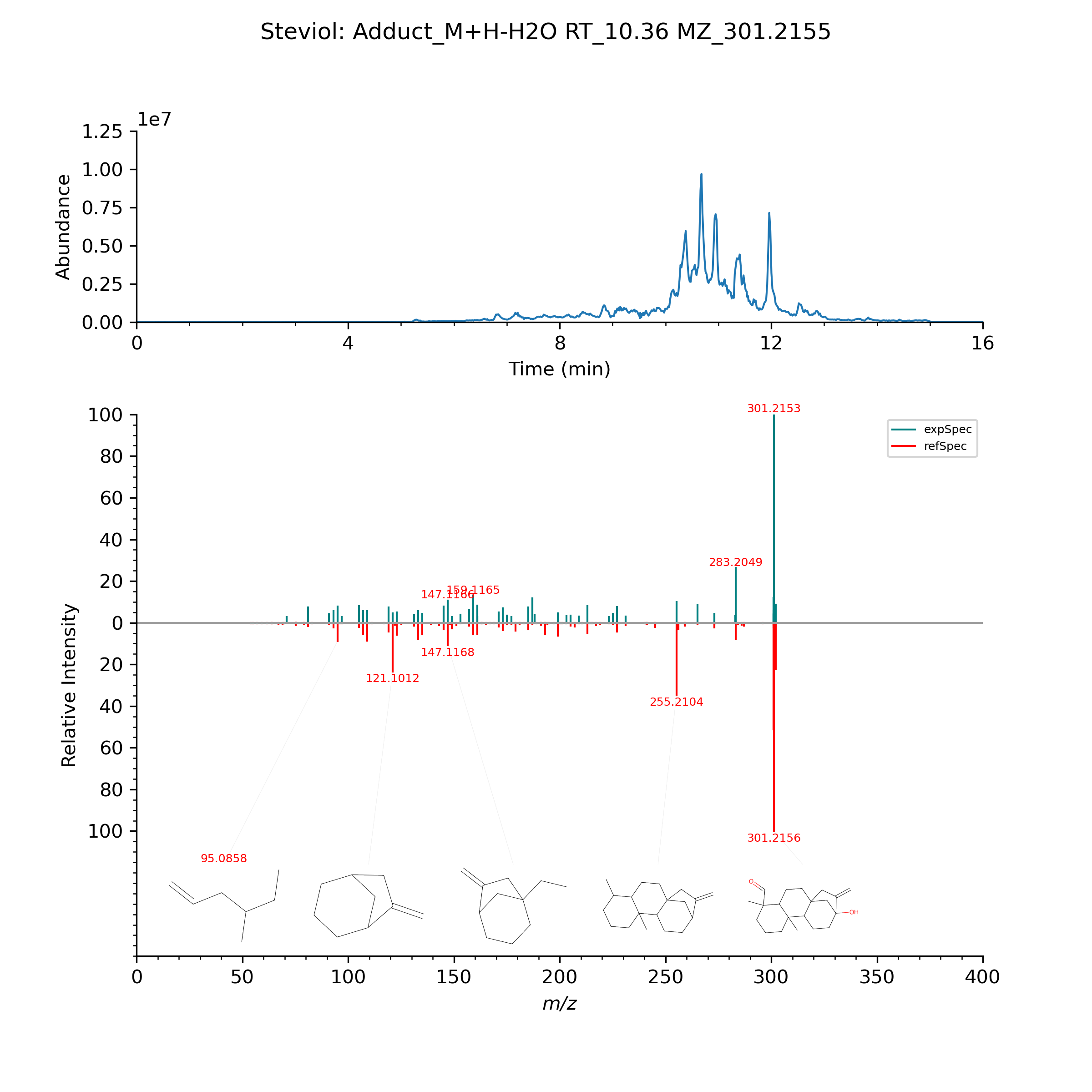

Supplement: Supplementary file 1 [file pharmaceuticals-18-01153-s001.zip › compound structures/M0068.png]

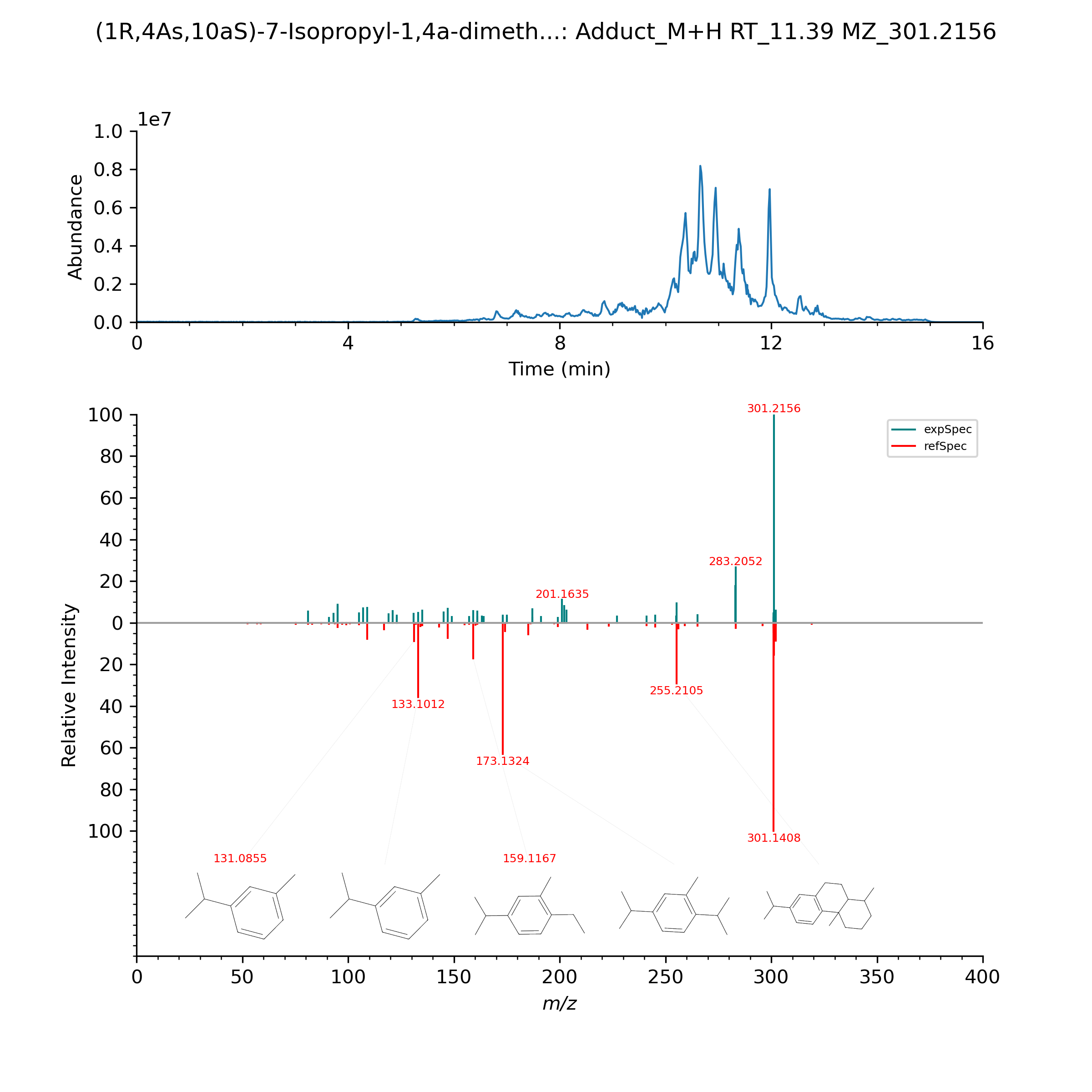

Supplement: Supplementary file 1 [file pharmaceuticals-18-01153-s001.zip › compound structures/M0069.png]

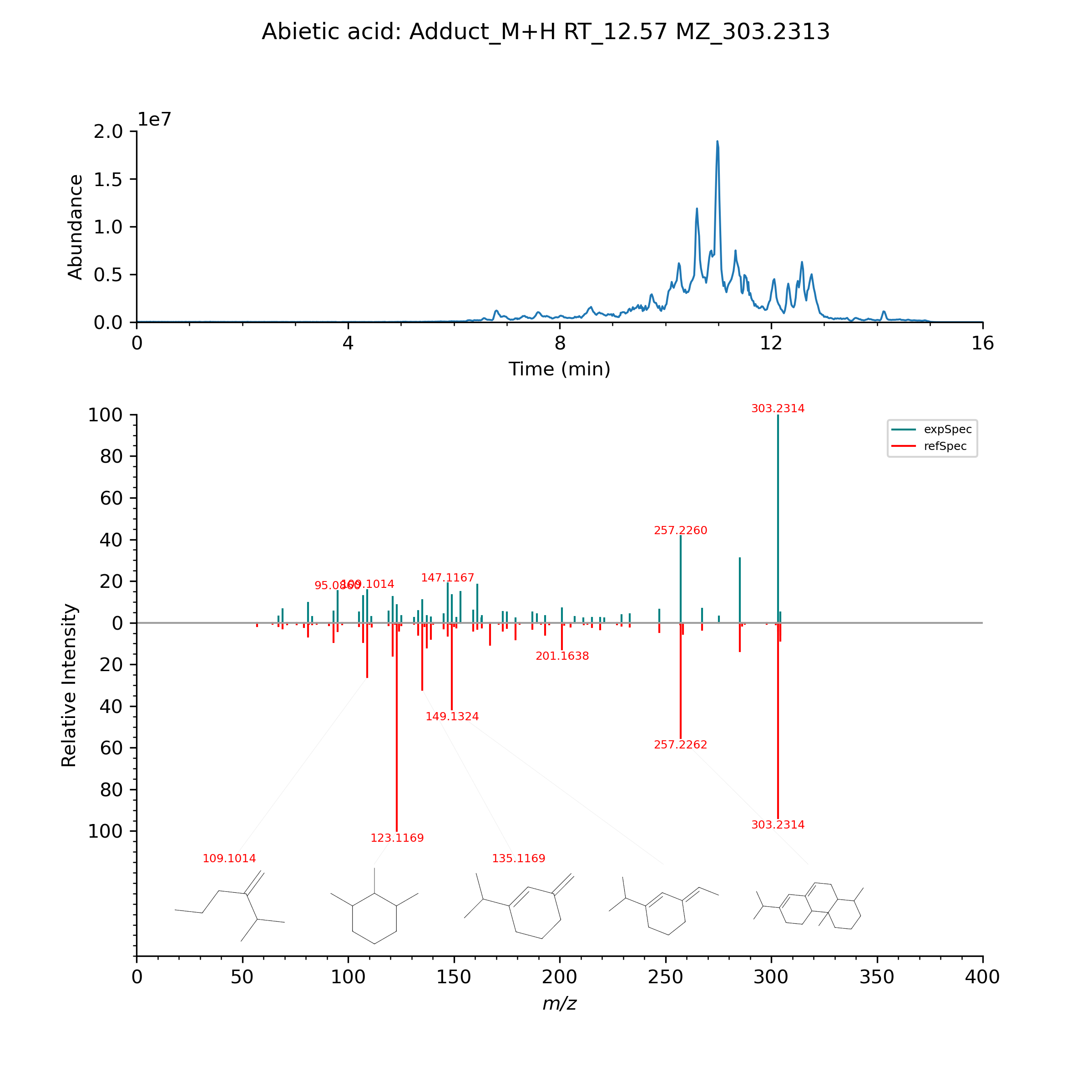

Supplement: Supplementary file 1 [file pharmaceuticals-18-01153-s001.zip › compound structures/M0070.png]

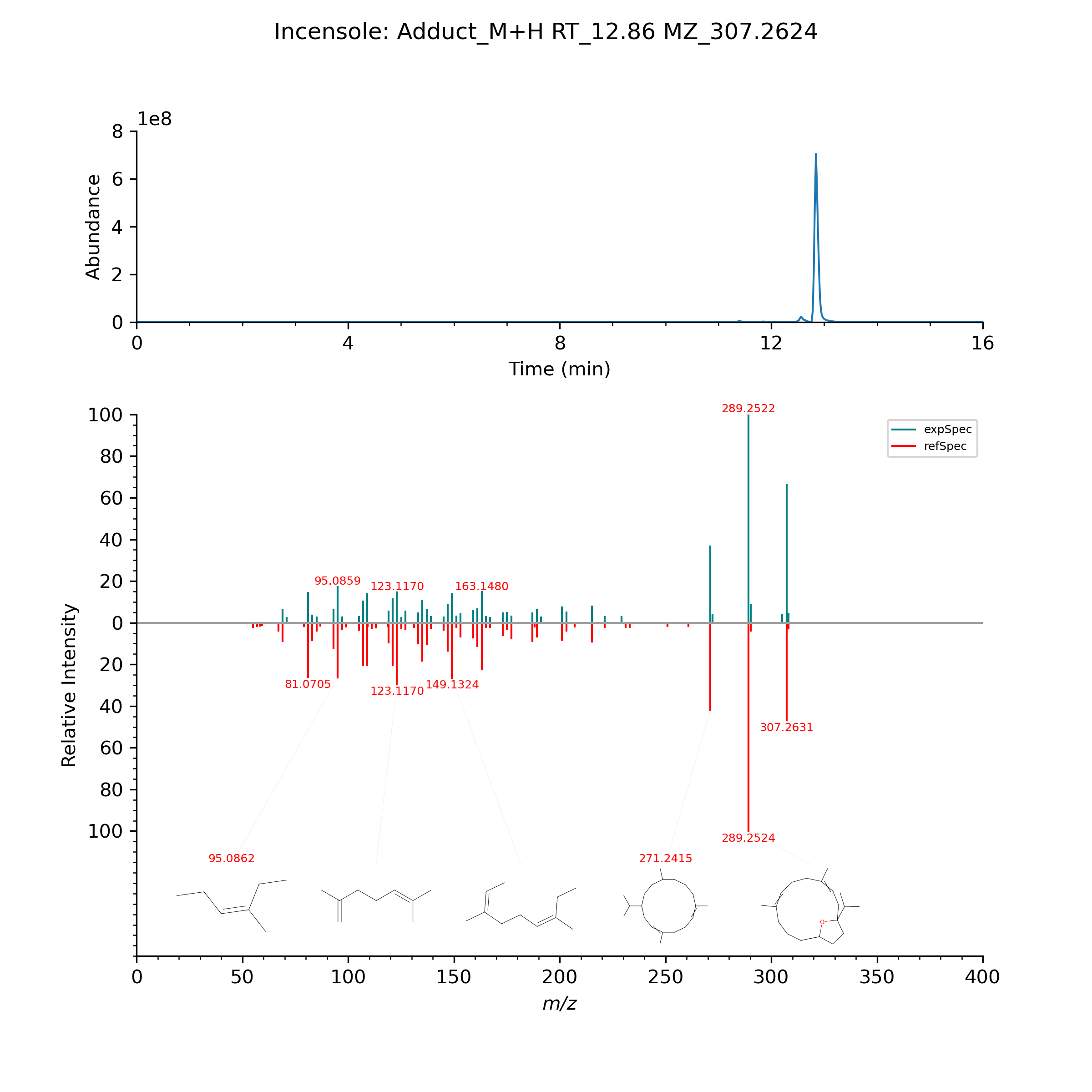

Supplement: Supplementary file 1 [file pharmaceuticals-18-01153-s001.zip › compound structures/M0071.png]

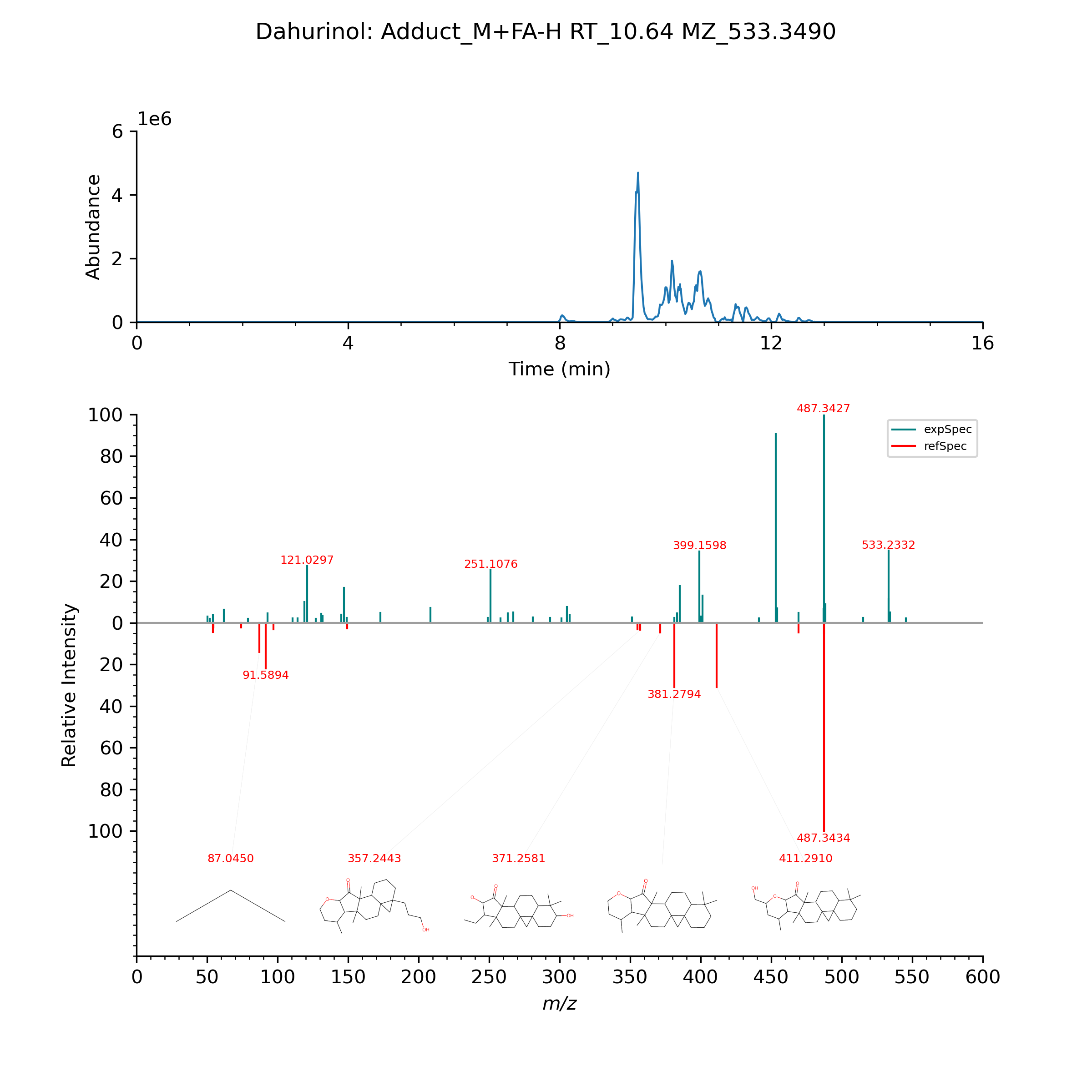

Supplement: Supplementary file 1 [file pharmaceuticals-18-01153-s001.zip › compound structures/M0072.png]

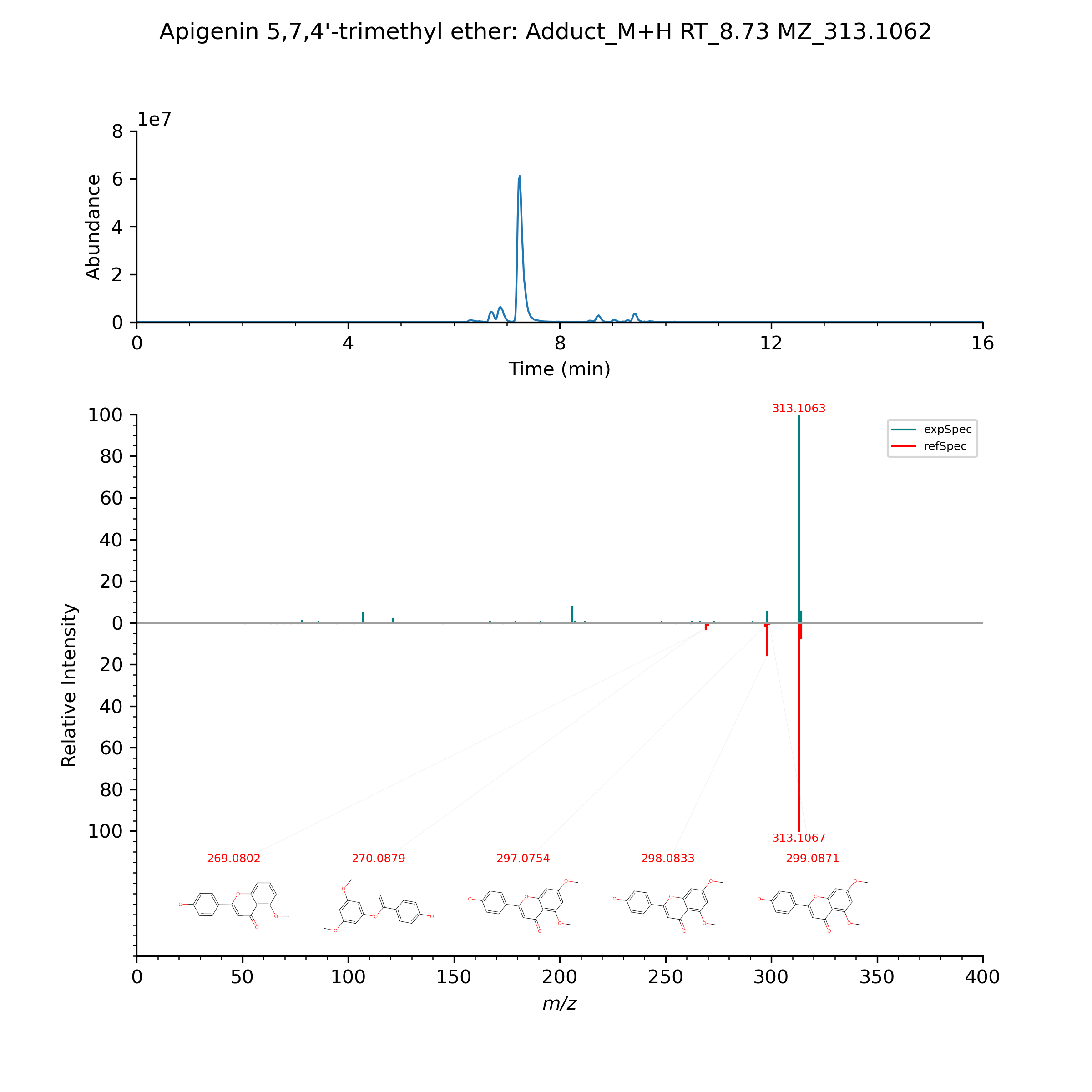

Supplement: Supplementary file 1 [file pharmaceuticals-18-01153-s001.zip › compound structures/M0073.png]

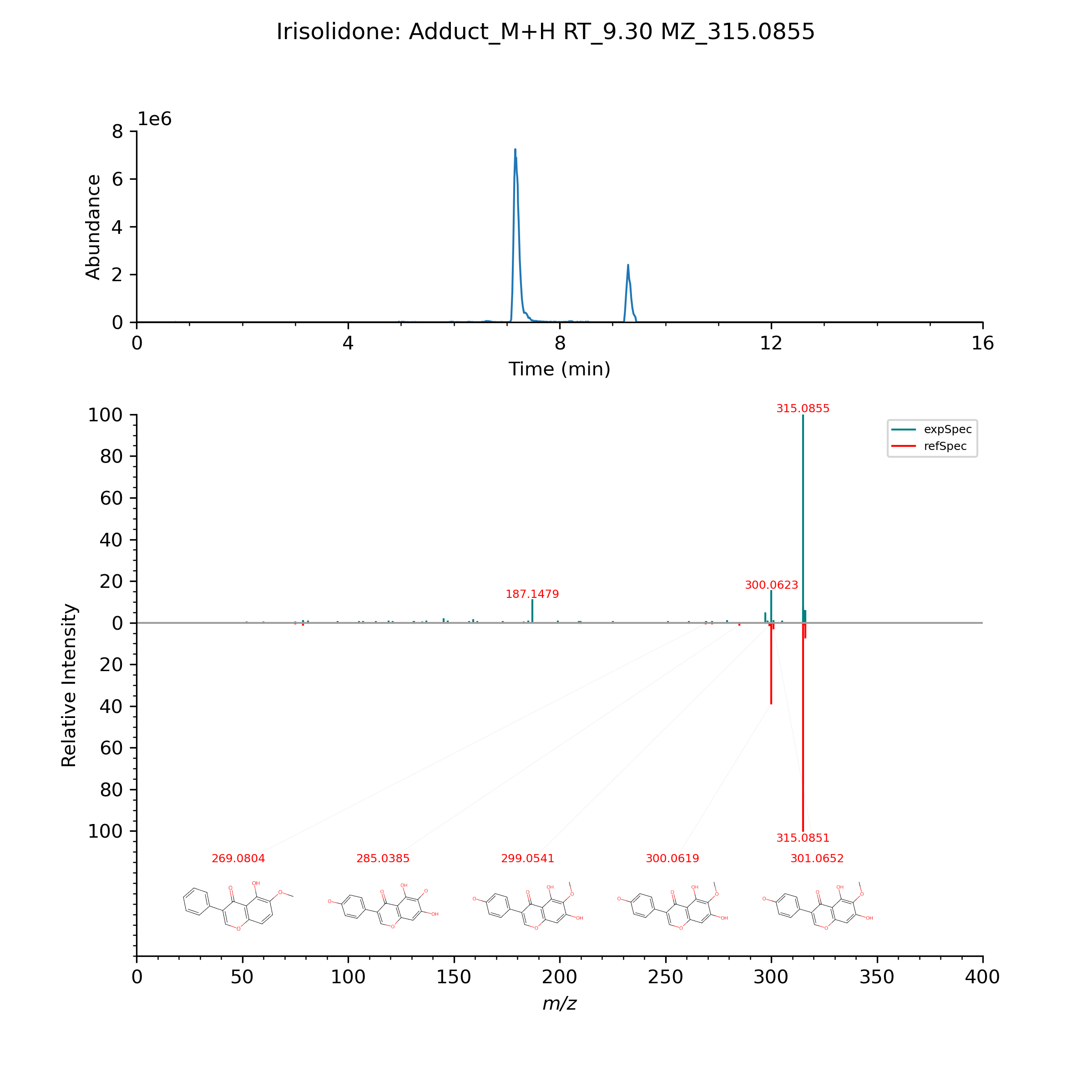

Supplement: Supplementary file 1 [file pharmaceuticals-18-01153-s001.zip › compound structures/M0074.png]

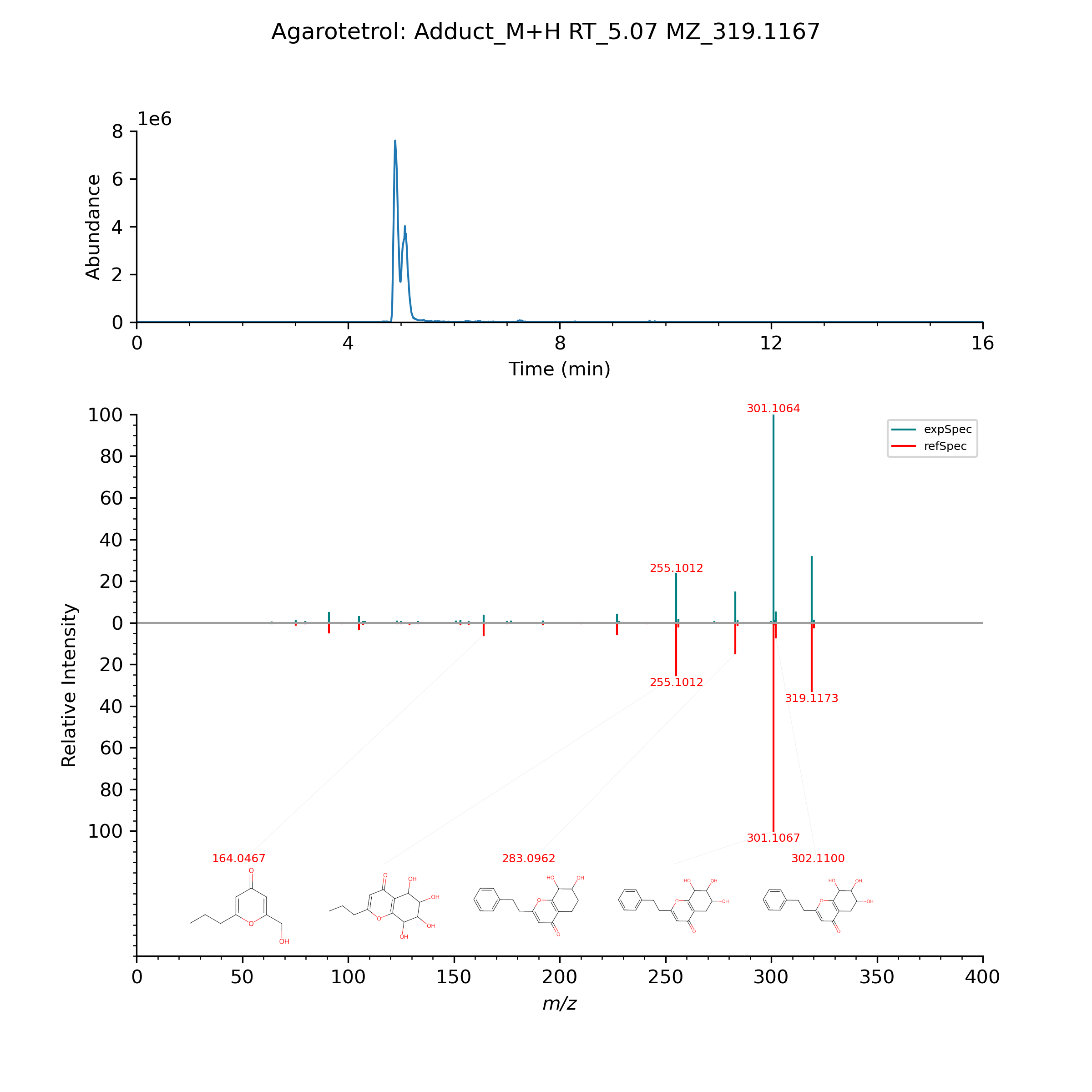

Supplement: Supplementary file 1 [file pharmaceuticals-18-01153-s001.zip › compound structures/M0075.png]

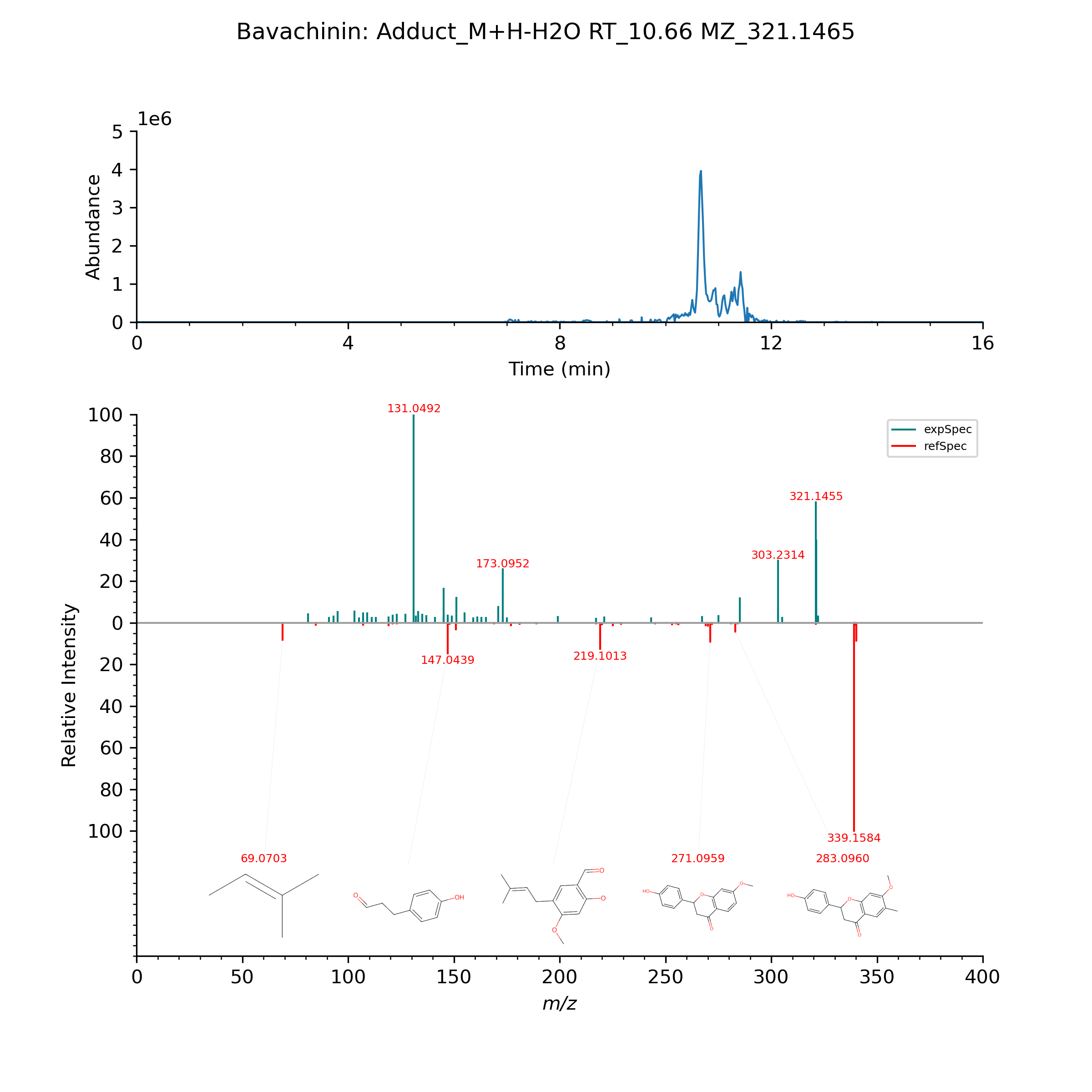

Supplement: Supplementary file 1 [file pharmaceuticals-18-01153-s001.zip › compound structures/M0076.png]

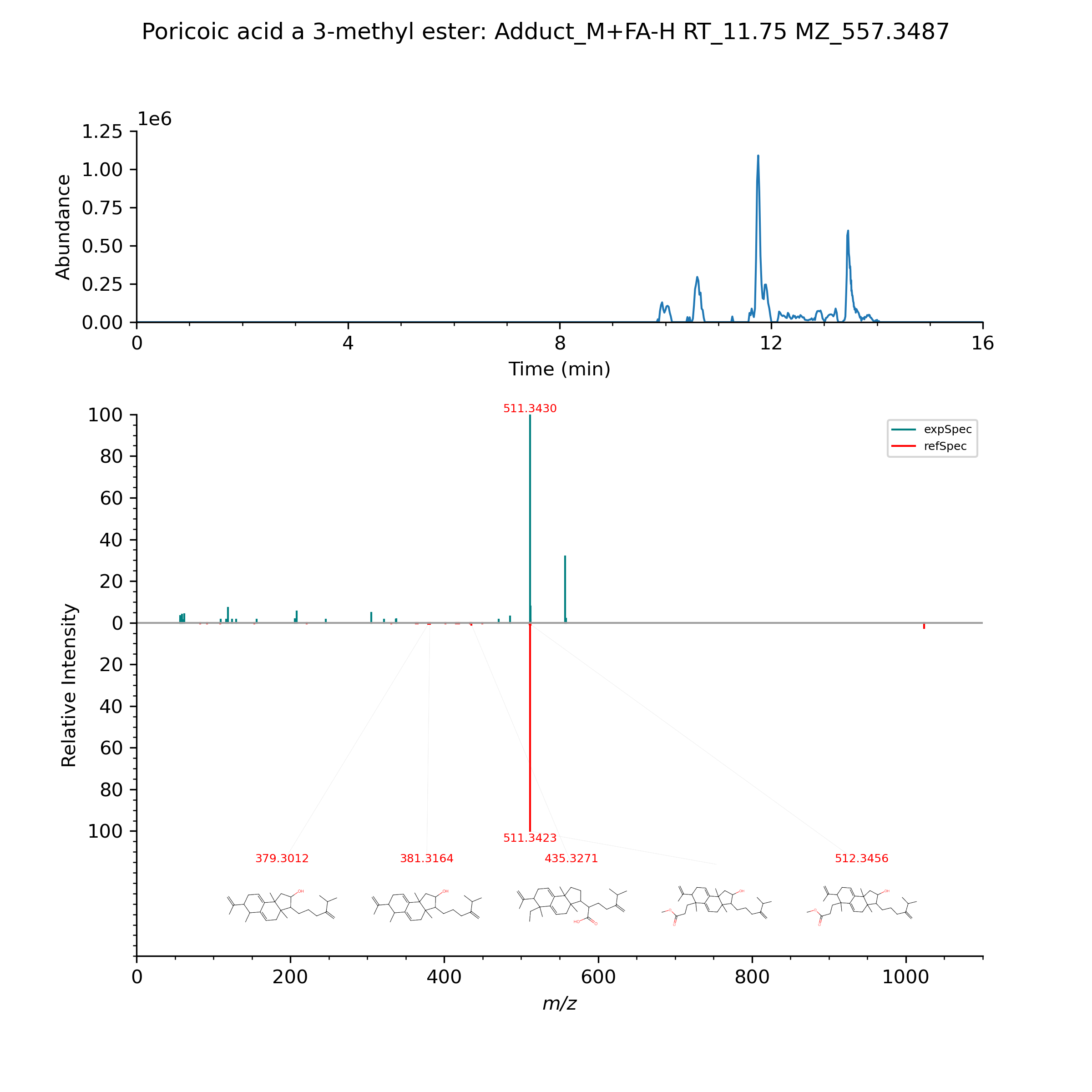

Supplement: Supplementary file 1 [file pharmaceuticals-18-01153-s001.zip › compound structures/M0077.png]

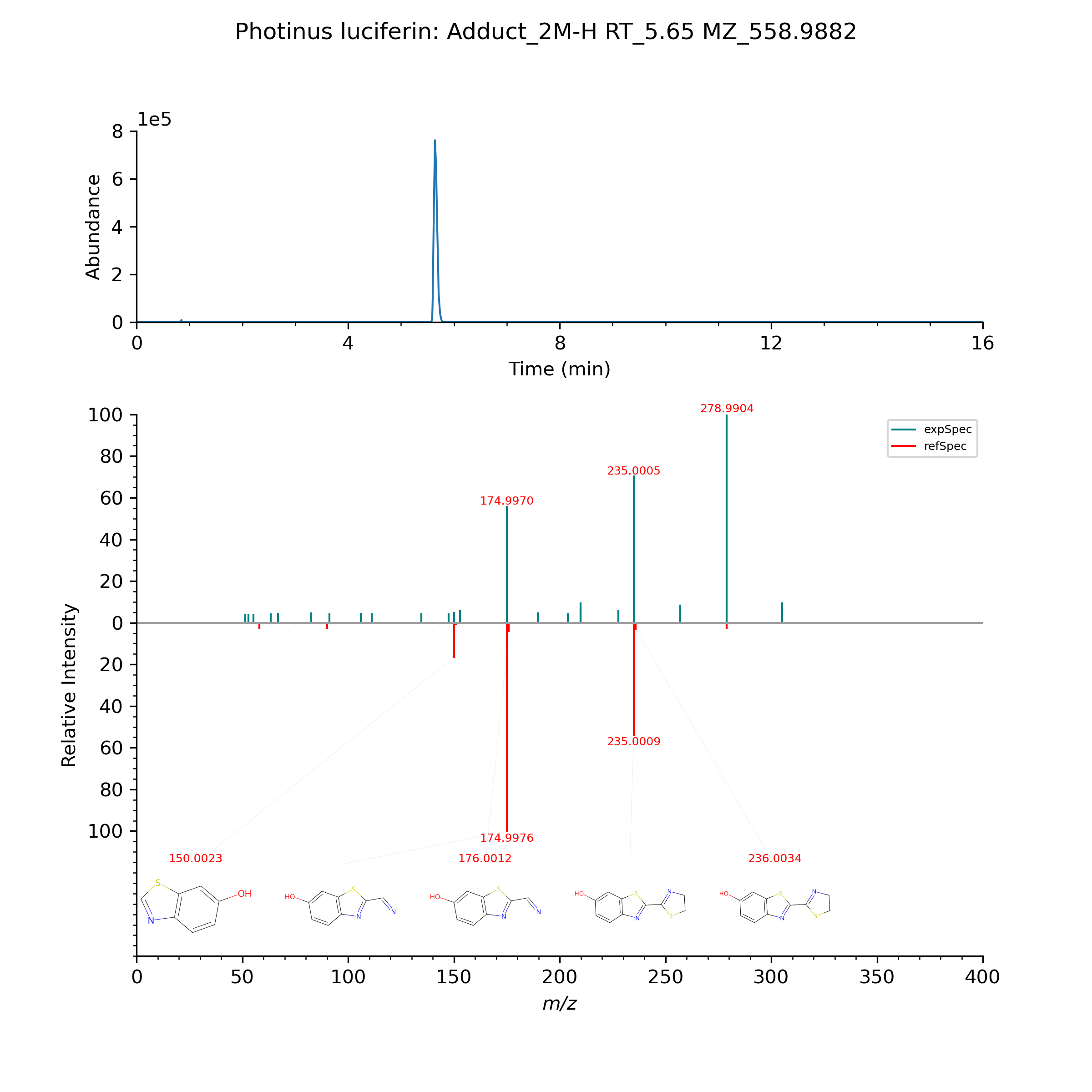

Supplement: Supplementary file 1 [file pharmaceuticals-18-01153-s001.zip › compound structures/M0078.png]

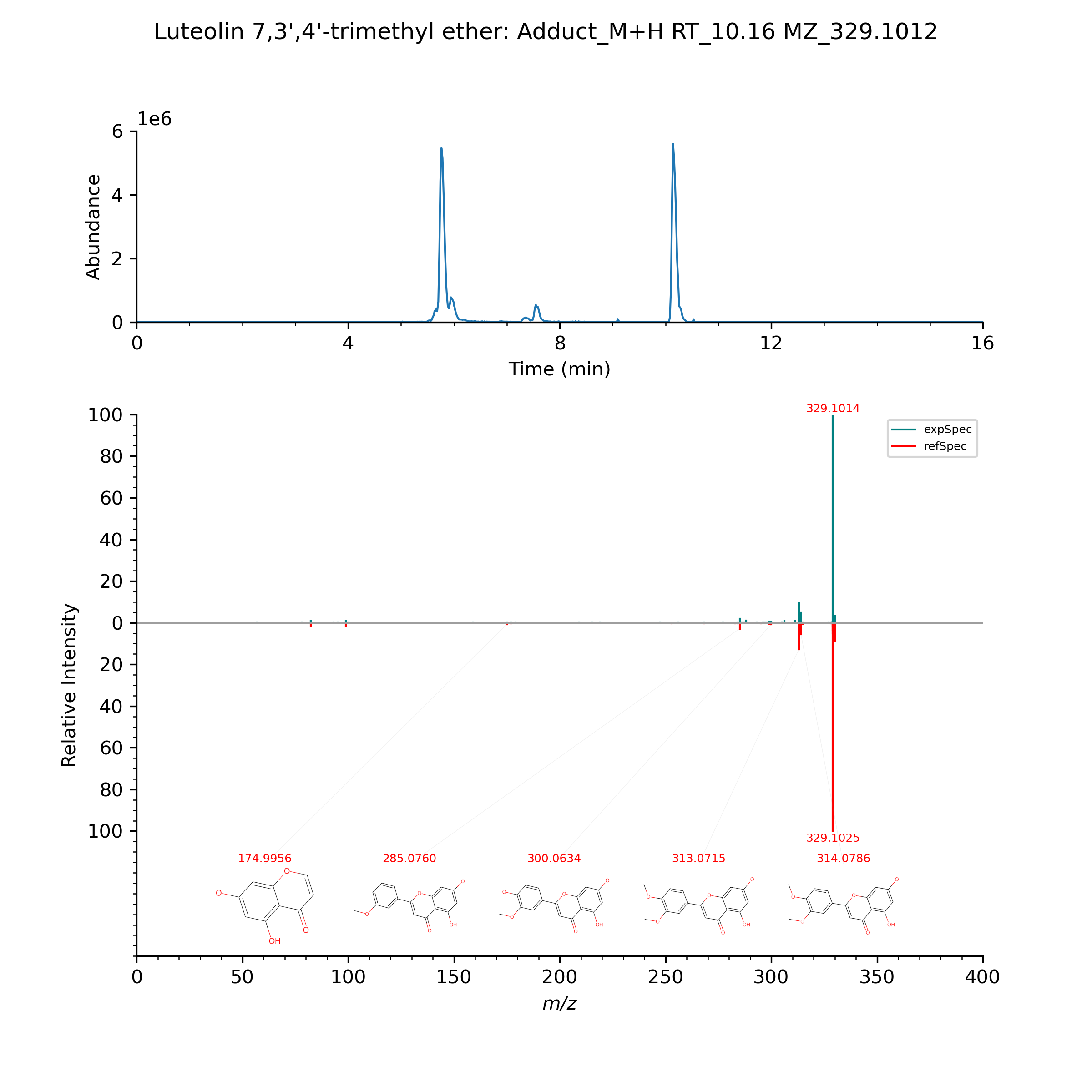

Supplement: Supplementary file 1 [file pharmaceuticals-18-01153-s001.zip › compound structures/M0079.png]

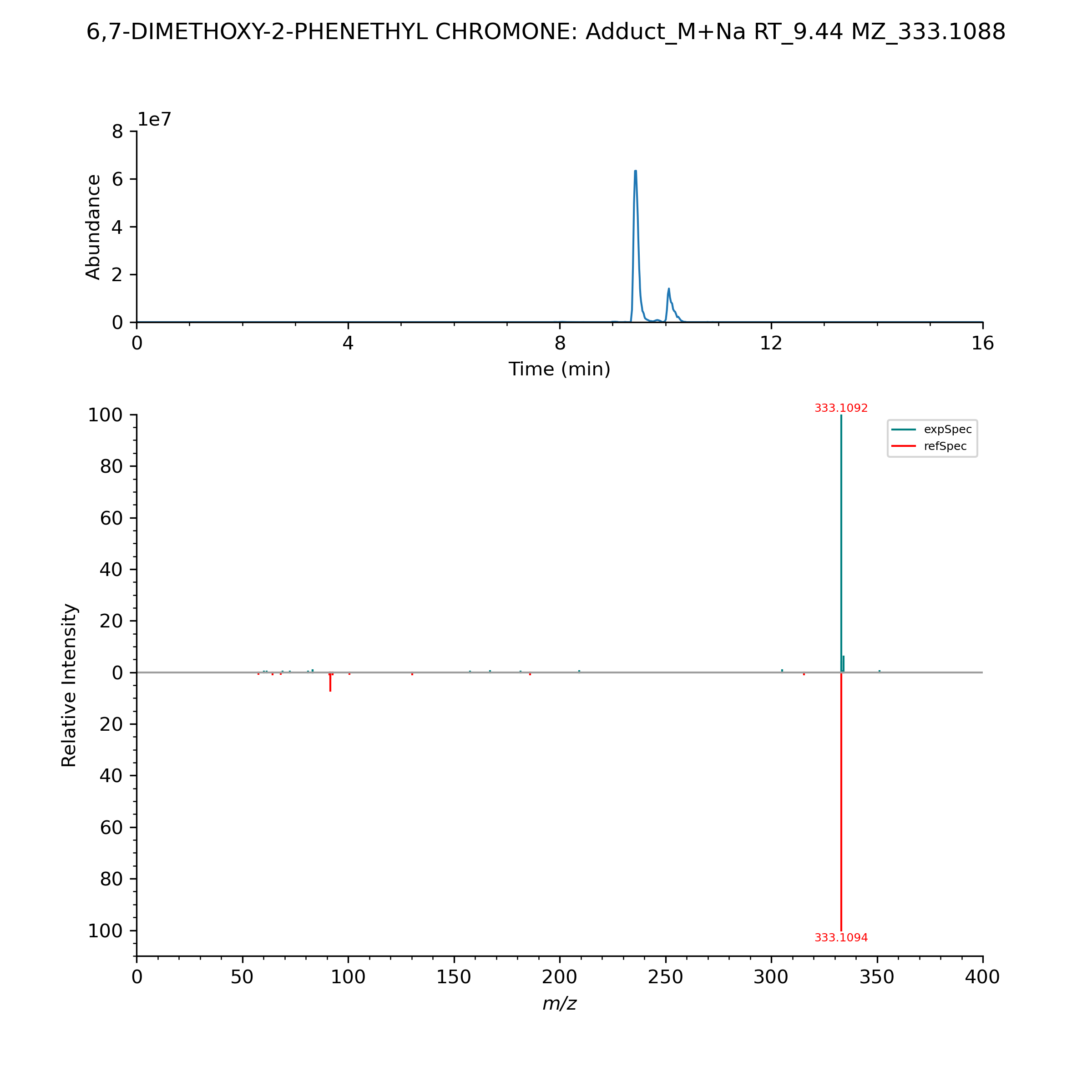

Supplement: Supplementary file 1 [file pharmaceuticals-18-01153-s001.zip › compound structures/M0080.png]

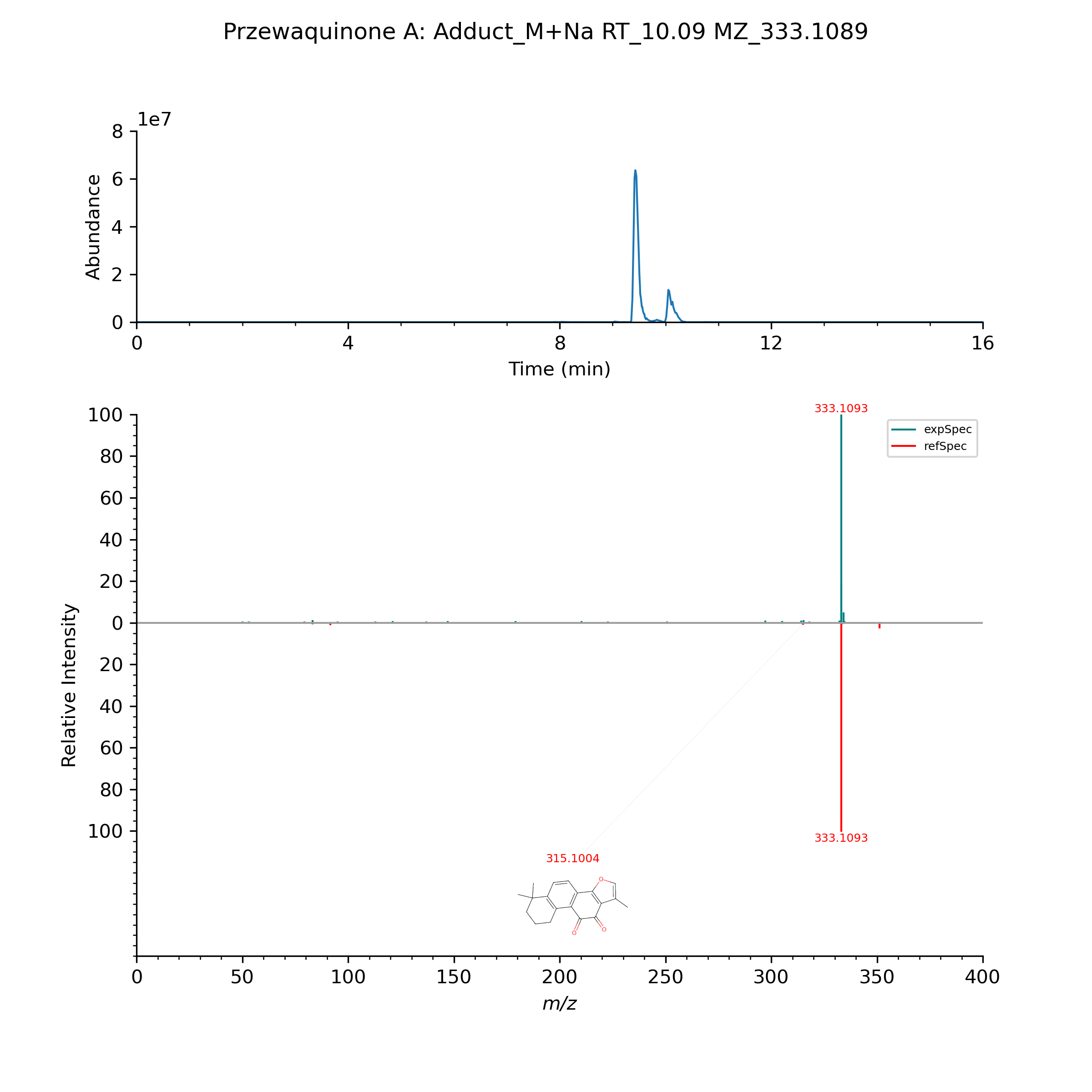

Supplement: Supplementary file 1 [file pharmaceuticals-18-01153-s001.zip › compound structures/M0081.png]

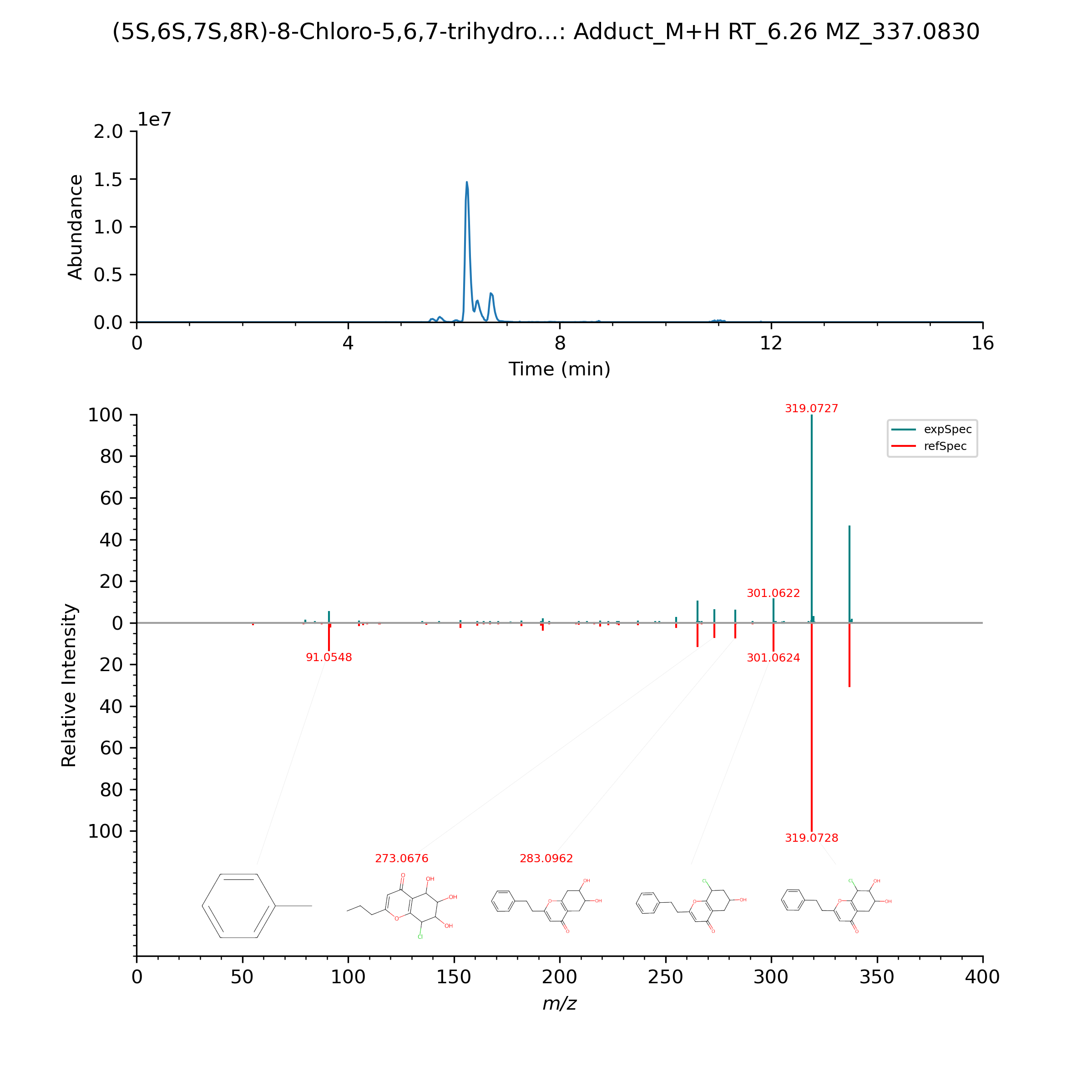

Supplement: Supplementary file 1 [file pharmaceuticals-18-01153-s001.zip › compound structures/M0082.png]

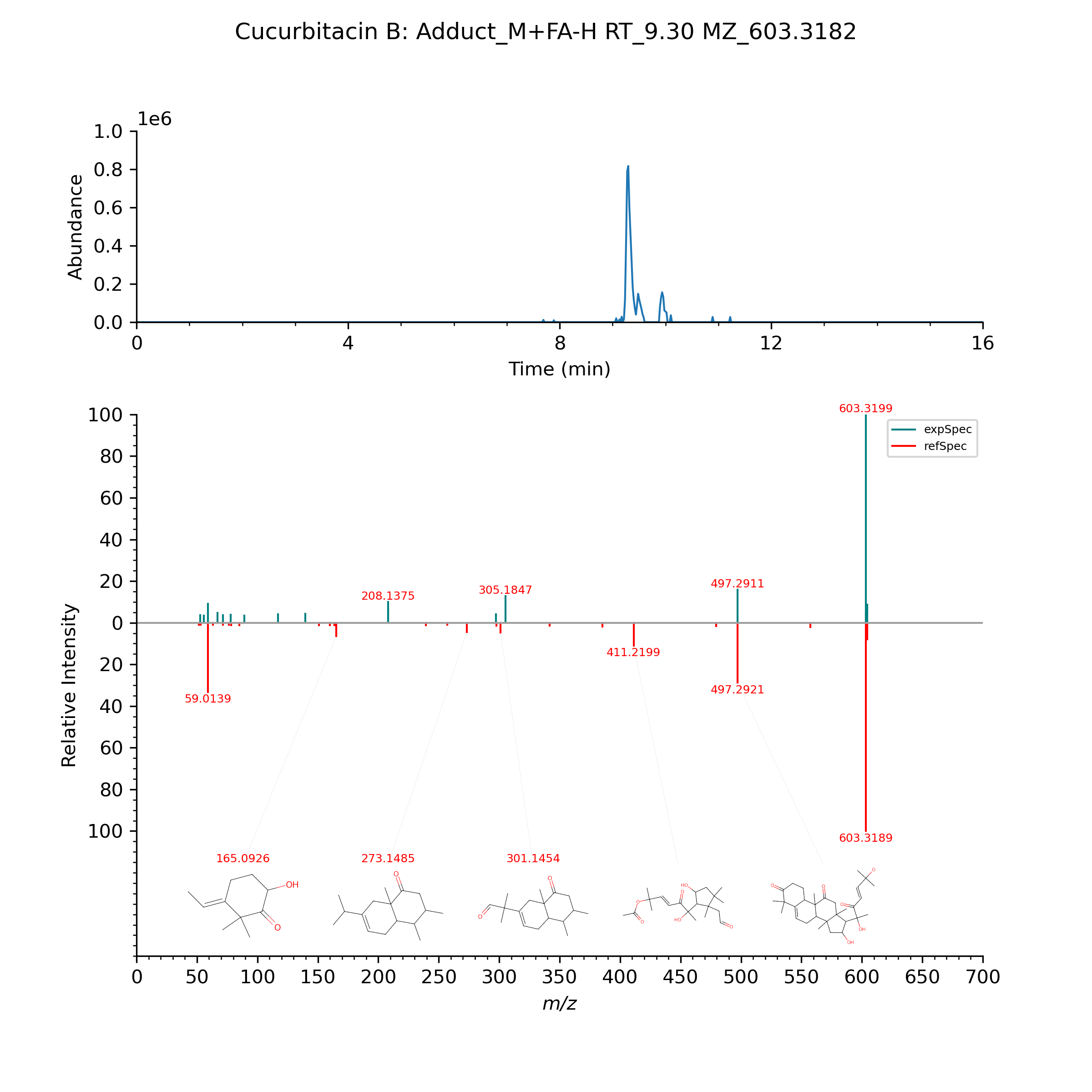

Supplement: Supplementary file 1 [file pharmaceuticals-18-01153-s001.zip › compound structures/M0083.png]

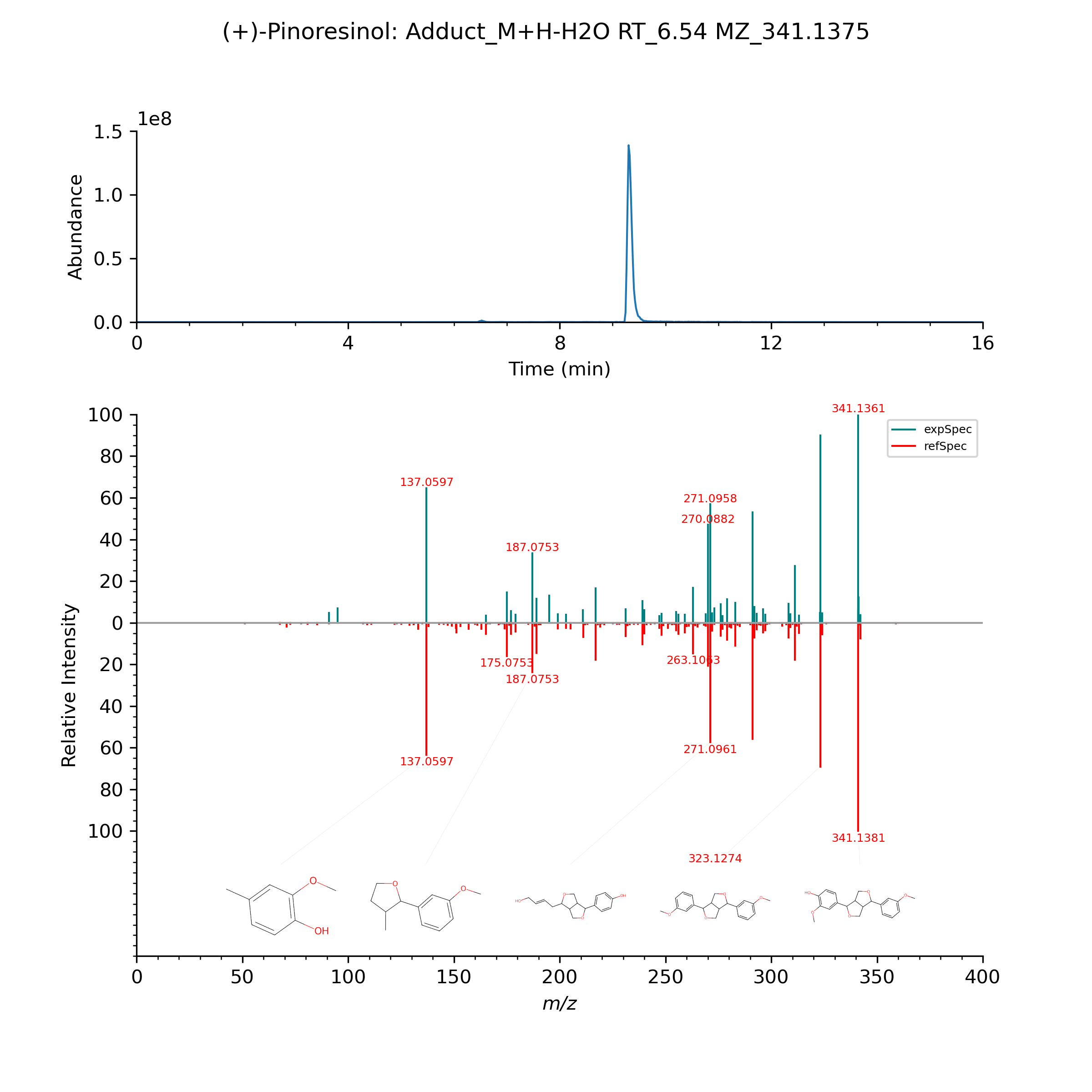

Supplement: Supplementary file 1 [file pharmaceuticals-18-01153-s001.zip › compound structures/M0084.png]

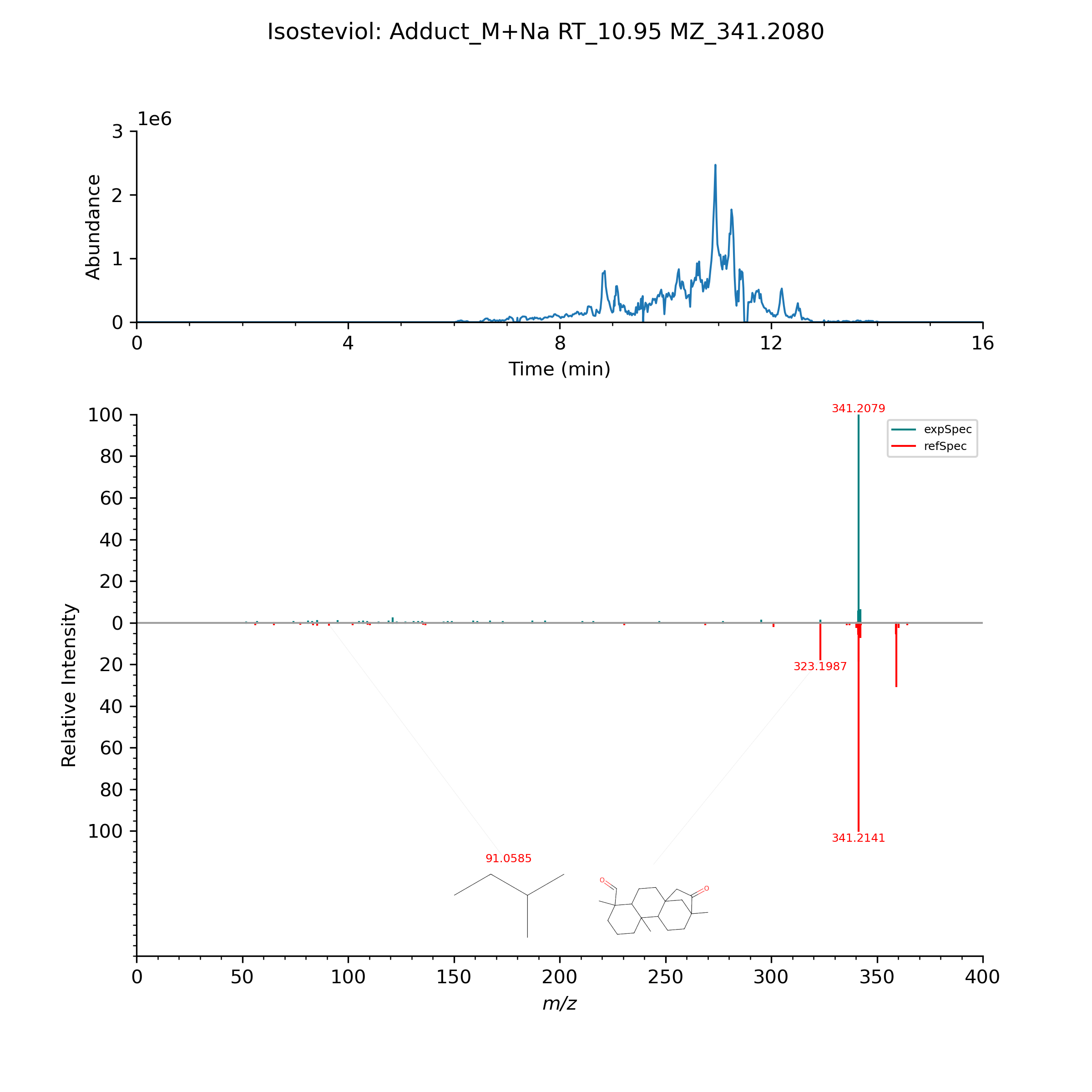

Supplement: Supplementary file 1 [file pharmaceuticals-18-01153-s001.zip › compound structures/M0085.png]

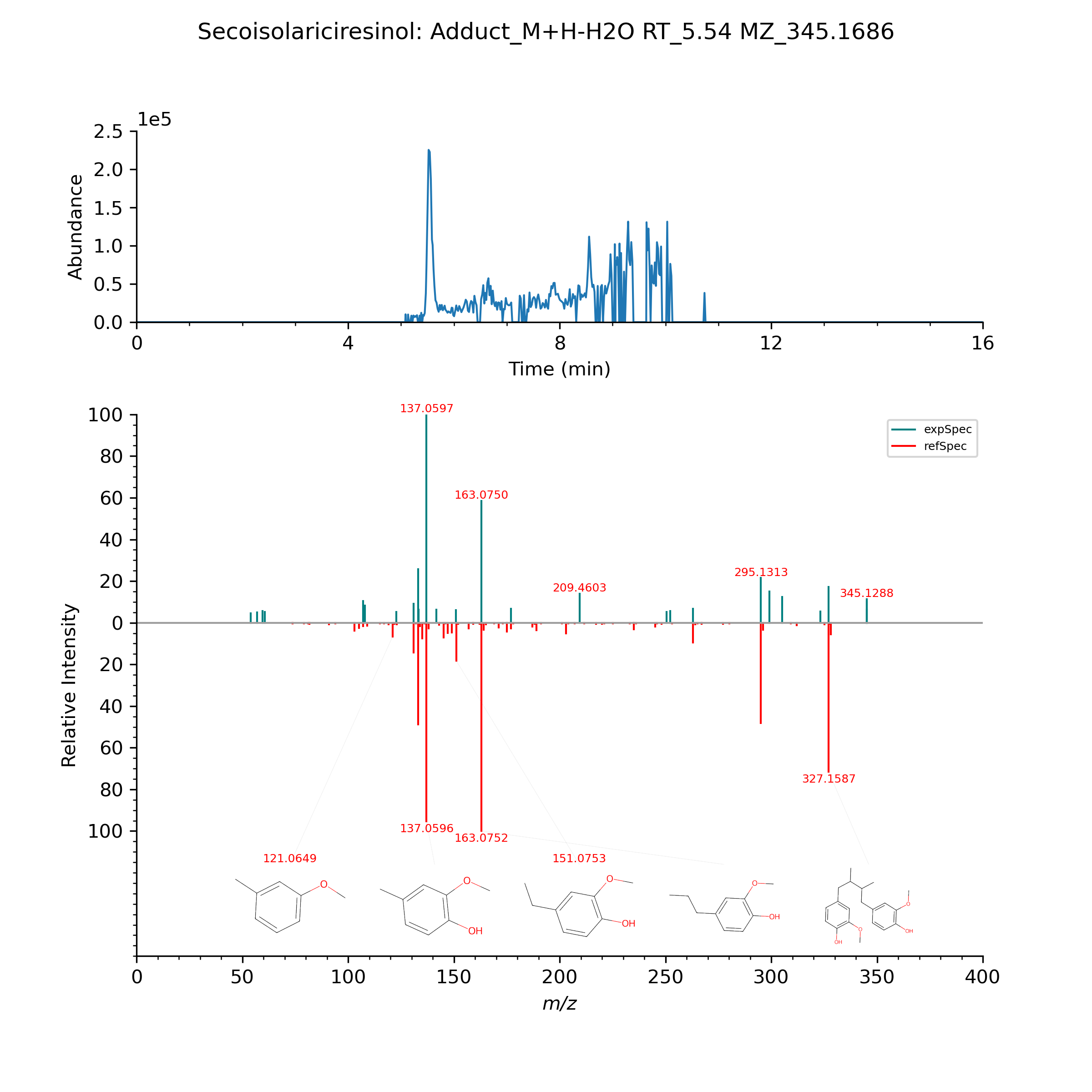

Supplement: Supplementary file 1 [file pharmaceuticals-18-01153-s001.zip › compound structures/M0086.png]

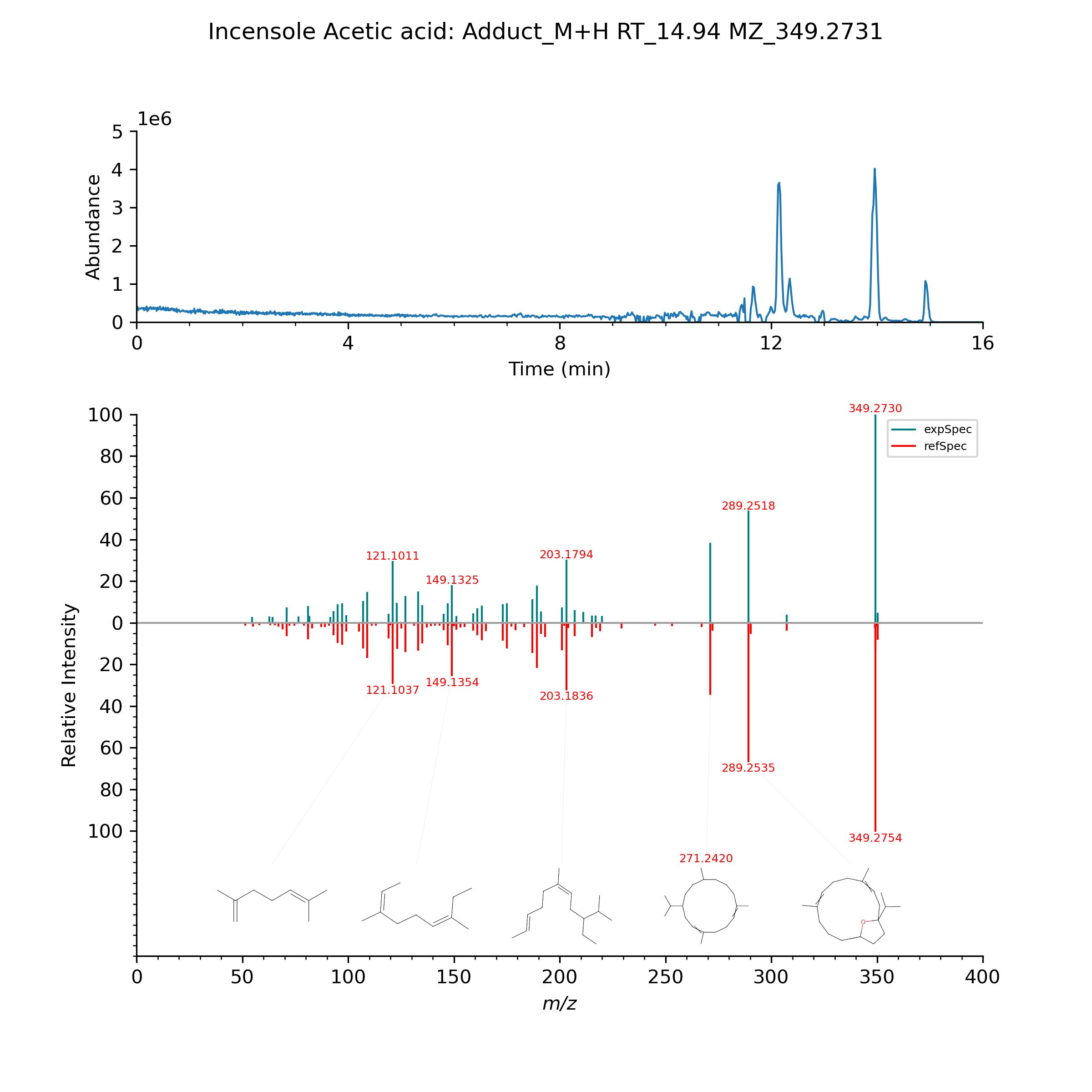

Supplement: Supplementary file 1 [file pharmaceuticals-18-01153-s001.zip › compound structures/M0087.png]

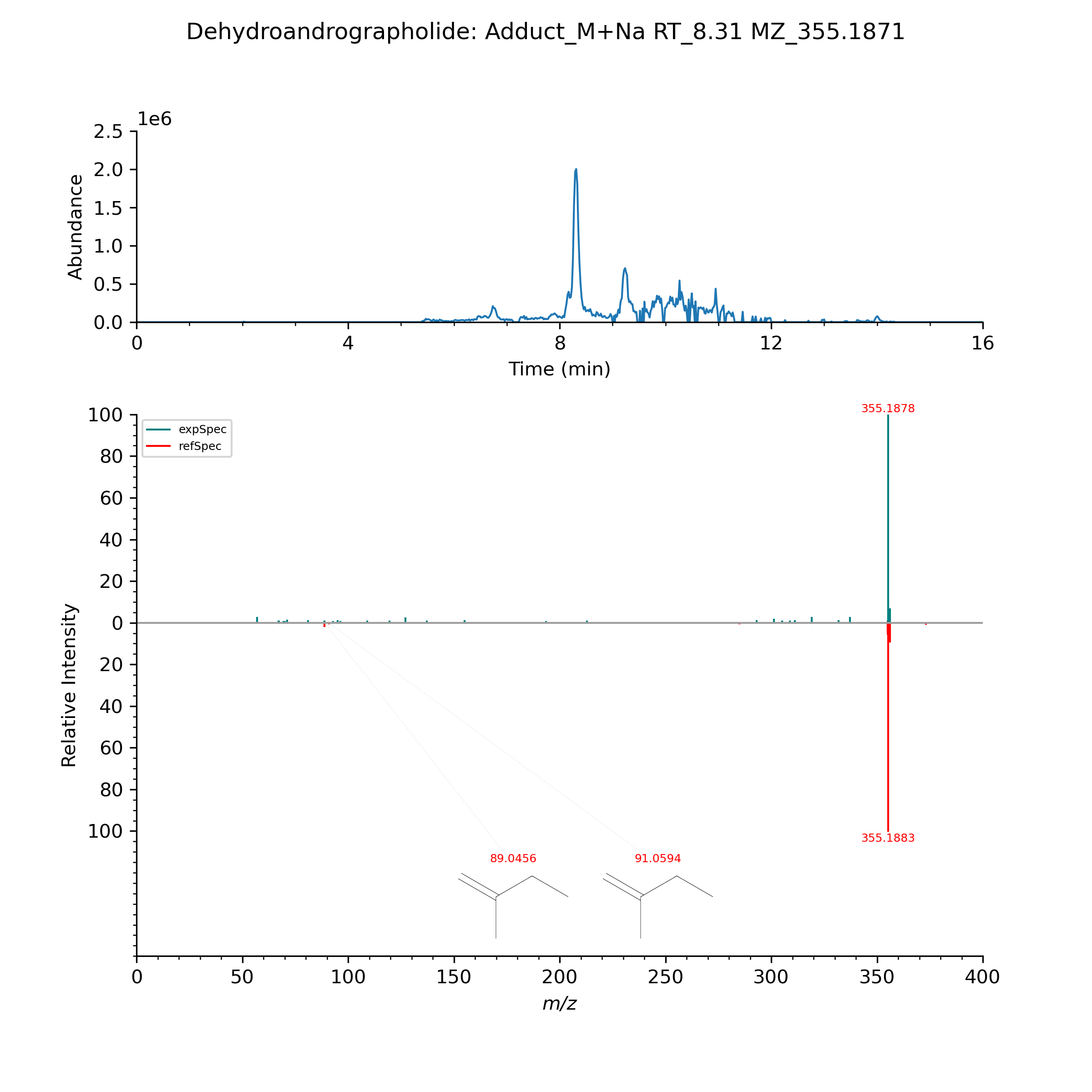

Supplement: Supplementary file 1 [file pharmaceuticals-18-01153-s001.zip › compound structures/M0088.png]

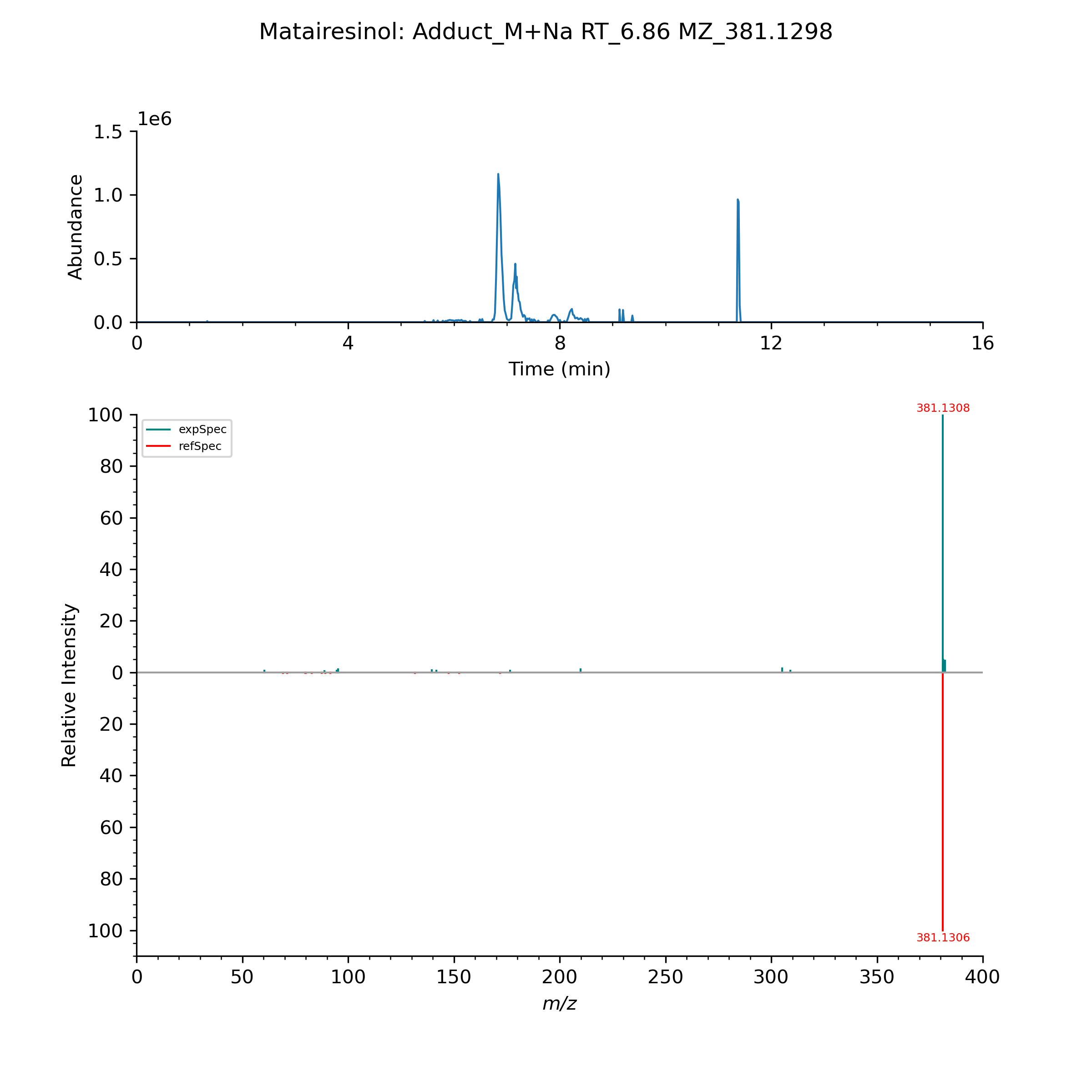

Supplement: Supplementary file 1 [file pharmaceuticals-18-01153-s001.zip › compound structures/M0089.png]

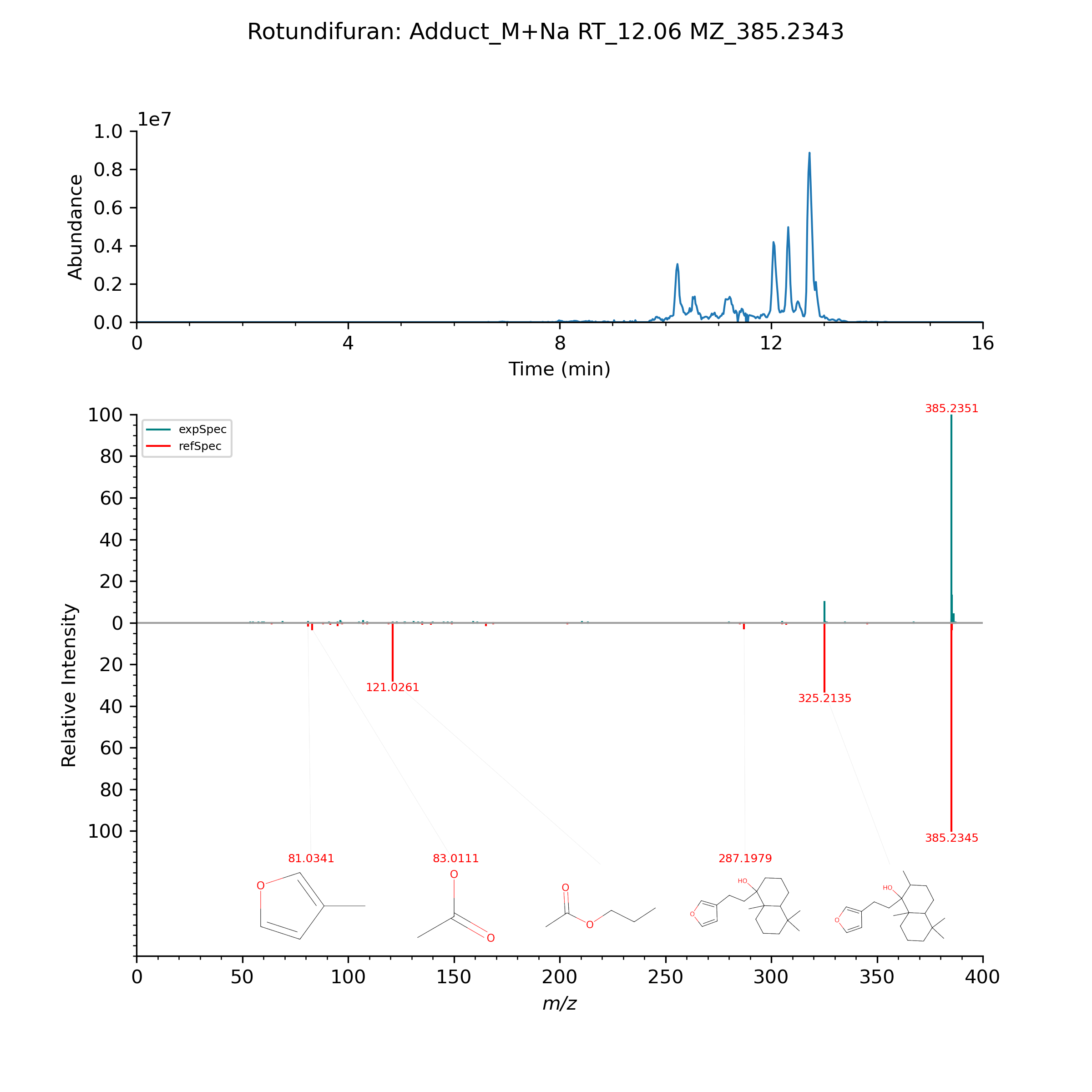

Supplement: Supplementary file 1 [file pharmaceuticals-18-01153-s001.zip › compound structures/M0090.png]

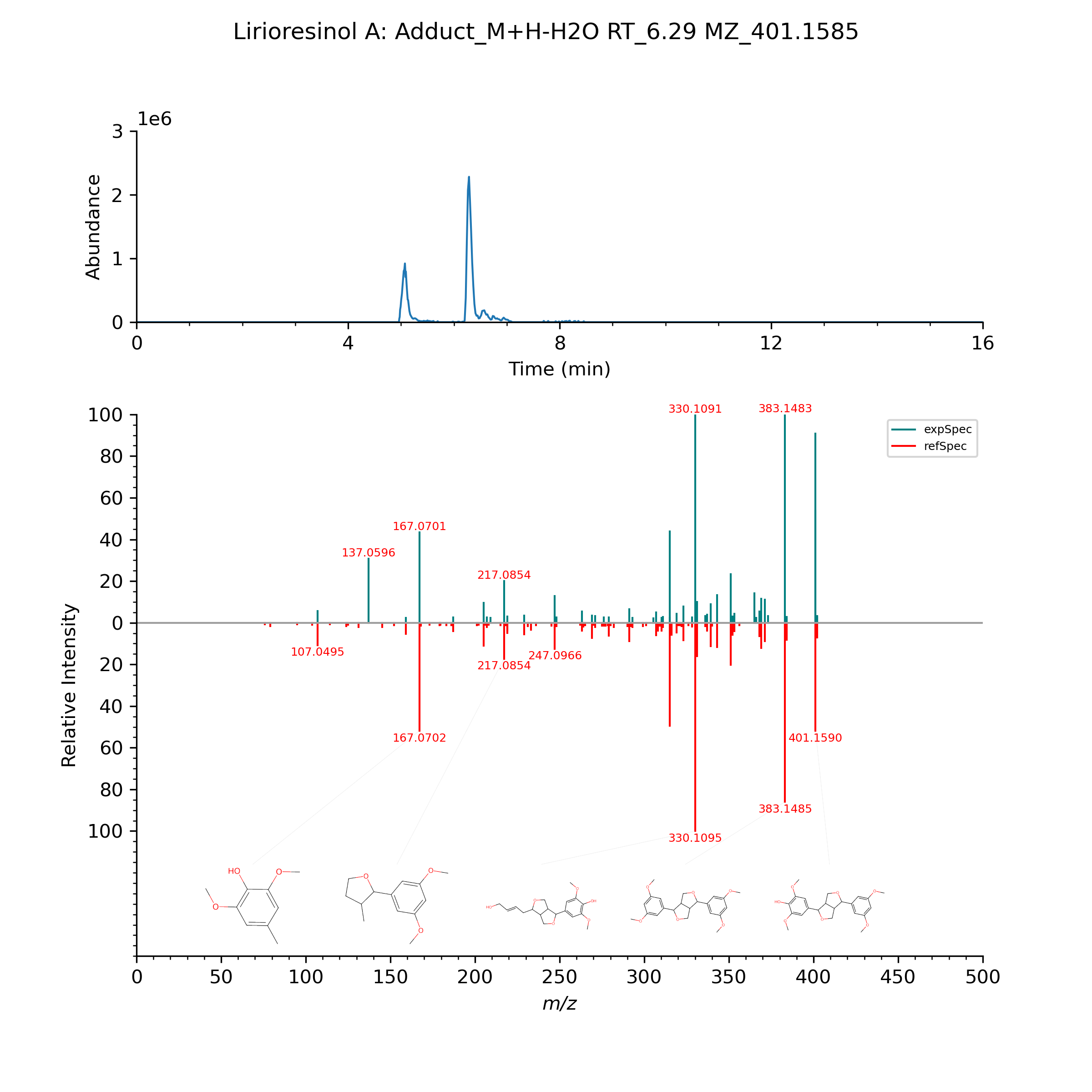

Supplement: Supplementary file 1 [file pharmaceuticals-18-01153-s001.zip › compound structures/M0091.png]

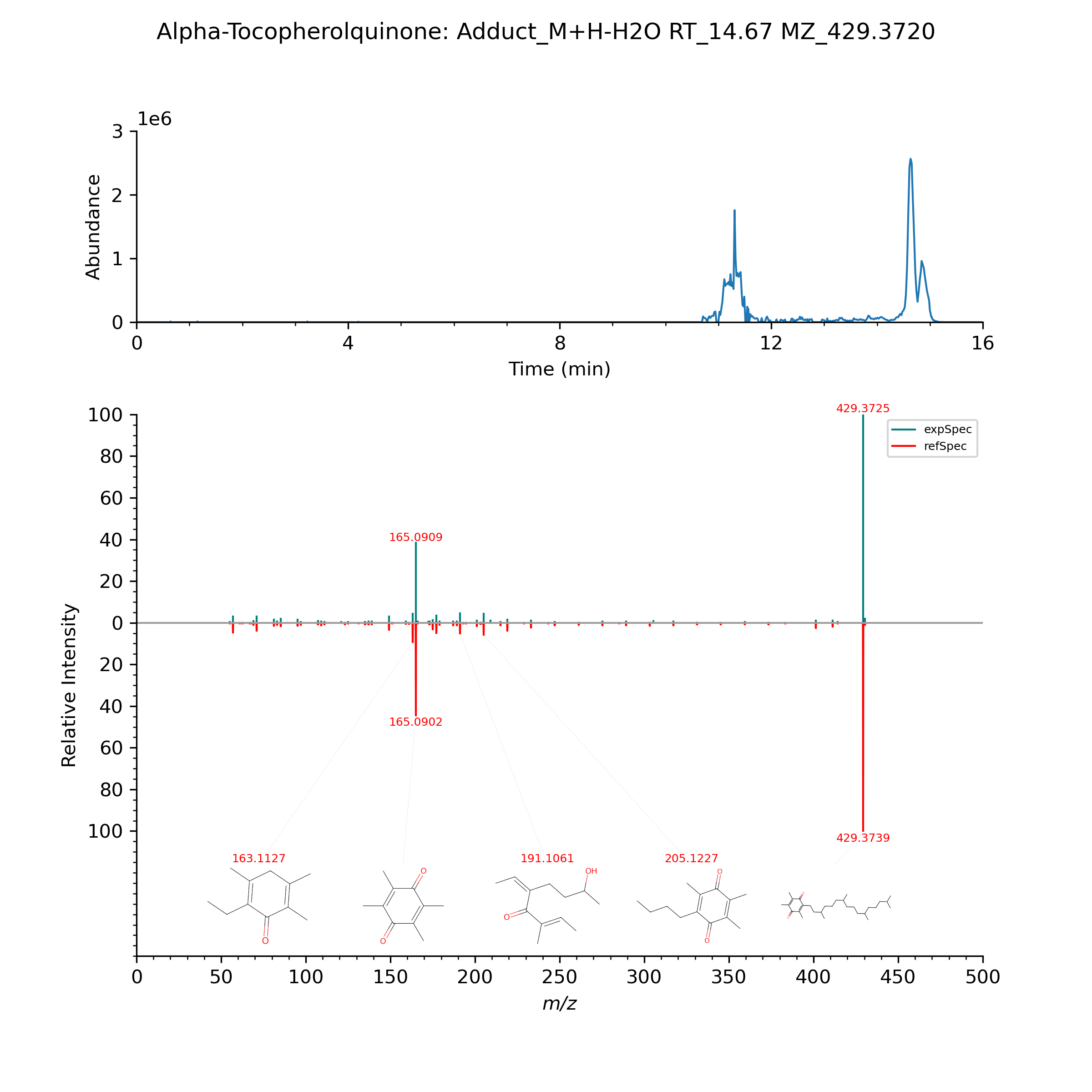

Supplement: Supplementary file 1 [file pharmaceuticals-18-01153-s001.zip › compound structures/M0092.png]

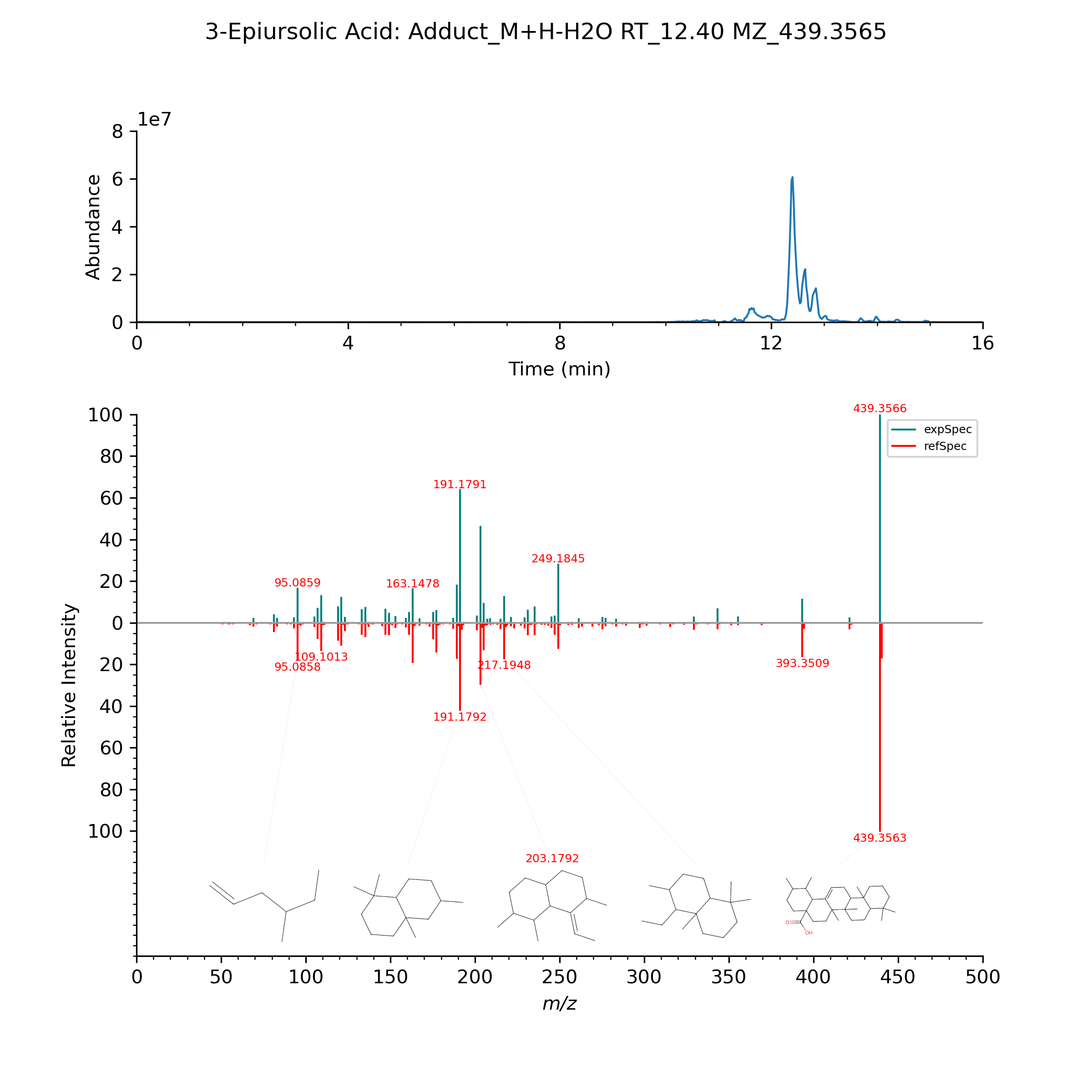

Supplement: Supplementary file 1 [file pharmaceuticals-18-01153-s001.zip › compound structures/M0093.png]

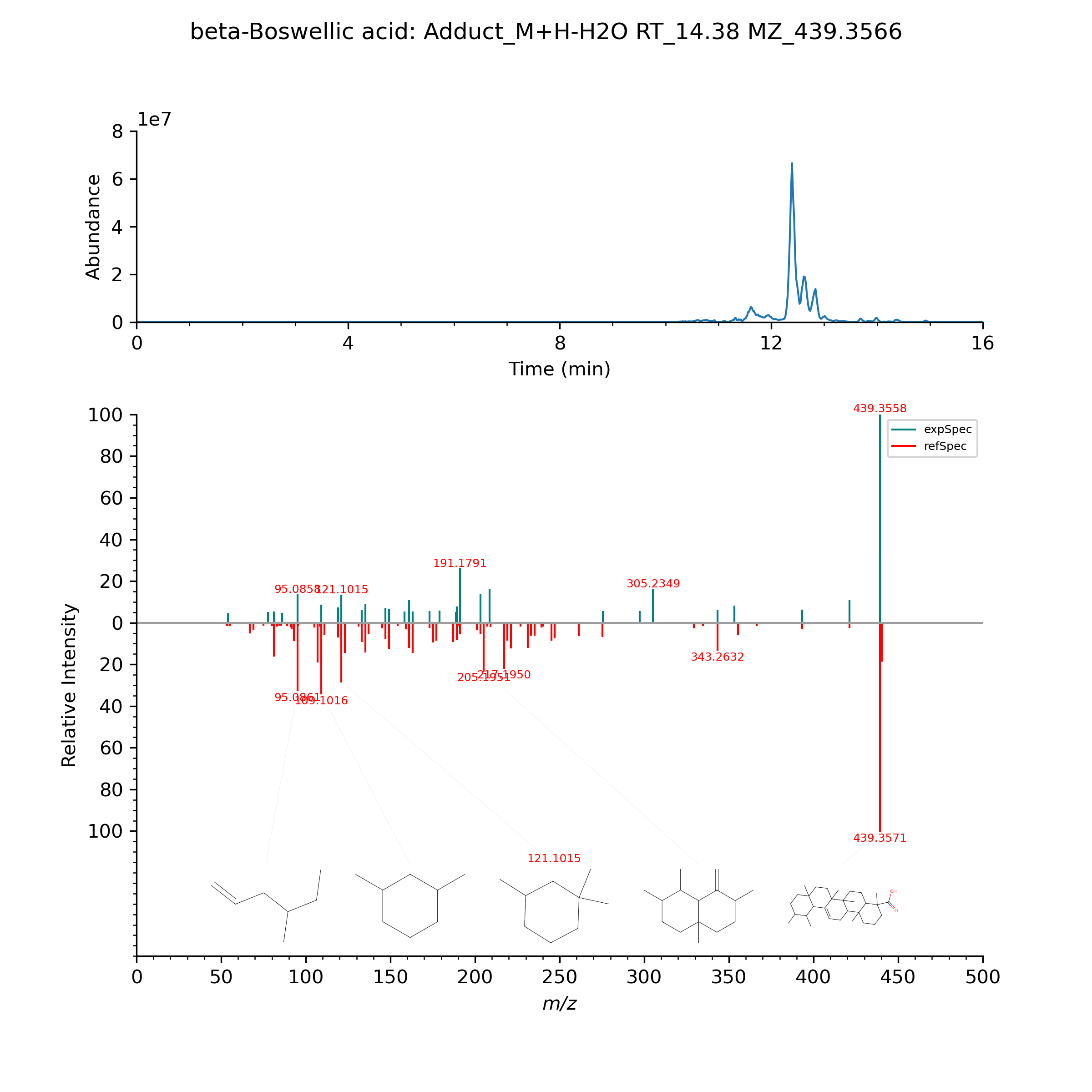

Supplement: Supplementary file 1 [file pharmaceuticals-18-01153-s001.zip › compound structures/M0094.png]

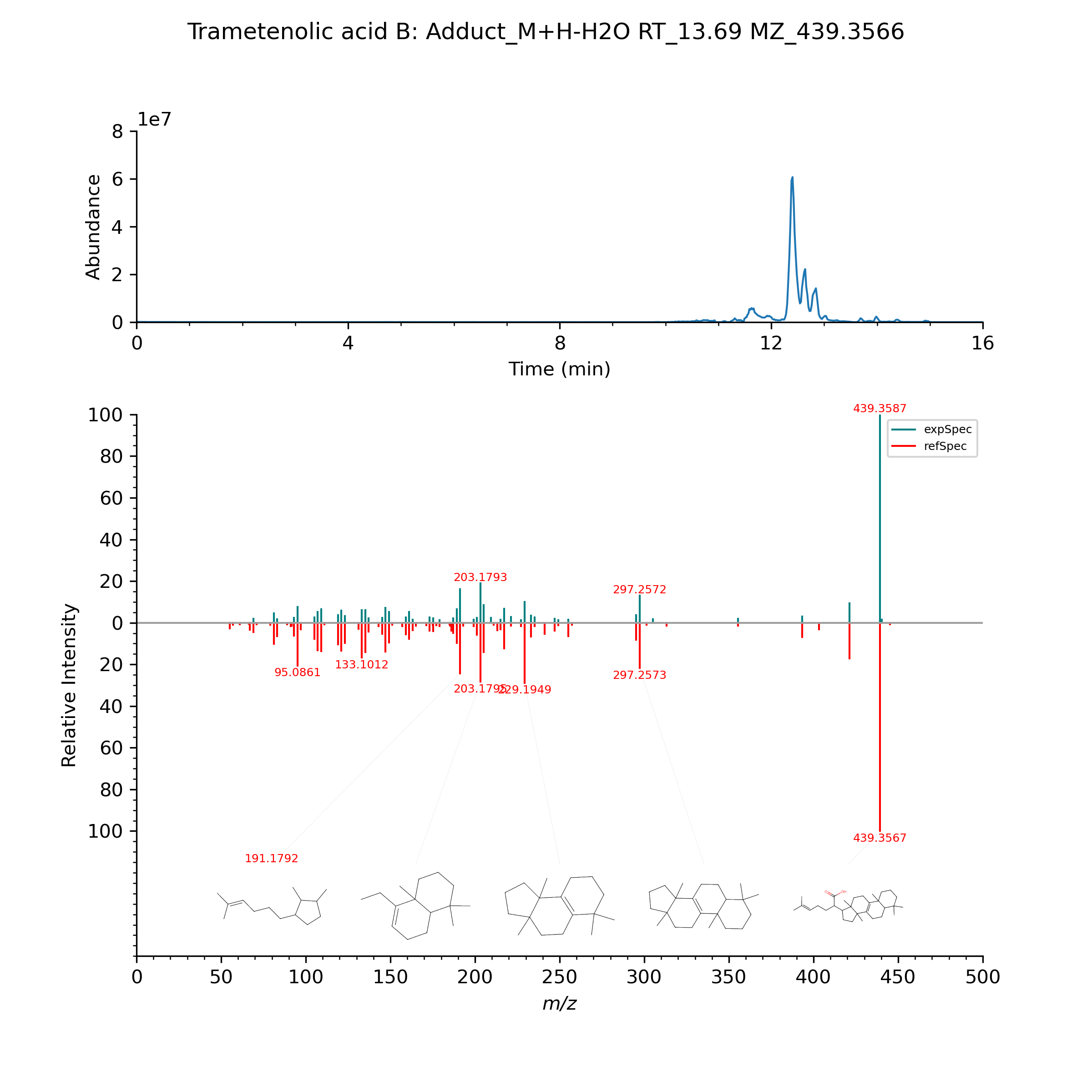

Supplement: Supplementary file 1 [file pharmaceuticals-18-01153-s001.zip › compound structures/M0095.png]

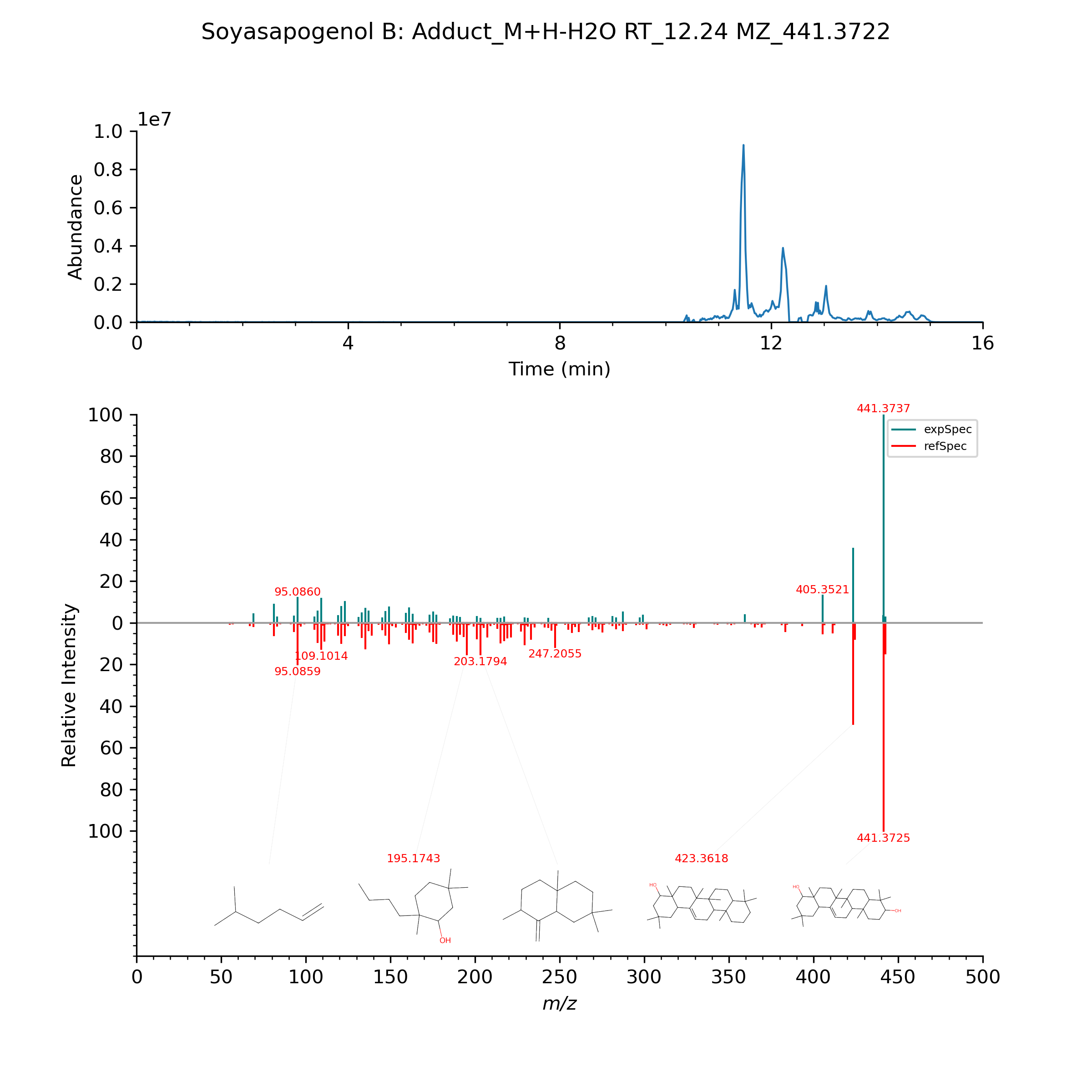

Supplement: Supplementary file 1 [file pharmaceuticals-18-01153-s001.zip › compound structures/M0096.png]

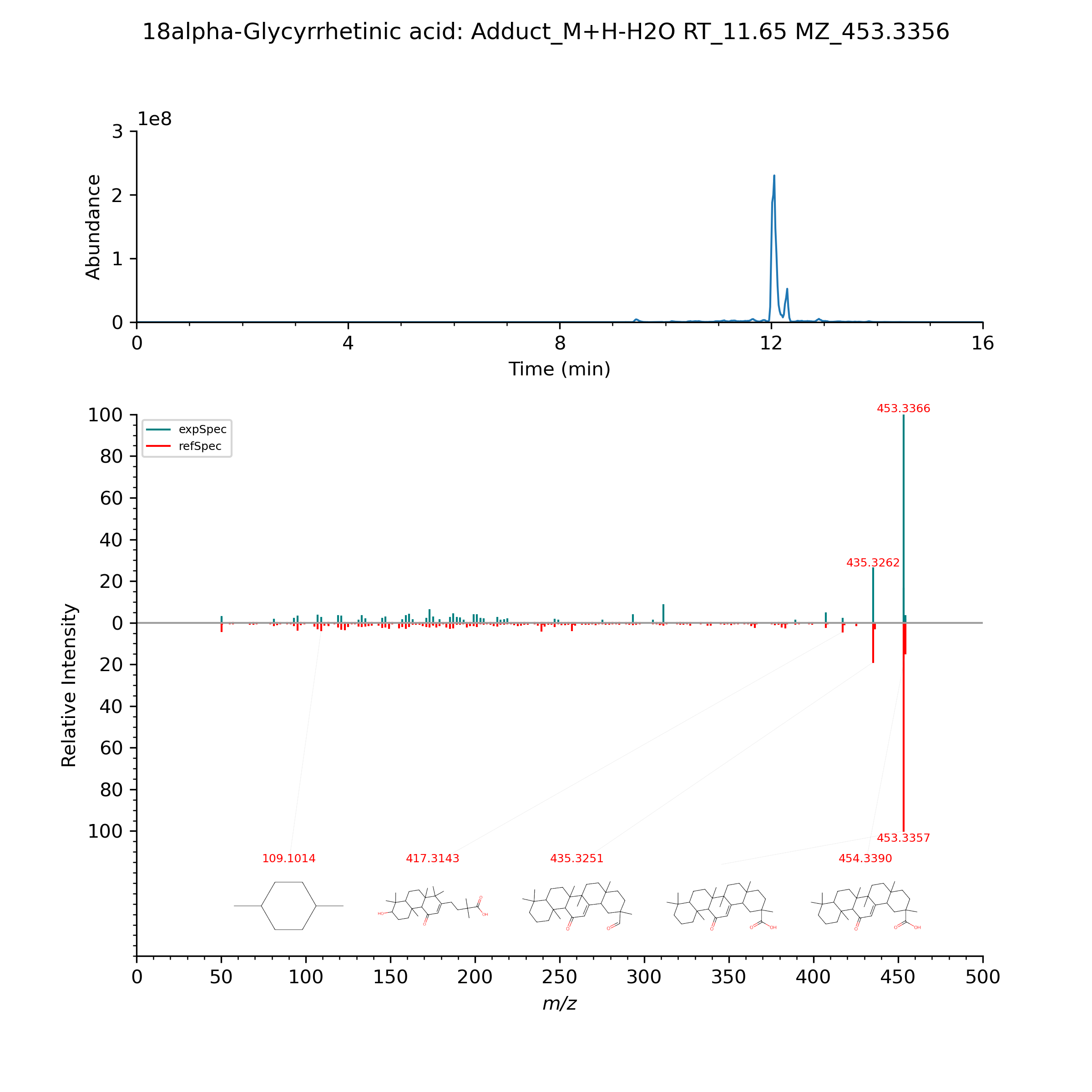

Supplement: Supplementary file 1 [file pharmaceuticals-18-01153-s001.zip › compound structures/M0097.png]

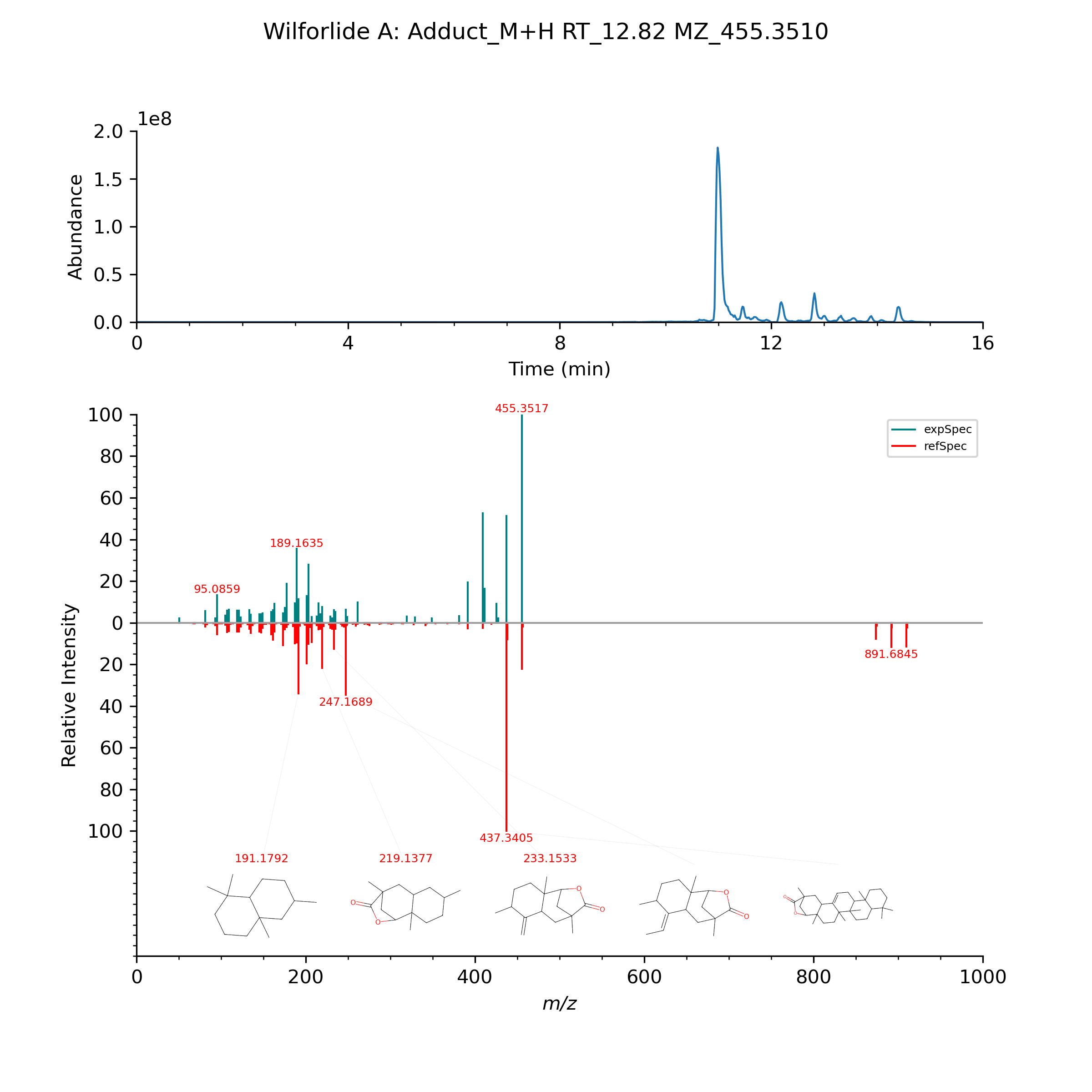

Supplement: Supplementary file 1 [file pharmaceuticals-18-01153-s001.zip › compound structures/M0098.png]

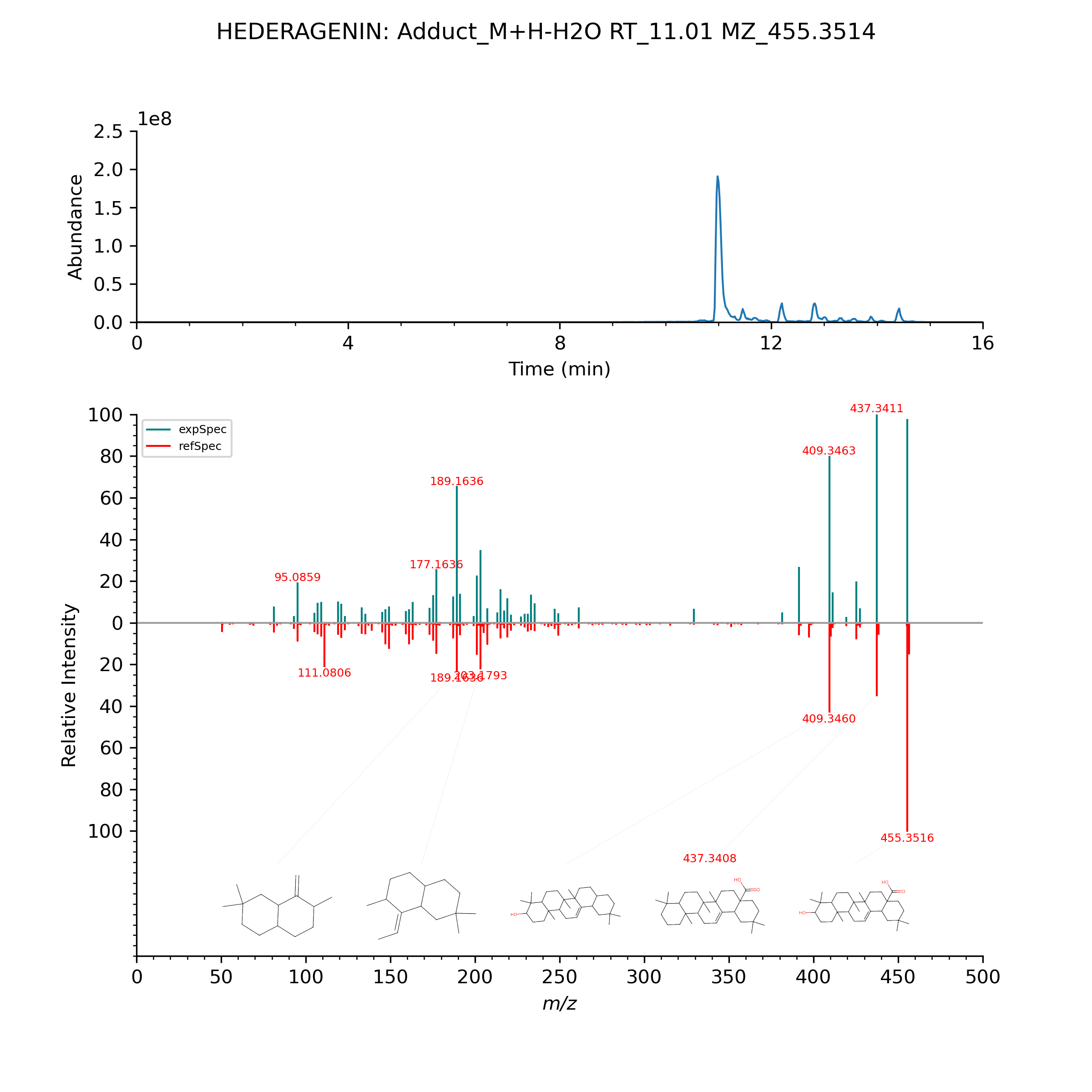

Supplement: Supplementary file 1 [file pharmaceuticals-18-01153-s001.zip › compound structures/M0099.png]

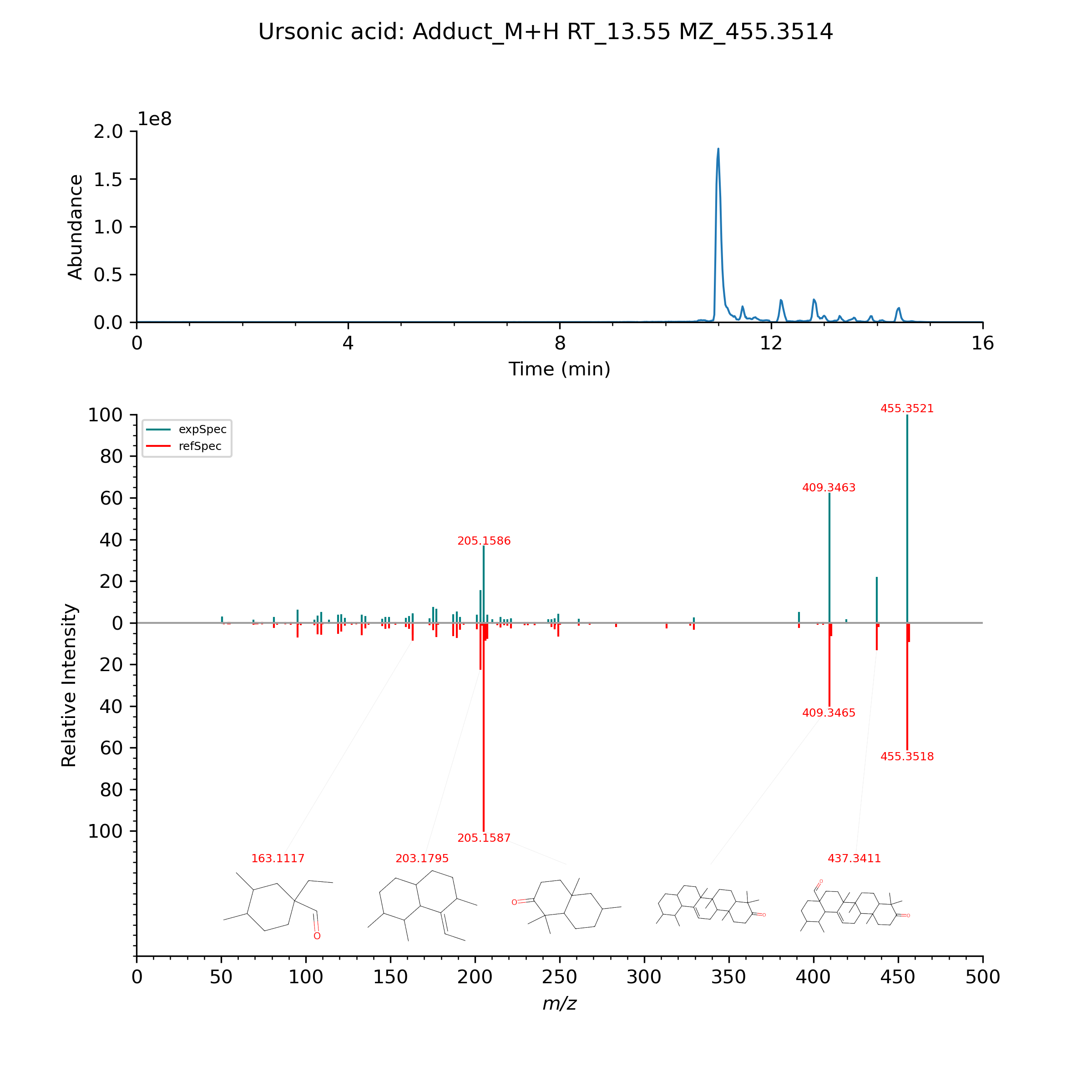

Supplement: Supplementary file 1 [file pharmaceuticals-18-01153-s001.zip › compound structures/M0100.png]
